# Supplementary material for: Protocol for a phase 3 trial to evaluate the effectiveness and safety of a heterologous, two-dose vaccine for Ebola virus disease in the Democratic Republic of the Congo
Source: BMJ Open. 2022 Mar 8;12(3):e055596. doi: 10.1136/bmjopen-2021-055596 (PMC8905941; doi:10.1136/bmjopen-2021-055596)
Supplement: Supplementary data [file bmjopen-2021-055596supp002.pdf]

Protocol for a phase 3 trial to evaluate the effectiveness and safety of a heterologous, two-dose vaccine for Ebola virus disease in the Democratic Republic of the Congo

Appendix 1B

Informed Consent Form in French, Version 7.1 (10 Aug 2020)

Watson-Jones D, *et al. BMJ Open* 2022; 12:e055596. doi: 10.1136/bmjopen-2021-055596

Évaluation de l'efficacité et de l'innocuité de l'emploi d'un vaccin préventif hétérologue à deux doses contre Ebola en République démocratique du Congo

Note d'information et formulaire de consentement éclairé de l'« étude TUJIOKOWE »

Protocole LSHTM : DRC-EB-001

Investigateur principal (IP) :
Professeur Jean Jacques Muyembe
Directeur général
Institut national de recherche biomédicale
Professeur de microbiologie, Faculté de médecine de l'Université de Kinshasa
Kinshasa Gombe, République démocratique du Congo (RDC)
Téléphone : 0898 949 289 ; E-mail : jjmuyembet@gmail.com

Promoteur : London School of Hygiene & Tropical Medicine, Royaume-Uni

Site : République démocratique du Congo

INTRODUCTION

Vous êtes invité(e) à participer à une étude de recherche appelée l'« étude TUJIOKOWE » pour savoir si un nouveau vaccin à deux doses appelé « vaccin Janssen Ebola » peut protéger contre le virus Ebola et pour vérifier si le vaccin est sans danger. L'étude TUJIOKOWE est mise en œuvre par le Ministère de la Santé de la RDC par l'intermédiaire de l'Institut national de recherche biomédicale (INRB), Epicentre et la London School of Hygiene & Tropical Medicine (LSHTM).

QU'EST-CE QUE LE VIRUS EBOLA ?

Ebola est une maladie dont le nom vient du virus Ebola. Lorsqu'une personne contracte le virus Ebola, elle peut parfois tomber très malade, et environ la moitié des personnes qui contractent le virus Ebola meurent. Une fois qu'une personne a contracté le virus Ebola, il peut se propager à d'autres personnes, en particulier si elles vivent sous le même toit.

QU'EST-CE QU'UN VACCIN ET QU'EST-CE QU'UNE ÉTUDE DE RECHERCHE CLINIQUE ?

Les vaccins sont des produits médicaux utilisés pour empêcher d'avoir certaines maladies. De nombreuses personnes sont vaccinées contre la rougeole, la polio et le choléra, par exemple.

Une étude de recherche clinique permet aux personnels de santé de comprendre de nouvelles façons de prévenir ou de traiter une maladie. Dans cette étude, le vaccin est encore sous test.

Les participants à des études de recherche sur les vaccins sont suivis pendant un certain temps afin de recueillir plus d'informations sur le vaccin.

D'AUTRES VACCINS SONT-ILS ADMINISTRÉS POUR PRÉVENIR LE VIRUS EBOLA EN RDC ?

Des vaccinations sont en cours avec un vaccin appelé « VSV » dans plusieurs endroits comme Beni, Katwa et Butembo. Ce vaccin est administré à des personnes à risque de contracter le virus Ebola, comme celles en contact avec une personne infectée par le virus Ebola ou avec des personnes en contact avec celle-ci. Il est également administré à des professionnels de la santé et d'autres travailleurs qui peuvent entrer en contact avec le virus Ebola. Cependant, environ la moitié des personnes infectées par le virus Ebola en RDC ne sont pas connues pour avoir été en contact avec un cas d'Ebola ; nous devons donc les protéger également. Par conséquent, nous cherchons d'autres moyens de protéger les gens contre Ebola, y compris ce vaccin.

EN QUOI CONSISTE CETTE ÉTUDE ?

Cette étude va déterminer si un autre vaccin peut aider à protéger la population contre Ebola. Ce nouveau vaccin est administré en deux injections. La première injection est une dose d'un vaccin appelé Ad26.ZEBOV. La seconde injection est administrée environ deux mois plus tard, avec une deuxième dose appelé MVA-BN-Filo. Dans cette étude, le vaccin « Janssen Ebola » désignera ces deux injections de deux vaccins différents.

Dans le reste de ce document, nous allons parler de vous. Le mot « vous » peut se rapporter à vous ou à votre enfant. Si vous êtes le parent ou le gardien d'un enfant de moins de 18 ans et si vous souhaitez que votre enfant participe à cette étude, vous devrez signer des documents distincts pour vous et votre enfant.

Si votre enfant est âgé de 12 à 17 ans, il/elle devra également accepter de participer à l'étude lui-même/elle-même et signer un autre document, appelé formulaire d'assentiment.

QUE SAVONS-NOUS DU VACCIN ?

Le vaccin Janssen Ebola est conçu pour protéger la population contre Ebola. **Vous ne pouvez pas contracter le virus Ebola par le vaccin.** Plus de 6 000 personnes ont reçu le vaccin dans de nombreux pays, dont 7 en Afrique. Jusqu'à présent, le vaccin est sûr et bien toléré chez les personnes vaccinées. Le vaccin pourrait aider le corps à lutter contre les infections à virus Ebola, mais nous ne savons pas encore avec certitude si le vaccin Janssen Ebola peut protéger la population contre Ebola.

QUI PEUT PARTICIPER À CETTE ÉTUDE ?

Pour participer à cette étude, vous devez vivre ou travailler au sein de l'une des communautés qui recevront le vaccin Janssen Ebola. Vous devez être en mesure de revenir pour recevoir la deuxième dose de vaccin et être disposé(e) à respecter les exigences nécessaires pour prendre part à cette étude. Les participants doivent être en bonne santé afin de recevoir chaque dose du

vaccin. Les personnes avec un diagnostic de VIH (qui sont en bonne santé), les femmes enceintes et allaitantes et les enfants âgés de 1 an ou plus peuvent être inclus dans cette étude.

Vous ne pouvez pas participer à cette étude si vous avez contracté la MVEbola.Vous ne pouvez pas non plus participer à cette étude si vous êtes allergique aux œufs ou aux produits contenant des œufs. Si vous avez été déjà vacciné contre la maladie à virus Ebola ou un autre vaccin de routine durant les 30 derniers jours ou si vous avez une fièvre de 38 °C ou plus le jour de la vaccination, vous devrez revenir un autre jour. Vous pouvez recevoir le vaccin si vous avez un léger rhume, de la toux ou une diarrhée légère.

SUIS-JE OBLIGÉ(E) DE PARTICIPER À CETTE ÉTUDE ?

Vous n'êtes pas obligé(e) de participer à cette étude si vous ne le souhaitez pas. Si vous décidez que vous ne souhaitez pas participer à l'étude, cela ne vous affectera en aucune façon. Si vous ne souhaitez pas participer à l'étude, mais que vous changez d'avis plus tard, vous pouvez participer à l'étude lorsque l'équipe de vaccination revient dans votre communauté. Après cette deuxième visite au sein de votre communauté, il peut ne plus être possible de participer à l'étude pour recevoir le vaccin Janssen Ebola. Vous devez être inclus dans l'étude pour recevoir le vaccin.

QUE SE PASSERA-T-IL SI J'ACCEPTE DE PARTICIPER À CETTE ÉTUDE ?

Nous allons décrire l'étude et répondre à toutes les questions que vous pourriez avoir. Si vous souhaitez une copie de cette note d'information, nous vous la donnerons. L'un des membres du personnel de l'étude vous posera des questions au sujet de l'étude pour vérifier que vous avez compris l'information. Nous vous demanderons de signer ou d'apposer votre empreinte digitale sur ce formulaire de consentement éclairé. En mettant votre nom ou en apposant votre empreinte digitale sur le formulaire de consentement, vous acceptez de participer à l'étude, mais vous pouvez changer d'avis et décider de quitter l'étude à tout moment.

QU'IMPLIQUE L'ÉTUDE ?

Après avoir signé ce formulaire de consentement, on vous posera quelques questions pour vérifier comment vous vous sentez. Si le médecin décide que vous pouvez être vacciné(e), vous recevrez une injection du premier vaccin Janssen Ebola contre Ebola dans la partie supérieure de votre bras ou dans la cuisse chez les jeunes enfants. Nous vous donnerons une carte de vaccination avec le numéro de téléphone de personnes à contacter en cas de besoin. La carte indiquera également la date à laquelle vous devrez revenir pour la deuxième dose de vaccin Janssen Ebola. Votre numéro de téléphone vous sera demandé, il est possible que vous soyez contacté afin de vous rappeler le rendez-vous de l'administration du second vaccin et également pour assurer un suivi.

Afin de vous identifier lors de la visite suivante, nous allons prendre une photographie de votre visage. Nous le faisons afin de savoir qui a été vacciné et veiller que vous receviez la deuxième dose au bon moment.

Vous serez invité(e) à vous présenter à une date spécifique pour le second vaccin Janssen Ebola. Après la deuxième dose, nous vous demanderons de nous appeler si vous avez des problèmes

médicaux dans les 30 jours après votre deuxième dose de vaccin. Vous devrez signaler toute maladie à l'équipe de l'étude dans les 30 jours en contactant le numéro de téléphone indiqué dans votre carte de vaccination. Nous veillerons à ce que des téléphones soient disponibles au sein de votre communauté.

Un certain nombre de participants seront inclus dans un groupe de suivi rapproché qui sera fait par téléphone ou par visite à domicile.

Suite à la pause de l'étude due à la COVID-19, certains participants ont vu leur deuxième dose de vaccin Janssen Ebola reportée. Ces participants seront appelés à recevoir leur deuxième dose à une date ultérieure. Le fait de recevoir le second vaccin Janssen Ebola à une date ultérieure n'aura pas d'effets négatifs sur la santé de ces participants.

QUELS SONT LES ÉVENTUELS RISQUES LIÉS À LA PARTICIPATION À CETTE ÉTUDE ?

Nous ne savons pas encore avec certitude si le vaccin Janssen Ebola protège la population contre Ebola. Il existe donc un risque que vous ne soyez pas protégé(e) contre le virus Ebola, même après la vaccination.

Ce vaccin a été administré à plus de 6 000 personnes dans d'autres études de recherche. Certains des effets secondaires que vous pourriez avoir après les injections sont les suivants :

- Douleur, gonflement, démangeaison, rougeur ou sensation de chaleur autour de la zone où vous avez reçu l'injection.
- Autour du site de l'injection, vous pourriez ressentir de la douleur lors de vos mouvements pendant quelques jours.
- Vous pourriez remarquer des bosses gonflées sous vos bras ou dans votre nuque pendant quelques jours.
- Vous pourriez avoir de la fièvre ou des maux de tête, une sensation de fatigue, des frissons, une éruption cutanée, des nausées, une modification de l'appétit, des vomissements, de la diarrhée, des étourdissements, des douleurs musculaires, articulaires ou au ventre, des plaies dans la bouche ou des courbatures.

Dans les études de recherche précédentes, tout le monde n'a pas eu ces effets secondaires après les injections. Les effets secondaires sont généralement légers et la plupart ne durent que quelques jours. Chez certaines personnes, ils peuvent durer pendant quelques semaines, très rarement plus longtemps. Certaines personnes ont signalé qu'elles avaient des picotements dans les mains et les pieds, ou une faiblesse musculaire. Généralement, ces symptômes ne duraient qu'un jour ou deux, mais parfois ils perduraient pendant plusieurs semaines. Une personne a signalé des picotements, un engourdissement et une douleur qui ont duré pendant plusieurs mois et interféré sur ses activités quotidiennes.

Nous avons appris que trois femmes de moins de 40 ans vivant dans d'autres pays ont présenté de graves caillots sanguins dans les trois semaines suivant l'administration d'un vaccin fabriqué de manière similaire au deuxième vaccin Janssen Ebola. L'une d'entre elles a eu un accident vasculaire cérébral et est décédée. Un homme de 50 ans a également signalé un manque de flux sanguin dans la jambe. Ces cas présentaient également des conditions médicales connues pour augmenter le risque de caillots sanguins et aucun de ces événements

n'a été considéré comme lié au vaccin. Le fait de recevoir le vaccin n'augmentait pas les risques d'avoir ces problèmes.
Des centaines de bébés (âgés de 1 à 3 ans), d'enfants (âgés de 4 à 11 ans) et d'adolescents (âgés de 12 à 17 ans) ont reçu le vaccin Janssen Ebola jusqu'à présent. Les effets secondaires signalés chez les enfants et les adolescents après la vaccination étaient similaires à ceux signalés par les adultes. La douleur au point d'injection était l'effet secondaire local le plus fréquemment signalé. Des maux de tête, de la fatigue et des frissons ont également été fréquemment signalés chez les enfants et les adolescents plus âgés. Une diminution de l'appétit, une baisse d'activité et de la fièvre ont été les effets secondaires les plus fréquemment signalés chez les bébés. Après la vaccination, quelques enfants ont eu une fièvre supérieure à 39ºC. La plupart de ces effets secondaires étaient légers à modérés et n'ont pas duré plus d'un ou deux jours. Aucun problème médical grave n'a été observé chez les enfants qui ont reçu le vaccin. Les effets secondaires chez les enfants sont similaires à ceux qui se produisent avec d'autres vaccins chez les enfants.

L’équipe médicale vous fournira fournir des soins si vous ressentez des effets secondaires liés aux injections jusqu’au un mois après la seconde dose. Elle est disponible à tout moment si vous avez des problèmes ou des questions.

QUELS SONT LES AVANTAGES LIÉS À LA PARTICIPATION À L’ÉTUDE ?

Les jours de vaccination, un médecin vous posera des questions pour vérifier votre état de santé ; si nécessaire, vous recevrez les soins nécessaires ou serez dirigé vers un spécialiste pour des affections médicales simples comme le paludisme. Vous apprendrez aussi comment vous protéger contre le virus Ebola. Votre participation à cette étude contribuera au développement de vaccins pour prévenir le virus Ebola et, à l’avenir, pourrait aider des personnes dans différentes régions du monde.

GROSSESSE ET L’ALLAITEMENT PENDANT L’ÉTUDE ?

Vous pouvez prendre part à cette étude si vous êtes enceinte ou si vous allaitez. Nous ne sommes pas sûr de tous les effets des vaccins de l’étude pendant la grossesse ou chez un bébé allaité. Si vous savez que vous êtes enceinte ou si vous allaitez au moment de la vaccination, informez-en le personnel de l’étude. Si vous n’êtes pas certaine, nous vous proposerons un test de grossesse et un entretien avec du personnel médical qui vous informera si vous etes emceinte avant chaque vaccination. Si vous êtes enceinte, ou si vous tombez enceinte dans le mois qui suit l’administration d’une des deux doses, vous serez contactee durant votre grossesse afin de vous poser des questions concernant votre sante et celle votre bebe. Dans ce cas, nous vous contacterons pendant votre grossesse pour vous poser des questions sur votre état de santé et celui de votre bébé. Nous vous contacterons également dans les 3 mois après votre accouchement. Si nous ne pouvons pas vous contacter par téléphone après votre accouchement, l’équipe de l’étude vous rendra visite à votre domicile. Parmi les femmes enceintes dans l’étude, certain nombre de participante seront invitée à faire partie d’un sous-groupe pour assurer un suivi plus rapproché par téléphone ou au domicile. Certains nouveaux-nés de maman faisant partie du sous-groupe seront également suivis dans les 3 mois suivant la naissance
Si vous tombez enceinte dans le mois qui suit une vaccination, merci d’appeler le numéro de téléphone indiqué sur votre carte de vaccination et de prévenir l’équipe de l’étude.

QUE DOIS-JE FAIRE POUR MA PROPRE SANTÉ PENDANT CETTE ÉTUDE ?

Si vous tombez malade pendant l'étude, vous devez vous rendre dans l'un des établissements de santé recommandés qui sont indiqués sur la liste que nous vous donnerons. . Vous devrez également appeler le numéro de téléphone de la personne de contact de l'étude indiquée sur votre carte de vaccination et lui dire si vous présentez un problème médical, jusqu'à 30 jours après l'administration de la deuxième dose du vaccin. Vous pourrez être orienté(e) vers des soins médicaux, si nécessaire.

Nous ne savons pas encore avec certitude si le vaccin Janssen Ebola peut protéger contre Ebola. Si vous recevez le vaccin, vous pourriez être protégé(e) contre le virus Ebola, mais il est possible que vous ne soyez pas protégé(e). **Par conséquent, vous devez continuer à éviter les contacts avec le virus Ebola.** Nous vous donnerons des informations spécifiques sur la prévention du virus Ebola, et n'hésitez pas à contacter un membre de l'équipe de l'étude si vous avez des questions sur la façon d'éviter les contacts avec le virus Ebola. Si vous présentez des symptômes qui ressemblent au virus Ebola, comme de la fièvre, de la diarrhée, des vomissements ou des saignements inexpliqués, il est très important que vous informiez l'équipe de l'étude que vous êtes malade et que vous receviez des soins médicaux dès que possible.

Si nous découvrons quelque chose de nouveau pendant cette étude qui pourrait être lié à votre santé ou influencer votre décision de poursuivre l'étude, nous vous en parlerons.

QUI POURRA CONSULTER MES INFORMATIONS ?

Nous préserverons la confidentialité de vos informations de l'étude.
Le personnel de l'étude, y compris des personnes en dehors du pays chargées de vérifier le bon déroulement de cette étude, les membres des comités d'éthique et les autorités réglementaires, peuvent consulter vos dossiers médicaux. Tous les membres du personnel de l'étude sont formés à maintenir la confidentialité et ne supprimeront aucun document avec votre nom dans les dossiers de l'étude. Toutes ces personnes comprennent qu'elles doivent garder votre identité confidentielle et qu'elles ne partageront votre nom ou vos coordonnées avec personne.

Une description de cette étude sera disponible sur le site internet http://www.ClinicalTrials.gov. Ce site internet ne contiendra aucune information permettant de vous identifier. Vous pouvez consulter ce site internet à tout moment.

Les résultats de l'étude seront partagés avec la population, mais aucune information personnelle ne sera mentionnée dans les résultats de l'étude. Si vous avez des questions spécifiques sur vos données, contactez l'investigateur principal de l'étude, le Professeur Jean Jacques Muyembe ou son représentant (voir les coordonnées ci-dessous).

QUI VÉRIFIE QUE L'ÉTUDE EST SANS DANGER ?

Cette étude est mise en œuvre par des partenaires, comme le Ministère de la Santé de la RDC par l'intermédiaire de l'Institut national de recherche biomédicale (INRB) avec l'aide d'Epicentre et de la London School of Hygiene & Tropical Medicine (LSHTM). Un comité

Watson-Jones D, *et al. BMJ Open* 2022; 12:e055596. doi: 10.1136/bmjopen-2021-055596

indépendant composé de médecins et d'experts qui ne sont pas directement impliqués dans l'étude examinera les informations de l'étude et vérifiera l'innocuité des vaccins.

À QUI PUIS-JE PARLER DE CETTE ÉTUDE ?

Si vous souhaitez parler à quelqu'un à propos de cette étude ou si vous pensez avoir subi un préjudice en lien avec votre participation à l'étude, vous pouvez contacter les personnes suivantes.

1) L'investigateur principal responsable de cette étude

Professeur Jean Jacques Muyembe<br>*Directeur général,* Institut national de recherche biomédicale,<br>Kinshasa, RDC<br>Téléphone : 0898 949 289<br>E-mail : jjmuyembet@gmail.com<br><br>Représentant locale de l'étude<br>Dr Hugo Kavunga<br>Téléphone: 0823 875 153<br>E-mail : hugokavunga@gmail.com

2) Les comités d'éthique de la RDC qui ont approuvé cette étude

Professeur Félicien Munday<br>Comité national d'éthique et de la santé<br>Kinshasa-Gombe, RDC<br>Téléphone : 0998 419 816<br>E-mail : feli1munday@yahoo.fr
Professeur Willy Bongopasi<br>Comité d'éthique de l'école de santé publique<br>Université de Kinshasa, RDC<br>Téléphone: 0999 952 341<br>E-mail : bongopasi@gmail.com

Si vous avez des questions concernant l'étude TUJIOKOWE ou sur vos droits en tant que participant(e) à l'étude, vous pouvez les poser à un membre de l'équipe de l'étude à tout moment.

FORMULAIRE DE CONSENTEMENT DESTINÉ AU/À LA PARTICIPANT(E) OU AU PARENT/TUTEUR

Titre : Étude TUJIOKOWE

Investigateur principal de cette étude : Prof. JJ Muyembe

**Déclarations**	**Signez ou apposez votre empreinte digitale dans chaque case**
J'ai lu les informations contenues dans ce formulaire concernant l'étude TUJIOKOWE (ou le personnel de l'étude m'a expliqué ces informations avec des mots que je comprends). Le but de cette étude et les procédures pour recevoir ce nouveau vaccin m'ont été expliqués en détail. J'ai pu poser des questions et j'ai reçu des réponses satisfaisantes à toutes mes questions.	
Je comprends que ma participation/la participation de mon enfant est volontaire, que je peux retirer mon consentement/mon enfant peut retirer son consentement et quitter cette étude à tout moment sans avoir à me/se justifier, et que cela n'affectera pas mes soins médicaux/les soins médicaux de mon enfant ni mes droits légaux/les droits légaux de mon enfant.	
J'ai été informé(e) que le nouveau vaccin Janssen Ebola est en cours d'étude, et que les risques possibles liés à l'administration de ce vaccin ne sont pas tous connus.	
Je confirme que je n'ai/mon enfant n'a **PAS** contracté le virus Ebola ou été vacciné(e) contre Ebola au cours des 30 derniers jours.	
J'accepte que l'équipe de l'étude me photographie/photographie mon enfant avant la vaccination.	
Si j'ai un numéro de téléphone, j'accepte de le donner à l'équipe de l'étude et j'accepte d'être contacté par appel ou SMS pour l'administration de la seconde dose ainsi que pour le suivi (ex. questions de santé).<br>Si je n'ai pas de téléphone, j'accepte dans la mesure du possible de fournir le numéro d'un proche ou d'un ami qui pourrait être utilisé pour me joindre.	
Je comprends que les informations recueillies à mon sujet/au sujet de mon enfant seront utilisées pour étayer d'autres recherches à l'avenir et peuvent être partagées de manière anonyme avec d'autres chercheurs.	
J'accepte que mes informations médicales/les informations médicales de mon enfant qui ne contiennent pas mon/son nom, puissent être partagées avec les autorités sanitaires nationales, le fabricant du vaccin (Janssen Vaccines & Prevention B.V.) et d'autres organismes étrangers en dehors de la RDC, comme la Food & Drug Administration aux Etats-Unis. J'autorise le personnel de ces organismes à consulter et analyser mes dossiers/les dossiers de mon enfant et les publier dans des revues scientifiques.	

FORMULAIRE DE CONSENTEMENT DESTINÉ AU/À LA PARTICIPANT(E) OU AU PARENT/TUTEUR

Déclarations concernant les procédures facultatives

**Déclarations**	**Signez ou apposez votre empreinte digitale dans chaque case si oui**	**Signez ou apposez votre empreinte digitale dans chaque case si non**

(Pour les femmes participantes uniquement) J'accepte que, si je suis/ma fille est enceinte au moment de la vaccination ou devient enceinte pendant le premier mois après avoir reçu chaque vaccin, et que je suis/ma fille est sélectionnée par l'équipe de l'étude pour faire partie du groupe femme enceinte, l'équipe de l'étude puisse me contacter plus frequement que les autres femmes enceintes incluses dans l'étude par téléphone, pendant ma grossesse/la grossesse de ma fille et après mon accouchement/l'accouchement de ma fille et pourrait passer à mon domicile/au domicile de ma fille si je/ma fille ne peut pas être contactée par téléphone.		
J'accepte que si je/mon enfant est choisi par l'équipe d'étude pour faire parti au groupe de suivi de l'innocuité, l'équipe d'étude peut me contacter par téléphone après ma deuxième vaccination, et peut me voir/mon enfant à la maison si je ne peux pas être contacté par téléphone.		

POUR LES PARTICIPANTS ADULTES

Je suis âgé(e) de 18 ans ou plus et j'accepte de participer à cette étude. (Signez ou apposez votre empreinte digitale ci-dessous)

		____/____/_____<br>*jj mmm aaaa*
Nom du/de la participant(e) adulte, en majuscules	Signature/Empreinte digitale du/de la participant(e) adulte	Date

POUR LE PARENT OU GARDIEN

Je suis le parent ou gardien d'un(e) participant(e) âgé(e) entre 1 et 17 ans et j'accepte que mon enfant participe à cette étude. (Signez ou apposez votre empreinte digitale ci-dessous)

		____/____/_____<br>*jj mmm aaaa*
Nom en caractères d'imprimerie du parent ou tuteur	Signature/Empreinte digitale du parent ou gardien	Date

POUR LE PARENT OU GARDIEN

Watson-Jones D, *et al. BMJ Open* 2022; 12:e055596. doi: 10.1136/bmjopen-2021-055596

Nom en caractères d'imprimerie du/de la participant(e) (si âgé[e] entre 1 et 17 ans)

		____/____/_____ *Jj mmm aaaa*
Nom de l'investigateur en caractères d'imprimerie	Signature de l'investigateur	Date

Remplissez la rubrique suivante si le/la participant(e) ou le parent/gardien est analphabète :

Témoin de l'entretien de consentement

J'ai assisté à l'entretien de consentement pour l'étude TUJIOKOWE dans ce document. J'atteste avoir expliqué les informations de l'étude avec précision au/à la participant(e) ou à son parent/tuteur et que ce dernier/cette dernière a compris, et qu'il/elle a donné librement son consentement pour participer/la participation de son enfant, en ma présence.

		____/____/_____ *jj mmm aaaa*
Nom du témoin impartial en caractères d'imprimerie	Signature du témoin impartial	Date

Apposez l'étiquette d'identification par code à barres ci-dessous :

Watson-Jones D, *et al. BMJ Open* 2022; 12:e055596. doi: 10.1136/bmjopen-2021-055596
