# Supplementary material for: Protocol for a phase 3 trial to evaluate the effectiveness and safety of a heterologous, two-dose vaccine for Ebola virus disease in the Democratic Republic of the Congo
Source: BMJ Open. 2022 Mar 8;12(3):e055596. doi: 10.1136/bmjopen-2021-055596 (PMC8905941; doi:10.1136/bmjopen-2021-055596)
Supplement: Supplementary data [file bmjopen-2021-055596supp003.pdf]

Protocol for a phase 3 trial to evaluate the effectiveness and safety of a heterologous, two-dose vaccine for Ebola virus disease in the Democratic Republic of the Congo

Appendix 2A

Informed Assent Form in English, Version 7.1 (10 Aug 2020)

Watson-Jones D, *et al. BMJ Open* 2022; 12:e055596. doi: 10.1136/bmjopen-2021-055596

Evaluation of a heterologous, two-dose preventive Ebola vaccine for effectiveness and safety in the Democratic Republic of the Congo

"The TUJIOKOWE Study" Information Sheet and Informed Assent Form for Children aged 12 years or older

LSHTM Protocol : DRC-EB-001

Principal Investigator (PI):
Professor Jean Jacques Muyembe
Director-General
Institut National de Recherche Biomédicale
Professor of Microbiology, Kinshasa University Medical School
Kinshasa Gombe, Democratic Republic of the Congo (DRC)
Phone: 0898 949 289; Email: jjmuyembet@gmail.com

Sponsor: London School of Hygiene & Tropical Medicine, United Kingdom

Site: Democratic Republic of the Congo

INTRODUCTION

You are being invited to take part in a research study called the 'TUJIOKOWE study' to find out if a new two-dose vaccine called the 'Janssen Ebola vaccine' can protect from getting Ebola and to check whether the vaccine is safe. The TUJIOKOWE study is being implemented by the Ministry of Health of DRC through the Institut National de Recherche Biomédicale (INRB), Epicentre and the London School of Hygiene & Tropical Medicine (LSHTM).

WHAT IS EBOLA?

Ebola is a disease that makes some people become very sick, and about half of the people who catch Ebola die from it. People catch Ebola from other people who have the disease.

WHAT IS A VACCINE AND WHAT IS A CLINICAL RESEARCH STUDY?

Vaccines are medical products that are used to stop people from getting certain diseases. You may have had vaccines when you were a young child to prevent you from catching diseases like measles and polio.

Clinical research studies help health professionals to understand if medicines or vaccines work to stop or treat diseases.

ARE ANY OTHER VACCINES BEING GIVEN TO PREVENT EBOLA IN DRC?

Lots of activities are ongoing to try to stop Ebola. Some people are being given a vaccine called VSV if they have been close to or looked after someone with Ebola, but that doesn't include everyone who might get Ebola – so we want to see if this vaccine can help.

WHAT IS THIS STUDY ABOUT?

People are getting sick with Ebola in North Kivu and Ituri provinces.

This study is going to find out if another new vaccine called the Janssen Ebola vaccine can help protect people against Ebola.

WHAT DO WE KNOW ABOUT THE VACCINE?

The Janssen Ebola vaccine is designed to protect people against Ebola. **It cannot make you sick with Ebola.** Over 6000 people have received the vaccine in many different countries, including7 in Africa. So far, the vaccine is safe in people who received it. The vaccine may help you fighting Ebola, but we don't know for sure yet if the Janssen Ebola vaccine can protect people against Ebola.

WHO CAN TAKE PART IN THIS STUDY?

To be given the Janssen Ebola vaccine, your parent or guardian must agree that you can join the study. You should also decide for yourself whether you want to join the study. You must be living in one of the communities chosen to be included in the study. You should be feeling well in order to get the vaccine.

People who have had Ebola cannot be in the TUJIOKOWE study. If you have received another Ebola vaccine or routine vaccine within the last month (30 days), or if you are sick on the day of vaccination, you may be asked to come back another time.

DO I HAVE TO BE IN THIS STUDY?

You do not have to join this study if you don't want to, even if your parents or guardians want you to join. If you agree to be in the study, we will ask you to sign this assent form. You can decide that you don't want to join, and it will not affect you in any way. If you don't want to be in the study at the moment, but you change your mind later, you can join the study when the vaccination team comes back. After that second visit by the team to your community, it may not be possible to get the Janssen Ebola vaccine. You need to be in the study to get this vaccine.

WHAT WILL HAPPEN IF I AGREE TO TAKE PART IN THIS STUDY?

Putting your name or your fingerprint on this assent form means that you agree to be in the study. You can change your mind and choose to leave the TUJIOKOWE study at any time.

You will be asked some questions about the study by one of the study staff members to check that you have understood the information.

WHAT DOES THE STUDY INVOLVE?

After you have signed this assent form, you will be asked some questions to check how you are feeling. If the doctor decides that you can be vaccinated, you will get an injection of the first Janssen Ebola vaccine against Ebola into your upper arm. We will give your parents or guardians a vaccination card with a phone number of who to contact in case you feel sick at any time after your visit. The vaccination card will also show the date of when you need to come back for the second Janssen Ebola vaccine. We will ask you for a phone number and we may text or call you for the second vaccine and follow ups.

In order to identify you at the next visit, we will take photographs of your face. We do this to make sure we know who has gotten the vaccine and that you receive the second dose at the right time.

A certain number of participants will be included in a group for follow up who will be contacted by phone or a home visit.

As a result of the study pause due to COVID-19, some participants had their second vaccination with the Janssen Ebola vaccine postponed. These participants will be contacted to receive their second vaccination at a later date. Receiving the second Janssen Ebola vaccine at a later date will not have negative effects on the health of these participants.

WHAT ARE THE POSSIBLE RISKS OF BEING IN THIS STUDY?

We do not know for sure yet if the Janssen Ebola vaccine protects people from Ebola, so there is a risk that you will not be protected from Ebola even after you have taken the vaccine.

As we have mentioned, this vaccine has been given to many people in other studies.

Some of the side effects that you may have after the injections are:

- pain, swelling, itchiness, redness, or warmth around the place where you got the injection and you may have a small sore which can happen after any other vaccine.
- in the area around the injection it may be sore and a bit painful to move for a few days.
- you may notice some swelling in your armpit or in your neck.
- you may get a fever or headache, feel tired, feel a bit sick or have some other side effects.

Not everyone gets these side effects and they should only last a few days. In a few people they may last for a few weeks and very rarely a few months.

In previous research studies some people have reported that they felt some tingling in the hands and feet, or their muscles felt weak. These symptoms usually only lasted one or two days, but sometimes for several weeks. One person reported tingling, numbness and pain which lasted for several months and interfered with the person's daily activities.

Very few people under 40 years old with other medical problems have had blood clots about 3

weeks after receiving a vaccine similar to the one we are giving; one woman died and one man had problems with his leg. These problems were not thought to be related to having the vaccine.

Hundreds of infants, children and teenagers have been given the Janssen Ebola vaccine. The most common problem reported by children is pain at the place where the injection was given. Older children and teenagers sometimes say they have headache, tiredness or can feel cold. Infants sometimes have less appetite or are less energetic or may have a fever for a short time. All these problems in children are the same as those after getting other vaccines and usually get better within 2 days. No serious medical problems have been reported in children who have been given the Janssen Ebola vaccine.

The medical team will help you if you don't feel well up until 1 month after your second injection. The medical team is available at any time if you have problems or questions.

WHAT ARE THE BENEFITS OF BEING IN THIS STUDY?

On the vaccination days a doctor will check your health status and you may receive treatment or a referral for simple medical conditions like malaria. You will also learn how to protect yourself against Ebola. Your participation in this study will help in the development of vaccines to prevent Ebola and, in the future, may help people in different parts of the world.

WHAT ABOUT PREGNANCY AND/OR BREASTFEEDING DURING THE STUDY?

Some older girls may be pregnant or breast feeding but they can still be in the study. We do not know all the effects of the Janssen Ebola vaccine in pregnancy or in a baby that is being breastfed. If you know you are pregnant or breastfeeding at the time of vaccination, please tell the study staff. If you are not sure, we will offer you a pregnancy test and meeting with a doctor so you can know whether you are pregnant before each injection. If you are pregnant or become pregnant within one month of each vaccination, we will contact you during your pregnancy until 3 months after delivery to ask about your health and the health of your baby. If we cannot contact you by phone after giving birth, the study team will visit you at your home. Among the pregnant women in the study, a number will be invited to join a subset and monitored more closely by telephone or through home visits. Some babies of women in this subset will also be seen within 3 months after birth. If you become pregnant within a month of vaccination, please call the phone number on your vaccination card and notify the study team.

WHAT DO I NEED TO DO FOR MY OWN HEALTH DURING THIS STUDY?

We will ask your parents or guardians to take you to a recommended health facility if you feel sick at any time during the study. They may also call the study contact number on your vaccination card to get advice up until 30 days after you receive the second dose of vaccine. You may be referred for medical care if necessary.

We do not know for sure yet if the Janssen Ebola vaccine can protect people against Ebola. **Therefore, you must continue to follow recommendations to protect yourself from Ebola.** We will give you information on how to prevent Ebola. If you experience anything like fever, diarrhea, vomiting, or unexplained bleeding, it is very important that you or your parents or

guardians let the study team know that you are sick and that you also get medical care as soon as possible.

WHO WILL BE ABLE TO SEE MY INFORMATION?

We will keep any information that you give us private.

Staff working on the study, as well as representatives of the ethics committee, including staff overseas, may look at your medical records. All of these people are trained to understand that they must keep your name and other details private.

The results of the study will be published in scientific journals so that other physicians and researchers can deepen their knowledge. It will not be possible to identify you.

If you have any specific questions about your data, please contact the study Principal Investigator, Professor Jean Jacques Muyembe or his representative (see contact details below).

WHO IS CHECKING THAT THE STUDY IS SAFE?

This study is being implemented by partners including the Ministry of Public Health of DRC through the Institut National de Recherche Biomédicale (INRB), Epicentre and the London School of Hygiene & Tropical Medicine (LSHTM). An independent committee made up of doctors and experts who are not directly involved in the study will be looking at the study information and checking that the study is safe.

WHO CAN I TALK TO ABOUT THIS STUDY?

If you or your parents or guardians would like to talk to someone about the TUJIOKOWE study or if you think you have been harmed by being part of the study, then your parents or guardians can contact the following people:

1) The Principal Investigator responsible for this study

Professor Jean Jacques Muyembe
Director General, Institut National de Recherche Biomédicale, Kinshasa, DRC
Phone: 0898 949 289
Email: jjmuyembet@gmail.com

Local study representative
Dr Hugo Kavunga
Phone: 0823 875 153
Email: hugokavunga@gmail.com

2) The DRC ethics committees that approved this study

Professor Félicien Munday
Comité National d'Ethique et de la Santé
Kinshasa-Gombe, DRC
Phone: 0998 419 816
Email: feli1munday@yahoo.fr

Professeur Willy Bongopasi
Comité d'éthique de l'école de santé Publique
Université de Kinshasa
Téléphone: 0999 952 341
Email: bongopasi@gmail.com

If you or your parents or guardians have any questions about the TUJIOKOWE study, you may ask someone on the study team at any time.

PARTICIPANT ASSENT FORM

Title: TUJIOKOWE Study

Principal Investigator of this study: Prof. JJ Muyembe

**Statements**	**Please sign or fingerprint each box**
I understand the information in this form, and I was able to ask questions and all of my questions were answered to my satisfaction.	
I understand that joining this study is voluntary and I can choose to leave the study at any time without it affecting me.	
I have been informed that the new Janssen Ebola vaccine is still being tested, and that not all the possible risks from having this vaccine are completely known.	
I confirm that I have **NOT** previously had Ebola or been vaccinated against Ebola within the past 30 days	
I agree to have my photograph taken by the study team before vaccination.	
I agree to give the study team a contact phone number if I have one and to be contacted for the second vaccine and for follow up calls or SMS e.g. for health questions. If I don't have a phone, I agree to try and provide a relative or friend's number that can be used to contact me.	
I understand that the information collected about me will be used to support other research in the future and may be shared anonymously with other researchers.	
I agree that my medical information that will not contain my name, can be shared with the national health authorities, the vaccine manufacturer (Janssen Vaccines & Prevention B.V.) and other foreign organizations outside the DRC, such as the US Food & Drug Administration. I give permission for individuals in these organizations to access and analyse my records and publish them in scientific journals.	

PARTICIPANT ASSENT FORM

Declarations regarding optional procedures

**Statements**	**Please sign or fingerprint each box for yes**	**Please sign or fingerprint each box for no**
(For female participants only) I agree that if I am pregnant or become pregnant during the first month after vaccination and I am selected to join the pregnancy subset, I may be telephoned during my pregnancy more often than other pregnant participants in the study and after the birth of my baby to collect some information on my health and my baby's health. If this information cannot be collected by telephone, I agree that I may be visited at home by a member of the study team.		
I agree that if I am selected by the study team to be in the safety group, the study team may contact me by telephone after my second vaccination and may see me at home if I cannot be contacted by telephone.		

I agree to take part in this study. (Please sign or put your fingerprint below)

		____/____/_____ *dd mon yyyy*
Printed name of child	Signature/fingerprint of child	Date

		____/____/_____ *dd mon yyyy*
Printed name of investigator	Signature of investigator	Date

Complete if participant is illiterate:

Witness to Assent Interview

I witnessed the assent interview for the TUJIOKOWE Study in this document. I attest that I have explained the study information accurately to the participant and was understood to the best of my knowledge by the participant, and that he/she has freely given their assent to participate in my presence.

		____/____/_____ *dd mon yyyy*
Printed name of impartial witness	Signature of impartial witness	Date

Attach ID barcode label below:
