# Supplementary material for: Protocol for a phase 3 trial to evaluate the effectiveness and safety of a heterologous, two-dose vaccine for Ebola virus disease in the Democratic Republic of the Congo
Source: BMJ Open. 2022 Mar 8;12(3):e055596. doi: 10.1136/bmjopen-2021-055596 (PMC8905941; doi:10.1136/bmjopen-2021-055596)
Supplement: Supplementary data [file bmjopen-2021-055596supp004.pdf]

Protocol for a phase 3 trial to evaluate the effectiveness and safety of a heterologous, two-dose vaccine for Ebola virus disease in the Democratic Republic of the Congo

Appendix 2B

Informed Assent Form in French, Version 7.1 (10 Aug 2020)

Watson-Jones D, *et al. BMJ Open* 2022; 12:e055596. doi: 10.1136/bmjopen-2021-055596

Évaluation de l'efficacité et de l'innocuité de l'emploi d'un vaccin préventif hétérologue à deux doses contre Ebola en République démocratique du Congo

Note d'information et formulaire d'assentiment éclairé de l'« étude TUJIOKOWE » destinés aux enfants âgés de 12 ans ou plus

Protocole LSHTM : DRC-EB-001

Investigateur principal (IP) :
Professeur Jean Jacques Muyembe
Directeur général
Institut national de recherche biomédicale
Professeur de microbiologie, Faculté de médecine de l'Université de Kinshasa
Kinshasa Gombe, République démocratique du Congo (RDC)
Téléphone : 0898 949 289 ; E-mail : jjmuyembet@gmail.com

Promoteur : London School of Hygiene & Tropical Medicine, Royaume-Uni

Site : République démocratique du Congo

INTRODUCTION

Tu es invité(e) à participer à une étude de recherche appelée l'« étude TUJIOKOWE » pour savoir si un nouveau vaccin à deux doses appelé « vaccin Janssen Ebola » peut protéger contre le virus Ebola et pour vérifier si le vaccin est sans danger. L'étude TUJIOKOWE est mise en œuvre par le ministère de la santé publique de la RDC par l'intermédiaire de l'Institut national de recherche biomédicale (INRB), d'Epicentre et de la London School of Hygiene & Tropical Medicine (LSHTM).

QU'EST-CE QUE LE VIRUS EBOLA ?

Ebola est une maladie qui rend certaines personnes très malades, et environ la moitié des personnes qui contractent le virus Ebola en meurent. Le virus Ebola est transmis par des personnes atteintes de la maladie.

QU'EST-CE QU'UN VACCIN ET QU'EST-CE QU'UNE ÉTUDE DE RECHERCHE CLINIQUE ?

Les vaccins sont des produits médicaux utilisés pour empêcher les personnes d'avoir certaines maladies. Tu peux avoir eu des vaccins lorsque tu étais un jeune enfant pour te protéger de maladies comme la rougeole et la polio.

Watson-Jones D, *et al. BMJ Open* 2022; 12:e055596. doi: 10.1136/bmjopen-2021-055596

Les études de recherche clinique aident les personnels de santé à comprendre si les médicaments ou les vaccins agissent pour arrêter ou traiter les maladies.

D'AUTRES VACCINS SONT-ILS ADMINISTRÉS POUR PRÉVENIR LE VIRUS EBOLA EN RDC ?

De nombreuses activités sont en cours pour essayer d'arrêter le virus Ebola. Certaines personnes reçoivent un vaccin appelé le vaccin VSV si elles ont été en contact avec une personne infectée par Ebola ou si elles s'en sont en contact avec quelqu'un qui s'occupe des malades, mais cela n'inclut pas tous ceux qui peuvent avoir Ebola dans l'avenir - alors nous voulons voir si ce vaccin peut aider.

EN QUOI CONSISTE CETTE ÉTUDE ?

Dans les provinces du Nord-Kivu et d'Ituri, des individus sont infectés par le virus Ebola.

Cette étude va déterminer si un autre nouveau vaccin, le vaccin Janssen Ebola, peut aider à protéger contre Ebola.

QUE SAVONS-NOUS DU VACCIN ?

Le vaccin Janssen Ebola est conçu pour protéger la population contre Ebola. **Tu ne peux pas contracter le virus Ebola à cause du vaccin.** Plus de 6 000 personnes ont reçu le vaccin dans de nombreux pays, dont 7 en Afrique. Jusqu'à présent, le vaccin est sûr chez les personnes vaccinées. Le vaccin pourrait t'aider lutter contre les infections à virus Ebola, mais nous ne savons pas encore avec certitude si le vaccin Janssen Ebola peut protéger contre Ebola.

QUI PEUT PARTICIPER À CETTE ÉTUDE ?

Pour recevoir le vaccin Janssen Ebola, tes parents/gardiens doivent accepter que tu participes à l'étude. Tu dois également décider si tu souhaites participer à l'étude. Pour être inclus(e) dans l'étude, tu dois faire partie de l'une des communautés sélectionnées. Tu dois te sentir bien afin de recevoir le vaccin.

Les personnes qui ont été atteintes du virus Ebola ne peuvent pas participer à l'étude TUJIOKOWE.

Si tu as été déjà vacciné contre la maladie à virus Ebola ou un autre vaccin de routine durant les 30 derniers jours ou si tu es malade le jour de vaccination, tu seras vacciné un autre jour.

SUIS-JE OBLIGÉ(E) DE PARTICIPER À CETTE ÉTUDE ?

Tu n'es pas obligé(e) de participer à cette étude si tu ne veux pas le faire, même si tes parents ou tes gardiens veulent que tu y participes. Si tu acceptes de participer à l'étude, nous te demanderons de signer ce formulaire d'assentiment. Tu peux décider que tu ne souhaites pas participer et cela n'aura aucun effet sur toi. Si tu ne souhaites pas participer à l'étude pour le moment, mais que tu changes d'avis plus tard, tu peux participer à l'étude lorsque l'équipe de vaccination revient dans ta communauté. Après cette deuxième visite au sein de ta

communauté, il peut ne plus être possible de recevoir le vaccin Janssen Ebola. Il faut être dans l'étude pour recevoir le vaccin.

QUE SE PASSERA-T-IL SI J'ACCEPTE DE PARTICIPER À CETTE ÉTUDE ?

En mettant ton nom ou en apposant ton empreinte digitale sur ce formulaire d'assentiment, tu acceptes de participer à l'étude. Tu peux changer d'avis et décider de quitter l'étude Janssen Ebola à tout moment. L'un des membres du personnel de l'étude te posera des questions sur l'étude pour vérifier que tu as compris l'information.

QU'IMPLIQUE L'ÉTUDE ?

Après avoir signé ce formulaire d'assentiment, on te posera quelques questions pour vérifier comment tu te sens. Si le médecin décide que tu peux être vacciné(e), tu recevras une injection de la première dose de vaccin de Janssen Ebola contre Ebola dans la partie supérieure de ton bras. Nous donnerons à tes parents ou gardiens une carte de vaccination avec le numéro de téléphone de personnes à contacter au cas où tu te sens mal, à tout moment après ta visite. La carte de vaccination indiquera également la date à laquelle tu devras revenir pour la deuxième dose de vaccin Janssen Ebola. Votre numéro de téléphone vous sera demandé, il est possible que vous soyez contacté afin de vous rappeler le rendez-vous de l'administration du second vaccin et également pour assurer un suivi.

Afin de t'identifier lors de la visite suivante, nous allons prendre une photographie de ton visage. Nous le faisons afin de veiller à ce que tu reçoives la deuxième dose au bon moment et pour savoir qui a été vacciné.

Un certain nombre de participants seront inclus dans un groupe de suivi qui sera contacté par téléphone ou une visite à domicile.
Suite à la pause de l'étude due à la COVID-19, certains participants ont vu leur deuxième vaccination avec le vaccin Janssen Ebola reportée. Ces participants seront contactés pour recevoir leur deuxième vaccination à une date ultérieure. Le fait de recevoir le deuxième vaccin Janssen Ebola à une date ultérieure n'aura pas d'effets négatifs sur la santé de ces participants.

QUELS SONT LES ÉVENTUELS RISQUES LIÉS À LA PARTICIPATION À CETTE ÉTUDE ?

Nous ne savons pas encore avec certitude si le vaccin Janssen Ebola protège la population contre Ebola. Il existe donc un risque que tu ne sois pas protégé(e) contre le virus Ebola, même après la vaccination.

Comme nous l'avons mentionné, ce vaccin a été administré à de nombreuses personnes dans d'autres études.

Certains des effets secondaires que tu pourrais avoir après les injections sont les suivants :

- Douleur, gonflement, démangeaisons, rougeur ou sensation de chaleur autour de l'endroit où tu as reçu l'injection et tu pourrais être un peu endolori(e) comme après tout vaccin.

- Au site autour de l'injection, tu pourrais ressentir de la douleur lors de tes mouvements pendant quelques jours.
- Tu pourrais remarquer un gonflement sous ton bras ou dans ton cou.
- Tu pourrais avoir de la fièvre ou des maux de tête, une sensation de fatigue, te sentir un peu mal ou avoir d'autres effets secondaires.

Tout le monde ne présente pas ces effets secondaires, et ils ne durent que quelques jours. Chez certaines personnes, ils peuvent durer pendant quelques semaines et très rarement quelques mois.

Dans des études de recherche précédentes, certaines personnes ont signalé qu'elles avaient des picotements dans les mains et les pieds, ou une faiblesse musculaire. Généralement, ces symptômes ne duraient qu'un jour ou deux, mais parfois ils ont duré pendant plusieurs semaines. Une personne a signalé des picotements, un engourdissement et une douleur qui ont duré pendant plusieurs mois et interféré avec ses activités quotidiennes.

Très peu de personnes de moins de 40 ans ayant d'autres problèmes médicaux ont eu des caillots de sang environ 3 semaines après avoir reçu un vaccin similaire à celui que nous administrons ; une femme est morte et un homme a eu des problèmes à la jambe. Ces problèmes n'ont pas été considérés comme étant liés à l'administration du vaccin.

Des centaines de nourrissons, d'enfants et d'adolescents ont reçu le vaccin Janssen Ebola. Le problème le plus fréquent signalé par les enfants est la douleur à l'endroit où l'injection a été faite. Les enfants plus âgés et les adolescents disent parfois avoir mal à la tête, être fatigués ou avoir froid. Les nourrissons ont parfois moins d'appétit ou sont moins énergiques ou peuvent avoir de la fièvre pendant une courte période. Tous ces problèmes chez les enfants sont les mêmes qu'après avoir reçu d'autres vaccins et se résorbent généralement en deux jours. Aucun problème médical grave n'a été signalé chez les enfants qui ont reçu le vaccin Janssen Ebola.

L'équipe médicale vous aidera si vous ne vous sentez pas bien jusqu'à 1 mois après votre deuxième injection. Elle est disponible à tout moment si vous avez des problèmes ou des questions.

QUELS SONT LES AVANTAGES LIÉS À LA PARTICIPATION À L'ÉTUDE ?

Les jours d'administration du vaccin, un médecin vérifiera ton état de santé et tu pourrais recevoir un traitement ou être redirigé vers un spécialiste pour des affections médicales simples comme le paludisme. Tu apprendras aussi comment te protéger contre le virus Ebola. Ta participation à cette étude contribuera au développement de vaccins pour prévenir le virus Ebola et, à l'avenir, pourrait aider des personnes dans différentes régions du monde.

QU'EN EST-IL DE LA GROSSESSE ET/OU DE L'ALLAITEMENT PENDANT L'ÉTUDE ?

Certaines filles plus âgées peuvent être enceintes ou allaiter, mais elles peuvent toujours

participer à l'étude. Nous ne sommes pas sûr de tous les effets des vaccins de l'étude pendant la grossesse ou chez un bébé allaité. Si vous savez que vous êtes enceinte ou si vous allaitez au moment de la vaccination, informez-en le personnel de l'étude. Si vous n'êtes pas certaine, nous vous proposerons un test de grossesse et un entretien avec du personnel médical qui vous renseignera avant chaque vaccination. Si vous êtes enceinte ou si vous tombez enceinte dans le mois qui suit l'administration d'une des deux doses, vous serez contactee durant votre grossesse afin de vous poser des questions concernant votre sante et celle votre bebe. Dans ce cas, nous vous contacterons pendant votre grossesse et juste après votre accouchement pour vous poser des questions sur votre état de santé et celui de votre bébé. Nous vous contacterons également dans les 3 mois après votre accouchement. Si nous ne pouvons pas vous contacter par téléphone après votre accouchement, l'équipe de l'étude vous rendra visite à votre domicile. Parmi les femmes enceintes dans l'étude, un certain nombre de participante seront invitée à faire partie d'un sous-groupe pour assurer un suivi plus rapproché par téléphone ou au domicile. Certains nouveaux-nés de maman faisant partie du sous-groupe seront également suivis dans les 3 mois suivant la naissance. Si vous tombez enceinte dans le mois qui suit une vaccination, merci d'appeler le numéro de téléphone indiqué sur votre carte de vaccination et de prévenir l'équipe de l'étude.

QUE DOIS-JE FAIRE POUR MA PROPRE SANTÉ PENDANT CETTE ÉTUDE ?

Nous allons demander à tes parents ou gardiens de t'emmener à un établissement de santé recommandé si tu te sens malade à tout moment pendant l'étude. Ils peuvent également appeler le numéro de contact de l'étude indiqué sur ta carte de vaccination pour obtenir des conseils et ce jusqu'à 30 jours après avoir reçu la seconde dose du vaccin. Tu pourrais être orienté(e) vers des soins médicaux si nécessaire.

Nous ne savons pas encore avec certitude si le vaccin Janssen Ebola peut protéger la population contre Ebola. **Par conséquent, tu dois continuer à suivre les recommandations pour te protéger contre le virus Ebola.** Nous te donnerons des informations sur la façon de prévenir le virus Ebola. Si tu présentes des symptômes comme de la fièvre, de la diarrhée, des vomissements ou des saignements inexpliqués, il est très important que toi ou tes parents/gardiens informiez l'équipe de l'étude que tu es malade et que tu reçoives des soins médicaux dès que possible.

QUI POURRA CONSULTER MES INFORMATIONS ?

Nous préserverons la confidentialité des informations que tu nous donnes.

Le personnel travaillant sur l'étude, y compris les personnes en dehors du pays, peuvent consulter ton dossier médical. Toutes ces personnes sont formées à la confidentialité et comprennent qu'elles doivent garder ton nom et d'autres informations privés.
Les résultats de l'étude seront publiés dans des revues scientifiques afin que d'autres médecins et chercheurs puissent approfondir leurs connaissances. Il ne sera pas possible de t'identifier.

Si tu as des questions spécifiques sur tes données, contacte l'investigateur principal de l'étude, le Professeur Jean Jacques Muyembe ou son représentant (voir les coordonnées ci-dessous).

QUI VÉRIFIE QUE L'ÉTUDE EST SANS DANGER ?

Cette étude est mise en œuvre par des partenaires, comme le Ministère de la Santé de la RDC par l'intermédiaire de l'Institut national de recherche biomédicale (INRB), d'Epicentre et de la London School of Hygiene & Tropical Medicine (LSHTM). Un comité indépendant composé de médecins et d'experts qui ne sont pas directement impliqués dans l'étude examinera les informations de l'étude et vérifiera la sûreté de l'étude.

À QUI PUIS-JE PARLER DE CETTE ÉTUDE ?

Si tes parents/gardiens ou toi souhaitez parler à quelqu'un de l'étude TUJIOKOWE ou si tu penses avoir subi un préjudice en lien avec ta participation à l'étude, tes parents ou gardiens peuvent contacter les personnes suivantes :

1) L'investigateur principal responsable de cette étude

Professeur Jean Jacques Muyembe<br>*Directeur général,* Institut national de recherche biomédicale,<br>Kinshasa, RDC<br>Téléphone : 0898 949 289<br>E-mail : jjmuyembet@gmail.com<br><br>Représentant locale de l'étude<br>Dr Hugo Kavunga<br>Téléphone : 0823 875 153<br>E-mail : hugokavunga@gmail.com

2) Les comités d'éthique de la RDC qui ont approuvé cette étude

Professeur Félicien Munday<br>Comité National d'Ethique et de la Santé<br>Kinshasa-Gombe, RDC<br>Téléphone : 0998 419 816<br>E-mail : feli1munday@yahoo.fr
Professeur Willy Bongopasi<br>Comité d'éthique de l'école de santé publique<br>Université de Kinshasa, RDC<br>Téléphone : 0999 952 341<br>E-mail : bongopasi@gmail.com

Si tes parents/gardiens ou toi avez des questions concernant l'étude TUJIOKOWE, vous pouvez les poser à un membre de l'équipe de l'étude à tout moment.

FORMULAIRE D'ASSENTIMENT DU/DE LA PARTICIPANT(E)

Titre : Étude TUJIOKOWE

Investigateur principal de cette étude : Prof. JJ Muyembe

**Déclarations**	**Signe ou appose ton empreinte digitale dans chaque case**
Je comprends les informations contenues dans ce formulaire et j'ai pu poser des questions et toutes mes questions ont reçu une réponse satisfaisante.	
Je comprends que ma participation à cette étude est volontaire et que je peux choisir de quitter l'étude à tout moment sans que cela ne m'affecte.	
J'ai été informé(e) que le nouveau vaccin Janssen Ebola est en cours d'étude, et que les risques possibles liés à l'administration de ce vaccin ne sont pas tous connus.	
Je confirme que je n'ai **PAS** contracté le virus Ebola ou été vacciné(e) contre Ebola au cours des 30 derniers jours	
J'accepte que l'équipe de l'étude me photographie avant la vaccination.	
Si j'ai un numéro de téléphone, j'accepte de le donner à l'équipe de l'étude et j'accepte d'être contacté par appel ou SMS pour l'administration de la seconde dose ainsi que pour le suivi (ex. questions de santé). Si je n'ai pas de téléphone, j'accepte dans la mesure du possible de fournir le numéro d'un proche ou d'un ami qui pourrait être utilisé pour me joindre.	
Je comprends que les informations recueillies à mon sujet seront utilisées pour étayer d'autres recherches à l'avenir et peuvent être partagées de manière anonyme avec d'autres chercheurs.	
J'accepte que mes informations médicales qui ne contiennent pas mon nom, puissent être partagées avec les autorités sanitaires nationales, le fabricant du vaccin (Janssen Vaccines & Prevention B.V.) et d'autres organismes étrangers en dehors de la RDC, comme la Food & Drug Administration des Etats Unis. J'autorise le personnel de ces organismes à consulter et analyser mes dossiers et les publier dans des revues scientifiques.	

FORMULAIRE D'ASSENTIMENT DU/DE LA PARTICIPANT(E)

Déclarations concernant les procédures facultatives

**Déclarations**	**Signe ou appose ton empreinte digitale dans chaque case si oui**	**Signe ou appose ton empreinte digitale dans chaque case si non**
(Pour les femmes participantes uniquement) J'accepte que si je suis enceinte au moment de la vaccination ou devient enceinte pendant le premier mois après avoir reçu chaque vaccin, et que je suis sélectionnée pour faire partie du groupe Grossesse, et qu'un membre de l'équipe de l'étude puisse me contacter plus frequement que les autres femmes enceintes incluses dans l'étude par téléphone pendant ma grossesse et, j'accepte également le suivi après mon accouchement pour recueillir des informations sur ma santé et celle de mon bébé. Si ces informations ne peuvent pas être recueillies par téléphone, j'accepte qu'un membre de l'équipe de l'étude vienne me voir à mon domicile.		
J'accepte que si je suis choisi par l'équipe d'étude pour faire appel au groupe de sécurité, l'équipe d'étude peut me contacter par téléphone après ma deuxième vaccination, et peut me voir à la maison si je ne peux pas être contacté par téléphone.		

J'accepte de participer à cette étude. (Signe ou appose ton empreinte digitale ci-dessous)

		____/____/_____ *jj mmm aaaa*
Nom de l'enfant en caractères d'imprimerie	Signature/Empreinte digitale de l'enfant	Date

		____/____/_____ *jj mmm aaaa*
Nom de l'investigateur en caractères d'imprimerie	Signature de l'investigateur	Date

Remplir si le/la participant(e) est analphabète :

Témoin de l'entretien d'assentiment

J'ai assisté à l'entretien d'assentiment pour l'étude [insérer nom] dans ce document. J'atteste avoir expliqué les informations de l'étude avec précision au/à la participant(e) et que ce dernier/cette dernière a compris, et qu'il/elle a donné librement son assentiment pour participer, en ma présence.

		____/____/_____ *jj mmm aaaa*
Nom du témoin impartial en caractères d'imprimerie	Signature du témoin impartial	Date

Apposez l'étiquette d'identification par code à barres ci-dessous :
