# Supplementary material for: Protocol for a phase 3 trial to evaluate the effectiveness and safety of a heterologous, two-dose vaccine for Ebola virus disease in the Democratic Republic of the Congo
Source: BMJ Open. 2022 Mar 8;12(3):e055596. doi: 10.1136/bmjopen-2021-055596 (PMC8905941; doi:10.1136/bmjopen-2021-055596)
Supplement: Supplementary data [file bmjopen-2021-055596supp001.pdf]

Protocol for a phase 3 trial to evaluate the effectiveness and safety of a heterologous, two-dose vaccine for Ebola virus disease in the Democratic Republic of the Congo

Appendix 1A

Informed Consent Form in English, Version 7.1 (10 Aug 2020)

Watson-Jones D, *et al. BMJ Open* 2022; 12:e055596. doi: 10.1136/bmjopen-2021-055596

Evaluation of a heterologous, two-dose preventive Ebola vaccine for effectiveness and safety in the Democratic Republic of the Congo

"The TUJIOKOWE Study" Information Sheet and Informed Consent Form

LSHTM Protocol: DRC-EB-001

Principal Investigator (PI):
Professor Jean Jacques Muyembe
Director-General
Institut National de Recherche Biomédicale
Professor of Microbiology, Kinshasa University Medical School
Kinshasa Gombe, Democratic Republic of the Congo (DRC)
Phone: 0898 949 289; Email: jjmuyembet@gmail.com

Sponsor: London School of Hygiene & Tropical Medicine, United Kingdom

Site: Democratic Republic of the Congo

INTRODUCTION

You are being invited to take part in a research study called the 'TUJIOKOWE study' to find out if a new two-dose vaccine called the 'Janssen Ebola vaccine' can protect people from getting Ebola and to check whether the vaccine is safe. The TUJIOKOWE study is being implemented by the Ministry of Health of DRC through the Institut National de Recherche Biomédicale (INRB), Epicentre and the London School of Hygiene & Tropical Medicine (LSHTM).

WHAT IS EBOLA?

Ebola is a disease named after the Ebola virus. When a person gets Ebola, they can become very sick, and about half of the people who catch Ebola die. Once a person has Ebola, the disease can be spread to other people, especially if people are living in the same house.

WHAT IS A VACCINE AND WHAT IS A CLINICAL RESEARCH STUDY?

Vaccines are medical products that are used to stop people from having certain diseases. Many people get vaccines to prevent them catching diseases like measles, polio, and cholera, for example.

A clinical research study helps health professionals understand new ways to prevent or treat a disease. In this study, the vaccine is still under testing. Participants in research studies about vaccines are followed over time so that the researchers can learn more about the vaccine.

ARE ANY OTHER VACCINES BEING GIVEN TO PREVENT EBOLA IN DRC?

Vaccination is occurring with a vaccine called "VSV" in several places like Beni, Katwa and Butembo. This vaccine is only being given to people who are at risk of catching Ebola, such as people who have come into contact with someone who has Ebola, or who have come into contact with people close to someone with Ebola. It is also being given to community health workers and other workers who may met Ebola. However, about half of the people who become infected with Ebola in DRC are not known to have had contact with an Ebola case, so we need to protect them too. Therefore, we are looking for additional ways to protect people from Ebola, including this vaccine.

WHAT IS THIS STUDY ABOUT?

This study is going to find out if a new vaccine can help protect people against Ebola. This new vaccine is given in two injections. The first injection is a dose of a vaccine called Ad26.ZEBOV. A second injection is given about two months later, with a second dose called MVA-BN-Filo. In this study, we will call these two injections with two different vaccines the 'Janssen Ebola vaccine'.

In the rest of this document, we will talk about you. The word 'you' can mean you or your child. If you are the parent or guardian of a child under 18 years of age and you would like your child to join this study, you will need to sign separate documents for you and your child.

If your child is aged 12 to 17 years old, he/she will also need to agree to join the study himself/herself and sign a different document, called the assent form.

WHAT DO WE KNOW ABOUT THE VACCINE?

The Janssen Ebola vaccine is designed to protect people against Ebola. **It cannot make you sick with Ebola.** Over 6000 people have received the vaccine in many different countries, including 7 in Africa. So far, the vaccine is safe and well-tolerated in people who received it. The vaccine may help the body to fight Ebola infections, but we don't know for sure yet if the Janssen Ebola vaccine can protect people against Ebola.

WHO CAN TAKE PART IN THIS STUDY?

To be in this study, you must be living in or working in one of the communities that will be offered the Janssen Ebola vaccine. You must be able to return to get the second dose of vaccine and be willing to follow the requirements needed to take part in this study. Participants should be well in order to get each dose of the vaccine. People diagnosed with HIV (who are well), pregnant and breastfeeding women, and children aged 1 year or older can be included in this study.

You cannot be in this study if you are an Ebola survivor. You also cannot be in this study if you are allergic to eggs or egg products.

If you have received another Ebola vaccine or routine vaccine within the last month (30 days) or if you have a fever of 38°C or higher on the vaccination day, you may be asked to come back another day. You may receive the vaccine if you have a mild cold, cough or mild diarrhea.

DO I HAVE TO BE IN THIS STUDY?

You do not have to join this study if you don't want to. If you agree to be in the study, we will ask you to sign this consent form. If you decide that you don't want to be in the study, then this will not affect you in any way. If you don't want to be in the study, but you change your mind later, you will have another chance to join the study. After the second visit by the team to your community, it may not be possible to join this study to get the Janssen Ebola vaccine. You must be in the study to receive the vaccine.

WHAT WILL HAPPEN IF I AGREE TO TAKE PART IN THIS STUDY?

We will describe the study and answer any questions that you may have. If you would like a copy of the written information, we will give it to you. You will be asked some questions about the study by one of the study staff members to check that you have understood the information. You will be asked to sign or put your fingerprint on this informed consent form. Putting your name or your fingerprint on the consent form means that you agree to be in the study, but you can change your mind and choose to leave at any time.

WHAT DOES THE STUDY INVOLVE?

After you sign this consent form, you will be asked some questions to check how you are feeling. If the doctor decides that you can be vaccinated, you will get an injection of the first Janssen Ebola vaccine into your upper arm, or the thigh for younger children. We will give you a vaccination card with a phone number of who to contact in case you need it. The card will also show the date of when you need to come back for the second Janssen Ebola vaccine. We will ask you for a phone number and we may call you for the second vaccine and follow ups.

In order to identify you at the next visit, we will take photographs of your face. We do this to make sure that we know who has been vaccinated and ensure you receive the second dose at the right time.

You will be asked to attend on a specific date for the second Janssen Ebola vaccine. After the second dose, we will ask you to call us if you have any medical problems in the 30 days after your second vaccine dose. You should report any sickness to the study team in those 30 days by calling the phone number on your vaccination card. We will ensure that there are telephones available in your community.

A certain number of participants will be included in a group for follow up who will be contacted by phone or a home visit.

As a result of the study pause due to COVID-19, some participants had their second vaccination with the Janssen Ebola vaccine postponed. These participants will be called to receive their

second vaccination at a later date. Receiving the second Janssen Ebola vaccine at a later date will not have negative effects on the health of these participants.

WHAT ARE THE POSSIBLE RISKS OF BEING IN THIS STUDY?

We do not know for sure yet if the Janssen Ebola vaccine protects people from Ebola, so there is a risk that you will not be protected from Ebola even after you have taken the vaccine.

This vaccine has been given to more than 6000 people in other research studies. Some of the side effects that you may have after the injections are:

- pain, swelling, itchiness, redness, or warmth around the place where you got the injection.
- in the area around the injection it may be sore and a bit painful to move for a few days.
- you may notice that you have swollen bumps under your arms or in your neck for a few days.
- you may get a fever or headache, feel tired, or have chills, rash, nausea, change in appetite, vomiting, diarrhea, dizziness, muscle, joint or belly aches, mouth sores, or body pains.

In the previous research studies, not everyone has gotten these side effects after the injections. Side effects are usually mild and most only last a few days. In a few people they may last for a few weeks. Some people have reported that they felt some tingling in the hands and feet, or their muscles felt weak. These symptoms usually only lasted one or two days, but sometimes for several weeks. One person reported tingling, numbness and pain which lasted for several months and interfered with the person's daily activities.

We have learnt that 3 women living in other countries who were less than 40 years old had serious blood clots within 3 weeks of receiving a vaccine that was made in a similar way to the second Janssen Ebola vaccine. One of them had a stroke and died. A 50-year-old man also reported lack of blood flow to the leg. These cases also had medical conditions that are known to increase the risk of blood clots and none of these events was considered related to vaccine. Receiving the vaccine did not increase a person's chance of experiencing these problems.

Hundreds of babies (aged 1-3 years), children (aged 4-11 years) and adolescents (aged 12-17 years) have received the Janssen Ebola vaccine so far. The reported side effects in children and adolescents after vaccination were similar to those reported by adults. Injection site pain was the most frequently reported local side effect. Headache, fatigue and chills were also frequently reported in older children and adolescents. Decreased appetite, decreased activity and fever were the most frequently reported side effects in babies. Following vaccination, a few children experienced a fever above 39°C. Most of these side effects were mild to moderate and did not last more than 1 or 2 days. No serious medical problems have been seen in children who received the vaccine. The side effects in children are similar to those that happen with other vaccines in children.

The medical team will provide care if you experience side-effects from the injections up until a month after the second dose. They are available if you have any problems or questions.

WHAT ARE THE BENEFITS OF BEING IN THIS STUDY?

On the days of vaccination, a doctor will question you to check if you have any symptoms and, if necessary, you may receive free treatment or a referral for simple medical conditions like malaria. You will also learn how to protect yourself against Ebola. Your participation in this study will help in the development of vaccines to prevent Ebola and, in the future, may help people in different parts of the world.

WHAT ABOUT PREGNANCY AND/OR BREASTFEEDING DURING THE STUDY?

You can participate in this study if you are pregnant or breastfeeding. We do not know all the effects of the study vaccines in pregnancy or in a baby that is being breastfed. If you know you are pregnant or breastfeeding at the time of vaccination, please tell the study staff. If you are not sure, we will offer you a pregnancy test and medical interview and will let you know if you are pregnant before each vaccination. If you are pregnant or become pregnant within one month of each vaccination, we will contact you during your pregnancy to ask you questions about your health and your baby's health. We will also contact you within 3 months after the delivery of your baby. If we cannot contact you by telephone after your baby is born, the study team may visit you at your home. Among the pregnant women in the study, a certain number will be invited to join a pregnancy subset and will be followed up by telephone or through home visits more frequently and some babies of women in the pregnancy subset will also be seen within 3 months after birth. If you become pregnant within one month after vaccination, please contact the phone number on your vaccination card and let the study team know.

WHAT DO I NEED TO DO FOR MY OWN HEALTH DURING THIS STUDY?

If you become sick during the study, you should go to one of the recommended health facilities that are on the list that we will give you. You should also call the study contact phone number on your vaccination card and tell us if you are having any medical problems up until 30 days after you received the second dose of the vaccine. You may be referred for medical care if necessary.

We do not know for sure yet if the Janssen Ebola vaccine can protect people against Ebola. If you get the vaccine, you may be protected from Ebola, but it is possible that you may not be protected. **Therefore, you must continue to protect yourself from contact with Ebola.** We will give you specific information on how to prevent Ebola, and you should always contact a member of the study team if you have any questions about how to protect yourself from contact with Ebola. If you experience anything that seems like the symptoms of Ebola, such as fever, diarrhea, vomiting, or unexplained bleeding, it is very important that you let the study team know that you are sick and that you seek medical care right away.

If we find out anything new during this study that may be related to your health or affect your decision to continue in the study, we will talk to you about it.

WHO WILL BE ABLE TO SEE MY INFORMATION?

We will keep your study information private.

Staff working on the study, including people from overseas who are responsible for checking that this study is done properly, ethics committees, and regulatory authorities, may look at your medical records. All study staff are trained in confidentiality and will not remove any document with your name on it from the study files. All of these people understand that they must keep your identity private and they won't share your name or contact details with anyone.

A description of this study will be available on the internet at http://www.ClinicalTrials.gov. No information that can identify you will be on this website. You can look at this website at any time.

The study results will be shared, but no personal information will be included in the published study results. If you have any specific questions about your data, please contact the study Principal Investigator, Professor Jean Jacques Muyembe or his representative (see contact details below).

WHO IS CHECKING THAT THE STUDY IS SAFE?

This study is being implemented by partners including the Ministry of Health of DRC through the Institut National de Recherche Biomédicale (INRB), Epicentre, and the London School of Hygiene & Tropical Medicine (LSHTM). An independent committee made up of doctors and experts who are not directly involved in the study will be looking at the study information and checking on the safety of the vaccines.

WHO CAN I TALK TO ABOUT THIS STUDY?

If you want to talk to someone about this study or if you think you have been harmed by being part of the study, you can contact the following.

1) The Principal Investigator responsible for this study

Professor Jean Jacques Muyembe<br>*Director General,* Institut National de Recherche Biomédicale, Kinshasa, DRC<br>Phone: 0898 949 289<br>Email: jjmuyembet@gmail.com<br><br>Local study representative<br>Dr Hugo Kavunga<br>Phone: 0823 875 153<br>Email: hugokavunga@gmail.com

2) The DRC ethics committees that approved this study

Professor Félicien Munday<br>National Ethics Committee<br>Kinshasa-Gombe, DRC<br>Phone: 0998 419 816<br>Email: feli1munday@yahoo.fr
Professeur Willy Bongopasi<br>Comité d'éthique de l'école de santé publique<br>Université de Kinshasa<br>Téléphone: 0999 952 341<br>Email: bongopasi@gmail.com

If you have any questions about the TUJIOKOWE study or about your rights as a study participant, you may ask anyone on the study team at any time.

PARTICIPANT OR PARENT/GUARDIAN CONSENT FORM

Title: TUJIOKOWE Study

Principal Investigator of this study: Prof. JJ Muyembe

**Statements**	**Please sign or fingerprint each box**
I have read the information in this form about the TUJIOKOWE Study (or I have had this information explained to me by the study staff in a language that I understand). The purpose of this study and the procedures to receive this new vaccine have been fully explained to me. I was able to ask questions and have all of these questions answered to my satisfaction.	
I understand that my/my child's participation is voluntary, that I/my child can withdraw consent and leave this study at any time without giving any reason, and that this will not affect my/my child's medical care or legal rights.	
I have been informed that the new Janssen Ebola vaccine is still being tested, and that not all the possible risks from having this vaccine are completely known.	
I confirm that I/my child have **NOT** previously had Ebola or been vaccinated against Ebola within the past 30 days.	
I agree to have my/my child's photograph taken by the study team before vaccination.	
I agree to give the study team a contact phone number if I have one and to be contacted for the second vaccine and for follow up calls or SMS e.g. for health questions. If I don't have a phone, I agree to try and provide a relative or friend's number that can be used to contact me.	
I understand that the information collected about me/my child will be used to support other research in the future and may be shared anonymously with other researchers.	
I agree that my/my child's medical information that will not contain my/my child's name, can be shared with the national health authorities, the vaccine manufacturer (Janssen Vaccines & Prevention B.V.) and other foreign organizations outside the DRC, such as the US Food & Drug Administration. I give permission for individuals in these organizations to access and analyse my/my child's records and publish them in scientific journals.	

PARTICIPANT OR PARENT/GUARDIAN CONSENT FORM

Declarations Regarding Optional Procedures

**Statements**	**Please sign or fingerprint each box for yes**	**Please sign or fingerprint each box for no**
(For female participants only) I agree that, if I/my child am pregnant at the time of vaccination or become pregnant during the first month after receiving each vaccine, and I/my child am selected by the study team to participate in the pregnancy subset, the study team will contact me by telephone during my/my child's pregnancy more often than other pregnant women in the study and after the birth of my/my child's baby a member of the study team may see me/my child at home if I cannot be contacted by telephone.		
I agree that if I/ my child am selected by the study team to be in the safety group, the study team may contact me by telephone after my second vaccination and may see me/ my child at home if I cannot be contacted by telephone.		

FOR ADULT PARTICIPANT

I am aged 18 years or older and I agree to take part in this study. (Please sign or put your fingerprint below)

		____/____/_____ *dd mon yyyy*
Printed name of adult participant	Signature/fingerprint of adult participant	Date

FOR PARENT OR GUARDIAN

I am the parent or guardian of a participant aged 1 to 17 years and I agree for my child to take part in this study. (Please sign or put your fingerprint below)

		____/____/_____ *dd mon yyyy*
Printed name of the parent or guardian	Signature/fingerprint of the parent or guardian	Date

FOR PARENT OR GUARDIAN

Printed name of the participant (if aged 1-17)

		____/____/_____ *dd mon yyyy*
Printed name of investigator	Signature of investigator	Date

Complete next section if participant or parent/guardian is illiterate:

Witness to Consent Interview

I witnessed the consent interview for the TUJIOKOWE Study in this document. I attest that I have explained the study information accurately to the participant or parent/guardian and was understood to the best of my knowledge by the participant or parent/guardian, and that he/she has freely given their consent to participate/for his/her child to participate in my presence.

		____/____/_____ *dd mon yyyy*
Printed name of impartial witness	Signature of impartial witness	Date

---

Attach ID barcode label below:

Watson-Jones D, *et al. BMJ Open* 2022; 12:e055596. doi: 10.1136/bmjopen-2021-055596
