# Supplementary material for: Protocol for a phase 3 trial to evaluate the effectiveness and safety of a heterologous, two-dose vaccine for Ebola virus disease in the Democratic Republic of the Congo
Source: BMJ Open. 2022 Mar 8;12(3):e055596. doi: 10.1136/bmjopen-2021-055596 (PMC8905941; doi:10.1136/bmjopen-2021-055596)
Supplement: Supplementary data [file bmjopen-2021-055596supp010.pdf]

Protocol for a phase 3 trial to evaluate the effectiveness and safety of a heterologous, two-dose vaccine for Ebola virus disease in the Democratic Republic of the Congo

Appendix 5B

Immunogenicity Informed Consent Form for Parents in French, Version 1.1 (10 Aug 2020)

Watson-Jones D, *et al. BMJ Open* 2022; 12:e055596. doi: 10.1136/bmjopen-2021-055596

Supplemental material BMJ Publishing Group Limited (BMJ) disclaims all liability and responsibility arising from any reliance placed on this supplemental material which has been supplied by the author(s) BMJ Open

Évaluation de l'efficacité et de la sécurité d'emploi d'un vaccin préventif hétérologue à deux doses contre Ebola en République démocratique du Congo

Note d'information et formulaire de consentement éclairé pour le sous-groupe Immunogénicité de l'« étude TUJIOKOWE » destinés aux parents/tuteurs des participants pédiatriques

Protocole LSHTM : DRC-EB-001

Investigateur principal (IP) :
Professeur Jean Jacques Muyembe
Directeur général
Institut national de recherche biomédicale
Professeur de microbiologie, Faculté de médecine de l'Université de Kinshasa
Kinshasa Gombe, République démocratique du Congo (RDC)
Téléphone : 0898 949 289 ; Adresse électronique : jjmuyembet@gmail.com

Promoteur : London School of Hygiene & Tropical Medicine, Royaume-Uni

Centre : République démocratique du Congo

INTRODUCTION

L'« étude TUJIOKOWE » est une étude de recherche visant à déterminer si un nouveau vaccin à deux doses appelé « vaccin contre le virus Ebola de Janssen » peut protéger contre le virus Ebola et à vérifier si le vaccin est sans danger. L'étude TUJIOKOWE est mise en œuvre par le ministère de la Santé de la RDC par l'intermédiaire de l'Institut national de recherche biomédicale (INRB), Epicentre et la London School of Hygiene & Tropical Medicine.

Lorsque nous avons commencé l'étude TUJIOKOWE, nous avions prévu d'administrer la seconde dose du vaccin contre le virus Ebola à tous les participants 2 mois environ après la première dose. Certaines personnes vont maintenant recevoir la seconde dose du vaccin contre le virus Ebola plus tard que prévu. Cela s'explique par la nécessité de fermer temporairement les centres de l'étude en raison de l'épidémie de COVID-19. Nous voulons comprendre si les personnes qui reçoivent leur seconde dose de vaccin plus de 2 mois après présentent la même réponse immunitaire que les personnes qui l'ont reçue 2 mois après la première dose. À cet effet, nous allons devoir prélever des échantillons de sang auprès de 50 adultes, 25 adolescents et 25 enfants environ participant à l'étude TUJIOKOWE, qui ont reçu la seconde dose du vaccin contre le virus Ebola après juillet 2020. Nous demandons à votre enfant de participer à cette petite étude (que nous appelons « sous-groupe Immunogénicité ») et de fournir deux échantillons de sang, car votre enfant va recevoir la seconde dose du vaccin après juillet 2020. Si vous acceptez que votre enfant participe à l'étude, un échantillon de sang lui sera prélevé

Watson-Jones D, *et al. BMJ Open* 2022; 12:e055596. doi: 10.1136/bmjopen-2021-055596

avant l'administration de la seconde dose du vaccin contre le virus Ebola et le second échantillon de sang sera prélevé 21 jours plus tard.

Nous voulons nous assurer que l'organisme de votre enfant réagit au vaccin aussi bien que celui des enfants qui ont reçu la seconde dose du vaccin 2 mois après la première dose. À cet effet, nous allons rechercher les anticorps que produit l'organisme de votre enfant après l'administration du vaccin. Les anticorps sont produits par l'organisme après la vaccination pour vous protéger contre l'infection. Les informations obtenues en analysant le sang de votre enfant pour rechercher la présence d'anticorps seront très importantes lorsque le vaccin sera utilisé pour protéger contre le virus Ebola à l'avenir, car il ne sera peut-être pas toujours possible d'administrer la seconde dose du vaccin 2 mois exactement après la première dose.

Si vous êtes le parent ou le tuteur d'un enfant de moins de 18 ans et si vous acceptez qu'il fournisse les échantillons de sang, vous devrez signer des documents distincts pour vous et votre enfant. Si votre enfant est âgé de 12 à 17 ans, il devra également accepter de lui-même de fournir les échantillons de sang et signer un autre document, appelé « formulaire d'assentiment » pour le sous-groupe Immunogénicité.

VOTRE ENFANT EST-IL OBLIGÉ DE DONNER DES ÉCHANTILLONS DE SANG ?

Votre enfant n'est pas obligé de fournir des échantillons de sang. Si vous acceptez que votre enfant fournisse des échantillons de sang, nous vous inviterons à signer ce formulaire de consentement. Si vous ne souhaitez pas que votre enfant donne des échantillons de sang, cela ne remettra pas en cause sa participation à l'étude TUJIOKOWE.

QUE SE PASSERA-T-IL SI J'ACCEPTE QUE MON ENFANT DONNE DES ÉCHANTILLONS DE SANG ?

Nous allons décrire les modalités de prise de sang et répondre à toutes les questions que vous pourriez avoir. Si vous souhaitez une copie de cette note d'information, nous vous la remettrons. Nous vous inviterons à signer ce formulaire de consentement ou à y apposer votre empreinte digitale. En inscrivant votre nom ou en apposant votre empreinte digitale sur le formulaire de consentement, vous acceptez que votre enfant fournisse des échantillons de sang, mais vous pourrez changer d'avis à tout moment.

Une fois que votre enfant aura donné son premier échantillon de sang, il suivra les procédures habituelles de l'étude TUJIOKOWE pour recevoir la seconde dose de vaccin. Pour le prélèvement du second échantillon de sang de votre enfant, vous devrez amener votre enfant au centre de l'étude pour une visite supplémentaire 21 jours après l'administration de la seconde injection du vaccin à votre enfant. Si vous acceptez que votre enfant participe au sous-groupe Immunogénicité, chaque fois qu'il donnera son sang, nous lui donnerons des rafraîchissements.

EN QUOI CONSISTE LE PRELEVEMENT DE SANG ?

Une petite quantité de sang sera prélevée à votre enfant à l'aide d'une petite aiguille propre. Nous lui prélèverons un échantillon de sang avant qu'il reçoive la seconde injection du vaccin et 21 jours après la seconde injection. Pour le prélèvement du second échantillon de sang de votre enfant, il devra venir effectuer une visite supplémentaire au centre de l'étude 21 jours après l'administration de la seconde dose de vaccin. Pour les enfants âgés de 6 à 17 ans, nous prélèverons 5 ml environ (soit 1 cuillère à café environ) de sang à chaque fois. Pour les jeunes enfants âgés de 4 à 5 ans, nous ne prélèverons que 2,5 ml de sang à chaque fois.

Comme indiqué précédemment, les échantillons de sang que votre enfant donnera serviront à vérifier si son organisme a produit des anticorps dans son sang contre le virus Ebola après la vaccination. Vous et votre enfant ne recevrez pas les résultats de ces analyses, car ils sont destinés uniquement à la recherche scientifique.

Après avoir réalisé nos analyses sur les échantillons de sang de votre enfant pour le sous-groupe Immunogénicité de l'étude TUJIOKOWE, nous détruirons tous les échantillons de sang restants prélevés à votre enfant. Nous n'utiliserons pas les échantillons de sang de votre enfant à d'autres fins que les analyses de détection d'anticorps mentionnés dans ce formulaire de consentement. Les échantillons de sang de votre enfant ne seront pas vendus.

Les échantillons de sang de votre enfant seront étiquetés avec un code et non avec son nom. D'autres informations, comme le sexe, l'âge ou les antécédents médicaux, pourront également être partagées avec d'autres investigateurs, mais le nom de votre enfant ne sera pas communiqué.

QUELS SONT LES RISQUES EVENTUELS LIES AU DON D'UN ECHANTILLON DE SANG ?

Le prélèvement d'un échantillon de sang est généralement sans danger, mais il arrive que des personnes ressentent des douleurs ou présentent un bleu, un saignement ou (dans de très rares cas) une infection après un prélèvement de sang. Si votre enfant présente des effets secondaires après la prise de sang, l'équipe médicale lui apportera les soins nécessaires. L'équipe est disponible à tout moment si votre enfant a des problèmes ou des questions.

QUELS SONT LES BENEFICES LIES AU DON D'UN ECHANTILLON DE SANG ?

Il n'y a aucun bénéfice direct pour votre enfant à fournir des échantillons de sang dans le cadre du sous-groupe Immunogénicité de l'étude TUJIOKOWE. Le consentement de votre enfant à fournir des échantillons de sang contribuera au développement de vaccins pour prévenir le virus Ebola et, à l'avenir, pourrait aider des personnes dans différentes régions du monde.

QU'ADVIENDRA-IL DES ECHANTILLONS DE SANG PRELEVES A MON ENFANT ?

Les échantillons de sang prélevés à votre enfant dans le cadre du sous-groupe Immunogénicité de l'étude TUJIOKOWE ne seront utilisés que pour comprendre l'efficacité du vaccin contre le virus Ebola lorsque la seconde dose est administrée plus de 2 mois après la première dose.

Les échantillons seront codés de manière à limiter le nombre de membres du personnel de l'étude qui peut les relier à votre enfant. Les échantillons de sang de votre enfant seront analysés dans un laboratoire aux États-Unis. S'il reste des échantillons de sang en RDC, ils seront conservés dans le laboratoire de l'INRB à Goma. Nous détruirons tous les échantillons conservés aux États-Unis et à Goma à la fin de l'étude.

À QUI PUIS-JE M'ADRESSER CONCERNANT LE PRÉLÈVEMENT D'ÉCHANTILLONS DE SANG À ENFANT ?

Si vous souhaitez parler à quelqu'un au sujet du prélèvement d'échantillons de sang à votre enfant ou si vous pensez que votre enfant a subi un préjudice en fournissant un échantillon de sang, vous pouvez contacter les personnes suivantes :

1) Investigateur principal responsable de cette étude

Professeur Jean-Jacques Muyembe<br>*Directeur général*, Institut national de recherche biomédicale, Kinshasa, RDC<br>Téléphone : 0898 949 289<br>Adresse électronique : jjmuyembet@gmail.com<br><br>Représentant locale de l'étude<br>Dr Hugo Kavunga<br>Téléphone : 0823 875 153<br>Adresse électronique : hugokavunga@gmail.com

2) Comités d'éthique de la RDC ayant approuvé cette étude

Professeur Félicien Munday<br>Comité national d'éthique et de la santé<br>Kinshasa-Gombe, RDC<br>Téléphone : 0998 419 816<br>Adresse électronique : feli1munday@yahoo.fr
Professeur Willy Bongopasi<br>Comité d'éthique de l'école de santé publique<br>Université de Kinshasa<br>Téléphone: 0999 952 341<br>Adresse électronique : bongopasi@gmail.com

Si vous avez des questions sur le prélèvement d'échantillons de sang à votre enfant pour le sous-groupe Immunogénicité de l'étude TUJIOKOWE ou sur les droits de votre enfant, vous pouvez les poser à tout moment à n'importe quel membre de l'équipe de l'étude.

FORMULAIRE DE CONSENTEMENT DES PARTICIPANTS POUR LE SOUS-GROUPE IMMUNOGÉNICITÉ

Titre : Sous-groupe Immunogénicité de l'étude TUJIOKOWE

Investigateur principal de cette étude : Prof. JJ Muyembe

**Déclarations**	**Signez ou apposez votre empreinte digitale dans chaque case**
J'ai lu les informations contenues dans ce formulaire sur le prélèvement d'un échantillon de sang à mon enfant lors de deux visites pour le sous-groupe Immunogénicité de l'étude TUJIOKOWE (ou le personnel de l'étude m'a expliqué ces informations avec des mots que je comprends). L'objectif du prélèvement d'échantillons de sang et les procédures correspondantes m'ont été expliqués en détail. J'ai pu poser des questions et j'ai reçu des réponses satisfaisantes à toutes mes questions.	
Je comprends que la participation de mon enfant est volontaire, que mon enfant peut retirer son consentement à fournir des échantillons de sang à tout moment sans avoir à se justifier et que cela ne remettra pas en cause sa participation à l'étude TUJIOKOWE.	

Je suis le parent ou le tuteur d'un participant âgé de 4 à 17 ans et j'accepte que mon enfant fournisse des échantillons de sang pendant sa participation à l'étude TUJIOKOWE. (Signez ou apposez votre empreinte digitale ci-dessous)

		____/____/_____<br>*jj mmm aaaa*
Nom du parent ou tuteur (en caractères d'imprimerie)	Signature/Empreinte digitale du parent ou tuteur	Date

Nom de l'enfant participant (si âgé de 4 à 17 ans ; en caractères d'imprimerie)

		____/____/_____<br>*jj mmm aaaa*
Nom de l'investigateur (en caractères d'imprimerie)	Signature de l'investigateur	Date

Si le parent/tuteur est analphabète, remplissez cette section :

Témoin de l'entretien de consentement

J'ai assisté à l'entretien de consentement pour le sous-groupe Immunogénicité de l'étude TUJIOKOWE décrite dans ce document. J'atteste avoir expliqué avec exactitude les informations sur l'étude au parent/tuteur et que, à ma connaissance, cette personne les a comprises et a donné librement son consentement pour que son enfant participe, en ma présence.

		____/____/_____<br>*jj mmm aaaa*
Nom du témoin impartial (en caractères d'imprimerie)	Signature du témoin impartial	Date

Apposez l'étiquette d'identification par code à barres ci-dessous :
