# Supplementary material for: Protocol for a phase 3 trial to evaluate the effectiveness and safety of a heterologous, two-dose vaccine for Ebola virus disease in the Democratic Republic of the Congo
Source: BMJ Open. 2022 Mar 8;12(3):e055596. doi: 10.1136/bmjopen-2021-055596 (PMC8905941; doi:10.1136/bmjopen-2021-055596)
Supplement: Supplementary data [file bmjopen-2021-055596supp009.pdf]

Protocol for a phase 3 trial to evaluate the effectiveness and safety of a heterologous, two-dose vaccine for Ebola virus disease in the Democratic Republic of the Congo

Appendix 5A

Immunogenicity Informed Consent Form for Parents in English, Version 1.1 (10 Aug 2020)

Watson-Jones D, *et al. BMJ Open* 2022; 12:e055596. doi: 10.1136/bmjopen-2021-055596

Evaluation of a heterologous, two-dose preventive Ebola vaccine for effectiveness and safety in the Democratic Republic of the Congo

"The TUJIOKOWE Study" Immunogenicity Subset Information Sheet and Informed Consent Form for Parents/Guardians of Paediatric Participants

LSHTM Protocol: DRC-EB-001

Principal Investigator (PI):
Professor Jean Jacques Muyembe
Director-General
Institut National de Recherche Biomédicale
Professor of Microbiology, Kinshasa University Medical School
Kinshasa Gombe, Democratic Republic of the Congo (DRC)
Phone: 0898 949 289; Email: jjmuyembet@gmail.com

Sponsor: London School of Hygiene & Tropical Medicine, United Kingdom

Site: Democratic Republic of the Congo

INTRODUCTION

The 'TUJIOKOWE study' is a research study to find out if a new two-dose vaccine called the 'Janssen Ebola vaccine' can protect people from getting Ebola and to check whether the vaccine is safe. The TUJIOKOWE study is being implemented by the Ministry of Health of DRC through the Institut National de Recherche Biomédicale (INRB) and with Médecins Sans Frontières, Epicentre, and the London School of Hygiene & Tropical Medicine.

When we started the TUJIOKOWE study, we planned to give everybody the second dose of the Ebola vaccine about two months after the first dose. Some people will now receive the second dose of Ebola vaccine later than originally planned. This is because it was necessary to temporarily close the study clinics due to the COVID-19 outbreak. We want to understand if people who receive their second dose of vaccine later than two months have the same immune responses as the people who received their second dose of vaccine at two months after the first dose. To do this, we will need to collect blood samples from approximately 50 adults, 25 adolescents, and 25 children participating in the TUJIOKOWE study who received the second dose of the Ebola vaccine after July 2020. We are asking your child to join this small study (which we are calling the Immunogenicity Subset) and to provide two blood samples because your child will be receiving the second dose of the vaccine after July 2020. If you agree for your child to participate, one blood sample will be collected from your child before the second dose of Ebola vaccine is given and the second blood sample will be collected 21 days later.

We would like to be sure that your child's body responds to the vaccine as well as the children who received the second dose of the vaccine two months after the first dose. To check this, we will be looking at the antibodies that your child's body produces after he/she receives a vaccine. Antibodies are produced by your body after vaccination to protect you from infection. The information from testing your child's blood for antibodies will be very important when the vaccine is used to protect against Ebola in the future because it may not always be possible to give the second dose at exactly two months after the first dose of the vaccine.

If you are the parent or guardian of a child under 18 years of age and you agree to your child providing blood samples, you will need to sign separate documents for you and your child. If your child is aged 12 to 17 years old, he/she will also need to agree to provide blood samples himself/herself and sign a different document, called the immunogenicity assent form.

DOES YOUR CHILD HAVE TO GIVE BLOOD SAMPLES?

Your child does not have to provide any blood samples. If you agree to your child providing blood samples, we will ask you to sign this consent form. If you do not want your child to give any blood samples, this will not affect your child's participation in the TUJIOKOWE study.

WHAT WILL HAPPEN IF I AGREE TO MY CHILD GIVING BLOOD SAMPLES?

We will describe the blood collection process and answer any questions that you may have. If you would like a copy of the written information, we will give it to you. You will be asked to sign or put your fingerprint on this consent form. Putting your name or your fingerprint on the consent form means that you agree to your child providing blood samples, but you can change your mind at any time.

After your child has given his/her first blood sample , your child will follow the normal TUJIOKOWE study procedures to receive the second dose of vaccine. To have your child's second blood sample taken, you will need to bring your child to the study clinic for an extra visit 21 days after your child received the second injection of vaccine. If you agree to your child participating in the Immunogenicity Subset, then we will give your child some refreshments at the clinic.

WHAT DOES GIVING BLOOD INVOLVE?

A small amount of your child's blood will be collected from your child using a small clean needle. We will take blood before your child gets the second injection of vaccine and at 21 days after the second injection. To have your child's second blood sample taken your child will need to come to the study clinic for an extra visit 21 days after your child has received the second dose of vaccine. For children aged 6 to 17 years, we will collect approximately 5 mL (about 1 teaspoon) of blood each time. For young children aged 4 to 5 years, we will only collect 2.5 mL of blood each time.

As explained to you earlier, the blood samples your child gives will be used to test whether your child's body has produced antibodies in his/her blood against Ebola after vaccination. You

Watson-Jones D, *et al. BMJ Open* 2022; 12:e055596. doi: 10.1136/bmjopen-2021-055596

and your child will not receive the results of these tests because they are only for scientific research.

After we do our tests on your child's blood samples for the Immunogenicity Subset of the TUJIOKOWE study, we will destroy any remaining blood that has been collected from your child. We will not use your child's blood for any other purpose except the antibody tests that we have explained in this consent form. Your child's blood samples will not be sold.

Your child's blood samples will be labelled with a code and not his/her name. Other information, such as sex, age, or health history might also be shared with other investigators, but your child's name will not be shared.

WHAT ARE THE POSSIBLE RISKS OF GIVING A BLOOD SAMPLE?

Providing a blood sample is generally safe but occasionally people experience pain, bruising, bleeding, or (very rarely) infection from giving a blood sample. The medical team will provide care if your child experiences side-effects from the blood sampling. They are available if your child has any problems or if you have any questions.

WHAT ARE THE BENEFITS OF GIVING A BLOOD SAMPLE?

There is no direct benefit to your child for providing blood samples in the Immunogenicity Subset of the TUJIOKOWE study. Your agreement for your child to provide blood samples will help in the development of vaccines to prevent Ebola and, in the future, may help people in different parts of the world.

WHAT HAPPENS TO THE BLOOD SAMPLES COLLECTED FROM MY CHILD?

The blood samples collected from your child during the Immunogenicity Subset of the TUJIOKOWE study, will only be used to understand how the Ebola vaccine works when the second dose is given more than two months after the first dose.

The samples will be coded in a way that only limited study staff can link them to your child. Your child's blood samples will be tested in a laboratory in the United States. If any blood remains in DRC, it will be stored in the INRB laboratory in Goma. We will destroy all the samples in the United States and in Goma at the end of the study.

WHO CAN I TALK TO ABOUT MY CHILD GIVING BLOOD SAMPLES?

If you want to talk to someone about your child giving blood samples or if you think your child has been harmed by providing a blood sample, you can contact the following people:

1) The Principal Investigator responsible for this study

Professor Jean Jacques Muyembe<br>*Director General,* Institut National de Recherche Biomédicale, Kinshasa, DRC<br>Phone: 0898 949 289<br>Email: jjmuyembet@gmail.com<br><br>Local study representative<br>Dr Hugo Kavunga<br>Phone: 0823 875 153<br>Email: hugokavunga@gmail.com

2) The DRC ethics committees that approved this study

Professeur Félicien Munday<br>National Ethics Committee<br>Kinshasa-Gombe, DRC<br>Phone: 0998 419 816<br>Email: feli1munday@yahoo.fr
Professeur Willy Bongopasi<br>Comité d'éthique de l'école de santé publique<br>Université de Kinshasa<br>Phone: 0999 952 341<br>Email: bongopasi@gmail.com

If you have any questions about your child providing blood samples for the Immunogenicity Subset of the TUJIOKOWE study or about your child's rights, you may ask anyone on the study team at any time.

PARTICIPANT IMMUNOGENICITY SUBSET CONSENT FORM

Title: Immunogenicity Subset of the TUJIOKOWE Study

Principal Investigator of this study: Prof. JJ Muyembe

**Statements**	**Please sign or fingerprint each box**
I have read the information in this form about my child giving a blood sample at two visits for the Immunogenicity Subset of the TUJIOKOWE Study (or I have had this information explained to me by the study staff in a language that I understand). The purpose of providing blood samples and the procedures to give blood samples have been fully explained to me. I was able to ask questions and have all of these questions answered to my satisfaction.	
I understand that my child's participation is voluntary, that my child can withdraw consent to provide blood samples at any time without giving any reason, and that this will not affect my child's participation in the TUJIOKOWE Study.	

I am the parent or guardian of a participant aged 4 to 17 years and I agree for my child to provide blood samples during his/her participation in the TUJIOKOWE study.
(Please sign or put your fingerprint below)

		____/____/_____ *dd mon yyyy*
Printed name of the parent or guardian	Signature/fingerprint of the parent or guardian	Date

Printed name of the child participant (if aged 4-17)

		____/____/_____ *dd mon yyyy*
Printed name of investigator	Signature of investigator	Date

Complete next section if the parent/guardian is illiterate:

Witness to Consent Interview

I witnessed the consent interview for the Immunogenicity Subset of the TUJIOKOWE Study in this document. I attest that I have explained the study information accurately to the parent/guardian and was understood to the best of my knowledge by the parent/guardian, and that he/she has freely given their consent for his/her child to participate in my presence.

		____/____/_____ *dd mon yyyy*
Printed name of impartial witness	Signature of impartial witness	Date

Attach ID barcode label below:
