# Supplementary material for: Protocol for a phase 3 trial to evaluate the effectiveness and safety of a heterologous, two-dose vaccine for Ebola virus disease in the Democratic Republic of the Congo
Source: BMJ Open. 2022 Mar 8;12(3):e055596. doi: 10.1136/bmjopen-2021-055596 (PMC8905941; doi:10.1136/bmjopen-2021-055596)
Supplement: Supplementary data [file bmjopen-2021-055596supp008.pdf]

Protocol for a phase 3 trial to evaluate the effectiveness and safety of a heterologous, two-dose vaccine for Ebola virus disease in the Democratic Republic of the Congo

Appendix 4B

Immunogenicity Informed Assent Form for children in French, Version 1.1 (10 Aug 2020)

Watson-Jones D, *et al. BMJ Open* 2022; 12:e055596. doi: 10.1136/bmjopen-2021-055596

Évaluation de l'efficacité et de la sécurité d'emploi d'un vaccin préventif hétérologue à deux doses contre Ebola en République démocratique du Congo

Note d'information et formulaire d'assentiment éclairé pour le sous-groupe Immunogénicité de l'« étude TUJIOKOWE » destinés aux enfants âgés de 12 ans ou plus

Protocole LSHTM : DRC-EB-001

Investigateur principal (IP) :
Professeur Jean Jacques Muyembe
Directeur général
Institut national de recherche biomédicale
Professeur de microbiologie, Faculté de médecine de l'Université de Kinshasa
Kinshasa Gombe, République démocratique du Congo (RDC)
Téléphone : 0898 949 289 ; Adresse électronique : jjmuyembet@gmail.com

Promoteur : London School of Hygiene & Tropical Medicine, Royaume-Uni

Centre : République démocratique du Congo

INTRODUCTION

L'« étude TUJIOKOWE » est une étude de recherche visant à déterminer si un nouveau vaccin à deux doses appelé « vaccin contre le virus Ebola de Janssen » peut protéger contre le virus Ebola et à vérifier si le vaccin est sans danger. L'étude TUJIOKOWE est mise en œuvre par le ministère de la Santé de la RDC par l'intermédiaire de l'Institut national de recherche biomédicale (INRB), Epicentre et la London School of Hygiene & Tropical Medicine.

Lorsque nous avons commencé l'étude TUJIOKOWE, nous avions prévu d'administrer la seconde injection du vaccin contre le virus Ebola à tous les participants 2 mois environ après la première injection. Certaines personnes vont maintenant recevoir la seconde injection du vaccin contre le virus Ebola plus tard que prévu. Cela s'explique par la nécessité de fermer temporairement les centres de l'étude en raison de l'épidémie de COVID-19. Nous voulons savoir si le vaccin contre le virus Ebola est aussi efficace lorsque les personnes reçoivent leur seconde injection plus de 2 mois après. À cet effet, nous allons devoir prélever des échantillons de sang auprès de 50 adultes, 25 adolescents et 25 enfants environ participant à l'étude TUJIOKOWE, qui ont reçu la seconde injection du vaccin contre le virus Ebola après juillet 2020. Nous vous demandons de participer à cette petite étude (que nous appelons « sous-groupe Immunogénicité ») et de fournir deux échantillons de sang, car vous allez recevoir la seconde injection du vaccin après juillet 2020. Si vous acceptez de participer à l'étude, un

échantillon de sang vous sera prélevé avant l'administration de la seconde injection du vaccin contre le virus Ebola, et le second échantillon de sang sera prélevé 21 jours plus tard.

Nous voulons nous assurer que votre organisme réagit au vaccin aussi bien que celui des personnes qui ont reçu la seconde injection du vaccin 2 mois après la première injection. À cet effet, nous allons rechercher les anticorps que produit votre organisme après l'administration du vaccin. Les anticorps sont produits par l'organisme après la vaccination pour vous protéger contre l'infection. Les informations obtenues en analysant votre sang pour rechercher la présence d'anticorps seront très importantes lorsque le vaccin sera utilisé pour protéger contre le virus Ebola à l'avenir, car il ne sera peut-être pas toujours possible d'administrer la seconde injection du vaccin 2 mois exactement après la première injection du vaccin.

SUIS-JE OBLIGÉ DE DONNER DES ÉCHANTILLONS DE SANG ?

Vous n'êtes pas obligé de fournir des échantillons de sang si vous ne le souhaitez pas, même si vos parents ou tuteurs le souhaitent. Si vous acceptez de fournir des échantillons de sang, nous vous inviterons à signer ce formulaire d'assentiment. Si vous ne souhaitez pas donner d'échantillons de sang, cela ne remettra pas en cause votre participation à l'étude TUJIOKOWE.

QUE SE PASSERA-T-IL SI J'ACCEPTE DE DONNER DES ÉCHANTILLONS DE SANG ?

Nous allons décrire les modalités de prise de sang et répondre à toutes les questions que vous pourriez avoir. Si vous souhaitez une copie de cette note d'information, nous vous la remettrons. Nous vous inviterons à signer ce formulaire d'assentiment ou à y apposer votre empreinte digitale. En inscrivant votre nom sur le formulaire d'assentiment ou en y apposant votre empreinte digitale, vous acceptez de fournir des échantillons de sang, mais vous pourrez changer d'avis à tout moment.

Une fois que vous aurez donné votre premier échantillon de sang, vous suivrez les procédures habituelles de l'étude TUJIOKOWE pour recevoir la seconde injection du vaccin. Pour le prélèvement de votre second échantillon de sang, vous devrez venir effectuer une visite supplémentaire au centre de l'étude 21 jours après l'administration de la seconde injection du vaccin. Si vous participez au sous-groupe Immunogénicité, chaque fois que vous donnerez votre sang, nous vous donnerons des rafraîchissements.

EN QUOI CONSISTE LE PRELEVEMENT DE SANG ?

Une petite quantité de votre sang vous sera prélevée à l'aide d'une petite aiguille propre. Nous vous prélèverons un échantillon de sang avant que vous receviez la seconde injection du vaccin et 21 jours après la seconde injection. Pour le prélèvement de votre second échantillon de sang, vous devrez venir effectuer une visite supplémentaire au centre de l'étude 21 jours après l'administration de la seconde injection du vaccin. Pour les enfants âgés de 6 ans et plus, nous prélèverons 5 ml environ (soit 1 cuillère à café environ) de sang chaque fois que vous fournirez un échantillon de sang.

Comme indiqué précédemment, les échantillons de sang que vous donnerez serviront à vérifier si votre organisme a produit des anticorps dans votre sang contre le virus Ebola après la vaccination. Vous ne recevrez pas les résultats de ces analyses, car ils sont destinés uniquement à la recherche scientifique.

Après avoir réalisé nos analyses sur vos échantillons de sang pour le sous-groupe Immunogénicité de l'étude TUJIOKOWE, nous détruirons tous les échantillons de sang restants qui vous ont été prélevés. Nous n'utiliserons pas vos échantillons de sang à d'autres fins que les analyses de détection d'anticorps mentionnés dans ce formulaire d'assentiment. Vos échantillons de sang ne seront pas vendus.

Vos échantillons de sang seront étiquetés avec un code et non avec votre nom. D'autres informations, comme le sexe, l'âge ou les antécédents médicaux, pourront également être partagées avec d'autres investigateurs, mais votre nom ne sera pas communiqué.

QUELS SONT LES RISQUES EVENTUELS LIES AU DON D'UN ECHANTILLON DE SANG ?

Le prélèvement d'un échantillon de sang est généralement sans danger, mais il arrive que des personnes ressentent des douleurs ou présentent un bleu, un saignement ou (dans de très rares cas) une infection après un don de sang. Si vous présentez des effets secondaires après la prise de sang, l'équipe médicale vous apportera les soins nécessaires. L'équipe est disponible à tout moment si vous avez des problèmes ou des questions.

QUELS SONT LES BENEFICES LIES AU DON D'UN ECHANTILLON DE SANG ?

Il n'y a aucun bénéfice direct pour vous à fournir des échantillons de sang dans le cadre du sous-groupe Immunogénicité de l'étude TUJIOKOWE. Le consentement à fournir des échantillons de sang contribuera au développement de vaccins pour prévenir le virus Ebola et, à l'avenir, pourrait aider des personnes dans différentes régions du monde.

QU'ADVIENDRA-IL DES ECHANTILLONS DE SANG QUI ME SERONT PRELEVES ?

Les échantillons de sang qui vous ont été prélevés dans le cadre du sous-groupe Immunogénicité de l'étude TUJIOKOWE ne seront utilisés que pour comprendre l'efficacité du vaccin contre le virus Ebola lorsque la seconde injection est administrée plus de 2 mois après la première injection.

Les échantillons seront codés de manière à limiter le nombre de membres du personnel de l'étude qui peut les relier à vous. Vos échantillons de sang seront analysés dans un laboratoire aux États-Unis. S'il reste des échantillons de sang en RDC, ils seront conservés dans le laboratoire de l'INRB à Goma. Nous détruirons tous les échantillons conservés aux États-Unis et à Goma à la fin de l'étude.

À QUI PUIS-JE M'ADRESSER CONCERNANT LE PRÉLÈVEMENT D'ÉCHANTILLONS DE SANG ?

Si vous souhaitez parler à quelqu'un du prélèvement d'un échantillon de sang ou si vous pensez avoir subi un préjudice en fournissant un échantillon de sang, vous pouvez contacter les personnes suivantes :

1) Investigateur principal responsable de cette étude

Professeur Jean-Jacques Muyembe<br>*Directeur général*, Institut national de recherche biomédicale, Kinshasa, RDC<br>Téléphone : 0898 949 289<br>Adresse électronique : jjmuyembet@gmail.com<br><br>Représentant locale de l'étude<br>Dr Hugo Kavunga<br>Téléphone : 0823 875 153<br>Adresse électronique : hugokavunga@gmail.com

2) Comités d'éthique de la RDC qui ont approuvé cette étude

Professeur Félicien Munday<br>Comité national d'éthique et de la santé<br>Kinshasa-Gombe, RDC<br>Téléphone : 0998 419 816<br>Adresse électronique : feli1munday@yahoo.fr
Professeur Willy Bongopasi<br>Comité d'éthique de l'école de santé publique<br>Université de Kinshasa, RDC<br>Téléphone : 0999 952 341<br>Adresse électronique : bongopasi@gmail.com

Si vous avez des questions sur le prélèvement d'échantillons de sang pour le sous-groupe Immunogénicité de l'étude TUJIOKOWE ou sur vos droits, vous pouvez les poser à tout moment à n'importe quel membre de l'équipe de l'étude.

FORMULAIRE DE ASSENTIMENT DES PARTICIPANTS POUR LE SOUS-GROUPE IMMUNOGÉNICITÉ

Titre : Sous-groupe Immunogénicité de l'étude TUJIOKOWE

Investigateur principal de cette étude : Prof. JJ Muyembe

**Déclarations**	**Signez ou apposez votre empreinte digitale dans chaque case**
J'ai lu les informations contenues dans ce formulaire sur le prélèvement d'un échantillon de sang lors de deux visites pour le sous-groupe Immunogénicité de l'étude TUJIOKOWE (ou le personnel de l'étude m'a expliqué ces informations avec des mots que je comprends). L'objectif du prélèvement d'échantillons de sang et les procédures correspondantes m'ont été expliqués en détail. J'ai pu poser des questions et j'ai reçu des réponses satisfaisantes à toutes mes questions.	
Je comprends que ma participation est volontaire, que je peux retirer son consentement à fournir des échantillons de sang à tout moment sans avoir à me justifier et que cela ne remettra pas en cause ma participation à l'étude TUJIOKOWE.	

Je suis âgé de 12 à 17 ans et j'accepte de fournir des échantillons de sang pour le sous-groupe Immunogénicité pendant ma participation à l'étude TUJIOKOWE. (Signez ou apposez votre empreinte digitale ci-dessous)

		____/____/_____<br>*jj mmm aaaa*
Nom de l'enfant (en caractères d'imprimerie)	Signature/Empreinte digitale de l'enfant	Date

		____/____/_____<br>*jj mmm aaaa*
Nom de l'investigateur (en caractères d'imprimerie)	Signature de l'investigateur	Date

Si le participant est analphabète, remplissez cette section :

Témoin de l'entretien d'Assentiment

J'ai assisté à l'entretien d'assentiment pour le sous-groupe Immunogénicité de l'étude TUJIOKOWE décrite dans ce document. J'atteste avoir expliqué avec exactitude les informations sur l'étude au participant et que, à ma connaissance, le participant les a comprises et a donné librement son assentiment à participer, en ma présence.

		____/____/_____<br>*jj mmm aaaa*
Nom du témoin impartial (en caractères d'imprimerie)	Signature du témoin impartial	Date

Apposez l'étiquette d'identification par code à barres ci-dessous :
