# Supplementary material for: Protocol for a phase 3 trial to evaluate the effectiveness and safety of a heterologous, two-dose vaccine for Ebola virus disease in the Democratic Republic of the Congo
Source: BMJ Open. 2022 Mar 8;12(3):e055596. doi: 10.1136/bmjopen-2021-055596 (PMC8905941; doi:10.1136/bmjopen-2021-055596)
Supplement: Supplementary data [file bmjopen-2021-055596supp007.pdf]

Protocol for a phase 3 trial to evaluate the effectiveness and safety of a heterologous, two-dose vaccine for Ebola virus disease in the Democratic Republic of the Congo

Appendix 4A

Immunogenicity Informed Assent Form for children in English, Version 1.1 (10 Aug 2020)

Watson-Jones D, *et al. BMJ Open* 2022; 12:e055596. doi: 10.1136/bmjopen-2021-055596

Evaluation of a heterologous, two-dose preventive Ebola vaccine for effectiveness and safety in the Democratic Republic of the Congo

"The TUJIOKOWE Study" Immunogenicity Subset
Information Sheet and Informed Assent Form for Children Aged 12 Years or Older

LSHTM Protocol: DRC-EB-001

Principal Investigator (PI):
Professor Jean Jacques Muyembe
Director-General
Institut National de Recherche Biomédicale
Professor of Microbiology, Kinshasa University Medical School
Kinshasa Gombe, Democratic Republic of the Congo (DRC)
Phone: 0898 949 289; Email: jjmuyembet@gmail.com

Sponsor: London School of Hygiene & Tropical Medicine, United Kingdom

Site: Democratic Republic of the Congo

INTRODUCTION

The 'TUJIOKOWE study' is a research study to find out if a new two-dose vaccine called the 'Janssen Ebola vaccine' can protect people from getting Ebola and to check whether the vaccine is safe. The TUJIOKOWE study is being implemented by the Ministry of Health of DRC through the Institut National de Recherche Biomédicale (INRB), Epicentre, and the London School of Hygiene & Tropical Medicine.

When we started the TUJIOKOWE study, we planned to give everybody the second injection of the Ebola vaccine about two months after the first injection. Some people will now receive the second injection of Ebola vaccine later than originally planned. This is because we needed to temporarily close the study clinics due to the COVID-19 outbreak. We want to understand if the Ebola vaccine works well if people receive their second injection later than two months. To do this, we will need to collect blood samples from about 50 adults, 25 adolescents, and 25 children participating in the TUJIOKOWE study who received the second injection of the Ebola vaccine after July 2020. We are asking you to join this small study (which we are calling the Immunogenicity Subset) and to provide two blood samples because you will be receiving the second injection of the vaccine after July 2020. If you agree to participate, one blood sample will be collected from you before the second injection of Ebola vaccine is given and the second blood sample will be collected 21 days later.

We would like to be sure that your body responds to the vaccine as well as people who received the second injection of the vaccine two months after the first injection. To check this, we will

be looking at the antibodies that your body produces after you receive a vaccine. Antibodies are produced by your body after vaccination to protect you from infection. The information from testing your blood for antibodies will be very important when the vaccine is used to protect against Ebola in the future because it may not always be possible to give the second injection at exactly two months after the first injection of the vaccine.

DO I HAVE TO GIVE BLOOD SAMPLES?

You do not have to provide any blood samples if you do not want to, even if your parents or guardians want you to. If you agree to provide blood samples, we will ask you to sign this assent form. If you do not want to give any blood samples, this will not affect your participation in the TUJIOKOWE study.

WHAT WILL HAPPEN IF I AGREE TO GIVE BLOOD SAMPLES?

We will describe the blood collection process and answer any questions that you may have. If you would like a copy of the written information, we will give it to you. You will be asked to sign or put your fingerprint on this assent form. Putting your name or your fingerprint on the assent form means that you agree to provide blood samples, but you can change your mind at any time.

After the first blood sample you give, you will follow the normal TUJIOKOWE study procedures to receive the second injection of vaccine. To have your second blood sample taken you will need to come to the study clinic for an extra visit 21 days after you have received the second injection of vaccine. If you participate in the immunogenicity subset, we will give you some refreshments at the clinic.

WHAT DOES GIVING BLOOD INVOLVE?

A small amount of your blood will be collected from you using a small clean needle. We will take blood before you get the second injection of vaccine and at 21 days after the second injection. To have your second blood sample taken you will need to come to the study clinic for an extra visit 21 days after you have received the second injection of vaccine. For children aged 6 years and older, we will collect approximately 5 mL (about 1 teaspoon) of blood each time you provide a blood sample.

As explained to you earlier, the blood samples you give will be used to test whether your body has produced antibodies in your blood against Ebola after vaccination. You will not receive the results of these tests because they are only for scientific research.

After we do our tests on your blood samples for the Immunogenicity Subset of the TUJIOKOWE study, we will destroy any remaining blood that you have given us. We will not use your blood for any other purpose except the antibody tests that we have explained in this assent form. Your blood samples will not be sold.

Your blood samples will be labelled with a code and not your name. Other information, such as sex, age, or health history might also be shared with other investigators, but your name will not be shared.

WHAT ARE THE POSSIBLE RISKS OF GIVING A BLOOD SAMPLE?

Giving a blood sample is generally safe but occasionally people experience pain, bruising, bleeding, or (very rarely) infection from giving a blood sample. The medical team will provide care if you experience side-effects from the blood sampling. They are available if you have any problems or questions.

WHAT ARE THE BENEFITS OF GIVING A BLOOD SAMPLE?

There is no direct benefit to you for providing blood samples in the Immunogenicity Subset of the TUJIOKOWE study. Your agreement to provide blood samples will help in the development of vaccines to prevent Ebola and, in the future, may help people in different parts of the world.

WHAT HAPPENS TO THE BLOOD SAMPLES COLLECTED FROM ME?

The blood samples collected from you during the Immunogenicity Subset of the TUJIOKOWE study, will only be used to understand how the Ebola vaccine works when the second injection is given more than two months after the first injection.

The samples will be coded in a way that only limited study staff can link them to you. Your blood samples will be tested in a laboratory in the United States. If any blood remains in DRC, it will be stored in the INRB laboratory in Goma. We will destroy all the samples in the United States and in Goma at the end of the study.

WHO CAN I TALK TO ABOUT GIVING BLOOD SAMPLES?

If you want to talk to someone about giving a blood sample or if you think you have been harmed by providing a blood sample, you can contact the following people:

1) The Principal Investigator responsible for this study

Professor Jean Jacques Muyembe<br>*Director General,* Institut National de Recherche Biomédicale, Kinshasa, DRC<br>Phone: 0898 949 289<br>Email: jjmuyembet@gmail.com<br><br>Local study representative<br>Dr Hugo Kavunga<br>Phone: 0823 875 153<br>Email: hugokavunga@gmail.com

2) The DRC ethics committees that approved this study

Professeur Félicien Munday<br>National Ethics Committee<br>Kinshasa-Gombe, DRC<br>Phone: 0998 419 816<br>Email: feli1munday@yahoo.fr
Professeur Willy Bongopasi<br>Comité d'éthique de l'école de santé publique<br>Université de Kinshasa<br>Phone: 0999 952 341<br>Email: bongopasi@gmail.com

If you have any questions about providing blood samples for the Immunogenicity Subset of the TUJIOKOWE study or about your rights, you may ask anyone on the study team at any time.

PARTICIPANT IMMUNOGENICITY SUBSET ASSENT FORM

Title: Immunogenicity Subset of the TUJIOKOWE Study

Principal Investigator of this study: Prof. JJ Muyembe

**Statements**	**Please sign or fingerprint each box**
I have read the information in this form about giving a blood sample at two visits for the Immunogenicity Subset of the TUJIOKOWE Study (or I have had this information explained to me by the study staff in a language that I understand). The purpose of providing blood samples and the procedures to give blood samples have been fully explained to me. I was able to ask questions and have all of these questions answered to my satisfaction.	
I understand that my participation is voluntary, that I can withdraw consent to provide blood samples at any time without giving any reason, and that this will not affect my participation in the TUJIOKOWE Study.	

I am aged 12-17 years and I agree to provide Immunogenicity Subset blood samples during my participation in the TUJIOKOWE study. (Please sign or put your fingerprint below)

		____/____/_____ *dd mon yyyy*
Printed name of child	Signature/fingerprint of child	Date

		____/____/_____ *dd mon yyyy*
Printed name of investigator	Signature of investigator	Date

Watson-Jones D, *et al. BMJ Open* 2022; 12:e055596. doi: 10.1136/bmjopen-2021-055596

Complete this section if the participant is illiterate:

Witness to Assent Interview

I witnessed the assent interview for the Immunogenicity Subset of the TUJIOKOWE Study in this document. I attest that I have explained the study information accurately to the participant and was understood to the best of my knowledge by the participant and that he/she has freely given their assent to participate in my presence.

		____/____/_____ *dd mon yyyy*
Printed name of impartial witness	Signature of impartial witness	Date

Attach ID barcode label below:
