# Supplementary material for: Protocol for a phase 3 trial to evaluate the effectiveness and safety of a heterologous, two-dose vaccine for Ebola virus disease in the Democratic Republic of the Congo
Source: BMJ Open. 2022 Mar 8;12(3):e055596. doi: 10.1136/bmjopen-2021-055596 (PMC8905941; doi:10.1136/bmjopen-2021-055596)
Supplement: Supplementary data [file bmjopen-2021-055596supp011.pdf]

Protocol for a phase 3 trial to evaluate the effectiveness and safety of a heterologous, two-dose vaccine for Ebola virus disease in the Democratic Republic of the Congo

Appendix 6

Data and Safety Monitoring Board (DSMB)

The following will be invited to participate on the Data and Safety Monitoring Board (DSMB) of this study. For detailed description of the DSMB responsibilities and activities, refer to the latest DSMB Charter.

Point of contact: Yazdan Yazdanpanah

Yazdan Yazdanpanah<br>Adult Clinician<br>yazdan.yazdanpanah@aphp.fr	Matthias Egger<br>Methodologist (DSMB Statistician)<br>matthias.egger@ispm.unibe.ch
Ebunoluwa Adejuyigbe<br>Paediatrician<br>ebunadejuyigbe@hotmail.com	Florian Marks<br>Vaccinologist/Epidemiologist<br>fmarks@ivi.int
Benoit Kebela Ilunga<br>Epidemiologist<br>kebelailunga@gmail.com	Tamara Giles-Vernick<br>Social Scientist<br>tamara.giles-vernick@pasteur.fr
Oumou Younoussa Bah Sow<br>Ethicist; President of the National Committee of Ethics for Health Research (CNERS)<br>oumou45@yahoo.fr	Marie Onyamboko<br>Epidemiologist; Kinshasa School of Public Health<br>akatshimarie@yahoo.fr

Trial Steering Committee (TSC)

The TSC is responsible for conducting the clinical study and for solving strategic problems that require high-level interventions.

Point of Contact: Richard Hatchett

Richard Hatchett/Gerald Voss<br>Coalition for Epidemic Preparedness Innovations<br>Richard.Hatchett@cepi.net<br>gerald.voss@cepi.net	Sylvain Yuma<br>Ministère de la Santé, DRC<br>sylvainyuma@gmail.com
Daniel Bausch<br>UK Public Health Rapid Support Team/LSHTM<br>Daniel.Bausch@phe.gov.uk	Jean-Jacques Muyembe-Tamfum (and Steve Ahuka)<br>Institut National de Recherche Biomédicale, DRC<br>jjmuyembet@gmail.com (amstev04@yahoo.fr)
Mike Ryan/Ana Maria Henao-Restrepo<br>World Health Organization<br>ryanm@who.int; henaorestrepoa@who.int	Jeremy Farrar/Josie Golding<br>Wellcome Trust<br>J.Farrar@wellcome.ac.uk<br>J.Golding@wellcome.ac.uk

Peter Piot/Deborah Watson-Jones<br>London School of Hygiene & Tropical Medicine<br>Peter.Piot@lshtm.ac.uk;<br>deborah.watson-jones@lshtm.ac.uk	Johan Van Hoof (and Macaya Douoguih)<br>Janssen Pharmaceuticals R&D<br>JVHOOF1@its.jnj.com (MDouogui@its.jnj.com)
Rebecca F Grais<br>Director of Research<br>Epicentre MSF, Paris, France<br>rebecca.grais@epicentre.msf.org	Isabelle Defourny/Clair Mills<br>MSF Operational Centre Paris<br>Médecins Sans Frontières, Paris, France<br>Isabelle.defourny@paris.msf.org;<br>Clair.Mills@paris.msf.org
Robert Kanwagi<br>World Vision<br>Robert.kanwagi@wveu.org	
