# Supplementary material for: Protocol for a phase 3 trial to evaluate the effectiveness and safety of a heterologous, two-dose vaccine for Ebola virus disease in the Democratic Republic of the Congo
Source: BMJ Open. 2022 Mar 8;12(3):e055596. doi: 10.1136/bmjopen-2021-055596 (PMC8905941; doi:10.1136/bmjopen-2021-055596)
Supplement: Supplementary data [file bmjopen-2021-055596supp012.pdf]

Protocol for a phase 3 trial to evaluate the effectiveness and safety of a heterologous, two-dose vaccine for Ebola virus disease in the Democratic Republic of the Congo

Appendix 7

Sponsor and Funders Responsibilities

Funders CEPI, Wellcome and DFID advised on the study design and protocol.

	Sponsor	Funders
Study design	A/R	R
Data collection	A/R	I
Data management	A/R	I
Data analysis	A/R	I
Data interpretation	A/R	I
Report writing	A/R	C
Report submission for publication	A/R	C

A = Accountable
R = Responsible
C = Contributing
I = Informed
