# Supplementary material for: Protocol for a phase 3 trial to evaluate the effectiveness and safety of a heterologous, two-dose vaccine for Ebola virus disease in the Democratic Republic of the Congo
Source: BMJ Open. 2022 Mar 8;12(3):e055596. doi: 10.1136/bmjopen-2021-055596 (PMC8905941; doi:10.1136/bmjopen-2021-055596)
Supplement: Author's manuscript [file bmjopen-2021-055596.draft_revisions.pdf]

BMJ Open

BMJ Open is committed to open peer review. As part of this commitment we make the peer review history of every article we publish publicly available.

When an article is published we post the peer reviewers' comments and the authors' responses online. We also post the versions of the paper that were used during peer review. These are the versions that the peer review comments apply to.

The versions of the paper that follow are the versions that were submitted during the peer review process. They are not the versions of record or the final published versions. They should not be cited or distributed as the published version of this manuscript.

BMJ Open is an open access journal and the full, final, typeset and author-corrected version of record of the manuscript is available on our site with no access controls, subscription charges or pay-per-view fees (http://bmjopen.bmj.com).

If you have any questions on BMJ Open's open peer review process please email info.bmjopen@bmj.com

BMJ Open

Protocol for a phase 3 trial to evaluate effectiveness and safety of a heterologous, two-dose vaccine for Ebola virus disease in the Democratic Republic of the Congo

Journal:	*BMJ Open*
Manuscript ID	bmjopen-2021-055596
Article Type:	Protocol
Date Submitted by the Author:	16-Jul-2021
Complete List of Authors:	Watson-Jones, Deborah; London School of Hygiene & Tropical Medicine<br>Kavunga-Membo, Hugo; L'Institut National de Recherche Biomédicale<br>Grais, RF; Epicentre<br>Ahuka, Steve; L'Institut National de Recherche Biomédicale<br>Roberts, Natalie; MSF<br>Edmunds, John; London School of Hygiene & Tropical Medicine<br>Choi, Edward; London School of Hygiene & Tropical Medicine, Department of Clinical Research<br>Roberts, Chrissy; London School of Hygiene & Tropical Medicine<br>Edwards, Tansy; London School of Hygiene & Tropical Medicine,<br>Camacho, Anton; Epicentre<br>Lees, Shelley; London School of Hygiene & Tropical Medicine<br>Leyssen, Maarten; Janssen Vaccines and Prevention BV<br>Spiessens, Bart; Janssen Vaccines and Prevention BV<br>Luhn, Kerstin; Janssen Vaccines and Prevention BV<br>Douoguih, Macaya; Janssen Vaccines and Prevention BV<br>Hatchett, Richard; Coalition for Epidemic Preparedness Innovations<br>Bausch, Daniel; London School of Hygiene & Tropical Medicine; Public Health England, UK Public Health Support Team<br>Muyembe, Jean-Jacques; L'Institut National de Recherche Biomédicale
Keywords:	PUBLIC HEALTH, VIROLOGY, IMMUNOLOGY, INFECTIOUS DISEASES, TROPICAL MEDICINE

SCHOLARONE™
Manuscripts

Protocol for a phase 3 trial to evaluate effectiveness and safety of a heterologous, two-dose vaccine for Ebola virus disease in the Democratic Republic of the Congo

Deborah Watson-Jones[1,2], Hugo Kavunga-Membo[3], Rebecca Grais[4], Steve Ahuka[3], Natalie Roberts[5], W. John Edmunds[1], Edward M. Choi[1], Chrissy H. Roberts[1], Tansy Edwards[1], Anton Camacho[4], Shelley Lees[1], Maarten Leyssen[6], Bart Spiessens[6], Kerstin Luhn[6], Macaya Douoguih[6], Richard Hatchett[7], Daniel G. Bausch[1,8], Jean Jacques Muyembe[3], DRC-EB-001 protocol writing team[9].

Correspondence to Deborah Watson-Jones; Deborah.watson-jones@lshtm.ac.uk

Keywords: Public health, virology, immunology, infectious diseases, tropical medicine

Word Count: 5070

For peer review only

Abstract

Introduction

Ebola virus disease (EVD) continue to be a significant public health problem in sub-Saharan Africa, especially in the Democratic Republic of the Congo (DRC). Large-scale vaccination during outbreaks may reduce virus transmission. We established a large population-based clinical trial of a heterologous, two-dose prophylactic vaccine during an outbreak in eastern DRC to determine vaccine effectiveness.

Methods and analysis

This open label, non-randomised population-based trial enrolled eligible adults and children aged one year and above. Participants were offered the two-dose candidate EVD vaccine regimen VAC52150 (Ad26.ZEBOV, MVA-BN-Filo), with the doses being given 56 days apart. After vaccination, serious adverse events (SAEs) were passively recorded until 1 month post-dose 2. 1000 safety subset participants were telephoned at 1 month post-dose 2 to collect SAEs. 500 pregnancy subset participants are contacted to collect SAEs at D7 and D21 post-dose 1 and at D7, 1 month, 3 months and 6 months post-dose 2, unless delivery is before these time points. The first 100 infants born to these women are given a clinical examination 3 months post-delivery. Due to COVID-19 and temporary suspension of dose 2 vaccinations, 50 paediatric and 50 adult participants were enrolled into an immunogenicity subset to examine immune responses following a delayed second dose. Samples collected pre-dose 2 and at 21 days post-dose 2 will be tested using the EBOV GP Filovirus Animal Nonclinical Group (FANG) enzyme-linked immunosorbent assay. For qualitative research, in-depth interviews and focus group discussions are being conducted with participants or parents/care-providers of paediatric participants.

Ethics and dissemination

Approved by Comité National d'Ethique et de la Santé du Ministère de la santé de RDC, Comité d'Ethique de l'Ecole de Santé Publique de l'Université de Kinshasa, the LSHTM Ethics Committee and the MSF Ethics Review Board. Findings will be presented to stakeholders and conferences. Study data will be made available for open access.

Trial registration number

For peer review only - http://bmjopen.bmj.com/site/about/guidelines.xhtml

NCT04152486

STRENGTHS AND LIMITATIONS OF THIS STUDY

- This large-scale trial is the first designed to measure vaccine effectiveness of a two-dose prophylactic Ebola vaccine regimen through open label delivery of vaccine to general populations in an Ebola-affected area. This is also the first clinical trial to administer this vaccine regimen to pregnant women.
- The pragmatic study design facilitated vaccine delivery and encompassed measurement of vaccine effectiveness during an active Ebola epidemic where the future location of new cases is uncertain.
- A test negative case control analysis was incorporated for estimating vaccine effectiveness in protecting against laboratory-confirmed EVD in a situation where a randomised controlled trial with a control arm was not possible.
- A non-randomised design is a limitation of this study.

For peer review only

For peer review only - http://bmjopen.bmj.com/site/about/guidelines.xhtml

INTRODUCTION

Background and rationale

Ebola virus disease (EVD) is caused by Ebola viruses (EBOV) and has a case fatality rate ranging from 30-90%.[1] Bats are thought to be the reservoir, with human infection resulting from contact with excrement or saliva from bats or other intermediate animal hosts.[2-5]. Large outbreaks are fueled almost exclusively by subsequent human-to-human transmission.[2,6,7]

Vaccination is now a proven preventative measure for EVD control. During the 2013-16 EVD outbreak in West Africa, a single dose of the rVSV-ZEBOV-GP vaccine (ERVEBO®, Merck & Co., Inc.) given through a phase 3 cluster-randomised, ring-vaccination trial in Guinea showed 100% protective efficacy against EVD between day 10 and day 31 post-randomisation.[8] In 2016, the World Health Organization (WHO) Strategic Advisory Group of Experts on Immunization (SAGE) recommended the rapid deployment of rVSV-ZEBOV-GP for EVD outbreaks under an expanded access (compassionate use) protocol and the vaccine was licensed by the Democratic Republic of the Congo (DRC), Burundi, Ghana and Zambia in February 2020.[9,10]

Since the discovery of EBOV in 1976, the DRC has recorded 12 outbreaks. On 1 August 2018, the tenth EVD outbreak was declared in North Kivu Province.[11] The outbreak progressively expanded across a large, highly populated area, characterized in recent years by civil unrest, armed militia groups, internally displaced persons and a substantial United Nations peacekeeping mission.[12] The outbreak response focused on strategies that were previously adopted for the control of EVD outbreaks but was periodically hindered by attacks on Ebola Treatment Centres (ETCs) and on response workers. New cases continued to emerge in North Kivu and neighbouring provinces, prompting consideration of additional vaccine programmes that might assist in preventing the spread of this infection to unaffected communities. In early 2019, WHO conducted an evaluation of the suitability of candidate EVD vaccines for further clinical study and recommended several vaccines including VAC52150 (Ad26.ZEBOV, MVA-BN-Filo), a heterologous two-dose prophylactic vaccine manufactured by Janssen Vaccines and Prevention B.V.[13]

For peer review only - http://bmjopen.bmj.com/site/about/guidelines.xhtml

Objectives

Following the WHO evaluation, we are conducting an open label, non-randomised, population-based trial in North Kivu Province. This trial is named "Evaluation of a heterologous, two-dose preventive Ebola vaccine for effectiveness and safety in the Democratic Republic of the Congo". The protocol number is DRC-EB-001. The primary objective is to estimate the vaccine effectiveness (VE) of Ad26.ZEBOV, MVA-BN-Filo for prevention of EVD in adults and children aged 1 year or above. Secondary objectives are to: (1) assess the safety of Ad26.ZEBOV, MVA-BN-Filo, (2) estimate the coverage of dose 1 and dose 2 of Ad26.ZEBOV, MVA-BN-Filo overall and in different target groups (by gender, age and location), and (3) to explore the knowledge and perceptions of persons eligible for large-scale delivery of a preventative EVD vaccine with a two-dose vaccine strategy. An exploratory objective is assessing immunogenicity of Ad26.ZEBOV, MVA-BN-Filo in a subset of participants who received dose 2 outside the recommended interval between doses 1 and 2. This is the first study providing Ad26.ZEBOV, MVA-BN-Filo to pregnant women (Table 1).

METHODS

The vaccine

The vaccination regimen comprised two vaccine candidates, both given as a 0.5ml intramuscular injection in the upper section of the deltoid muscle or in the thigh in the case of young children:

1) Ad26.ZEBOV: a monovalent vaccine expressing the full-length glycoprotein (GP) from EBOV Mayinga, that is produced in the human cell line PER.C6®, $5x10^{10}$ viral particles and given at day zero
2) MVA-mBN226B, or Modified Vaccinia Ankara (MVA)-BN®-Filo: a multivalent vaccine expressing the GP of EBOV (100% homologous with the GP expressed by Ad26.ZEBOV), Sudan, and Marburg Musoke viruses; and the nucleoprotein of Taï Forest virus, $1x10^8$ infectious units given at day 56 (-14 day +28 day).

This regimen has been evaluated for immunogenicity and safety in 11 clinical trials in the United Kingdom, United States, and East and West Africa, including in previously EVD-affected countries.[14-16, 18-22] When administered in a 0, 56 day schedule in Phase 2 and 3 studies, geometric mean concentrations of immunoglobulin G (IgG) binding antibody to the EBOV

For peer review only - http://bmjopen.bmj.com/site/about/guidelines.xhtml

glycoprotein (GP) measured by the Filovirus Animal Non-Clinical Group (FANG) Enzyme-Linked Immunosorbent Assay (ELISA) 21 days post-dose-2 were 3,810-11,790 EU/mL in healthy participants. The FANG ELISA assay has been validated for use in human sera.[17] Unblinded safety data from 2,390 adults showed only mild-to-moderate adverse events of short duration with no sequelae.[18] No safety concerns were raised in HIV-infected individuals.[19] In 649 children, the vaccine was highly immunogenic and had similar safety profile as adults, with no Suspected Unexpected Serious Adverse Reactions.[20] The European Medicines Agency granted marketing authorization for Ad26.ZEBOV, MVA-BN-Filo on 1 July 2020.[21, 22]

For this study, vaccine was donated by Janssen Vaccines and Prevention B.V.. Vials were stored and shipped according to Good Manufacturing Practice guidelines with maintenance of the cold chain from Belgium to the vaccine depot in Goma, DRC, and onto the vaccination clinics.

Trial design

The protocol development team and investigators considered various trial designs that would have allow estimation of vaccine efficacy or effectiveness in the context of an epidemic and vaccination activities with the rVSV-ZEBOV-GP vaccine. The value of placebo-controlled randomised controlled trial (RCT) designs was recognized but, given the ongoing EVD outbreak at the time of initiation of the study and taking into account socio-cultural sensitivities and civil instability in the region, an RCT was not considered feasible or ethical. Rather, we adopted a test-negative case-control design, in which vaccine effectiveness (VE) is estimated by comparing the history of vaccination in cases (test positives) compared to that in controls (test negatives), among individuals presenting with suspected EVD, with cases and controls defined retrospectively after EVD test results were confirmed. The test-negative design allows evaluation of VE in the general population without randomization, and has been proposed as a valid approach for evaluation of EVD vaccines when RCTs are not feasible.[23,24] The validity of this approach has been demonstrated by comparing results from test-negative case-control studies with those from randomized clinical trials of influenza and rotavirus vaccines. The two type of study yielding similar VE estimates.[25-27]

For peer review only - http://bmjopen.bmj.com/site/about/guidelines.xhtml

Trial setting

After discussion with local authorities, we initiated the trial in two administrative health areas in Goma, the provincial capital of North Kivu Province (population 634,197 in 2020). The choice of these locations was determined based on their potential risk for EVD transmission since there were strong ethnic, family and business links between these two health areas and the epidemic epicentres of Beni and Butembo, with frequent travel between the communities. Provincial administrative and health authorities provided data on the number of residents of these areas as well as the number of individuals working in health and community health settings. These people were provided with information about when and where vaccination would take place. We planned to subsequently move to health areas closer to the outbreak epicentre, guided by data on estimated risk of EVD, population density, security issues and transport links from Goma. However, plans to expand into new study sites were abandoned when the outbreak came under control and was declared over on 25 June 2020. As a consequence there was no possibility of acquiring vaccine effectiveness data through the trial as the epidemic had not spread to the initial trial areas.

Sample size

The required sample size for the primary effectiveness evaluation was 110 cases (test positives), to provide 80% power with alpha = 0.05 for a two-sided test to demonstrate 70% VE under assumptions for coverage (percentage of vaccinated test negative individuals) ranging from 20-70% and ratio ranging from 1-4 test negatives per positive case (Table 3).

This sample size also provides 80% power to demonstrate 90% VE under assumptions for coverage ranging from 10-70% and ratio of test positives to test negatives ranging from 1-4 test negatives to a test positive case. It will also provide 80% power to demonstrate 50% VE under assumptions for coverage ranging from 50-70% and ratio of test positives to test negatives ranging from 2-4 test negatives to a test positive case.

We estimated that vaccination of approximately 500,000 people with Ad26.ZEBOV, MVA-BN-Filo in transmission areas would achieve the sample size of 110 confirmed EVD cases under the following assumptions: i) an EVD incidence of 4.1/10,000 in the absence of Ad26.ZEBOV/MVA-BN-Filo-induced immunity, corresponding to the median attack rate per health area after the first year of the epidemic in the DRC; and ii) 30% of confirmed cases had

For peer review only - http://bmjopen.bmj.com/site/about/guidelines.xhtml

received the rVSV-ZEBOV-GP (in accordance with surveillance data) and would therefore be excluded from the effectiveness analysis. We assumed a homogeneous attack rate, while recognising that it could vary according to the location of vaccine deployment and the evolution of the epidemic.

After the VE analysis had been conducted based on 110 cases, the study team planned discussions to review the findings and the value of continuing to enrol participants.

Two subsets of individuals, 1000 vaccinated individuals (500 pre-dose 1 and 500 pre-dose 2) and 500 pregnant participants (250 pre-dose 1 and 250 pre-dose 2), were enrolled for more active follow-up for safety reporting (Table 2). The sample size allowed for 95% probability to detect at least one vaccine related SAE if the true incidence of that SAE is at least 1.2% with 250 individuals, 0.6% with 500 individuals, or 0.3% with 1000 individuals.

Community engagement and feedback

Prior to recruiting participants, we set up a specific community engagement structure. Its mission was to inform, sensitize and engage directly with the local population. Initially overseen by Médecins Sans Frontières France (MSF), World Vision was involved in the introduction of specific techniques, including mobile messaging.

Within each health area, Community Advisory Committees (CACs) were constituted. They conducted initial meetings with local political and administrative leaders, social groups and organisations, local associations and other nongovernmental organisations, and traditional and religious leaders, and their input was included in the planning of the trial implementation. We then conducted regular, separate discussion meetings with these different groups every two weeks to receive and integrate feedback on social harms, individual and community level risks, perceptions about the vaccine and the study, legal or administrative complaints and vaccination implementation issues.

Eligibility, recruitment and enrolment

Using pre-defined eligibility criteria (Box 1), we invited all adults and children aged one year or greater, including pregnant and breastfeeding women, to participate if they lived or worked

For peer review only - http://bmjopen.bmj.com/site/about/guidelines.xhtml

in the selected health zones and, at the time of vaccination, planned to remain there for one month post-dose 2. Health workers were specifically encouraged to participate. We provided information about the study to local populations through the distribution of posters and flyers, banner displays in the target health areas, radio and television broadcasts and interviews during church gatherings and through interactive street visits using loudspeakers. Until the outbreak was declared over, we provided participants with information on where to present for care if they developed EVD symptoms and informed participants that they could still receive rVSV-ZEBOV-GP as recommended by WHO if eligible.(11) We instructed Ebola Emergency Treatment Centres (ETCs) on how to report that a presenting patient had received Ad26.ZEBOV, MVA-BN-Filo. We required a parent or caregiver to accompany any child aged less than 18 years to the study vaccination site.

Intervention delivery

We established six study vaccination sites within the participating health zones in Goma. Communicating in Swahili, French or a local dialect, Nande, we conducted symptom screening and a temperature check for possible EVD for participants at a triage desk before they could proceed into the clinic. We provided a short medical consultation to participants presenting at triage with fever and/or illness and including, if appropriate, a test for malaria, and treatment if positive, as well as free treatment for other minor illnesses according to national medical protocols. A post-vaccination medical consultation was also offered for any participants complaining of illness during the observation period. We then referred participants to the nearest appropriate health facility if any further medical intervention was required.

At the first visit, delegated medical staff of the trial confirmed eligibility, explained the study aims and procedures, answered any questions, and sought informed consent (and informed assent for children aged 12-17 years), with witnessed consent for illiterate individuals. We offered urine pregnancy tests at the time of vaccination if a participant believed that she might be pregnant and/or if her last menstrual period was more than one month ago. We gave participants the Ad26.ZEBOV vaccination and asked them to remain at the site for 15 minutes to monitor for any immediate adverse effects. We provided participants with a laminated vaccination card with their photograph, which we asked them to present at subsequent visits. Enrolment and dose 1 vaccination took place between 14 November 2019-29 February 2020.

For peer review only - http://bmjopen.bmj.com/site/about/guidelines.xhtml

We collected data on place of residence and telephone numbers from all trial participants and contacted them through mobile phone calls and automated SMS to remind them to attend for dose 2 and followed up, with home visits if these were needed. At the clinic visits for dose 2, similar triage, eligibility and health checks were conducted. Participants who attended more than 84 days post-dose 1 were still able to receive the second dose until the study vaccination sites closed on 10 February 2021.

After the study start, all individuals presenting to an ETC, peripheral health facility or an EVD testing centre were expected to have their rVSV-ZEBOV-GP and Ad26.ZEBOV, MVA-BN-Filo vaccination status confirmed as part of routine data collection, with this information being recorded in the routine Ministry of Health EVD surveillance database. This included recording the participant ID number from the study vaccination card. If the patients were not able to present their cards at the ETC but mentioned being a study participant, ETC staff requested the family to bring the card to the ETC. For lost cards, we instructed ETC staff to collect the patient's name, vaccination and clinic site details, and, if possible, the last vaccination date, and provide this information to the study data management team to retrieve the participant's ID and manually complete the ETC line-list.

Due to the waning EVD outbreak, the Trial Steering Committee decided to suspend dose 1 vaccination on 29 February 2020, after 20,427 participants had been vaccinated. Dose 2 vaccination continued, recognizing that, while the objective of evaluating VE could no longer be met, the other objectives were still achievable. However, due to the COVID-19 outbreak in DRC, all study vaccinations were suspended from 10 April to14 September 2020, while special precautions to ensure the safety of participants and staff from COVID-19 were established. As a result of suspension of activities, the protocol was amended (10 August 2020, version 7.0) to include immunogenicity assessment of the impact of delayed dose 2 vaccination beyond 84 days post-dose 1 and safety follow up windows (Table 2). Following implementation of infection control measures vaccination continued and dose 2 delivery was completed on 9 February 2021.

In addition to the main study, we enrolled participants into three subsets, with consent sought in separate sections in the main study informed consent and assent forms (Appendices 1-5):

1) Immunogenicity sub-study: 57 non-pregnant adults and 90 children (54 children aged 4-11 years and 36 adolescents aged 12-17 years) were enrolled in an immunogenicity sub-study. At selected vaccination centres, we collected venous blood samples (5 mL

For peer review only - http://bmjopen.bmj.com/site/about/guidelines.xhtml

per visit for adults and children aged 6-17 years and 2.5mL per visit for children aged 4-5 years) from consenting participants at the dose 2 visit and 21 days later (window -3/+7 days). We made 0.5mL serum aliquots, which were stored at -20°C in the INRB laboratory in Goma. One aliquot was shipped to Q2 Solutions in the USA for measurement of IgG binding antibodies to EBOV GP by FANG ELISA. The remaining aliquots are being stored in Goma until the end of study and will then be destroyed. We provided participants with an immunogenicity subset attendance card with a photograph and ID number for identification at the 21 days post-dose 2 visit. We deferred blood drawing if participants were unwell at the second visit. Enrolment and site visits of this subset were completed on 7 December 2020. Details of laboratory handling of biological specimens can be found in the Laboratory Analytical Plan.

2) Safety subset: 1000 participants (approximately 500 adults and 500 children pre-dose 1) were asked to consent to a telephone call at 1 month post-dose 2 to collect data on SAEs.

3) Pregnancy subset: up to 500 pregnant participants (250 each, pre-dose 1 and pre-dose 2) were asked to consent to receive telephone calls to collect SAEs at D7 and D21 post-dose 1, and at D7, 1 month, 3 months and 6 months post-dose 2 (unless delivery has occurred before these time points) and data are being collected on pregnancy outcomes as described below.

Safety measurements

Following each vaccination, we gave participants instructions to contact the study team for any SAEs occurring up to one month post-dose 2 and where to seek care for medical problems, including antenatal care for pregnant women (Table 2). Whenever possible, we documented the diagnosis or syndrome related to SAEs, rather than multiple symptoms. Investigators then assessed any potential causal relationship to vaccination. We followed up SAEs to resolution or stabilization, irrespective of severity or whether considered vaccine-related. We allowed unscheduled study follow up visits on adverse events at the investigator's discretion.

Follow-up of pregnant women is ongoing. We compiled a registry of pregnant women and we encouraged women who become pregnant within 1 month of any vaccine dose to contact the team. We are telephoning all known pregnant women 1-3 months after delivery to record birth

For peer review only - http://bmjopen.bmj.com/site/about/guidelines.xhtml

outcomes or, when possible, a study clinician visits them in person if they are not contactable by telephone. In the pregnancy subset, we actively follow by telephone 250 women who were pregnant at the time of dose 1 and up to 250 who become pregnant within 30 days (one month) of administration of either dose 1 or 2. We make calls to collect SAE data at D7 (-3/+7 day) and D21 (-6/+7 day) post-dose 1, D7 (-3/+7 day) post-dose 2, and at 1 month (-7 day/+1 month), 3 months (+/-14 day) and 6 months (-14/+28 day) post-dose 2 (unless delivery occurs before these time points). We are performing a clinical examination on the first 100 infants born to women in the pregnancy subset at around 3 months of age (ranging from 1-6 months). We offer a small bag of rice, cassava or equivalent to mothers returning to the clinic for infant safety follow-up visits.

In the safety subset, we actively followed the first 500 adults and first 500 children enrolled by telephone one month (-7 days/+1 month) after administration of dose 2.

For acute medical problems, we provide care to participants with referral to health clinic and hospitals as necessary. The trial sponsor and funder have put in place global and local clinical trial insurance covers to compensate any potential harm to participants.

Social science methods

We are using multiple modalities to explore the socio-economic and socio-cultural contexts of EVD and the study, including perception of illness and disease, trust in medical research and the motives for the study, key community dynamics and power relations, barriers and opportunities. This information can be used to trace any ongoing rumours and concerns emerging at the community level and to provide appropriate feedback to the study team.

The social science component of the study includes the following:

1) 30 In-depth Interviews: 5 women, 5 men, 5 girls and 5 boys aged 12-17 years old and 10 parents/care-providers of children aged 1-11 years, post-dose-1 or 2.
2) Eight Focus Group Discussions: Including adult participants, parents of children aged 1-11 years, and boys and girls aged 12-17 years.
3) Up to 10 Key Informant Interviews: Local and national stakeholders.
4) Participant exit interviews at vaccination visits to document immediate concerns related to clinic experiences.

For peer review only - http://bmjopen.bmj.com/site/about/guidelines.xhtml

5) Ethnographic observation in study clinics and key sites (e.g. markets, motorbike parking grounds).

At the beginning of all interviews, the social science team ask participants to state that they consent to taking part, recording this both digitally and by hand on the consent form, or only by hand if permission is not given for digital recording. Parents are being asked to provide consent for children under 17 years and children aged 12-17 years to provide assent. Interviews are being conducted in the participant's/parent's/guardian's primary language. Exit interviews are extremely basic and rapid and simply record whether participants had any immediate concerns they wished to report. If in-person interviews are not possible, we conduct phone interviews with adult participants, with researchers reading out information from an information sheet.

Interviews are conducted in French, Swahili, or Nande and then translated to English. We remove personal identifiable information prior to uploading recordings to a SharePoint which is only accessible to the study team. We give each interview participant a unique ID number and delete their phone numbers from study databases immediately after use.

OUTCOMES

The primary study outcomes are:

1) Numbers and odds of vaccination status in EVD cases and in EVD-negative controls in a test negative case control study with a target sample size of 110 laboratory confirmed EVD cases matched to controls who test negative for EVD. Effectiveness is derived from the odds ratio for vaccination in cases compared to controls to calculate VE
2) Number and proportion of adults and children with solicited and unsolicited SAEs using data on SAEs within one month of post-dose 2 that are considered related to vaccination with Ad26.ZEBOV, MVA-BN®-Filo in adults and children.

The secondary outcomes include:

1) The number and proportion of pregnant participants with solicited and unsolicited SAEs, including congenital abnormalities in their infants.
2) the number and proportion of adults and children receiving dose 1.
3) The number and proportion of adults and children receiving dose 2.

For peer review only - http://bmjopen.bmj.com/site/about/guidelines.xhtml

DATA COLLECTION, MANAGEMENT AND ANALYSIS

Data Collection

Data collection is ongoing. We collect data using electronic case record forms (eCRFs) developed using Open Data Kit (ODK) software on password protected tablets, and on standardized paper CRFs when eCRF use is not possible.[31] We designed a modular system of ODK forms in English and French to collect data on different aspects of the study, including consent, vaccinations and errors on eCRF completion. Prior to starting the study, we provided and reviewed guidelines for CRF completion with study site personnel. Automated programs in R or STATA summarise study progress and create line-lists for follow-up and dose 2 visits, and to identify errors. Data can be exported as delimited text files. Automated checks ensure data completion. Study team members receive daily updates and reports via a password protected dashboard. eCRFS are also transferred into REDCAP GCP compliant software for an audit trail of data cleaning. Data collected in paper-based CRFs are double-entered directly into a RedCAP database.

After encryption, data are sent via end-to-end encrypted https protocols to a server hosted at the London School of Hygiene & Tropical Medicine (LSHTM), with ongoing transfer of data to servers hosted by INRB and Epicentre via SSH File Transfer Protocol (sFTP). Only the data management team at Epicentre and INRB are able to decrypt the raw data. Study personnel save, store, back-up, transfer and share data in encrypted file formats and over encrypted transmission routes. To the extent possible, we use de-identified data for operational and analytic purposes. Permissions to decrypt data are limited to key personnel. Working copies of databases such as those used by staff who are responsible for progress monitoring and statistical analysis are automatically stripped of personal identification data via a process applied by data managers at Epicentre.

We anonymise interview transcripts and field notes and download them to password protected and encrypted computers for transcription and translation and analysis using NVIVO software. We keep all recordings, transcripts and field notes in locked filing cabinets at the study's Goma data centre, before planned long-term storage at INRB Kinshasa. Details of database validation,

For peer review only - http://bmjopen.bmj.com/site/about/guidelines.xhtml

data collection, coding, security, automatic, query, cleaning and safety data reconciliation can be found in the Data Management Plan.

Vaccine effectiveness analysis

We define cases (test positives) as laboratory-confirmed EVD cases as per DRC Ministry of Health centralized reporting and controls (test-negatives) as those with suspected EVD but whose laboratory tests were negative. Prior to the end of the outbreak, we envisaged that identification of controls would be retrospective, blind to vaccination status, on individuals residing or working in areas that were offered vaccination prior to presentation with symptoms at health facilities, with geographical matching to allow cases and controls to have had equal opportunities to have been vaccinated. Frequency matching or adjustments using regression techniques for other characteristics such as age, sex and possibly also exposure to EBOV, were to be described in the Statistical Analysis Plan.

Since the likely ratio of test-negatives to test-positives at screening centres was unknown, it was recognised that, if a high number of individuals with a very low percentage of confirmed cases presented in a short period of time, random sampling of test-negatives on a frequency matched basis may have been be required.

If the duration of Ebola outbreak had permitted measurement of vaccine effectiveness, individuals who received rVSV-ZEBOV-GP, or the first but not the second dose of Ad26.ZEBOV, MVA-BN-Filo or who received the second dose less than 21 days prior to testing for EVD would have been excluded from the primary analysis.

For the primary analysis, the odds of having been fully vaccinated (having received the 2 doses, in the right order, at least one month apart, at least 21 days before the onset of symptoms) would have been compared with the odds of not being vaccinated between the cases and controls through the odds ratio (OR), providing an estimate of VE: (VE(%)=(1-OR)x100), with regression analyses allowing for appropriate account of any matching and adjustment for potential confounders.

We also planned to examine VE of at least one dose of the vaccine and development of disease within 21 days after the second dose as secondary analyses.

Safety analysis

For peer review only - http://bmjopen.bmj.com/site/about/guidelines.xhtml

Safety analyses will include all participants receiving at least dose 1. We will summarise the incidence of serious adverse events (SAEs) overall, by time since vaccination and by age and whether or not vaccination was received during pregnancy. The incidence of SAEs will also be tabulated by MedDRA preferred term and system organ class (Medical Dictionary for Regulatory Activities version 23.1).

Immunogenicity subset analysis

We will determine the number of overall participants with detectable antibodies and maximum titres post-vaccination, including analysis by age group and by time since dose 1 vaccination. We will make descriptive comparisons with other studies of the same vaccine in sub-Saharan Africa where dose 2 was given according to the recommended 56-day interval and, for adults, with other delayed dose 2 immunogenicity data. We will analyse continuous immunologic parameters (e.g. geometric mean concentration of IgG binding antibodies to EBOV GP) and responder rates, and assess the impact of extending the interval between the two doses.

More detailed information on analysis populations, data handling and sub-group analyses is provided in the Statistical Analysis Plan.

Qualitative research analysis

We are conducting inductive and deductive analysis of qualitative data collected by in-depth interviews, focus group discussions and exit interviews. All recordings are being transcribed into French and imported to NVIVO 12. Data are being coded by constant comparative methods to create emergent themes.

Trial Management

LSHTM is the study sponsor. A Trial Steering Committee holds regular meetings by teleconference to monitor study progress (Appendix 6). An independent Data Safety and Monitoring Board (DSMB) periodically reviews the safety data and meets according to the participant recruitment timeline specified in the DSMB Charter (Appendix 6). Fatal, life-threatening events and Suspected Unexpected Serious Adverse Reactions are reported to the DSMB within 48 hours and other SAEs within 7 days. An independent Good Clinical Practice (GCP) monitor checks to compliance with the protocol, GCP, standard operating procedures and informed consent processes through review of documents for 5% of study participants

For peer review only - http://bmjopen.bmj.com/site/about/guidelines.xhtml

through on-site visits and remote monitoring. There will also be one on-site GCP audit by a sponsor representative not involved in conducting the trial before primary data collection has completed.

ETHICS AND DISSEMINATION

The protocol was reviewed and approved by the Comité National d'Ethique et de la Santé and Comité d'Ethique de l'Ecole de Santé Publique in DRC, the LSHTM Ethics Committee and the Ethics Review Board of Médecins Sans Frontières (Table 1). This study is being implemented according to the Declaration of Helsinki principles and the principles of the International Conference on Harmonisation (ICH) Guideline for GCP. Since the original approved protocol version 5.0, dated 12 September 2019, there has been 1 protocol amendment. This article is based on protocol version 7.0, dated 10 August 2020. All versions and amendments have been approved by the four ethics committees listed above and by the DRC Direction de la Pharmacie et du Médicament. The study has been registered in ClinicalTrials.gov (ClinicalTrials.gov Identifier: NCT04152486).

The population of North Kivu Province is considered vulnerable. Study participation is strictly voluntary. Participants can withdraw from the trial at any time. If provided, the reasons for withdrawal will be recorded. Both communities and, at the time of the outbreak, individuals were informed that study participation did not mean that other EVD infection protection and control measures should be discarded and that they should endeavour to attend for the second dose.

Results of the study will be presented to relevant stakeholders in DRC, shared with WHO's technical expert groups and published in Open Access journals. Any research articles generating from this trial will be subject to the Publication Policy and the Authorship Guidelines agreed by all partners.

Data Availability Statement

The rights of study subjects and partners, the sharing of data between partners and the transfer of data to external third party will be governed by the Data Sharing Agreement. Deidentified participant-level data collected in this trial will be disseminated through a FAIR-compliant data

For peer review only - http://bmjopen.bmj.com/site/about/guidelines.xhtml

repository, such as the LSHTM Data Compass (https://datacompass.lshtm.ac.uk/), from 6 to 60 months after the publication of the main trial results. Other study documents (e.g. full protocol, statistical codes, Statistical Analytical Plan, DSMB Charter) will be available upon request to Deborah Watson-Jones (corresponding author, ORCID: 0000-0001-6247-1746), Tansy Edwards (study statistician, ORCID: 0000-0002-6110-014X) or Edward Choi (study coordinator, ORCID: 0000-0002-8148-120X).

Patient and Public Involvement Statement

Because we invited the general population to participate in this trial, no patients were involved in the discussions on trial design, conduct, reporting or dissemination. Prior to trial commencement, we engaged the Goma population through consultation with local authorities and leaders. Refer to the main article for details.

Author affiliations

[1] London School of Hygiene & Tropical Medicine, London, United Kingdom
[2] Mwanza Intervention Trials Unit, National Institute for Medical Research, Mwanza, Tanzania
[3] L'Institut National de Recherche Biomédicale (INRB), Kinshasa, Democratic Republic of Congo
[4] Epicentre, Paris, France
[5] Médecins Sans Frontières France, Paris, France
[6] Janssen Vaccines and Prevention B.V., Leiden, Netherlands
[7] Coalition for Epidemic Preparedness Innovations (CEPI), Oslo, Norway
[8] UK Public Health Rapid Support Team, Public Health England, United Kingdom
[9] DRC-EB-001 protocol writing team

PROTOCOL WRITING TEAM FOR DRC-EB-001 TRIAL

London School of Hygiene & Tropical Medicine (LSHTM)
Deborah Watson-Jones
John Edmunds
Edward M Choi
Chrissy H Roberts
Shelley Lees
Tansy Edwards
Daniela Manno
Peter G Smith
Brian Greenwood
Daniel G Bausch

L'Institut National de Recherche Biomédicale (INRB)

For peer review only - http://bmjopen.bmj.com/site/about/guidelines.xhtml

Jean-Jacques Muyembe-Tamfum
Steve Ahuka
Hugo Kavunga-Membo

EPICENTRE
Rebecca Grais
Susan Rattigan
Anton Camacho
Grace Mambula

MSF France
Natalie Roberts
John Johnson
Patient Mumbere Kighoma
Marie Burton

Janssen Vaccines and Prevention B.V.
Maarten Leyssen
Macaya Douoguih
Bart Spiessens

Coalition for Epidemic Preparedness Innovations (CEPI)
Richard Hatchett
Nathalie Imbault
Gerald Voss
Melanie Saville

Inserm-IRD-Université de Montpellier
Eric Delaporte

World Health Organization
Ira M Longini

Acknowledgements

We thank participants, medical and nursing staff for their participation in this study and World Vision for community engagement support.

Contributors

DWJ, JJM, DB, CR, RG, JE, NR, PS, TE, HKM, ML, AC, EC, BG, JF, IL, ED and SA conceived and designed the study. DWJ and EC wrote the protocol with inputs from RG, SR, DB, HKM, PS, NI, TE, CR, JE, BG, SL, AC. TE and PS provided statistical advice. Authors and the protocol writing team approved the final submitted manuscript.

For peer review only - http://bmjopen.bmj.com/site/about/guidelines.xhtml

Funding statement

This study is supported through funds from the Coalition for Epidemic Preparedness Innovations (CEPI) and the Paul G. Allen Family Foundation. This work was also supported by the Department for International Development (DFID) and Wellcome [220506/Z/20/Z] and by the European Union's Horizon 2020 research and innovation programme under grant agreement No 857935. This publication reflects only the authors' view and the European Commission is not responsible for any use that may be made of the information it contains. CEPI, Wellcome and DFID advised on the study design and protocol (Appendix 7).

Competing interests

DWJ, BG and LSHTM are partners on two research consortia (EBOVAC1, EBOVAC3) with Janssen Vaccines and Prevention B.V. funded by the European Commission.

Patient consent for publication

Not required.

For peer review only

Table 1. The World Health Organization Trial Registration Data Set for the DRC-EB-001 trial

Data category	Information
**Primary registry and trial identifying number**	ClinicalTrials.gov NCT04152486
**Date of registration in primary registry**	5 November 2019
**Source(s) of monetary or material support**	Coalition for Epidemic Preparedness Innovations (CEPI), the Paul G. Allen Family Foundation and Wellcome
**Sponsor**	London School of Hygiene & Tropical Medicine (LSHTM)
**Sponsor contact and Contact for public queries**	Deborah Watson-Jones deborah.watson-jones@lshtm.ac.uk
**Public title**	Evaluating the Effectiveness and Safety of a Two-dose Ebola Vaccine in the Democratic Republic of the Congo
**Scientific title**	Evaluation of a heterologous, two-dose preventive Ebola vaccine for effectiveness and safety in the Democratic Republic of the Congo
**Countries of recruitment**	The Democratic Republic of the Congo
**Health condition(s) studied**	Ebola virus disease (EVD)
**Intervention(s)**	Ad26.ZEBOV, MVA-BN-Filo
**Key inclusion criteria**	Healthy volunteers<br>Aged ≥ 1 year<br>Both sexes (including pregnant and breastfeeding women)
**Key exclusion criteria**	Known EVD infection<br>Having received any experimental EVD vaccines within 1 month
**Study type**	Interventional
**Allocation**	Open label, single arm
**Primary purpose**	Prevention
**Phase**	III
**Date of first enrolment**	14 November 2019
**Target sample size**	500,000
**Recruitment status**	Complete

For peer review only - http://bmjopen.bmj.com/site/about/guidelines.xhtml

**Primary outcome(s)**	To estimate the vaccine effectiveness of population-based vaccination delivery with the Ad26.ZEBOV, MVA-BN-Filo vaccine regimen for the prevention of EVD in adults and children aged 1 year or above. (Timeframe: From 2 weeks after intervention to end of study)
**Key secondary outcomes**	To assess safety of the Ad26.ZEBOV, MVA-BN-Filo vaccine (Timeframe: From dose 1 vaccination to 1 month post dose 2)<br>To estimate the coverage of dose 1 and dose 2 of the Ad26.ZEBOV, MVA-BN-Filo vaccine regimen overall and in different target groups (Timeframe: vaccination period)<br>To explore the knowledge and perceptions of persons eligible for large-scale delivery of a preventative Ebola vaccine with a two-dose vaccine strategy (Timeframe: vaccination period)
**Other outcomes**	To assess immunogenicity of the Ad26.ZEBOV, MVA-BN-Filo vaccine in a subset of participants who received dose 2 outside the recommended interval between dose 1 and dose 2.
**Ethics Review 1**	Approved by LSHTM Ethics Committee<br>Date of approval: 15 Oct 2019<br>Name and contact details: Rebecca Carter<br>ethics@lshtm.ac.uk
**Ethics Review 2**	Approved by Médecins Sans Frontières Ethics Review Board<br>Date of approval: 15 Octo 2019<br>Name and contact details: Raffaella Ravinetto<br>raffaella.ravinetto@gmail.com
**Ethics Review 3**	Approved by Comité d'éthique de l'école de santé publique, Université de Kinshasa<br>Date of approval: 22 Oct 2019<br>Name and contact details: Willy Bongopasi<br>bongopasi@gmail.com
**Ethics Review 4**	Approved by Comité national d'éthique et de la santé du Ministère de la Santé Publique de la République Démocratique du Congo (RDC)<br>Date of approval: 23 Oct 2019<br>Name and contact details: Félicien Munday<br>feli1munday@yahoo.fr

For peer review only - http://bmjopen.bmj.com/site/about/guidelines.xhtml

Table 2. Time and Event Schedule for Intervention Delivery and Safety Evaluations

**Preparation**	**Up to 1 month before study starts**	**D0 Dose 1 visit**	**D7 (-3/ + 7 day)**	**D21 (-6/ +7 day)**	**D56 Dose 2 visit (-14/ +28 day)**	**D7 post-dose 2 (-3/ +7 day)**	**D21 post-dose 2 (-3/ +7 day)**	**1 month post-dose 2 (-7 day/+1 month)**	**3 months post-dose 2 (+/-14 day)**	**6 months post-dose 2 (-14/ +28 day)**	**3 months post-delivery**
Mapping & selection of study sites	X										
Community engagement & Social Mobilization	X										
Consent, Vaccination dose 1		X									
Vaccination dose 2					X[2]						
SAE assessment					X[1]			X[3]			
Safety subset					X			X[4]			
All pregnant women					X[9]			X			X[6]
Pregnancy subset		X	X[5]	X[5]	X	X[5]		X[5]	X[5]	X[5]	X[7]
Immuno-genicity subset					X[8]		X[8]				

[1] D56 safety data are collected at dose 2 visits.
[2] Dose 2 may be given between D42 and D84. Participants who return for dose 2 after 84 days will still be vaccinated.
[3] Passive SAE recording from main study participants through participant-initiated phone calls or other contact with team, until 1 month post-dose 2.
[4] Safety subset: 1000 participants will be actively followed up for SAEs by phone at 1 month post-dose 2.
[5] Pregnancy subset: safety telephone calls to collect SAEs at D7 and D21 post-dose 1, and then at D7, 1 month, 3 months and 6 months post-dose 2, unless delivery is before these time points.
[6] All pregnant women will have their birth outcomes collected around 3 months post-delivery (between 1 to 3 months). When feasible, team to visit if the woman is not contactable by telephone.
[7] 100 infants born to women in the pregnancy subset will be given a full clinical examination around 3 months after delivery (between 1 to 6 months).
[8] Immunogenicity subset: A subset of paediatric and adult participants will have a venous blood draw at the dose 2 visit before vaccination. A second blood sample will be taken 21 days (-3/+7 day) after their dose 2 vaccination. Enrolment of this subset will continue until at least 50 adults and 50 children have had blood samples collected for immunogenicity at dose 2 and 21 days post-dose 2.
[9] If study vaccinations have been paused, all pregnant participants who have not received dose 2 will be called at D56 (+/-7 days) to collect safety data.

For peer review only

For peer review only - http://bmjopen.bmj.com/site/about/guidelines.xhtml

Box 1 DRC-EB-001 inclusion and exclusion criteria for vaccination

Inclusion criteria

1. Must provide a written or witnessed (if illiterate) informed consent form indicating that he or she understands the reasons for the study and is willing to participate in the study and to be vaccinated. If less than 18 years old, must have a parent or guardian that is able to meet this criterion.
2. Must be aged 1 year or older.
3. Must be healthy in the investigator's clinical judgment as assessed on the day of vaccination.
4. Must be willing to have a photograph taken.
5. Must be available and willing to participate for duration of study visits and follow up

Exclusion criteria

1. Known history of EVD.
2. Has received any experimental EVD vaccine less than one month prior to visit 1.
3. Known allergy or history of anaphylaxis or other serious adverse reactions to vaccines or vaccine products, egg and egg proteins or gentamicin.
4. Presence of an acute illness (excluding minor illnesses such as mild diarrhea or mild upper respiratory tract infection) or temperature ≥38.0ºC at visit 1 (dose 1 visit). Participants with such symptoms will be temporarily excluded from vaccination at that time but may be rescheduled for vaccination at a later date if feasible (and if within 84 days of the first dose).
5. Presence of significant conditions or clinically significant findings at the vaccination visit for which, in the opinion of the investigator, vaccination would not be in the best interest of the participant.
6. History of recurrent generalized hives

Note: Participants who have received treatment for acute, uncomplicated malaria are eligible for vaccination if at least 3 days have elapsed from the conclusion of a standard, recommended course of therapy for malaria

Rescheduling for dose 1 or dose 2 is possible in case of acute illness at the time of planned vaccination as long as rescheduling for dose 2 is within 84 days of dose 1.

For peer review only

For peer review only - http://bmjopen.bmj.com/site/about/guidelines.xhtml

Table 3. Sample sizes for a test-negative case-control study design, assuming 80% power to detect an OR = 1-VE, by percent coverage amongst controls (those testing negative) and number of controls per case, with a two-sided alpha=0.05 test.

Vaccine efficacy (VE)	Coverage (% vaccinated amongst controls)	Sample size (no. of cases) by number of controls per case			
		1	2	3	
90%	5%	210	171	157	
	10%	103	84	77	
	20%	50	40	37	
	30%	32	26	23	
	50%	19	15	13	
	70%	14	11	9	
70%	5%	418	333	303	
	10%	211	167	152	
	20%	107	84	76	
	30%	74	57	52	
	50%	50	38	34	
	70%	46	34	30	
50%	5%	962	749	677	
	10%	492	383	345	
	20%	260	201	181	
	30%	186	143	128	
	50%	137	103	92	
	70%	141	105	92	
30%	5%	3070	2350	2109	
	10%	1593	1218	1092	
	20%	866	659	590	
	30%	638	483	432	
	50%	499	375	334	
	70%	553	412	364	

For peer review only

For peer review only - http://bmjopen.bmj.com/site/about/guidelines.xhtml

References

1. Blumberg L ED, Bausch D G. Viral Haemorrhagic Fevers. Oxford: Elsevier Expert Consult; 2014.
2. Leroy EM, Kumulungui B, Pourrut X et al. Fruit bats as reservoirs of Ebola virus. Nature. 2005;438(7068):575-6.
3. Letko M, Seifert SN, Olival KJ et al. Bat-borne virus diversity, spillover and emergence. Nat Rev Microbiol. 2020;18(8):461-71.
4. Goldstein T, Anthony SJ, Gbakima A et al. The discovery of Bombali virus adds further support for bats as hosts of ebolaviruses. Nat Microbiol. 2018;3(10):1084-9.
5. De Nys HM, Kingebeni PM, Keita AK et al. Survey of Ebola Viruses in Frugivorous and Insectivorous Bats in Guinea, Cameroon, and the Democratic Republic of the Congo, 2015-2017. Emerg Infect Dis. 2018;24(12):2228-40.
6. Leroy EM, Epelboin A, Mondonge V et al. Human Ebola outbreak resulting from direct exposure to fruit bats in Luebo, Democratic Republic of Congo, 2007. Vector Borne Zoonotic Dis. 2009;9(6):723-8.
7. Jacob ST, Crozier I, Fischer WA, et al. Ebola virus disease. Nat Rev Dis Primers. 2020;6(1):13. DOI: 10.1038/s41572-020-0147-3
8. Henao-Restrepo AM, Camacho A, Longini IM et al. Efficacy and effectiveness of an rVSV-vectored vaccine in preventing Ebola virus disease: final results from the Guinea ring vaccination, open-label, cluster-randomised trial (Ebola ça Suffit!). Lancet. 2017;389(10068):505-18.
9. World Health Organization SAGE Ebola Virus Disease, Meeting of the Strategic Advisory Group of Experts on Immunization, October 2018 - Conclusions and recommendations. Wkly Epidemiol Rec. 2018 49, 2018, 93, 661-680.
10. World Health Organization. News. Four countries in the African region license vaccine in milestone for Ebola prevention. 14 Feb 2020. Available at: https://www.who.int/news/item/14-02-2020-four-countries-in-the-african-region-license-vaccine-in-milestone-for-ebola-prevention (accessed 6 Mar 2021).
11. World Health Organization. Ebola Virus Disease - Democratic Republic of the Congo External Situation Report 1. World Health Organization; 7 August 2018. https://reliefweb.int/sites/reliefweb.int/files/resources/SITREP-EVD-DRC-20180511.pdf (accessed 8 May 2021).
12. MONUSCO. United Nations Organization Stabilization Mission in the DR Congo. 2019. https://monusco.unmissions.org/en (accessed 5 May 2021).
13. World Health Organization R&D Blueprint. Ebola vaccine candidates. World Health Organization; 12 April 2019. https://www.who.int/blueprint/priority-diseases/key-action/ebola-vaccine-candidates/en/ (accessed 7 Mar 2021).
14. Milligan ID, Gibani MM, Sewell R et al. Safety and Immunogenicity of Novel Adenovirus Type 26- and Modified Vaccinia Ankara-Vectored Ebola Vaccines: A Randomized Clinical Trial. JAMA 2016;315(15):1610-23.
15. Mutua G, Anzala O, Luhn K et al. Safety and Immunogenicity of a 2-Dose Heterologous Vaccine Regimen With Ad26.ZEBOV and MVA-BN-Filo Ebola Vaccines: 12-Month Data From a Phase 1 Randomized Clinical Trial in Nairobi, Kenya. J Infect Dis. 2019;220(1):57-67.
16. Pollard AJ, Launay O, Lelievre JD et al. Safety and immunogenicity of a two-dose heterologous Ad26.ZEBOV and MVA-BN-Filo Ebola vaccine regimen in adults in Europe

For peer review only - http://bmjopen.bmj.com/site/about/guidelines.xhtml

(EBOVAC2): a randomised, observer-blind, participant-blind, placebo-controlled, phase 2 trial. Lancet Infect Dis. 2020;21(4):493-506.
17. Rudge TL, Jr., Sankovich KA, Niemuth NA et al. Development, qualification, and validation of the Filovirus Animal Nonclinical Group anti-Ebola virus glycoprotein immunoglobulin G enzyme-linked immunosorbent assay for human serum samples. PLoS One. 2019;14(4):e0215457.
18. Ishola D MD, Afolabi MO, Keshinro B et al. Safety and long-term immunogenicity of a two-dose Ad26.ZEBOV, MVA-BN-Filo Ebola vaccine regimen in adults: a randomised, double-blind, controlled trial in Sierra Leone. Lancet Infect Dis. 2021;In press.
19. Barry H MG, Kibuuka H, Anywaine Z et al. A randomized clinical trial of the safety and immunogenicity of a 2-dose heterologoour ebola vaccine regimen with Ad26.ZEBOV and MVA-BN-FILO® in healthy and HIV+ African Adults. American Society of Tropical Medicine and Hygiene, 68th Annual Meeting, National Harbor, MD, USA. 20-24 Nov 2019. Abstract number 1291.
20. Afolabi MO, Manno D, Keshinro B et al. Safety and immunogenicity of a two-dose Ad26. ZEBOV, MVA-BN-Filo Ebola vaccine in children: a randomised, double-blind, controlled trial in Sierra Leone. Lancet Infect Dis. 2021;In press.
21. Johnson & Johnson. Johnson & Johnson Announces European Commission Approval for Janssen's Preventive Ebola Vaccine. 1 Jul 2020. Available at: https://www.jnj.com/johnson-johnson-announces-european-commission-approval-for-janssens-preventive-ebola-vaccine (accessed 24 Jan 2021).
22. European Medicines Agency. New vaccine for prevention of Ebola virus disease recommended for approval in the European Union. 29 May 2020. Available at: https://www.ema.europa.eu/en/news/new-vaccine-prevention-ebola-virus-disease-recommended-approval-european-union (accessed 24 Jan 2021).
23. Dean NE, Gsell P-S, Brookmeyer R et al. Design of vaccine efficacy trials during public health emergencies. Sci Transl Med. 2019; 11(499) eaat0360.
24. World Health Organization R&D Blueprint. Design of vaccine efficacy trials to be used during public health emergencies – Points of considerations and key principles. 2018. https://www.who.int/docs/default-source/blue-print/working-group-for-vaccine-evaluation-(4th-consultation)/ap1-guidelines-online-consultation.pdf (accessed 4 May 2021).
25. World Health Organization Global Ebola Vaccine Implementation Team (GEVIT) Practical guidance on the use of Ebola vaccine in an outbreak response. World Health Organization. May 2016. https://www.who.int/csr/resources/publications/ebola/gevit_guidance_may2016.pdf (accessed 4 May 2021)
26. Verani JR, Baqui AH, Broome CV et al. Case-control vaccine effectiveness studies: Preparation, design, and enrollment of cases and controls. Vaccine. 2017;35(25):3295-302.
27. De Serres G, Skowronski DM, Wu XW et al. The test-negative design: validity, accuracy and precision of vaccine efficacy estimates compared to the gold standard of randomised placebo-controlled clinical trials. Euro Surveill. 2013;18(37). DOI: 10.2807/1560-7917.es2013.18.37.20585.
28. Marks M, Lai S, Brindle H et al. Electronic data management for vaccine trials in low resource settings: Upgrades, scalability and impact of ODK. MedRxiv 20210208 [Preprint]. February 10, 2021. https://www.doi.org/10.1101/2021.02.08.20191908 (accessed 4 May 2021).

For peer review only - http://bmjopen.bmj.com/site/about/guidelines.xhtml

Protocol for a phase 3 trial to evaluate effectiveness and safety of a heterologous, two-dose vaccine for Ebola virus disease in the Democratic Republic of the Congo

Appendix 1A

Informed Consent Form in English, Version 7.1 (10 Aug 2020)

For peer review only

For peer review only - http://bmjopen.bmj.com/site/about/guidelines.xhtml

Evaluation of a heterologous, two-dose preventive Ebola vaccine for effectiveness and safety in the Democratic Republic of the Congo

"The TUJIOKOWE Study" Information Sheet and Informed Consent Form

LSHTM Protocol: DRC-EB-001

Principal Investigator (PI):
Professor Jean Jacques Muyembe
Director-General
Institut National de Recherche Biomédicale
Professor of Microbiology, Kinshasa University Medical School
Kinshasa Gombe, Democratic Republic of the Congo (DRC)
Phone: 0898 949 289; Email: jjmuyembet@gmail.com

Sponsor: London School of Hygiene & Tropical Medicine, United Kingdom

Site: Democratic Republic of the Congo

For peer review only

INTRODUCTION

You are being invited to take part in a research study called the 'TUJIOKOWE study' to find out if a new two-dose vaccine called the 'Janssen Ebola vaccine' can protect people from getting Ebola and to check whether the vaccine is safe. The TUJIOKOWE study is being implemented by the Ministry of Health of DRC through the Institut National de Recherche Biomédicale (INRB), Epicentre and the London School of Hygiene & Tropical Medicine (LSHTM).

WHAT IS EBOLA?

Ebola is a disease named after the Ebola virus. When a person gets Ebola, they can become very sick, and about half of the people who catch Ebola die. Once a person has Ebola, the disease can be spread to other people, especially if people are living in the same house.

WHAT IS A VACCINE AND WHAT IS A CLINICAL RESEARCH STUDY?

Vaccines are medical products that are used to stop people from having certain diseases. Many people get vaccines to prevent them catching diseases like measles, polio, and cholera, for example.

A clinical research study helps health professionals understand new ways to prevent or treat a disease. In this study, the vaccine is still under testing. Participants in research studies about vaccines are followed over time so that the researchers can learn more about the vaccine.

For peer review only - http://bmjopen.bmj.com/site/about/guidelines.xhtml

ARE ANY OTHER VACCINES BEING GIVEN TO PREVENT EBOLA IN DRC?

Vaccination is occurring with a vaccine called "VSV" in several places like Beni, Katwa and Butembo. This vaccine is only being given to people who are at risk of catching Ebola, such as people who have come into contact with someone who has Ebola, or who have come into contact with people close to someone with Ebola. It is also being given to community health workers and other workers who may met Ebola. However, about half of the people who become infected with Ebola in DRC are not known to have had contact with an Ebola case, so we need to protect them too. Therefore, we are looking for additional ways to protect people from Ebola, including this vaccine.

WHAT IS THIS STUDY ABOUT?

This study is going to find out if a new vaccine can help protect people against Ebola. This new vaccine is given in two injections. The first injection is a dose of a vaccine called Ad26.ZEBOV. A second injection is given about two months later, with a second dose called MVA-BN-Filo. In this study, we will call these two injections with two different vaccines the 'Janssen Ebola vaccine'.

In the rest of this document, we will talk about you. The word 'you' can mean you or your child. If you are the parent or guardian of a child under 18 years of age and you would like your child to join this study, you will need to sign separate documents for you and your child.

If your child is aged 12 to 17 years old, he/she will also need to agree to join the study himself/herself and sign a different document, called the assent form.

WHAT DO WE KNOW ABOUT THE VACCINE?

The Janssen Ebola vaccine is designed to protect people against Ebola. **It cannot make you sick with Ebola.** Over 6000 people have received the vaccine in many different countries, including 7 in Africa. So far, the vaccine is safe and well-tolerated in people who received it. The vaccine may help the body to fight Ebola infections, but we don't know for sure yet if the Janssen Ebola vaccine can protect people against Ebola.

WHO CAN TAKE PART IN THIS STUDY?

To be in this study, you must be living in or working in one of the communities that will be offered the Janssen Ebola vaccine. You must be able to return to get the second dose of vaccine and be willing to follow the requirements needed to take part in this study. Participants should be well in order to get each dose of the vaccine. People diagnosed with HIV (who are well), pregnant and breastfeeding women, and children aged 1 year or older can be included in this study.

You cannot be in this study if you are an Ebola survivor. You also cannot be in this study if you are allergic to eggs or egg products.

For peer review only - http://bmjopen.bmj.com/site/about/guidelines.xhtml

If you have received another Ebola vaccine or routine vaccine within the last month (30 days) or if you have a fever of 38°C or higher on the vaccination day, you may be asked to come back another day. You may receive the vaccine if you have a mild cold, cough or mild diarrhea.

DO I HAVE TO BE IN THIS STUDY?

You do not have to join this study if you don't want to. If you agree to be in the study, we will ask you to sign this consent form. If you decide that you don't want to be in the study, then this will not affect you in any way. If you don't want to be in the study, but you change your mind later, you will have another chance to join the study. After the second visit by the team to your community, it may not be possible to join this study to get the Janssen Ebola vaccine. You must be in the study to receive the vaccine.

WHAT WILL HAPPEN IF I AGREE TO TAKE PART IN THIS STUDY?

We will describe the study and answer any questions that you may have. If you would like a copy of the written information, we will give it to you. You will be asked some questions about the study by one of the study staff members to check that you have understood the information. You will be asked to sign or put your fingerprint on this informed consent form. Putting your name or your fingerprint on the consent form means that you agree to be in the study, but you can change your mind and choose to leave at any time.

WHAT DOES THE STUDY INVOLVE?

After you sign this consent form, you will be asked some questions to check how you are feeling. If the doctor decides that you can be vaccinated, you will get an injection of the first Janssen Ebola vaccine into your upper arm, or the thigh for younger children. We will give you a vaccination card with a phone number of who to contact in case you need it. The card will also show the date of when you need to come back for the second Janssen Ebola vaccine. We will ask you for a phone number and we may call you for the second vaccine and follow ups.

In order to identify you at the next visit, we will take photographs of your face. We do this to make sure that we know who has been vaccinated and ensure you receive the second dose at the right time.

You will be asked to attend on a specific date for the second Janssen Ebola vaccine. After the second dose, we will ask you to call us if you have any medical problems in the 30 days after your second vaccine dose. You should report any sickness to the study team in those 30 days by calling the phone number on your vaccination card. We will ensure that there are telephones available in your community.

A certain number of participants will be included in a group for follow up who will be contacted by phone or a home visit.

As a result of the study pause due to COVID-19, some participants had their second vaccination with the Janssen Ebola vaccine postponed. These participants will be called to receive their

For peer review only - http://bmjopen.bmj.com/site/about/guidelines.xhtml

second vaccination at a later date. Receiving the second Janssen Ebola vaccine at a later date will not have negative effects on the health of these participants.

WHAT ARE THE POSSIBLE RISKS OF BEING IN THIS STUDY?

We do not know for sure yet if the Janssen Ebola vaccine protects people from Ebola, so there is a risk that you will not be protected from Ebola even after you have taken the vaccine.

This vaccine has been given to more than 6000 people in other research studies. Some of the side effects that you may have after the injections are:

- pain, swelling, itchiness, redness, or warmth around the place where you got the injection.
- in the area around the injection it may be sore and a bit painful to move for a few days.
- you may notice that you have swollen bumps under your arms or in your neck for a few days.
- you may get a fever or headache, feel tired, or have chills, rash, nausea, change in appetite, vomiting, diarrhea, dizziness, muscle, joint or belly aches, mouth sores, or body pains.

In the previous research studies, not everyone has gotten these side effects after the injections. Side effects are usually mild and most only last a few days. In a few people they may last for a few weeks. Some people have reported that they felt some tingling in the hands and feet, or their muscles felt weak. These symptoms usually only lasted one or two days, but sometimes for several weeks. One person reported tingling, numbness and pain which lasted for several months and interfered with the person's daily activities.

We have learnt that 3 women living in other countries who were less than 40 years old had serious blood clots within 3 weeks of receiving a vaccine that was made in a similar way to the second Janssen Ebola vaccine. One of them had a stroke and died. A 50-year-old man also reported lack of blood flow to the leg. These cases also had medical conditions that are known to increase the risk of blood clots and none of these events was considered related to vaccine. Receiving the vaccine did not increase a person's chance of experiencing these problems.

Hundreds of babies (aged 1-3 years), children (aged 4-11 years) and adolescents (aged 12-17 years) have received the Janssen Ebola vaccine so far. The reported side effects in children and adolescents after vaccination were similar to those reported by adults. Injection site pain was the most frequently reported local side effect. Headache, fatigue and chills were also frequently reported in older children and adolescents. Decreased appetite, decreased activity and fever were the most frequently reported side effects in babies. Following vaccination, a few children experienced a fever above 39°C. Most of these side effects were mild to moderate and did not last more than 1 or 2 days. No serious medical problems have been seen in children who received the vaccine. The side effects in children are similar to those that happen with other vaccines in children.

The medical team will provide care if you experience side-effects from the injections up until a month after the second dose. They are available if you have any problems or questions.

For peer review only - http://bmjopen.bmj.com/site/about/guidelines.xhtml

WHAT ARE THE BENEFITS OF BEING IN THIS STUDY?

On the days of vaccination, a doctor will question you to check if you have any symptoms and, if necessary, you may receive free treatment or a referral for simple medical conditions like malaria. You will also learn how to protect yourself against Ebola. Your participation in this study will help in the development of vaccines to prevent Ebola and, in the future, may help people in different parts of the world.

WHAT ABOUT PREGNANCY AND/OR BREASTFEEDING DURING THE STUDY?

You can participate in this study if you are pregnant or breastfeeding. We do not know all the effects of the study vaccines in pregnancy or in a baby that is being breastfed. If you know you are pregnant or breastfeeding at the time of vaccination, please tell the study staff. If you are not sure, we will offer you a pregnancy test and medical interview and will let you know if you are pregnant before each vaccination. If you are pregnant or become pregnant within one month of each vaccination, we will contact you during your pregnancy to ask you questions about your health and your baby's health. We will also contact you within 3 months after the delivery of your baby. If we cannot contact you by telephone after your baby is born, the study team may visit you at your home. Among the pregnant women in the study, a certain number will be invited to join a pregnancy subset and will be followed up by telephone or through home visits more frequently and some babies of women in the pregnancy subset will also be seen within 3 months after birth. If you become pregnant within one month after vaccination, please contact the phone number on your vaccination card and let the study team know.

WHAT DO I NEED TO DO FOR MY OWN HEALTH DURING THIS STUDY?

If you become sick during the study, you should go to one of the recommended health facilities that are on the list that we will give you. You should also call the study contact phone number on your vaccination card and tell us if you are having any medical problems up until 30 days after you received the second dose of the vaccine. You may be referred for medical care if necessary.

We do not know for sure yet if the Janssen Ebola vaccine can protect people against Ebola. If you get the vaccine, you may be protected from Ebola, but it is possible that you may not be protected. **Therefore, you must continue to protect yourself from contact with Ebola.** We will give you specific information on how to prevent Ebola, and you should always contact a member of the study team if you have any questions about how to protect yourself from contact with Ebola. If you experience anything that seems like the symptoms of Ebola, such as fever, diarrhea, vomiting, or unexplained bleeding, it is very important that you let the study team know that you are sick and that you seek medical care right away.

If we find out anything new during this study that may be related to your health or affect your decision to continue in the study, we will talk to you about it.

For peer review only - http://bmjopen.bmj.com/site/about/guidelines.xhtml

WHO WILL BE ABLE TO SEE MY INFORMATION?

We will keep your study information private.

Staff working on the study, including people from overseas who are responsible for checking that this study is done properly, ethics committees, and regulatory authorities, may look at your medical records. All study staff are trained in confidentiality and will not remove any document with your name on it from the study files. All of these people understand that they must keep your identity private and they won't share your name or contact details with anyone.

A description of this study will be available on the internet at http://www.ClinicalTrials.gov. No information that can identify you will be on this website. You can look at this website at any time.

The study results will be shared, but no personal information will be included in the published study results. If you have any specific questions about your data, please contact the study Principal Investigator, Professor Jean Jacques Muyembe or his representative (see contact details below).

WHO IS CHECKING THAT THE STUDY IS SAFE?

This study is being implemented by partners including the Ministry of Health of DRC through the Institut National de Recherche Biomédicale (INRB), Epicentre, and the London School of Hygiene & Tropical Medicine (LSHTM). An independent committee made up of doctors and experts who are not directly involved in the study will be looking at the study information and checking on the safety of the vaccines.

For peer review only

For peer review only - http://bmjopen.bmj.com/site/about/guidelines.xhtml

WHO CAN I TALK TO ABOUT THIS STUDY?

If you want to talk to someone about this study or if you think you have been harmed by being part of the study, you can contact the following.

1) The Principal Investigator responsible for this study

Professor Jean Jacques Muyembe<br>*Director General,* Institut National de Recherche Biomédicale, Kinshasa, DRC<br>Phone: 0898 949 289<br>Email: jjmuyembet@gmail.com<br><br>Local study representative<br>Dr Hugo Kavunga<br>Phone: 0823 875 153<br>Email: hugokavunga@gmail.com

2) The DRC ethics committees that approved this study

Professor Félicien Munday<br>National Ethics Committee<br>Kinshasa-Gombe, DRC<br>Phone: 0998 419 816<br>Email: feli1munday@yahoo.fr
Professeur Willy Bongopasi<br>Comité d'éthique de l'école de santé publique<br>Université de Kinshasa<br>Téléphone: 0999 952 341<br>Email: bongopasi@gmail.com

If you have any questions about the TUJIOKOWE study or about your rights as a study participant, you may ask anyone on the study team at any time.

For peer review only - http://bmjopen.bmj.com/site/about/guidelines.xhtml

PARTICIPANT OR PARENT/GUARDIAN CONSENT FORM

Title: TUJIOKOWE Study

Principal Investigator of this study: Prof. JJ Muyembe

**Statements**	**Please sign or fingerprint each box**
I have read the information in this form about the TUJIOKOWE Study (or I have had this information explained to me by the study staff in a language that I understand). The purpose of this study and the procedures to receive this new vaccine have been fully explained to me. I was able to ask questions and have all of these questions answered to my satisfaction.	
I understand that my/my child's participation is voluntary, that I/my child can withdraw consent and leave this study at any time without giving any reason, and that this will not affect my/my child's medical care or legal rights.	
I have been informed that the new Janssen Ebola vaccine is still being tested, and that not all the possible risks from having this vaccine are completely known.	
I confirm that I/my child have **NOT** previously had Ebola or been vaccinated against Ebola within the past 30 days.	
I agree to have my/my child's photograph taken by the study team before vaccination.	
I agree to give the study team a contact phone number if I have one and to be contacted for the second vaccine and for follow up calls or SMS e.g. for health questions. If I don't have a phone, I agree to try and provide a relative or friend's number that can be used to contact me.	
I understand that the information collected about me/my child will be used to support other research in the future and may be shared anonymously with other researchers.	
I agree that my/my child's medical information that will not contain my/my child's name, can be shared with the national health authorities, the vaccine manufacturer (Janssen Vaccines & Prevention B.V.) and other foreign organizations outside the DRC, such as the US Food & Drug Administration. I give permission for individuals in these organizations to access and analyse my/my child's records and publish them in scientific journals.	

For peer review only - http://bmjopen.bmj.com/site/about/guidelines.xhtml

PARTICIPANT OR PARENT/GUARDIAN CONSENT FORM

Declarations Regarding Optional Procedures

**Statements**	**Please sign or fingerprint each box for yes**	**Please sign or fingerprint each box for no**
(For female participants only) I agree that, if I/my child am pregnant at the time of vaccination or become pregnant during the first month after receiving each vaccine, and I/my child am selected by the study team to participate in the pregnancy subset, the study team will contact me by telephone during my/my child's pregnancy more often than other pregnant women in the study and after the birth of my/my child's baby a member of the study team may see me/my child at home if I cannot be contacted by telephone.		
I agree that if I/ my child am selected by the study team to be in the safety group, the study team may contact me by telephone after my second vaccination and may see me/ my child at home if I cannot be contacted by telephone.		

FOR ADULT PARTICIPANT

I am aged 18 years or older and I agree to take part in this study. (Please sign or put your fingerprint below)

		____/____/_____<br>*dd mon yyyy*
Printed name of adult participant	Signature/fingerprint of adult participant	Date

FOR PARENT OR GUARDIAN

I am the parent or guardian of a participant aged 1 to 17 years and I agree for my child to take part in this study. (Please sign or put your fingerprint below)

		____/____/_____<br>*dd mon yyyy*
Printed name of the parent or guardian	Signature/fingerprint of the parent or guardian	Date

For peer review only - http://bmjopen.bmj.com/site/about/guidelines.xhtml

FOR PARENT OR GUARDIAN

Printed name of the participant (if aged 1-17)

		____/____/_____ *dd mon yyyy*
Printed name of investigator	Signature of investigator	Date

Complete next section if participant or parent/guardian is illiterate:

Witness to Consent Interview

I witnessed the consent interview for the TUJIOKOWE Study in this document. I attest that I have explained the study information accurately to the participant or parent/guardian and was understood to the best of my knowledge by the participant or parent/guardian, and that he/she has freely given their consent to participate/for his/her child to participate in my presence.

		____/____/_____ *dd mon yyyy*
Printed name of impartial witness	Signature of impartial witness	Date

---

Attach ID barcode label below:

For peer review only - http://bmjopen.bmj.com/site/about/guidelines.xhtml

Protocol for a phase 3 trial to evaluate effectiveness and safety of a heterologous, two-dose vaccine for Ebola virus disease in the Democratic Republic of the Congo

Appendix 1B

Informed Consent Form in French, Version 7.1 (10 Aug 2020)

For peer review only

For peer review only - http://bmjopen.bmj.com/site/about/guidelines.xhtml

Évaluation de l'efficacité et de l'innocuité de l'emploi d'un vaccin préventif hétérologue à deux doses contre Ebola en République démocratique du Congo

Note d'information et formulaire de consentement éclairé de l'« étude TUJIOKOWE »

Protocole LSHTM : DRC-EB-001

Investigateur principal (IP) :
Professeur Jean Jacques Muyembe
Directeur général
Institut national de recherche biomédicale
Professeur de microbiologie, Faculté de médecine de l'Université de Kinshasa
Kinshasa Gombe, République démocratique du Congo (RDC)
Téléphone : 0898 949 289 ; E-mail : jjmuyembet@gmail.com

Promoteur : London School of Hygiene & Tropical Medicine, Royaume-Uni

Site : République démocratique du Congo

INTRODUCTION

Vous êtes invité(e) à participer à une étude de recherche appelée l'« étude TUJIOKOWE » pour savoir si un nouveau vaccin à deux doses appelé « vaccin Janssen Ebola » peut protéger contre le virus Ebola et pour vérifier si le vaccin est sans danger. L'étude TUJIOKOWE est mise en œuvre par le Ministère de la Santé de la RDC par l'intermédiaire de l'Institut national de recherche biomédicale (INRB), Epicentre et la London School of Hygiene & Tropical Medicine (LSHTM).

QU'EST-CE QUE LE VIRUS EBOLA ?

Ebola est une maladie dont le nom vient du virus Ebola. Lorsqu'une personne contracte le virus Ebola, elle peut parfois tomber très malade, et environ la moitié des personnes qui contractent le virus Ebola meurent. Une fois qu'une personne a contracté le virus Ebola, il peut se propager à d'autres personnes, en particulier si elles vivent sous le même toit.

QU'EST-CE QU'UN VACCIN ET QU'EST-CE QU'UNE ÉTUDE DE RECHERCHE CLINIQUE ?

Les vaccins sont des produits médicaux utilisés pour empêcher d'avoir certaines maladies. De nombreuses personnes sont vaccinées contre la rougeole, la polio et le choléra, par exemple.

Une étude de recherche clinique permet aux personnels de santé de comprendre de nouvelles façons de prévenir ou de traiter une maladie. Dans cette étude, le vaccin est encore sous test.

For peer review only - http://bmjopen.bmj.com/site/about/guidelines.xhtml

Les participants à des études de recherche sur les vaccins sont suivis pendant un certain temps afin de recueillir plus d'informations sur le vaccin.

D'AUTRES VACCINS SONT-ILS ADMINISTRÉS POUR PRÉVENIR LE VIRUS EBOLA EN RDC ?

Des vaccinations sont en cours avec un vaccin appelé « VSV » dans plusieurs endroits comme Beni, Katwa et Butembo. Ce vaccin est administré à des personnes à risque de contracter le virus Ebola, comme celles en contact avec une personne infectée par le virus Ebola ou avec des personnes en contact avec celle-ci. Il est également administré à des professionnels de la santé et d'autres travailleurs qui peuvent entrer en contact avec le virus Ebola. Cependant, environ la moitié des personnes infectées par le virus Ebola en RDC ne sont pas connues pour avoir été en contact avec un cas d'Ebola ; nous devons donc les protéger également. Par conséquent, nous cherchons d'autres moyens de protéger les gens contre Ebola, y compris ce vaccin.

EN QUOI CONSISTE CETTE ÉTUDE ?

Cette étude va déterminer si un autre vaccin peut aider à protéger la population contre Ebola. Ce nouveau vaccin est administré en deux injections. La première injection est une dose d'un vaccin appelé Ad26.ZEBOV. La seconde injection est administrée environ deux mois plus tard, avec une deuxième dose appelé MVA-BN-Filo. Dans cette étude, le vaccin « Janssen Ebola » désignera ces deux injections de deux vaccins différents.

Dans le reste de ce document, nous allons parler de vous. Le mot « vous » peut se rapporter à vous ou à votre enfant. Si vous êtes le parent ou le gardien d'un enfant de moins de 18 ans et si vous souhaitez que votre enfant participe à cette étude, vous devrez signer des documents distincts pour vous et votre enfant.

Si votre enfant est âgé de 12 à 17 ans, il/elle devra également accepter de participer à l'étude lui-même/elle-même et signer un autre document, appelé formulaire d'assentiment.

QUE SAVONS-NOUS DU VACCIN ?

Le vaccin Janssen Ebola est conçu pour protéger la population contre Ebola. **Vous ne pouvez pas contracter le virus Ebola par le vaccin.** Plus de 6 000 personnes ont reçu le vaccin dans de nombreux pays, dont 7 en Afrique. Jusqu'à présent, le vaccin est sûr et bien toléré chez les personnes vaccinées. Le vaccin pourrait aider le corps à lutter contre les infections à virus Ebola, mais nous ne savons pas encore avec certitude si le vaccin Janssen Ebola peut protéger la population contre Ebola.

QUI PEUT PARTICIPER À CETTE ÉTUDE ?

Pour participer à cette étude, vous devez vivre ou travailler au sein de l'une des communautés qui recevront le vaccin Janssen Ebola. Vous devez être en mesure de revenir pour recevoir la deuxième dose de vaccin et être disposé(e) à respecter les exigences nécessaires pour prendre part à cette étude. Les participants doivent être en bonne santé afin de recevoir chaque dose du

For peer review only - http://bmjopen.bmj.com/site/about/guidelines.xhtml

vaccin. Les personnes avec un diagnostic de VIH (qui sont en bonne santé), les femmes enceintes et allaitantes et les enfants âgés de 1 an ou plus peuvent être inclus dans cette étude.

Vous ne pouvez pas participer à cette étude si vous avez contracté la MVEbola.Vous ne pouvez pas non plus participer à cette étude si vous êtes allergique aux œufs ou aux produits contenant des œufs. Si vous avez été déjà vacciné contre la maladie à virus Ebola ou un autre vaccin de routine durant les 30 derniers jours ou si vous avez une fièvre de 38 °C ou plus le jour de la vaccination, vous devrez revenir un autre jour. Vous pouvez recevoir le vaccin si vous avez un léger rhume, de la toux ou une diarrhée légère.

SUIS-JE OBLIGÉ(E) DE PARTICIPER À CETTE ÉTUDE ?

Vous n'êtes pas obligé(e) de participer à cette étude si vous ne le souhaitez pas. Si vous décidez que vous ne souhaitez pas participer à l'étude, cela ne vous affectera en aucune façon. Si vous ne souhaitez pas participer à l'étude, mais que vous changez d'avis plus tard, vous pouvez participer à l'étude lorsque l'équipe de vaccination revient dans votre communauté. Après cette deuxième visite au sein de votre communauté, il peut ne plus être possible de participer à l'étude pour recevoir le vaccin Janssen Ebola. Vous devez être inclus dans l'étude pour recevoir le vaccin.

QUE SE PASSERA-T-IL SI J'ACCEPTE DE PARTICIPER À CETTE ÉTUDE ?

Nous allons décrire l'étude et répondre à toutes les questions que vous pourriez avoir. Si vous souhaitez une copie de cette note d'information, nous vous la donnerons. L'un des membres du personnel de l'étude vous posera des questions au sujet de l'étude pour vérifier que vous avez compris l'information. Nous vous demanderons de signer ou d'apposer votre empreinte digitale sur ce formulaire de consentement éclairé. En mettant votre nom ou en apposant votre empreinte digitale sur le formulaire de consentement, vous acceptez de participer à l'étude, mais vous pouvez changer d'avis et décider de quitter l'étude à tout moment.

QU'IMPLIQUE L'ÉTUDE ?

Après avoir signé ce formulaire de consentement, on vous posera quelques questions pour vérifier comment vous vous sentez. Si le médecin décide que vous pouvez être vacciné(e), vous recevrez une injection du premier vaccin Janssen Ebola contre Ebola dans la partie supérieure de votre bras ou dans la cuisse chez les jeunes enfants. Nous vous donnerons une carte de vaccination avec le numéro de téléphone de personnes à contacter en cas de besoin. La carte indiquera également la date à laquelle vous devrez revenir pour la deuxième dose de vaccin Janssen Ebola. Votre numéro de téléphone vous sera demandé, il est possible que vous soyez contacté afin de vous rappeler le rendez-vous de l'administration du second vaccin et également pour assurer un suivi.

Afin de vous identifier lors de la visite suivante, nous allons prendre une photographie de votre visage. Nous le faisons afin de savoir qui a été vacciné et veiller que vous receviez la deuxième dose au bon moment.

Vous serez invité(e) à vous présenter à une date spécifique pour le second vaccin Janssen Ebola. Après la deuxième dose, nous vous demanderons de nous appeler si vous avez des problèmes

For peer review only - http://bmjopen.bmj.com/site/about/guidelines.xhtml

médicaux dans les 30 jours après votre deuxième dose de vaccin. Vous devrez signaler toute maladie à l'équipe de l'étude dans les 30 jours en contactant le numéro de téléphone indiqué dans votre carte de vaccination. Nous veillerons à ce que des téléphones soient disponibles au sein de votre communauté.

Un certain nombre de participants seront inclus dans un groupe de suivi rapproché qui sera fait par téléphone ou par visite à domicile.

Suite à la pause de l'étude due à la COVID-19, certains participants ont vu leur deuxième dose de vaccin Janssen Ebola reportée. Ces participants seront appelés à recevoir leur deuxième dose à une date ultérieure. Le fait de recevoir le second vaccin Janssen Ebola à une date ultérieure n'aura pas d'effets négatifs sur la santé de ces participants.

QUELS SONT LES ÉVENTUELS RISQUES LIÉS À LA PARTICIPATION À CETTE ÉTUDE ?

Nous ne savons pas encore avec certitude si le vaccin Janssen Ebola protège la population contre Ebola. Il existe donc un risque que vous ne soyez pas protégé(e) contre le virus Ebola, même après la vaccination.

Ce vaccin a été administré à plus de 6 000 personnes dans d'autres études de recherche. Certains des effets secondaires que vous pourriez avoir après les injections sont les suivants :

- Douleur, gonflement, démangeaison, rougeur ou sensation de chaleur autour de la zone où vous avez reçu l'injection.
- Autour du site de l'injection, vous pourriez ressentir de la douleur lors de vos mouvements pendant quelques jours.
- Vous pourriez remarquer des bosses gonflées sous vos bras ou dans votre nuque pendant quelques jours.
- Vous pourriez avoir de la fièvre ou des maux de tête, une sensation de fatigue, des frissons, une éruption cutanée, des nausées, une modification de l'appétit, des vomissements, de la diarrhée, des étourdissements, des douleurs musculaires, articulaires ou au ventre, des plaies dans la bouche ou des courbatures.

Dans les études de recherche précédentes, tout le monde n'a pas eu ces effets secondaires après les injections. Les effets secondaires sont généralement légers et la plupart ne durent que quelques jours. Chez certaines personnes, ils peuvent durer pendant quelques semaines, très rarement plus longtemps. Certaines personnes ont signalé qu'elles avaient des picotements dans les mains et les pieds, ou une faiblesse musculaire. Généralement, ces symptômes ne duraient qu'un jour ou deux, mais parfois ils perduraient pendant plusieurs semaines. Une personne a signalé des picotements, un engourdissement et une douleur qui ont duré pendant plusieurs mois et interféré sur ses activités quotidiennes.

Nous avons appris que trois femmes de moins de 40 ans vivant dans d'autres pays ont présenté de graves caillots sanguins dans les trois semaines suivant l'administration d'un vaccin fabriqué de manière similaire au deuxième vaccin Janssen Ebola. L'une d'entre elles a eu un accident vasculaire cérébral et est décédée. Un homme de 50 ans a également signalé un manque de flux sanguin dans la jambe. Ces cas présentaient également des conditions médicales connues pour augmenter le risque de caillots sanguins et aucun de ces événements

For peer review only - http://bmjopen.bmj.com/site/about/guidelines.xhtml

n'a été considéré comme lié au vaccin. Le fait de recevoir le vaccin n'augmentait pas les risques d'avoir ces problèmes.
Des centaines de bébés (âgés de 1 à 3 ans), d'enfants (âgés de 4 à 11 ans) et d'adolescents (âgés de 12 à 17 ans) ont reçu le vaccin Janssen Ebola jusqu'à présent. Les effets secondaires signalés chez les enfants et les adolescents après la vaccination étaient similaires à ceux signalés par les adultes. La douleur au point d'injection était l'effet secondaire local le plus fréquemment signalé. Des maux de tête, de la fatigue et des frissons ont également été fréquemment signalés chez les enfants et les adolescents plus âgés. Une diminution de l'appétit, une baisse d'activité et de la fièvre ont été les effets secondaires les plus fréquemment signalés chez les bébés. Après la vaccination, quelques enfants ont eu une fièvre supérieure à 39°C. La plupart de ces effets secondaires étaient légers à modérés et n'ont pas duré plus d'un ou deux jours. Aucun problème médical grave n'a été observé chez les enfants qui ont reçu le vaccin. Les effets secondaires chez les enfants sont similaires à ceux qui se produisent avec d'autres vaccins chez les enfants.

L'équipe médicale vous fournira fournir des soins si vous ressentez des effets secondaires liés aux injections jusqu'au un mois après la seconde dose. Elle est disponible à tout moment si vous avez des problèmes ou des questions.

QUELS SONT LES AVANTAGES LIÉS À LA PARTICIPATION À L'ÉTUDE ?

Les jours de vaccination, un médecin vous posera des questions pour vérifier votre état de santé ; si nécessaire, vous recevrez les soins nécessaires ou serez dirigé vers un spécialiste pour des affections médicales simples comme le paludisme. Vous apprendrez aussi comment vous protéger contre le virus Ebola. Votre participation à cette étude contribuera au développement de vaccins pour prévenir le virus Ebola et, à l'avenir, pourrait aider des personnes dans différentes régions du monde.

GROSSESSE ET L'ALLAITEMENT PENDANT L'ÉTUDE ?

Vous pouvez prendre part à cette étude si vous êtes enceinte ou si vous allaitez. Nous ne sommes pas sûr de tous les effets des vaccins de l'étude pendant la grossesse ou chez un bébé allaité. Si vous savez que vous êtes enceinte ou si vous allaitez au moment de la vaccination, informez-en le personnel de l'étude. Si vous n'êtes pas certaine, nous vous proposerons un test de grossesse et un entretien avec du personnel médical qui vous informera si vous etes emceinte avant chaque vaccination. Si vous êtes enceinte, ou si vous tombez enceinte dans le mois qui suit l'administration d'une des deux doses, vous serez contactee durant votre grossesse afin de vous poser des questions concernant votre sante et celle votre bebe. Dans ce cas, nous vous contacterons pendant votre grossesse pour vous poser des questions sur votre état de santé et celui de votre bébé. Nous vous contacterons également dans les 3 mois après votre accouchement. Si nous ne pouvons pas vous contacter par téléphone après votre accouchement, l'équipe de l'étude vous rendra visite à votre domicile. Parmi les femmes enceintes dans l'étude, certain nombre de participante seront invitée à faire partie d'un sous-groupe pour assurer un suivi plus rapproché par téléphone ou au domicile. Certains nouveaux-nés de maman faisant partie du sous-groupe seront également suivis dans les 3 mois suivant la naissance
Si vous tombez enceinte dans le mois qui suit une vaccination, merci d'appeler le numéro de téléphone indiqué sur votre carte de vaccination et de prévenir l'équipe de l'étude.

For peer review only - http://bmjopen.bmj.com/site/about/guidelines.xhtml

QUE DOIS-JE FAIRE POUR MA PROPRE SANTÉ PENDANT CETTE ÉTUDE ?

Si vous tombez malade pendant l'étude, vous devez vous rendre dans l'un des établissements de santé recommandés qui sont indiqués sur la liste que nous vous donnerons. . Vous devrez également appeler le numéro de téléphone de la personne de contact de l'étude indiquée sur votre carte de vaccination et lui dire si vous présentez un problème médical, jusqu'à 30 jours après l'administration de la deuxième dose du vaccin. Vous pourrez être orienté(e) vers des soins médicaux, si nécessaire.

Nous ne savons pas encore avec certitude si le vaccin Janssen Ebola peut protéger contre Ebola. Si vous recevez le vaccin, vous pourriez être protégé(e) contre le virus Ebola, mais il est possible que vous ne soyez pas protégé(e). **Par conséquent, vous devez continuer à éviter les contacts avec le virus Ebola.** Nous vous donnerons des informations spécifiques sur la prévention du virus Ebola, et n'hésitez pas à contacter un membre de l'équipe de l'étude si vous avez des questions sur la façon d'éviter les contacts avec le virus Ebola. Si vous présentez des symptômes qui ressemblent au virus Ebola, comme de la fièvre, de la diarrhée, des vomissements ou des saignements inexpliqués, il est très important que vous informiez l'équipe de l'étude que vous êtes malade et que vous receviez des soins médicaux dès que possible.

Si nous découvrons quelque chose de nouveau pendant cette étude qui pourrait être lié à votre santé ou influencer votre décision de poursuivre l'étude, nous vous en parlerons.

QUI POURRA CONSULTER MES INFORMATIONS ?

Nous préserverons la confidentialité de vos informations de l'étude.
Le personnel de l'étude, y compris des personnes en dehors du pays chargées de vérifier le bon déroulement de cette étude, les membres des comités d'éthique et les autorités réglementaires, peuvent consulter vos dossiers médicaux. Tous les membres du personnel de l'étude sont formés à maintenir la confidentialité et ne supprimeront aucun document avec votre nom dans les dossiers de l'étude. Toutes ces personnes comprennent qu'elles doivent garder votre identité confidentielle et qu'elles ne partageront votre nom ou vos coordonnées avec personne.

Une description de cette étude sera disponible sur le site internet http://www.ClinicalTrials.gov. Ce site internet ne contiendra aucune information permettant de vous identifier. Vous pouvez consulter ce site internet à tout moment.

Les résultats de l'étude seront partagés avec la population, mais aucune information personnelle ne sera mentionnée dans les résultats de l'étude. Si vous avez des questions spécifiques sur vos données, contactez l'investigateur principal de l'étude, le Professeur Jean Jacques Muyembe ou son représentant (voir les coordonnées ci-dessous).

QUI VÉRIFIE QUE L'ÉTUDE EST SANS DANGER ?

Cette étude est mise en œuvre par des partenaires, comme le Ministère de la Santé de la RDC par l'intermédiaire de l'Institut national de recherche biomédicale (INRB) avec l'aide d'Epicentre et de la London School of Hygiene & Tropical Medicine (LSHTM). Un comité

For peer review only - http://bmjopen.bmj.com/site/about/guidelines.xhtml

indépendant composé de médecins et d'experts qui ne sont pas directement impliqués dans l'étude examinera les informations de l'étude et vérifiera l'innocuité des vaccins.

À QUI PUIS-JE PARLER DE CETTE ÉTUDE ?

Si vous souhaitez parler à quelqu'un à propos de cette étude ou si vous pensez avoir subi un préjudice en lien avec votre participation à l'étude, vous pouvez contacter les personnes suivantes.

1) L'investigateur principal responsable de cette étude

Professeur Jean Jacques Muyembe<br>*Directeur général,* Institut national de recherche biomédicale, Kinshasa, RDC<br>Téléphone : 0898 949 289<br>E-mail : jjmuyembet@gmail.com<br><br>Représentant locale de l'étude<br>Dr Hugo Kavunga<br>Téléphone: 0823 875 153<br>E-mail : hugokavunga@gmail.com

2) Les comités d'éthique de la RDC qui ont approuvé cette étude

Professeur Félicien Munday<br>Comité national d'éthique et de la santé<br>Kinshasa-Gombe, RDC<br>Téléphone : 0998 419 816<br>E-mail : feli1munday@yahoo.fr
Professeur Willy Bongopasi<br>Comité d'éthique de l'école de santé publique<br>Université de Kinshasa, RDC<br>Téléphone: 0999 952 341<br>E-mail : bongopasi@gmail.com

Si vous avez des questions concernant l'étude TUJIOKOWE ou sur vos droits en tant que participant(e) à l'étude, vous pouvez les poser à un membre de l'équipe de l'étude à tout moment.

FORMULAIRE DE CONSENTEMENT DESTINÉ AU/À LA PARTICIPANT(E) OU AU PARENT/TUTEUR

Titre : Étude TUJIOKOWE

Investigateur principal de cette étude : Prof. JJ Muyembe

For peer review only

For peer review only - http://bmjopen.bmj.com/site/about/guidelines.xhtml

**Déclarations**	**Signez ou apposez votre empreinte digitale dans chaque case**
J'ai lu les informations contenues dans ce formulaire concernant l'étude TUJIOKOWE (ou le personnel de l'étude m'a expliqué ces informations avec des mots que je comprends). Le but de cette étude et les procédures pour recevoir ce nouveau vaccin m'ont été expliqués en détail. J'ai pu poser des questions et j'ai reçu des réponses satisfaisantes à toutes mes questions.	
Je comprends que ma participation/la participation de mon enfant est volontaire, que je peux retirer mon consentement/mon enfant peut retirer son consentement et quitter cette étude à tout moment sans avoir à me/se justifier, et que cela n'affectera pas mes soins médicaux/les soins médicaux de mon enfant ni mes droits légaux/les droits légaux de mon enfant.	
J'ai été informé(e) que le nouveau vaccin Janssen Ebola est en cours d'étude, et que les risques possibles liés à l'administration de ce vaccin ne sont pas tous connus.	
Je confirme que je n'ai/mon enfant n'a **PAS** contracté le virus Ebola ou été vacciné(e) contre Ebola au cours des 30 derniers jours.	
J'accepte que l'équipe de l'étude me photographie/photographie mon enfant avant la vaccination.	
Si j'ai un numéro de téléphone, j'accepte de le donner à l'équipe de l'étude et j'accepte d'être contacté par appel ou SMS pour l'administration de la seconde dose ainsi que pour le suivi (ex. questions de santé).<br>Si je n'ai pas de téléphone, j'accepte dans la mesure du possible de fournir le numéro d'un proche ou d'un ami qui pourrait être utilisé pour me joindre.	
Je comprends que les informations recueillies à mon sujet/au sujet de mon enfant seront utilisées pour étayer d'autres recherches à l'avenir et peuvent être partagées de manière anonyme avec d'autres chercheurs.	
J'accepte que mes informations médicales/les informations médicales de mon enfant qui ne contiennent pas mon/son nom, puissent être partagées avec les autorités sanitaires nationales, le fabricant du vaccin (Janssen Vaccines & Prevention B.V.) et d'autres organismes étrangers en dehors de la RDC, comme la Food & Drug Administration aux Etats-Unis. J'autorise le personnel de ces organismes à consulter et analyser mes dossiers/les dossiers de mon enfant et les publier dans des revues scientifiques.	

FORMULAIRE DE CONSENTEMENT DESTINÉ AU/À LA PARTICIPANT(E) OU AU PARENT/TUTEUR

Déclarations concernant les procédures facultatives

**Déclarations**	**Signez ou apposez votre empreinte digitale dans chaque case si oui**	**Signez ou apposez votre empreinte digitale dans chaque case si non**

For peer review only - http://bmjopen.bmj.com/site/about/guidelines.xhtml

(Pour les femmes participantes uniquement) J'accepte que, si je suis/ma fille est enceinte au moment de la vaccination ou devient enceinte pendant le premier mois après avoir reçu chaque vaccin, et que je suis/ma fille est sélectionnée par l'équipe de l'étude pour faire partie du groupe femme enceinte, l'équipe de l'étude puisse me contacter plus frequement que les autres femmes enceintes incluses dans l'étude par téléphone, pendant ma grossesse/la grossesse de ma fille et après mon accouchement/l'accouchement de ma fille et pourrait passer à mon domicile/au domicile de ma fille si je/ma fille ne peut pas être contactée par téléphone.		
J'accepte que si je/mon enfant est choisi par l'équipe d'étude pour faire parti au groupe de suivi de l'innocuité, l'équipe d'étude peut me contacter par téléphone après ma deuxième vaccination, et peut me voir/mon enfant à la maison si je ne peux pas être contacté par téléphone.		

POUR LES PARTICIPANTS ADULTES

Je suis âgé(e) de 18 ans ou plus et j'accepte de participer à cette étude. (Signez ou apposez votre empreinte digitale ci-dessous)

		____/____/_____ *jj mmm aaaa*
Nom du/de la participant(e) adulte, en majuscules	Signature/Empreinte digitale du/de la participant(e) adulte	Date

POUR LE PARENT OU GARDIEN

Je suis le parent ou gardien d'un(e) participant(e) âgé(e) entre 1 et 17 ans et j'accepte que mon enfant participe à cette étude. (Signez ou apposez votre empreinte digitale ci-dessous)

		____/____/_____ *jj mmm aaaa*
Nom en caractères d'imprimerie du parent ou tuteur	Signature/Empreinte digitale du parent ou gardien	Date

POUR LE PARENT OU GARDIEN

For peer review only - http://bmjopen.bmj.com/site/about/guidelines.xhtml

Nom en caractères d'imprimerie du/de la participant(e) (si âgé[e] entre 1 et 17 ans)

		____/____/_____ *Jj mmm aaaa*
Nom de l'investigateur en caractères d'imprimerie	Signature de l'investigateur	Date

Remplissez la rubrique suivante si le/la participant(e) ou le parent/gardien est analphabète :

Témoin de l'entretien de consentement

J'ai assisté à l'entretien de consentement pour l'étude TUJIOKOWE dans ce document. J'atteste avoir expliqué les informations de l'étude avec précision au/à la participant(e) ou à son parent/tuteur et que ce dernier/cette dernière a compris, et qu'il/elle a donné librement son consentement pour participer/la participation de son enfant, en ma présence.

		____/____/_____ *jj mmm aaaa*
Nom du témoin impartial en caractères d'imprimerie	Signature du témoin impartial	Date

Apposez l'étiquette d'identification par code à barres ci-dessous :

For peer review only - http://bmjopen.bmj.com/site/about/guidelines.xhtml

For peer review only

Protocol for a phase 3 trial to evaluate effectiveness and safety of a heterologous, two-dose vaccine for Ebola virus disease in the Democratic Republic of the Congo

Appendix 2A

Informed Assent Form in English, Version 7.1 (10 Aug 2020)

For peer review only

For peer review only - http://bmjopen.bmj.com/site/about/guidelines.xhtml

Evaluation of a heterologous, two-dose preventive Ebola vaccine for effectiveness and safety in the Democratic Republic of the Congo

"The TUJIOKOWE Study" Information Sheet and Informed Assent Form for Children aged 12 years or older

LSHTM Protocol : DRC-EB-001

Principal Investigator (PI):
Professor Jean Jacques Muyembe
Director-General
Institut National de Recherche Biomédicale
Professor of Microbiology, Kinshasa University Medical School
Kinshasa Gombe, Democratic Republic of the Congo (DRC)
Phone: 0898 949 289; Email: jjmuyembet@gmail.com

Sponsor: London School of Hygiene & Tropical Medicine, United Kingdom

Site: Democratic Republic of the Congo

INTRODUCTION

You are being invited to take part in a research study called the 'TUJIOKOWE study' to find out if a new two-dose vaccine called the 'Janssen Ebola vaccine' can protect from getting Ebola and to check whether the vaccine is safe. The TUJIOKOWE study is being implemented by the Ministry of Health of DRC through the Institut National de Recherche Biomédicale (INRB), Epicentre and the London School of Hygiene & Tropical Medicine (LSHTM).

WHAT IS EBOLA?

Ebola is a disease that makes some people become very sick, and about half of the people who catch Ebola die from it. People catch Ebola from other people who have the disease.

WHAT IS A VACCINE AND WHAT IS A CLINICAL RESEARCH STUDY?

Vaccines are medical products that are used to stop people from getting certain diseases. You may have had vaccines when you were a young child to prevent you from catching diseases like measles and polio.

Clinical research studies help health professionals to understand if medicines or vaccines work to stop or treat diseases.

ARE ANY OTHER VACCINES BEING GIVEN TO PREVENT EBOLA IN DRC?

For peer review only - http://bmjopen.bmj.com/site/about/guidelines.xhtml

Lots of activities are ongoing to try to stop Ebola. Some people are being given a vaccine called VSV if they have been close to or looked after someone with Ebola, but that doesn't include everyone who might get Ebola – so we want to see if this vaccine can help.

WHAT IS THIS STUDY ABOUT?

People are getting sick with Ebola in North Kivu and Ituri provinces.

This study is going to find out if another new vaccine called the Janssen Ebola vaccine can help protect people against Ebola.

WHAT DO WE KNOW ABOUT THE VACCINE?

The Janssen Ebola vaccine is designed to protect people against Ebola. **It cannot make you sick with Ebola.** Over 6000 people have received the vaccine in many different countries, including7 in Africa. So far, the vaccine is safe in people who received it. The vaccine may help you fighting Ebola, but we don't know for sure yet if the Janssen Ebola vaccine can protect people against Ebola.

WHO CAN TAKE PART IN THIS STUDY?

To be given the Janssen Ebola vaccine, your parent or guardian must agree that you can join the study. You should also decide for yourself whether you want to join the study. You must be living in one of the communities chosen to be included in the study. You should be feeling well in order to get the vaccine.

People who have had Ebola cannot be in the TUJIOKOWE study. If you have received another Ebola vaccine or routine vaccine within the last month (30 days), or if you are sick on the day of vaccination, you may be asked to come back another time.

DO I HAVE TO BE IN THIS STUDY?

You do not have to join this study if you don't want to, even if your parents or guardians want you to join. If you agree to be in the study, we will ask you to sign this assent form. You can decide that you don't want to join, and it will not affect you in any way. If you don't want to be in the study at the moment, but you change your mind later, you can join the study when the vaccination team comes back. After that second visit by the team to your community, it may not be possible to get the Janssen Ebola vaccine. You need to be in the study to get this vaccine.

WHAT WILL HAPPEN IF I AGREE TO TAKE PART IN THIS STUDY?

Putting your name or your fingerprint on this assent form means that you agree to be in the study. You can change your mind and choose to leave the TUJIOKOWE study at any time.

For peer review only - http://bmjopen.bmj.com/site/about/guidelines.xhtml

You will be asked some questions about the study by one of the study staff members to check that you have understood the information.

WHAT DOES THE STUDY INVOLVE?

After you have signed this assent form, you will be asked some questions to check how you are feeling. If the doctor decides that you can be vaccinated, you will get an injection of the first Janssen Ebola vaccine against Ebola into your upper arm. We will give your parents or guardians a vaccination card with a phone number of who to contact in case you feel sick at any time after your visit. The vaccination card will also show the date of when you need to come back for the second Janssen Ebola vaccine. We will ask you for a phone number and we may text or call you for the second vaccine and follow ups.

In order to identify you at the next visit, we will take photographs of your face. We do this to make sure we know who has gotten the vaccine and that you receive the second dose at the right time.

A certain number of participants will be included in a group for follow up who will be contacted by phone or a home visit.

As a result of the study pause due to COVID-19, some participants had their second vaccination with the Janssen Ebola vaccine postponed. These participants will be contacted to receive their second vaccination at a later date. Receiving the second Janssen Ebola vaccine at a later date will not have negative effects on the health of these participants.

WHAT ARE THE POSSIBLE RISKS OF BEING IN THIS STUDY?

We do not know for sure yet if the Janssen Ebola vaccine protects people from Ebola, so there is a risk that you will not be protected from Ebola even after you have taken the vaccine.

As we have mentioned, this vaccine has been given to many people in other studies.

Some of the side effects that you may have after the injections are:

- pain, swelling, itchiness, redness, or warmth around the place where you got the injection and you may have a small sore which can happen after any other vaccine.
- in the area around the injection it may be sore and a bit painful to move for a few days.
- you may notice some swelling in your armpit or in your neck.
- you may get a fever or headache, feel tired, feel a bit sick or have some other side effects.

Not everyone gets these side effects and they should only last a few days. In a few people they may last for a few weeks and very rarely a few months.

In previous research studies some people have reported that they felt some tingling in the hands and feet, or their muscles felt weak. These symptoms usually only lasted one or two days, but sometimes for several weeks. One person reported tingling, numbness and pain which lasted for several months and interfered with the person's daily activities.

Very few people under 40 years old with other medical problems have had blood clots about 3

For peer review only - http://bmjopen.bmj.com/site/about/guidelines.xhtml

weeks after receiving a vaccine similar to the one we are giving; one woman died and one man had problems with his leg. These problems were not thought to be related to having the vaccine.

Hundreds of infants, children and teenagers have been given the Janssen Ebola vaccine. The most common problem reported by children is pain at the place where the injection was given. Older children and teenagers sometimes say they have headache, tiredness or can feel cold. Infants sometimes have less appetite or are less energetic or may have a fever for a short time. All these problems in children are the same as those after getting other vaccines and usually get better within 2 days. No serious medical problems have been reported in children who have been given the Janssen Ebola vaccine.

The medical team will help you if you don't feel well up until 1 month after your second injection. The medical team is available at any time if you have problems or questions.

WHAT ARE THE BENEFITS OF BEING IN THIS STUDY?

On the vaccination days a doctor will check your health status and you may receive treatment or a referral for simple medical conditions like malaria. You will also learn how to protect yourself against Ebola. Your participation in this study will help in the development of vaccines to prevent Ebola and, in the future, may help people in different parts of the world.

WHAT ABOUT PREGNANCY AND/OR BREASTFEEDING DURING THE STUDY?

Some older girls may be pregnant or breast feeding but they can still be in the study. We do not know all the effects of the Janssen Ebola vaccine in pregnancy or in a baby that is being breastfed. If you know you are pregnant or breastfeeding at the time of vaccination, please tell the study staff. If you are not sure, we will offer you a pregnancy test and meeting with a doctor so you can know whether you are pregnant before each injection. If you are pregnant or become pregnant within one month of each vaccination, we will contact you during your pregnancy until 3 months after delivery to ask about your health and the health of your baby. If we cannot contact you by phone after giving birth, the study team will visit you at your home. Among the pregnant women in the study, a number will be invited to join a subset and monitored more closely by telephone or through home visits. Some babies of women in this subset will also be seen within 3 months after birth. If you become pregnant within a month of vaccination, please call the phone number on your vaccination card and notify the study team.

WHAT DO I NEED TO DO FOR MY OWN HEALTH DURING THIS STUDY?

We will ask your parents or guardians to take you to a recommended health facility if you feel sick at any time during the study. They may also call the study contact number on your vaccination card to get advice up until 30 days after you receive the second dose of vaccine. You may be referred for medical care if necessary.

We do not know for sure yet if the Janssen Ebola vaccine can protect people against Ebola. **Therefore, you must continue to follow recommendations to protect yourself from Ebola.** We will give you information on how to prevent Ebola. If you experience anything like fever, diarrhea, vomiting, or unexplained bleeding, it is very important that you or your parents or

For peer review only - http://bmjopen.bmj.com/site/about/guidelines.xhtml

guardians let the study team know that you are sick and that you also get medical care as soon as possible.

WHO WILL BE ABLE TO SEE MY INFORMATION?

We will keep any information that you give us private.

Staff working on the study, as well as representatives of the ethics committee, including staff overseas, may look at your medical records. All of these people are trained to understand that they must keep your name and other details private.

The results of the study will be published in scientific journals so that other physicians and researchers can deepen their knowledge. It will not be possible to identify you.

If you have any specific questions about your data, please contact the study Principal Investigator, Professor Jean Jacques Muyembe or his representative (see contact details below).

WHO IS CHECKING THAT THE STUDY IS SAFE?

This study is being implemented by partners including the Ministry of Public Health of DRC through the Institut National de Recherche Biomédicale (INRB), Epicentre and the London School of Hygiene & Tropical Medicine (LSHTM). An independent committee made up of doctors and experts who are not directly involved in the study will be looking at the study information and checking that the study is safe.

For peer review only

For peer review only - http://bmjopen.bmj.com/site/about/guidelines.xhtml

WHO CAN I TALK TO ABOUT THIS STUDY?

If you or your parents or guardians would like to talk to someone about the TUJIOKOWE study or if you think you have been harmed by being part of the study, then your parents or guardians can contact the following people:

1) The Principal Investigator responsible for this study

Professor Jean Jacques Muyembe<br>*Director General,* Institut National de Recherche Biomédicale, Kinshasa, DRC<br>Phone: 0898 949 289<br>Email: jjmuyembet@gmail.com<br><br>Local study representative<br>Dr Hugo Kavunga<br>Phone: 0823 875 153<br>Email: hugokavunga@gmail.com

2) The DRC ethics committees that approved this study

Professor Félicien Munday<br>Comité National d'Ethique et de la Santé<br>Kinshasa-Gombe, DRC<br>Phone: 0998 419 816<br>Email: feli1munday@yahoo.fr
Professeur Willy Bongopasi<br>Comité d'éthique de l'école de santé Publique<br>Université de Kinshasa<br>Téléphone: 0999 952 341<br>Email: bongopasi@gmail.com

If you or your parents or guardians have any questions about the TUJIOKOWE study, you may ask someone on the study team at any time.

For peer review only - http://bmjopen.bmj.com/site/about/guidelines.xhtml

PARTICIPANT ASSENT FORM

Title: TUJIOKOWE Study

Principal Investigator of this study: Prof. JJ Muyembe

Statements	Please sign or fingerprint each box
I understand the information in this form, and I was able to ask questions and all of my questions were answered to my satisfaction.	
I understand that joining this study is voluntary and I can choose to leave the study at any time without it affecting me.	
I have been informed that the new Janssen Ebola vaccine is still being tested, and that not all the possible risks from having this vaccine are completely known.	
I confirm that I have **NOT** previously had Ebola or been vaccinated against Ebola within the past 30 days	
I agree to have my photograph taken by the study team before vaccination.	
I agree to give the study team a contact phone number if I have one and to be contacted for the second vaccine and for follow up calls or SMS e.g. for health questions. If I don't have a phone, I agree to try and provide a relative or friend's number that can be used to contact me.	
I understand that the information collected about me will be used to support other research in the future and may be shared anonymously with other researchers.	
I agree that my medical information that will not contain my name, can be shared with the national health authorities, the vaccine manufacturer (Janssen Vaccines & Prevention B.V.) and other foreign organizations outside the DRC, such as the US Food & Drug Administration. I give permission for individuals in these organizations to access and analyse my records and publish them in scientific journals.	

For peer review only

PARTICIPANT ASSENT FORM

Declarations regarding optional procedures

**Statements**	**Please sign or fingerprint each box for yes**	**Please sign or fingerprint each box for no**
(For female participants only) I agree that if I am pregnant or become pregnant during the first month after vaccination and I am selected to join the pregnancy subset, I may be telephoned during my pregnancy more often than other pregnant participants in the study and after the birth of my baby to collect some information on my health and my baby's health. If this information cannot be collected by telephone, I agree that I may be visited at home by a member of the study team.		
I agree that if I am selected by the study team to be in the safety group, the study team may contact me by telephone after my second vaccination and may see me at home if I cannot be contacted by telephone.		

I agree to take part in this study. (Please sign or put your fingerprint below)

		____/____/_____ *dd mon yyyy*
Printed name of child	Signature/fingerprint of child	Date

		____/____/_____ *dd mon yyyy*
Printed name of investigator	Signature of investigator	Date

For peer review only - http://bmjopen.bmj.com/site/about/guidelines.xhtml

Complete if participant is illiterate:

Witness to Assent Interview

I witnessed the assent interview for the TUJIOKOWE Study in this document. I attest that I have explained the study information accurately to the participant and was understood to the best of my knowledge by the participant, and that he/she has freely given their assent to participate in my presence.

		____/____/_____ *dd mon yyyy*
Printed name of impartial witness	Signature of impartial witness	Date

Attach ID barcode label below:

For peer review only

For peer review only - http://bmjopen.bmj.com/site/about/guidelines.xhtml

Protocol for a phase 3 trial to evaluate effectiveness and safety of a heterologous, two-dose vaccine for Ebola virus disease in the Democratic Republic of the Congo

Appendix 2B

Informed Assent Form in French, Version 7.1 (10 Aug 2020)

For peer review only

For peer review only - http://bmjopen.bmj.com/site/about/guidelines.xhtml

Évaluation de l'efficacité et de l'innocuité de l'emploi d'un vaccin préventif hétérologue à deux doses contre Ebola en République démocratique du Congo

Note d'information et formulaire d'assentiment éclairé de l'« étude TUJIOKOWE » destinés aux enfants âgés de 12 ans ou plus

Protocole LSHTM : DRC-EB-001

Investigateur principal (IP) :
Professeur Jean Jacques Muyembe
Directeur général
Institut national de recherche biomédicale
Professeur de microbiologie, Faculté de médecine de l'Université de Kinshasa
Kinshasa Gombe, République démocratique du Congo (RDC)
Téléphone : 0898 949 289 ; E-mail : jjmuyembet@gmail.com

Promoteur : London School of Hygiene & Tropical Medicine, Royaume-Uni

Site : République démocratique du Congo

INTRODUCTION

Tu es invité(e) à participer à une étude de recherche appelée l'« étude TUJIOKOWE » pour savoir si un nouveau vaccin à deux doses appelé « vaccin Janssen Ebola » peut protéger contre le virus Ebola et pour vérifier si le vaccin est sans danger. L'étude TUJIOKOWE est mise en œuvre par le ministère de la santé publique de la RDC par l'intermédiaire de l'Institut national de recherche biomédicale (INRB), d'Epicentre et de la London School of Hygiene & Tropical Medicine (LSHTM).

QU'EST-CE QUE LE VIRUS EBOLA ?

Ebola est une maladie qui rend certaines personnes très malades, et environ la moitié des personnes qui contractent le virus Ebola en meurent. Le virus Ebola est transmis par des personnes atteintes de la maladie.

QU'EST-CE QU'UN VACCIN ET QU'EST-CE QU'UNE ÉTUDE DE RECHERCHE CLINIQUE ?

Les vaccins sont des produits médicaux utilisés pour empêcher les personnes d'avoir certaines maladies. Tu peux avoir eu des vaccins lorsque tu étais un jeune enfant pour te protéger de maladies comme la rougeole et la polio.

For peer review only - http://bmjopen.bmj.com/site/about/guidelines.xhtml

Les études de recherche clinique aident les personnels de santé à comprendre si les médicaments ou les vaccins agissent pour arrêter ou traiter les maladies.

D'AUTRES VACCINS SONT-ILS ADMINISTRÉS POUR PRÉVENIR LE VIRUS EBOLA EN RDC ?

De nombreuses activités sont en cours pour essayer d'arrêter le virus Ebola. Certaines personnes reçoivent un vaccin appelé le vaccin VSV si elles ont été en contact avec une personne infectée par Ebola ou si elles s'en sont en contact avec quelqu'un qui s'occupe des malades, mais cela n'inclut pas tous ceux qui peuvent avoir Ebola dans l'avenir - alors nous voulons voir si ce vaccin peut aider.

EN QUOI CONSISTE CETTE ÉTUDE ?

Dans les provinces du Nord-Kivu et d'Ituri, des individus sont infectés par le virus Ebola.

Cette étude va déterminer si un autre nouveau vaccin, le vaccin Janssen Ebola, peut aider à protéger contre Ebola.

QUE SAVONS-NOUS DU VACCIN ?

Le vaccin Janssen Ebola est conçu pour protéger la population contre Ebola. **Tu ne peux pas contracter le virus Ebola à cause du vaccin.** Plus de 6 000 personnes ont reçu le vaccin dans de nombreux pays, dont 7 en Afrique. Jusqu'à présent, le vaccin est sûr chez les personnes vaccinées. Le vaccin pourrait t'aider lutter contre les infections à virus Ebola, mais nous ne savons pas encore avec certitude si le vaccin Janssen Ebola peut protéger contre Ebola.

QUI PEUT PARTICIPER À CETTE ÉTUDE ?

Pour recevoir le vaccin Janssen Ebola, tes parents/gardiens doivent accepter que tu participes à l'étude. Tu dois également décider si tu souhaites participer à l'étude. Pour être inclus(e) dans l'étude, tu dois faire partie de l'une des communautés sélectionnées. Tu dois te sentir bien afin de recevoir le vaccin.

Les personnes qui ont été atteintes du virus Ebola ne peuvent pas participer à l'étude TUJIOKOWE.

Si tu as été déjà vacciné contre la maladie à virus Ebola ou un autre vaccin de routine durant les 30 derniers jours ou si tu es malade le jour de vaccination, tu seras vacciné un autre jour.

SUIS-JE OBLIGÉ(E) DE PARTICIPER À CETTE ÉTUDE ?

Tu n'es pas obligé(e) de participer à cette étude si tu ne veux pas le faire, même si tes parents ou tes gardiens veulent que tu y participes. Si tu acceptes de participer à l'étude, nous te demanderons de signer ce formulaire d'assentiment. Tu peux décider que tu ne souhaites pas participer et cela n'aura aucun effet sur toi. Si tu ne souhaites pas participer à l'étude pour le moment, mais que tu changes d'avis plus tard, tu peux participer à l'étude lorsque l'équipe de vaccination revient dans ta communauté. Après cette deuxième visite au sein de ta

For peer review only - http://bmjopen.bmj.com/site/about/guidelines.xhtml

communauté, il peut ne plus être possible de recevoir le vaccin Janssen Ebola. Il faut être dans l'étude pour recevoir le vaccin.

QUE SE PASSERA-T-IL SI J'ACCEPTE DE PARTICIPER À CETTE ÉTUDE ?

En mettant ton nom ou en apposant ton empreinte digitale sur ce formulaire d'assentiment, tu acceptes de participer à l'étude. Tu peux changer d'avis et décider de quitter l'étude Janssen Ebola à tout moment. L'un des membres du personnel de l'étude te posera des questions sur l'étude pour vérifier que tu as compris l'information.

QU'IMPLIQUE L'ÉTUDE ?

Après avoir signé ce formulaire d'assentiment, on te posera quelques questions pour vérifier comment tu te sens. Si le médecin décide que tu peux être vacciné(e), tu recevras une injection de la première dose de vaccin de Janssen Ebola contre Ebola dans la partie supérieure de ton bras. Nous donnerons à tes parents ou gardiens une carte de vaccination avec le numéro de téléphone de personnes à contacter au cas où tu te sens mal, à tout moment après ta visite. La carte de vaccination indiquera également la date à laquelle tu devras revenir pour la deuxième dose de vaccin Janssen Ebola. Votre numéro de téléphone vous sera demandé, il est possible que vous soyez contacté afin de vous rappeler le rendez-vous de l'administration du second vaccin et également pour assurer un suivi.

Afin de t'identifier lors de la visite suivante, nous allons prendre une photographie de ton visage. Nous le faisons afin de veiller à ce que tu reçoives la deuxième dose au bon moment et pour savoir qui a été vacciné.

Un certain nombre de participants seront inclus dans un groupe de suivi qui sera contacté par téléphone ou une visite à domicile.
Suite à la pause de l'étude due à la COVID-19, certains participants ont vu leur deuxième vaccination avec le vaccin Janssen Ebola reportée. Ces participants seront contactés pour recevoir leur deuxième vaccination à une date ultérieure. Le fait de recevoir le deuxième vaccin Janssen Ebola à une date ultérieure n'aura pas d'effets négatifs sur la santé de ces participants.

QUELS SONT LES ÉVENTUELS RISQUES LIÉS À LA PARTICIPATION À CETTE ÉTUDE ?

Nous ne savons pas encore avec certitude si le vaccin Janssen Ebola protège la population contre Ebola. Il existe donc un risque que tu ne sois pas protégé(e) contre le virus Ebola, même après la vaccination.

Comme nous l'avons mentionné, ce vaccin a été administré à de nombreuses personnes dans d'autres études.

Certains des effets secondaires que tu pourrais avoir après les injections sont les suivants :

- Douleur, gonflement, démangeaisons, rougeur ou sensation de chaleur autour de l'endroit où tu as reçu l'injection et tu pourrais être un peu endolori(e) comme après tout vaccin.

For peer review only - http://bmjopen.bmj.com/site/about/guidelines.xhtml

- Au site autour de l'injection, tu pourrais ressentir de la douleur lors de tes mouvements pendant quelques jours.
- Tu pourrais remarquer un gonflement sous ton bras ou dans ton cou.
- Tu pourrais avoir de la fièvre ou des maux de tête, une sensation de fatigue, te sentir un peu mal ou avoir d'autres effets secondaires.

Tout le monde ne présente pas ces effets secondaires, et ils ne durent que quelques jours. Chez certaines personnes, ils peuvent durer pendant quelques semaines et très rarement quelques mois.

Dans des études de recherche précédentes, certaines personnes ont signalé qu'elles avaient des picotements dans les mains et les pieds, ou une faiblesse musculaire. Généralement, ces symptômes ne duraient qu'un jour ou deux, mais parfois ils ont duré pendant plusieurs semaines. Une personne a signalé des picotements, un engourdissement et une douleur qui ont duré pendant plusieurs mois et interféré avec ses activités quotidiennes.

Très peu de personnes de moins de 40 ans ayant d'autres problèmes médicaux ont eu des caillots de sang environ 3 semaines après avoir reçu un vaccin similaire à celui que nous administrons ; une femme est morte et un homme a eu des problèmes à la jambe. Ces problèmes n'ont pas été considérés comme étant liés à l'administration du vaccin.

Des centaines de nourrissons, d'enfants et d'adolescents ont reçu le vaccin Janssen Ebola. Le problème le plus fréquent signalé par les enfants est la douleur à l'endroit où l'injection a été faite. Les enfants plus âgés et les adolescents disent parfois avoir mal à la tête, être fatigués ou avoir froid. Les nourrissons ont parfois moins d'appétit ou sont moins énergiques ou peuvent avoir de la fièvre pendant une courte période. Tous ces problèmes chez les enfants sont les mêmes qu'après avoir reçu d'autres vaccins et se résorbent généralement en deux jours. Aucun problème médical grave n'a été signalé chez les enfants qui ont reçu le vaccin Janssen Ebola.

L'équipe médicale vous aidera si vous ne vous sentez pas bien jusqu'à 1 mois après votre deuxième injection. Elle est disponible à tout moment si vous avez des problèmes ou des questions.

QUELS SONT LES AVANTAGES LIÉS À LA PARTICIPATION À L'ÉTUDE ?

Les jours d'administration du vaccin, un médecin vérifiera ton état de santé et tu pourrais recevoir un traitement ou être redirigé vers un spécialiste pour des affections médicales simples comme le paludisme. Tu apprendras aussi comment te protéger contre le virus Ebola. Ta participation à cette étude contribuera au développement de vaccins pour prévenir le virus Ebola et, à l'avenir, pourrait aider des personnes dans différentes régions du monde.

QU'EN EST-IL DE LA GROSSESSE ET/OU DE L'ALLAITEMENT PENDANT L'ÉTUDE ?

Certaines filles plus âgées peuvent être enceintes ou allaiter, mais elles peuvent toujours

For peer review only - http://bmjopen.bmj.com/site/about/guidelines.xhtml

participer à l'étude. Nous ne sommes pas sûr de tous les effets des vaccins de l'étude pendant la grossesse ou chez un bébé allaité. Si vous savez que vous êtes enceinte ou si vous allaitez au moment de la vaccination, informez-en le personnel de l'étude. Si vous n'êtes pas certaine, nous vous proposerons un test de grossesse et un entretien avec du personnel médical qui vous renseignera avant chaque vaccination. Si vous êtes enceinte ou si vous tombez enceinte dans le mois qui suit l'administration d'une des deux doses, vous serez contactee durant votre grossesse afin de vous poser des questions concernant votre sante et celle votre bebe. Dans ce cas, nous vous contacterons pendant votre grossesse et juste après votre accouchement pour vous poser des questions sur votre état de santé et celui de votre bébé. Nous vous contacterons également dans les 3 mois après votre accouchement. Si nous ne pouvons pas vous contacter par téléphone après votre accouchement, l'équipe de l'étude vous rendra visite à votre domicile. Parmi les femmes enceintes dans l'étude, un certain nombre de participante seront invitée à faire partie d'un sous-groupe pour assurer un suivi plus rapproché par téléphone ou au domicile. Certains nouveaux-nés de maman faisant partie du sous-groupe seront également suivis dans les 3 mois suivant la naissance. Si vous tombez enceinte dans le mois qui suit une vaccination, merci d'appeler le numéro de téléphone indiqué sur votre carte de vaccination et de prévenir l'équipe de l'étude.

QUE DOIS-JE FAIRE POUR MA PROPRE SANTÉ PENDANT CETTE ÉTUDE ?

Nous allons demander à tes parents ou gardiens de t'emmener à un établissement de santé recommandé si tu te sens malade à tout moment pendant l'étude. Ils peuvent également appeler le numéro de contact de l'étude indiqué sur ta carte de vaccination pour obtenir des conseils et ce jusqu'à 30 jours après avoir reçu la seconde dose du vaccin. Tu pourrais être orienté(e) vers des soins médicaux si nécessaire.

Nous ne savons pas encore avec certitude si le vaccin Janssen Ebola peut protéger la population contre Ebola. **Par conséquent, tu dois continuer à suivre les recommandations pour te protéger contre le virus Ebola.** Nous te donnerons des informations sur la façon de prévenir le virus Ebola. Si tu présentes des symptômes comme de la fièvre, de la diarrhée, des vomissements ou des saignements inexpliqués, il est très important que toi ou tes parents/gardiens informiez l'équipe de l'étude que tu es malade et que tu reçoives des soins médicaux dès que possible.

QUI POURRA CONSULTER MES INFORMATIONS ?

Nous préserverons la confidentialité des informations que tu nous donnes.

Le personnel travaillant sur l'étude, y compris les personnes en dehors du pays, peuvent consulter ton dossier médical. Toutes ces personnes sont formées à la confidentialité et comprennent qu'elles doivent garder ton nom et d'autres informations privés.
Les résultats de l'étude seront publiés dans des revues scientifiques afin que d'autres médecins et chercheurs puissent approfondir leurs connaissances. Il ne sera pas possible de t'identifier.

Si tu as des questions spécifiques sur tes données, contacte l'investigateur principal de l'étude, le Professeur Jean Jacques Muyembe ou son représentant (voir les coordonnées ci-dessous).

For peer review only - http://bmjopen.bmj.com/site/about/guidelines.xhtml

QUI VÉRIFIE QUE L'ÉTUDE EST SANS DANGER ?

Cette étude est mise en œuvre par des partenaires, comme le Ministère de la Santé de la RDC par l'intermédiaire de l'Institut national de recherche biomédicale (INRB), d'Epicentre et de la London School of Hygiene & Tropical Medicine (LSHTM). Un comité indépendant composé de médecins et d'experts qui ne sont pas directement impliqués dans l'étude examinera les informations de l'étude et vérifiera la sûreté de l'étude.

For peer review only

For peer review only - http://bmjopen.bmj.com/site/about/guidelines.xhtml

À QUI PUIS-JE PARLER DE CETTE ÉTUDE ?

Si tes parents/gardiens ou toi souhaitez parler à quelqu'un de l'étude TUJIOKOWE ou si tu penses avoir subi un préjudice en lien avec ta participation à l'étude, tes parents ou gardiens peuvent contacter les personnes suivantes :

1) L'investigateur principal responsable de cette étude

Professeur Jean Jacques Muyembe<br>*Directeur général,* Institut national de recherche biomédicale, Kinshasa, RDC<br>Téléphone : 0898 949 289<br>E-mail : jjmuyembet@gmail.com<br><br>Représentant locale de l'étude<br>Dr Hugo Kavunga<br>Téléphone : 0823 875 153<br>E-mail : hugokavunga@gmail.com

2) Les comités d'éthique de la RDC qui ont approuvé cette étude

Professeur Félicien Munday<br>Comité National d'Ethique et de la Santé<br>Kinshasa-Gombe, RDC<br>Téléphone : 0998 419 816<br>E-mail : feli1munday@yahoo.fr
Professeur Willy Bongopasi<br>Comité d'éthique de l'école de santé publique<br>Université de Kinshasa, RDC<br>Téléphone : 0999 952 341<br>E-mail : bongopasi@gmail.com

Si tes parents/gardiens ou toi avez des questions concernant l'étude TUJIOKOWE, vous pouvez les poser à un membre de l'équipe de l'étude à tout moment.

For peer review only - http://bmjopen.bmj.com/site/about/guidelines.xhtml

FORMULAIRE D'ASSENTIMENT DU/DE LA PARTICIPANT(E)

Titre : Étude TUJIOKOWE

Investigateur principal de cette étude : Prof. JJ Muyembe

**Déclarations**	**Signe ou appose ton empreinte digitale dans chaque case**
Je comprends les informations contenues dans ce formulaire et j'ai pu poser des questions et toutes mes questions ont reçu une réponse satisfaisante.	
Je comprends que ma participation à cette étude est volontaire et que je peux choisir de quitter l'étude à tout moment sans que cela ne m'affecte.	
J'ai été informé(e) que le nouveau vaccin Janssen Ebola est en cours d'étude, et que les risques possibles liés à l'administration de ce vaccin ne sont pas tous connus.	
Je confirme que je n'ai **PAS** contracté le virus Ebola ou été vacciné(e) contre Ebola au cours des 30 derniers jours	
J'accepte que l'équipe de l'étude me photographie avant la vaccination.	
Si j'ai un numéro de téléphone, j'accepte de le donner à l'équipe de l'étude et j'accepte d'être contacté par appel ou SMS pour l'administration de la seconde dose ainsi que pour le suivi (ex. questions de santé). Si je n'ai pas de téléphone, j'accepte dans la mesure du possible de fournir le numéro d'un proche ou d'un ami qui pourrait être utilisé pour me joindre.	
Je comprends que les informations recueillies à mon sujet seront utilisées pour étayer d'autres recherches à l'avenir et peuvent être partagées de manière anonyme avec d'autres chercheurs.	
J'accepte que mes informations médicales qui ne contiennent pas mon nom, puissent être partagées avec les autorités sanitaires nationales, le fabricant du vaccin (Janssen Vaccines & Prevention B.V.) et d'autres organismes étrangers en dehors de la RDC, comme la Food & Drug Administration des Etats Unis. J'autorise le personnel de ces organismes à consulter et analyser mes dossiers et les publier dans des revues scientifiques.	

For peer review only - http://bmjopen.bmj.com/site/about/guidelines.xhtml

FORMULAIRE D'ASSENTIMENT DU/DE LA PARTICIPANT(E)

Déclarations concernant les procédures facultatives

**Déclarations**	**Signe ou appose ton empreinte digitale dans chaque case si oui**	**Signe ou appose ton empreinte digitale dans chaque case si non**
(Pour les femmes participantes uniquement) J'accepte que si je suis enceinte au moment de la vaccination ou devient enceinte pendant le premier mois après avoir reçu chaque vaccin, et que je suis sélectionnée pour faire partie du groupe Grossesse, et qu'un membre de l'équipe de l'étude puisse me contacter plus frequement que les autres femmes enceintes incluses dans l'étude par téléphone pendant ma grossesse et, j'accepte également le suivi après mon accouchement pour recueillir des informations sur ma santé et celle de mon bébé. Si ces informations ne peuvent pas être recueillies par téléphone, j'accepte qu'un membre de l'équipe de l'étude vienne me voir à mon domicile.		
J'accepte que si je suis choisi par l'équipe d'étude pour faire appel au groupe de sécurité, l'équipe d'étude peut me contacter par téléphone après ma deuxième vaccination, et peut me voir à la maison si je ne peux pas être contacté par téléphone.		

J'accepte de participer à cette étude. (Signe ou appose ton empreinte digitale ci-dessous)

		____/____/_____ *jj mmm aaaa*
Nom de l'enfant en caractères d'imprimerie	Signature/Empreinte digitale de l'enfant	Date

		____/____/_____ *jj mmm aaaa*
Nom de l'investigateur en caractères d'imprimerie	Signature de l'investigateur	Date

For peer review only - http://bmjopen.bmj.com/site/about/guidelines.xhtml

Remplir si le/la participant(e) est analphabète :

Témoin de l'entretien d'assentiment

J'ai assisté à l'entretien d'assentiment pour l'étude [insérer nom] dans ce document. J'atteste avoir expliqué les informations de l'étude avec précision au/à la participant(e) et que ce dernier/cette dernière a compris, et qu'il/elle a donné librement son assentiment pour participer, en ma présence.

		____/____/_____ *jj mmm aaaa*
Nom du témoin impartial en caractères d'imprimerie	Signature du témoin impartial	Date

Apposez l'étiquette d'identification par code à barres ci-dessous :

For peer review only

Protocol for a phase 3 trial to evaluate effectiveness and safety of a heterologous, two-dose vaccine for Ebola virus disease in the Democratic Republic of the Congo

Appendix 3A

Immunogenicity Informed Consent Form for adults in English, Version 1.1 (10 Aug 2020)

For peer review only

For peer review only - http://bmjopen.bmj.com/site/about/guidelines.xhtml

Evaluation of a heterologous, two-dose preventive Ebola vaccine for effectiveness and safety in the Democratic Republic of the Congo

"The TUJIOKOWE Study" Immunogenicity Subset Information Sheet and Informed Consent Form

LSHTM Protocol: DRC-EB-001

Principal Investigator (PI):
Professor Jean Jacques Muyembe
Director-General
Institut National de Recherche Biomédicale
Professor of Microbiology, Kinshasa University Medical School
Kinshasa Gombe, Democratic Republic of the Congo (DRC)
Phone: 0898 949 289; Email: jjmuyembet@gmail.com

Sponsor: London School of Hygiene & Tropical Medicine, United Kingdom

Site: Democratic Republic of the Congo

INTRODUCTION

The 'TUJIOKOWE study' is a research study to find out if a new two-dose vaccine called the 'Janssen Ebola vaccine' can protect people from getting Ebola and to check whether the vaccine is safe. The TUJIOKOWE study is being implemented by the Ministry of Health of DRC through the Institut National de Recherche Biomédicale (INRB), Epicentre, and the London School of Hygiene & Tropical Medicine.

When we started the TUJIOKOWE study, we planned to give everybody the second dose of the Ebola vaccine about two months after the first dose. Some people will now receive the second dose of Ebola vaccine later than originally planned. This is because it was necessary to temporarily close the study clinics due to the COVID-19 outbreak. We want to understand if people who receive their second dose of vaccine later than two months have the same immune responses as the people who received their second dose of vaccine at two months after the first dose. To do this, we will need to collect blood samples from approximately 50 adults, 25 adolescents, and 25 children participating in the TUJIOKOWE study who received the second dose of the Ebola vaccine after July 2020. We are asking you to join this small study (which we are calling the Immunogenicity Subset) and to provide two blood samples because you will be receiving the second dose of the vaccine after July 2020. If you agree to participate, one blood sample will be collected from you before the second dose of Ebola vaccine is given and the second blood sample will be collected 21 days later.

For peer review only - http://bmjopen.bmj.com/site/about/guidelines.xhtml

We would like to be sure that your body responds to the vaccine as well as people who received the second dose of the vaccine two months after the first dose. To check this, we will be looking at the antibodies that your body produces after you receive a vaccine. Antibodies are produced by your body after vaccination to protect you from infection. The information from testing your blood for antibodies will be very important when the vaccine is used to protect against Ebola in the future because it may not always be possible to give the second dose at exactly two months after the first dose of the vaccine.

If you are the parent or guardian of a child under 18 years of age and you agree to your child providing blood samples, you will need to sign separate documents for you and your child. If your child is aged 12 to 17 years old, he/she will also need to agree to provide blood samples himself/herself and sign a different document, called the immunogenicity assent form.

DO I HAVE TO GIVE BLOOD SAMPLES?

You do not have to provide any blood samples. If you agree to provide blood samples, we will ask you to sign this consent form. If you do not want to give any blood samples, this will not affect your participation in the TUJIOKOWE study.

WHAT WILL HAPPEN IF I AGREE TO GIVE BLOOD SAMPLES?

We will describe the blood collection process and answer any questions that you may have. If you would like a copy of the written information, we will give it to you. You will be asked to sign or put your fingerprint on this consent form. Putting your name or your fingerprint on the consent form means that you agree to provide blood samples, but you can change your mind at any time.

After you have given your first blood sample, you will follow the normal TUJIOKOWE study procedures to receive the second dose of vaccine. To have your second blood sample taken you will need to come to the study clinic for an extra visit 21 days after you have received the second dose of vaccine. If you participate in the Immunogenicity Subset, then we will give you some refreshments at the clinic.

WHAT DOES GIVING BLOOD INVOLVE?

A small amount of your blood will be collected from you using a small clean needle. We will take blood before you get the second injection of vaccine and at 21 days after the second injection. To have your second blood sample taken, you will need to come to the study clinic for an extra visit 21 days after you have received the second dose of vaccine. For adults, we will collect approximately 5 mL (about 1 teaspoon) of blood each time you provide a blood sample.

As explained earlier, the blood samples you give will be used to test whether your body has produced antibodies in your blood against Ebola after vaccination. You will not receive the results of these tests because they are only for scientific research.

For peer review only - http://bmjopen.bmj.com/site/about/guidelines.xhtml

After we do our tests on your blood samples for the Immunogenicity Subset of the TUJIOKOWE study, we will destroy any remaining blood that has been collected from you. We will not use your blood for any other purpose except the antibody tests that we have explained in this consent form. Your blood samples will not be sold.

Your blood samples will be labelled with a code and not your name. Other information, such as sex, age, or health history might also be shared with other investigators, but your name will not be shared.

WHAT ARE THE POSSIBLE RISKS OF GIVING A BLOOD SAMPLE?

Providing a blood sample is generally safe but occasionally people experience pain, bruising, bleeding, or (very rarely) infection from giving a blood sample. The medical team will provide care if you experience side-effects from the blood sampling. They are available if you have any problems or questions.

WHAT ARE THE BENEFITS OF GIVING A BLOOD SAMPLE?

There is no direct benefit to you for providing blood samples in the Immunogenicity Subset of the TUJIOKOWE study. Your agreement to provide blood samples will help in the development of vaccines to prevent Ebola and, in the future, may help people in different parts of the world.

WHAT HAPPENS TO THE BLOOD SAMPLES COLLECTED FROM ME?

The blood samples collected from you during the Immunogenicity Subset of the TUJIOKOWE study, will only be used to understand how the Ebola vaccine works when the second dose is given more than two months after the first dose.

The samples will be coded in a way that only limited study staff can link them to you. Your blood samples will be tested in a laboratory in the United States. If any blood remains in DRC, it will be stored in the INRB laboratory in Goma. We will destroy all the samples in the United States and in Goma at the end of the study.

For peer review only - http://bmjopen.bmj.com/site/about/guidelines.xhtml

WHO CAN I TALK TO ABOUT GIVING BLOOD SAMPLES?

If you want to talk to someone about giving a blood sample or if you think you have been harmed by providing a blood sample, you can contact the following people:

1) The Principal Investigator responsible for this study

Professor Jean Jacques Muyembe<br>*Director General,* Institut National de Recherche Biomédicale, Kinshasa, DRC<br>Phone: 0898 949 289<br>Email: jjmuyembet@gmail.com<br><br>Local study representative<br>Dr Hugo Kavunga<br>Phone: 0823 875 153<br>Email: hugokavunga@gmail.com

2) The DRC ethics committees that approved this study

Professeur Félicien Munday<br>National Ethics Committee<br>Kinshasa-Gombe, DRC<br>Phone: 0998 419 816<br>Email: feli1munday@yahoo.fr
Professeur Willy Bongopasi<br>Comité d'éthique de l'école de santé publique<br>Université de Kinshasa<br>Phone: 0999 952 341<br>Email: bongopasi@gmail.com

If you have any questions about providing blood samples for the Immunogenicity Subset of the TUJIOKOWE study or about your rights, you may ask anyone on the study team at any time.

For peer review only - http://bmjopen.bmj.com/site/about/guidelines.xhtml

PARTICIPANT IMMUNOGENICITY SUBSET CONSENT FORM

Title: Immunogenicity Subset of the TUJIOKOWE Study

Principal Investigator of this study: Prof. JJ Muyembe

Statements	Please sign or fingerprint each box
I have read the information in this form about giving a blood sample at two visits for the Immunogenicity Subset of the TUJIOKOWE Study (or I have had this information explained to me by the study staff in a language that I understand). The purpose of providing blood samples and the procedures to give blood samples have been fully explained to me. I was able to ask questions and have all of these questions answered to my satisfaction.	
I understand that my participation is voluntary, that I can withdraw consent to provide blood samples at any time without giving any reason, and that this will not affect my participation in the TUJIOKOWE Study.	

I am aged 18 years or older and I agree to provide Immunogenicity Subset blood samples during my participation in the TUJIOKOWE study. (Please sign or put your fingerprint below)

		____/____/_____ *dd mon yyyy*
Printed name of adult participant	Signature/fingerprint of adult participant	Date

		____/____/_____ *dd mon yyyy*
Printed name of investigator	Signature of investigator	Date

For peer review only - http://bmjopen.bmj.com/site/about/guidelines.xhtml

Complete this section if the participant is illiterate:

Witness to Consent Interview

I witnessed the consent interview for the Immunogenicity Subset of the TUJIOKOWE Study in this document. I attest that I have explained the study information accurately to the participant or parent/guardian and was understood to the best of my knowledge by the participant and that he/she has freely given their consent to participate in my presence.

		____/____/_____ *dd mon yyyy*
Printed name of impartial witness	Signature of impartial witness	Date

Attach ID barcode label below:

For peer review only

For peer review only - http://bmjopen.bmj.com/site/about/guidelines.xhtml

Protocol for a phase 3 trial to evaluate effectiveness and safety of a heterologous, two-dose vaccine for Ebola virus disease in the Democratic Republic of the Congo

Appendix 3B

Immunogenicity Informed Consent Form for adults in French, Version 1.1 (10 Aug 2020)

For peer review only

For peer review only - http://bmjopen.bmj.com/site/about/guidelines.xhtml

Évaluation de l'efficacité et de la sécurité d'emploi d'un vaccin préventif hétérologue à deux doses contre Ebola en République démocratique du Congo

Note d'information et formulaire de consentement éclairé pour le sous-groupe Immunogénicité de l'« étude TUJIOKOWE »

Protocole LSHTM : DRC-EB-001

Investigateur principal (IP) :
Professeur Jean Jacques Muyembe
Directeur général
Institut national de recherche biomédicale
Professeur de microbiologie, Faculté de médecine de l'Université de Kinshasa
Kinshasa Gombe, République démocratique du Congo (RDC)
Téléphone : 0898 949 289 ; adresse électronique : jjmuyembet@gmail.com

Promoteur : London School of Hygiene & Tropical Medicine, Royaume-Uni

Centre : République démocratique du Congo

INTRODUCTION

L'« étude TUJIOKOWE » est une étude de recherche visant à déterminer si un nouveau vaccin à deux doses appelé « vaccin contre le virus Ebola de Janssen » peut protéger contre le virus Ebola et à vérifier si le vaccin est sans danger. L'étude TUJIOKOWE est mise en œuvre par le ministère de la Santé de la RDC par l'intermédiaire de l'Institut national de recherche biomédicale (INRB), Epicentre et la London School of Hygiene & Tropical Medicine.

Lorsque nous avons commencé l'étude TUJIOKOWE, nous avions prévu d'administrer la seconde dose du vaccin contre le virus Ebola à tous les participants 2 mois environ après la première dose. Certaines personnes vont maintenant recevoir la seconde dose du vaccin contre le virus Ebola plus tard que prévu. Cela s'explique par la nécessité de fermer temporairement les centres de l'étude en raison de l'épidémie de COVID-19. Nous voulons comprendre si les personnes qui reçoivent leur seconde dose de vaccin plus de 2 mois après présentent la même réponse immunitaire que les personnes qui l'ont reçue 2 mois après la première dose. À cet effet, nous allons devoir prélever des échantillons de sang auprès de 50 adultes, 25 adolescents et 25 enfants environ participant à l'étude TUJIOKOWE, qui ont reçu la seconde dose du vaccin contre le virus Ebola après juillet 2020. Nous vous demandons de participer à cette petite étude (que nous appelons « sous-groupe Immunogénicité ») et de fournir deux échantillons de sang, car vous allez recevoir la seconde dose du vaccin après juillet 2020. Si vous acceptez de participer à l'étude, un échantillon de sang vous sera prélevé avant l'administration de la seconde dose du vaccin contre le virus Ebola, et le second échantillon de sang sera prélevé 21 jours plus tard.

For peer review only - http://bmjopen.bmj.com/site/about/guidelines.xhtml

Nous voulons nous assurer que votre organisme réagit au vaccin aussi bien que celui des personnes qui ont reçu la seconde dose du vaccin 2 mois après la première dose. À cet effet, nous allons rechercher les anticorps que produit votre organisme après l'administration du vaccin. Les anticorps sont produits par l'organisme après la vaccination pour vous protéger contre l'infection. Les informations obtenues en analysant votre sang pour rechercher la présence d'anticorps seront très importantes lorsque le vaccin sera utilisé pour protéger contre le virus Ebola à l'avenir, car il ne sera peut-être pas toujours possible d'administrer la seconde dose du vaccin 2 mois exactement après la première dose.

Si vous êtes le parent ou le tuteur d'un enfant de moins de 18 ans et si vous acceptez qu'il fournisse les échantillons de sang, vous devrez signer des documents distincts pour vous et votre enfant. Si votre enfant est âgé de 12 à 17 ans, il devra également accepter de lui-même de fournir les échantillons de sang et signer un autre document, appelé « formulaire d'assentiment » pour le sous-groupe Immunogénicité.

SUIS-JE OBLIGÉ DE DONNER DES ÉCHANTILLONS DE SANG ?

Vous n'avez pas l'obligation de fournir des échantillons de sang. Si vous acceptez de fournir des échantillons de sang, nous vous inviterons à signer ce formulaire de consentement. Si vous ne souhaitez pas donner d'échantillons de sang, cela ne remettra pas en cause votre participation à l'étude TUJIOKOWE.

QUE SE PASSERA-T-IL SI J'ACCEPTE DE DONNER DES ÉCHANTILLONS DE SANG ?

Nous allons décrire les modalités de prise de sang et répondre à toutes les questions que vous pourriez avoir. Si vous souhaitez une copie de cette note d'information, nous vous la remettrons. Nous vous inviterons à signer ce formulaire de consentement ou à y apposer votre empreinte digitale. En inscrivant votre nom ou en apposant votre empreinte digitale sur le formulaire de consentement, vous acceptez de fournir des échantillons de sang, mais vous pourrez changer d'avis à tout moment.

Une fois que vous aurez donné votre premier échantillon de sang, vous suivrez les procédures habituelles de l'étude TUJIOKOWE pour recevoir la seconde dose de vaccin. Pour le prélèvement de votre second échantillon de sang, vous devrez venir effectuer une visite supplémentaire au centre de l'étude 21 jours après l'administration de la seconde dose de vaccin. Si vous participez au sous-groupe Immunogénicité, chaque fois que vous donnerez votre sang, nous vous donnerons des rafraîchissements.

For peer review only - http://bmjopen.bmj.com/site/about/guidelines.xhtml

EN QUOI CONSISTE LE PRÉLÈVEMENT DE SANG ?

Une petite quantité de votre sang vous sera prélevée à l'aide d'une petite aiguille propre. Nous vous prélèverons un échantillon de sang avant que vous receviez la seconde injection du vaccin et 21 jours après la seconde injection. Pour le prélèvement de votre second échantillon de sang, vous devrez effectuer une visite supplémentaire au centre de l'étude 21 jours après l'administration de la seconde dose de vaccin. Pour les adultes, nous prélèverons 5 ml environ (soit 1 cuillère à café environ) de sang chaque fois que vous fournirez un échantillon de sang.

Comme indiqué précédemment, les échantillons de sang que vous donnerez serviront à vérifier si votre organisme a produit des anticorps dans votre sang contre le virus Ebola après la vaccination. Vous ne recevrez pas les résultats de ces analyses, car ils sont destinés uniquement à la recherche scientifique.

Après avoir réalisé nos analyses sur vos échantillons de sang pour le sous-groupe Immunogénicité de l'étude TUJIOKOWE, nous détruirons tous les échantillons de sang restants qui vous ont été prélevés. Nous n'utiliserons pas vos échantillons de sang à d'autres fins que les analyses de détection d'anticorps mentionnés dans ce formulaire de consentement. Vos échantillons de sang ne seront pas vendus.

Vos échantillons de sang seront étiquetés avec un code et non avec votre nom. D'autres informations, comme le sexe, l'âge ou les antécédents médicaux, pourront également être partagées avec d'autres investigateurs, mais votre nom ne sera pas communiqué.

QUELS SONT LES RISQUES ÉVENTUELS LIÉS AU DON D'UN ÉCHANTILLON DE SANG ?

Le prélèvement d'un échantillon de sang est généralement sans danger, mais il arrive que des personnes ressentent des douleurs ou présentent un bleu, un saignement ou (dans de très rares cas) une infection après un prélèvement de sang. Si vous présentez des effets secondaires après la prise de sang, l'équipe médicale vous apportera les soins nécessaires. L'équipe est disponible à tout moment si vous avez des problèmes ou des questions.

QUELS SONT LES BÉNÉFICES LIÉS AU DON D'UN ÉCHANTILLON DE SANG ?

Il n'y a aucun bénéfice direct pour vous à fournir des échantillons de sang dans le cadre du sous-groupe Immunogénicité de l'étude TUJIOKOWE. Le consentement à fournir des échantillons de sang contribuera au développement de vaccins pour prévenir le virus Ebola et, à l'avenir, pourrait aider des personnes dans différentes régions du monde.

QU'ADVIENDRA-IL DES ÉCHANTILLONS DE SANG QUI ME SERONT PRÉLEVÉS ?

Les échantillons de sang qui vous ont été prélevés dans le cadre du sous-groupe Immunogénicité de l'étude TUJIOKOWE ne seront utilisés que pour comprendre l'efficacité du vaccin contre le virus Ebola lorsque la seconde dose est administrée plus de 2 mois après la première dose.

For peer review only - http://bmjopen.bmj.com/site/about/guidelines.xhtml

Les échantillons seront codés de manière à limiter le nombre de membres du personnel de l'étude qui peut les relier à vous. Vos échantillons de sang seront analysés dans un laboratoire aux États-Unis. S'il reste des échantillons de sang en RDC, ils seront conservés dans le laboratoire de l'INRB à Goma. Nous détruirons tous les échantillons conservés aux États-Unis et à Goma à la fin de l'étude.

À QUI PUIS-JE M'ADRESSER CONCERNANT LE PRÉLÈVEMENT D'ÉCHANTILLONS DE SANG ?

Si vous souhaitez parler à quelqu'un du prélèvement d'un échantillon de sang ou si vous pensez avoir subi un préjudice en fournissant un échantillon de sang, vous pouvez contacter les personnes suivantes :

1) Investigateur principal responsable de cette étude

Professeur Jean Jacques Muyembe<br>*Directeur général*, Institut national de recherche biomédicale, Kinshasa, RDC<br>Téléphone : 0898 949 289<br>Adresse électronique : jjmuyembet@gmail.com<br><br>Représentant locale de l'étude<br>Dr Hugo Kavunga<br>Téléphone : 0823 875 153<br>Adresse électronique : hugokavunga@gmail.com

2) Comités d'éthique de la RDC ayant approuvé cette étude

Professeur Félicien Munday<br>Comité national d'éthique et de la santé<br>Kinshasa-Gombe, RDC<br>Téléphone : 0998 419 816<br>Adresse électronique : feli1munday@yahoo.fr
Professeur Willy Bongopasi<br>Comité d'éthique de l'école de santé publique<br>Université de Kinshasa, RDC<br>Téléphone : 0999 952 341<br>Adresse électronique : bongopasi@gmail.com

Si vous avez des questions sur le prélèvement d'échantillons de sang pour le sous-groupe Immunogénicité de l'étude TUJIOKOWE ou sur vos droits, vous pouvez les poser à tout moment à n'importe quel membre de l'équipe de l'étude.

For peer review only - http://bmjopen.bmj.com/site/about/guidelines.xhtml

FORMULAIRE DE CONSENTEMENT DES PARTICIPANTS POUR LE SOUS-GROUPE IMMUNOGÉNICITÉ

Titre : Sous-groupe Immunogénicité de l'étude TUJIOKOWE

Investigateur principal de cette étude : Prof. JJ Muyembe

**Déclarations**	**Signez ou apposez votre empreinte digitale dans chaque case**
J'ai lu les informations contenues dans ce formulaire sur le prélèvement d'un échantillon de sang lors de deux visites pour le sous-groupe Immunogénicité de l'étude TUJIOKOWE (ou le personnel de l'étude m'a expliqué ces informations avec des mots que je comprends). L'objectif du prélèvement d'échantillons de sang et les procédures correspondantes m'ont été expliqués en détail. J'ai pu poser des questions et j'ai reçu des réponses satisfaisantes à toutes mes questions.	
Je comprends que ma participation est volontaire, que je peux retirer son consentement à fournir des échantillons de sang à tout moment sans avoir à me justifier et que cela ne remettra pas en cause ma participation à l'étude TUJIOKOWE.	

J'ai 18 ans ou plus et j'accepte de fournir des échantillons de sang pour le sous-groupe Immunogénicité pendant ma participation à l'étude TUJIOKOWE. (Signez ou apposez votre empreinte digitale ci-dessous)

		____/____/_____ *jj mmm aaaa*
Nom du participant adulte (en caractères d'imprimerie)	Signature/Empreinte digitale du participant adulte	Date

		____/____/_____ *jj mmm aaaa*
Nom de l'investigateur (en caractères d'imprimerie)	Signature de l'investigateur	Date

For peer review only - http://bmjopen.bmj.com/site/about/guidelines.xhtml

Si le participant est analphabète, remplissez cette section :

Témoin de l'entretien de consentement

J'ai assisté à l'entretien de consentement pour le sous-groupe Immunogénicité de l'étude TUJIOKOWE décrite dans ce document. J'atteste avoir expliqué avec exactitude les informations sur l'étude au participant ou au parent/tuteur et que, à ma connaissance, le participant les a comprises et a donné librement son consentement à participer, en ma présence.

		____/____/_____ *jj mmm aaaa*
Nom du témoin impartial (en caractères d'imprimerie)	Signature du témoin impartial	Date

Apposez l'étiquette d'identification par code à barres ci-dessous :

For peer review only

For peer review only - http://bmjopen.bmj.com/site/about/guidelines.xhtml

Protocol for a phase 3 trial to evaluate effectiveness and safety of a heterologous, two-dose vaccine for Ebola virus disease in the Democratic Republic of the Congo

Appendix 4A

Immunogenicity Informed Assent Form for children in English, Version 1.1 (10 Aug 2020)

For peer review only

For peer review only - http://bmjopen.bmj.com/site/about/guidelines.xhtml

Evaluation of a heterologous, two-dose preventive Ebola vaccine for effectiveness and safety in the Democratic Republic of the Congo

"The TUJIOKOWE Study" Immunogenicity Subset
Information Sheet and Informed Assent Form for Children Aged 12 Years or Older

LSHTM Protocol: DRC-EB-001

Principal Investigator (PI):
Professor Jean Jacques Muyembe
Director-General
Institut National de Recherche Biomédicale
Professor of Microbiology, Kinshasa University Medical School
Kinshasa Gombe, Democratic Republic of the Congo (DRC)
Phone: 0898 949 289; Email: jjmuyembet@gmail.com

Sponsor: London School of Hygiene & Tropical Medicine, United Kingdom

Site: Democratic Republic of the Congo

INTRODUCTION

The 'TUJIOKOWE study' is a research study to find out if a new two-dose vaccine called the 'Janssen Ebola vaccine' can protect people from getting Ebola and to check whether the vaccine is safe. The TUJIOKOWE study is being implemented by the Ministry of Health of DRC through the Institut National de Recherche Biomédicale (INRB), Epicentre, and the London School of Hygiene & Tropical Medicine.

When we started the TUJIOKOWE study, we planned to give everybody the second injection of the Ebola vaccine about two months after the first injection. Some people will now receive the second injection of Ebola vaccine later than originally planned. This is because we needed to temporarily close the study clinics due to the COVID-19 outbreak. We want to understand if the Ebola vaccine works well if people receive their second injection later than two months. To do this, we will need to collect blood samples from about 50 adults, 25 adolescents, and 25 children participating in the TUJIOKOWE study who received the second injection of the Ebola vaccine after July 2020. We are asking you to join this small study (which we are calling the Immunogenicity Subset) and to provide two blood samples because you will be receiving the second injection of the vaccine after July 2020. If you agree to participate, one blood sample will be collected from you before the second injection of Ebola vaccine is given and the second blood sample will be collected 21 days later.

We would like to be sure that your body responds to the vaccine as well as people who received the second injection of the vaccine two months after the first injection. To check this, we will

For peer review only - http://bmjopen.bmj.com/site/about/guidelines.xhtml

be looking at the antibodies that your body produces after you receive a vaccine. Antibodies are produced by your body after vaccination to protect you from infection. The information from testing your blood for antibodies will be very important when the vaccine is used to protect against Ebola in the future because it may not always be possible to give the second injection at exactly two months after the first injection of the vaccine.

DO I HAVE TO GIVE BLOOD SAMPLES?

You do not have to provide any blood samples if you do not want to, even if your parents or guardians want you to. If you agree to provide blood samples, we will ask you to sign this assent form. If you do not want to give any blood samples, this will not affect your participation in the TUJIOKOWE study.

WHAT WILL HAPPEN IF I AGREE TO GIVE BLOOD SAMPLES?

We will describe the blood collection process and answer any questions that you may have. If you would like a copy of the written information, we will give it to you. You will be asked to sign or put your fingerprint on this assent form. Putting your name or your fingerprint on the assent form means that you agree to provide blood samples, but you can change your mind at any time.

After the first blood sample you give, you will follow the normal TUJIOKOWE study procedures to receive the second injection of vaccine. To have your second blood sample taken you will need to come to the study clinic for an extra visit 21 days after you have received the second injection of vaccine. If you participate in the immunogenicity subset, we will give you some refreshments at the clinic.

WHAT DOES GIVING BLOOD INVOLVE?

A small amount of your blood will be collected from you using a small clean needle. We will take blood before you get the second injection of vaccine and at 21 days after the second injection. To have your second blood sample taken you will need to come to the study clinic for an extra visit 21 days after you have received the second injection of vaccine. For children aged 6 years and older, we will collect approximately 5 mL (about 1 teaspoon) of blood each time you provide a blood sample.

As explained to you earlier, the blood samples you give will be used to test whether your body has produced antibodies in your blood against Ebola after vaccination. You will not receive the results of these tests because they are only for scientific research.

After we do our tests on your blood samples for the Immunogenicity Subset of the TUJIOKOWE study, we will destroy any remaining blood that you have given us. We will not use your blood for any other purpose except the antibody tests that we have explained in this assent form. Your blood samples will not be sold.

Your blood samples will be labelled with a code and not your name. Other information, such as sex, age, or health history might also be shared with other investigators, but your name will not be shared.

For peer review only - http://bmjopen.bmj.com/site/about/guidelines.xhtml

WHAT ARE THE POSSIBLE RISKS OF GIVING A BLOOD SAMPLE?

Giving a blood sample is generally safe but occasionally people experience pain, bruising, bleeding, or (very rarely) infection from giving a blood sample. The medical team will provide care if you experience side-effects from the blood sampling. They are available if you have any problems or questions.

WHAT ARE THE BENEFITS OF GIVING A BLOOD SAMPLE?

There is no direct benefit to you for providing blood samples in the Immunogenicity Subset of the TUJIOKOWE study. Your agreement to provide blood samples will help in the development of vaccines to prevent Ebola and, in the future, may help people in different parts of the world.

WHAT HAPPENS TO THE BLOOD SAMPLES COLLECTED FROM ME?

The blood samples collected from you during the Immunogenicity Subset of the TUJIOKOWE study, will only be used to understand how the Ebola vaccine works when the second injection is given more than two months after the first injection.

The samples will be coded in a way that only limited study staff can link them to you. Your blood samples will be tested in a laboratory in the United States. If any blood remains in DRC, it will be stored in the INRB laboratory in Goma. We will destroy all the samples in the United States and in Goma at the end of the study.

For peer review only

For peer review only - http://bmjopen.bmj.com/site/about/guidelines.xhtml

WHO CAN I TALK TO ABOUT GIVING BLOOD SAMPLES?

If you want to talk to someone about giving a blood sample or if you think you have been harmed by providing a blood sample, you can contact the following people:

1) The Principal Investigator responsible for this study

Professor Jean Jacques Muyembe<br>*Director General,* Institut National de Recherche Biomédicale, Kinshasa, DRC<br>Phone: 0898 949 289<br>Email: jjmuyembet@gmail.com<br><br>Local study representative<br>Dr Hugo Kavunga<br>Phone: 0823 875 153<br>Email: hugokavunga@gmail.com

2) The DRC ethics committees that approved this study

Professeur Félicien Munday<br>National Ethics Committee<br>Kinshasa-Gombe, DRC<br>Phone: 0998 419 816<br>Email: feli1munday@yahoo.fr
Professeur Willy Bongopasi<br>Comité d'éthique de l'école de santé publique<br>Université de Kinshasa<br>Phone: 0999 952 341<br>Email: bongopasi@gmail.com

If you have any questions about providing blood samples for the Immunogenicity Subset of the TUJIOKOWE study or about your rights, you may ask anyone on the study team at any time.

For peer review only - http://bmjopen.bmj.com/site/about/guidelines.xhtml

PARTICIPANT IMMUNOGENICITY SUBSET ASSENT FORM

Title: Immunogenicity Subset of the TUJIOKOWE Study

Principal Investigator of this study: Prof. JJ Muyembe

**Statements**	**Please sign or fingerprint each box**
I have read the information in this form about giving a blood sample at two visits for the Immunogenicity Subset of the TUJIOKOWE Study (or I have had this information explained to me by the study staff in a language that I understand). The purpose of providing blood samples and the procedures to give blood samples have been fully explained to me. I was able to ask questions and have all of these questions answered to my satisfaction.	
I understand that my participation is voluntary, that I can withdraw consent to provide blood samples at any time without giving any reason, and that this will not affect my participation in the TUJIOKOWE Study.	

I am aged 12-17 years and I agree to provide Immunogenicity Subset blood samples during my participation in the TUJIOKOWE study. (Please sign or put your fingerprint below)

		____/____/_____ *dd mon yyyy*
Printed name of child	Signature/fingerprint of child	Date

		____/____/_____ *dd mon yyyy*
Printed name of investigator	Signature of investigator	Date

For peer review only - http://bmjopen.bmj.com/site/about/guidelines.xhtml

Complete this section if the participant is illiterate:

Witness to Assent Interview

I witnessed the assent interview for the Immunogenicity Subset of the TUJIOKOWE Study in this document. I attest that I have explained the study information accurately to the participant and was understood to the best of my knowledge by the participant and that he/she has freely given their assent to participate in my presence.

		____/____/_____ *dd mon yyyy*
Printed name of impartial witness	Signature of impartial witness	Date

Attach ID barcode label below:

For peer review only - http://bmjopen.bmj.com/site/about/guidelines.xhtml

Protocol for a phase 3 trial to evaluate effectiveness and safety of a heterologous, two-dose vaccine for Ebola virus disease in the Democratic Republic of the Congo

Appendix 4B

Immunogenicity Informed Assent Form for children in French, Version 1.1 (10 Aug 2020)

For peer review only

For peer review only - http://bmjopen.bmj.com/site/about/guidelines.xhtml

Évaluation de l'efficacité et de la sécurité d'emploi d'un vaccin préventif hétérologue à deux doses contre Ebola en République démocratique du Congo

Note d'information et formulaire d'assentiment éclairé pour le sous-groupe Immunogénicité de l'« étude TUJIOKOWE » destinés aux enfants âgés de 12 ans ou plus

Protocole LSHTM : DRC-EB-001

Investigateur principal (IP) :
Professeur Jean Jacques Muyembe
Directeur général
Institut national de recherche biomédicale
Professeur de microbiologie, Faculté de médecine de l'Université de Kinshasa
Kinshasa Gombe, République démocratique du Congo (RDC)
Téléphone : 0898 949 289 ; Adresse électronique : jjmuyembet@gmail.com

Promoteur : London School of Hygiene & Tropical Medicine, Royaume-Uni

Centre : République démocratique du Congo

INTRODUCTION

L'« étude TUJIOKOWE » est une étude de recherche visant à déterminer si un nouveau vaccin à deux doses appelé « vaccin contre le virus Ebola de Janssen » peut protéger contre le virus Ebola et à vérifier si le vaccin est sans danger. L'étude TUJIOKOWE est mise en œuvre par le ministère de la Santé de la RDC par l'intermédiaire de l'Institut national de recherche biomédicale (INRB), Epicentre et la London School of Hygiene & Tropical Medicine.

Lorsque nous avons commencé l'étude TUJIOKOWE, nous avions prévu d'administrer la seconde injection du vaccin contre le virus Ebola à tous les participants 2 mois environ après la première injection. Certaines personnes vont maintenant recevoir la seconde injection du vaccin contre le virus Ebola plus tard que prévu. Cela s'explique par la nécessité de fermer temporairement les centres de l'étude en raison de l'épidémie de COVID-19. Nous voulons savoir si le vaccin contre le virus Ebola est aussi efficace lorsque les personnes reçoivent leur seconde injection plus de 2 mois après. À cet effet, nous allons devoir prélever des échantillons de sang auprès de 50 adultes, 25 adolescents et 25 enfants environ participant à l'étude TUJIOKOWE, qui ont reçu la seconde injection du vaccin contre le virus Ebola après juillet 2020. Nous vous demandons de participer à cette petite étude (que nous appelons « sous-groupe Immunogénicité ») et de fournir deux échantillons de sang, car vous allez recevoir la seconde injection du vaccin après juillet 2020. Si vous acceptez de participer à l'étude, un

For peer review only - http://bmjopen.bmj.com/site/about/guidelines.xhtml

échantillon de sang vous sera prélevé avant l'administration de la seconde injection du vaccin contre le virus Ebola, et le second échantillon de sang sera prélevé 21 jours plus tard.

Nous voulons nous assurer que votre organisme réagit au vaccin aussi bien que celui des personnes qui ont reçu la seconde injection du vaccin 2 mois après la première injection. À cet effet, nous allons rechercher les anticorps que produit votre organisme après l'administration du vaccin. Les anticorps sont produits par l'organisme après la vaccination pour vous protéger contre l'infection. Les informations obtenues en analysant votre sang pour rechercher la présence d'anticorps seront très importantes lorsque le vaccin sera utilisé pour protéger contre le virus Ebola à l'avenir, car il ne sera peut-être pas toujours possible d'administrer la seconde injection du vaccin 2 mois exactement après la première injection du vaccin.

SUIS-JE OBLIGÉ DE DONNER DES ÉCHANTILLONS DE SANG ?

Vous n'êtes pas obligé de fournir des échantillons de sang si vous ne le souhaitez pas, même si vos parents ou tuteurs le souhaitent. Si vous acceptez de fournir des échantillons de sang, nous vous inviterons à signer ce formulaire d'assentiment. Si vous ne souhaitez pas donner d'échantillons de sang, cela ne remettra pas en cause votre participation à l'étude TUJIOKOWE.

QUE SE PASSERA-T-IL SI J'ACCEPTE DE DONNER DES ÉCHANTILLONS DE SANG ?

Nous allons décrire les modalités de prise de sang et répondre à toutes les questions que vous pourriez avoir. Si vous souhaitez une copie de cette note d'information, nous vous la remettrons. Nous vous inviterons à signer ce formulaire d'assentiment ou à y apposer votre empreinte digitale. En inscrivant votre nom sur le formulaire d'assentiment ou en y apposant votre empreinte digitale, vous acceptez de fournir des échantillons de sang, mais vous pourrez changer d'avis à tout moment.

Une fois que vous aurez donné votre premier échantillon de sang, vous suivrez les procédures habituelles de l'étude TUJIOKOWE pour recevoir la seconde injection du vaccin. Pour le prélèvement de votre second échantillon de sang, vous devrez venir effectuer une visite supplémentaire au centre de l'étude 21 jours après l'administration de la seconde injection du vaccin. Si vous participez au sous-groupe Immunogénicité, chaque fois que vous donnerez votre sang, nous vous donnerons des rafraîchissements.

EN QUOI CONSISTE LE PRÉLÈVEMENT DE SANG ?

Une petite quantité de votre sang vous sera prélevée à l'aide d'une petite aiguille propre. Nous vous prélèverons un échantillon de sang avant que vous receviez la seconde injection du vaccin et 21 jours après la seconde injection. Pour le prélèvement de votre second échantillon de sang, vous devrez venir effectuer une visite supplémentaire au centre de l'étude 21 jours après l'administration de la seconde injection du vaccin. Pour les enfants âgés de 6 ans et plus, nous prélèverons 5 ml environ (soit 1 cuillère à café environ) de sang chaque fois que vous fournirez un échantillon de sang.

For peer review only - http://bmjopen.bmj.com/site/about/guidelines.xhtml

Comme indiqué précédemment, les échantillons de sang que vous donnerez serviront à vérifier si votre organisme a produit des anticorps dans votre sang contre le virus Ebola après la vaccination. Vous ne recevrez pas les résultats de ces analyses, car ils sont destinés uniquement à la recherche scientifique.

Après avoir réalisé nos analyses sur vos échantillons de sang pour le sous-groupe Immunogénicité de l'étude TUJIOKOWE, nous détruirons tous les échantillons de sang restants qui vous ont été prélevés. Nous n'utiliserons pas vos échantillons de sang à d'autres fins que les analyses de détection d'anticorps mentionnés dans ce formulaire d'assentiment. Vos échantillons de sang ne seront pas vendus.

Vos échantillons de sang seront étiquetés avec un code et non avec votre nom. D'autres informations, comme le sexe, l'âge ou les antécédents médicaux, pourront également être partagées avec d'autres investigateurs, mais votre nom ne sera pas communiqué.

QUELS SONT LES RISQUES ÉVENTUELS LIÉS AU DON D'UN ÉCHANTILLON DE SANG ?

Le prélèvement d'un échantillon de sang est généralement sans danger, mais il arrive que des personnes ressentent des douleurs ou présentent un bleu, un saignement ou (dans de très rares cas) une infection après un don de sang. Si vous présentez des effets secondaires après la prise de sang, l'équipe médicale vous apportera les soins nécessaires. L'équipe est disponible à tout moment si vous avez des problèmes ou des questions.

QUELS SONT LES BÉNÉFICES LIÉS AU DON D'UN ÉCHANTILLON DE SANG ?

Il n'y a aucun bénéfice direct pour vous à fournir des échantillons de sang dans le cadre du sous-groupe Immunogénicité de l'étude TUJIOKOWE. Le consentement à fournir des échantillons de sang contribuera au développement de vaccins pour prévenir le virus Ebola et, à l'avenir, pourrait aider des personnes dans différentes régions du monde.

QU'ADVIENDRA-IL DES ÉCHANTILLONS DE SANG QUI ME SERONT PRÉLEVÉS ?

Les échantillons de sang qui vous ont été prélevés dans le cadre du sous-groupe Immunogénicité de l'étude TUJIOKOWE ne seront utilisés que pour comprendre l'efficacité du vaccin contre le virus Ebola lorsque la seconde injection est administrée plus de 2 mois après la première injection.

Les échantillons seront codés de manière à limiter le nombre de membres du personnel de l'étude qui peut les relier à vous. Vos échantillons de sang seront analysés dans un laboratoire aux États-Unis. S'il reste des échantillons de sang en RDC, ils seront conservés dans le laboratoire de l'INRB à Goma. Nous détruirons tous les échantillons conservés aux États-Unis et à Goma à la fin de l'étude.

For peer review only - http://bmjopen.bmj.com/site/about/guidelines.xhtml

À QUI PUIS-JE M'ADRESSER CONCERNANT LE PRÉLÈVEMENT D'ÉCHANTILLONS DE SANG ?

Si vous souhaitez parler à quelqu'un du prélèvement d'un échantillon de sang ou si vous pensez avoir subi un préjudice en fournissant un échantillon de sang, vous pouvez contacter les personnes suivantes :

1) Investigateur principal responsable de cette étude

Professeur Jean-Jacques Muyembe<br>*Directeur général*, Institut national de recherche biomédicale, Kinshasa, RDC<br>Téléphone : 0898 949 289<br>Adresse électronique : jjmuyembet@gmail.com<br><br>Représentant locale de l'étude<br>Dr Hugo Kavunga<br>Téléphone : 0823 875 153<br>Adresse électronique : hugokavunga@gmail.com

2) Comités d'éthique de la RDC qui ont approuvé cette étude

Professeur Félicien Munday<br>Comité national d'éthique et de la santé<br>Kinshasa-Gombe, RDC<br>Téléphone : 0998 419 816<br>Adresse électronique : feli1munday@yahoo.fr
Professeur Willy Bongopasi<br>Comité d'éthique de l'école de santé publique<br>Université de Kinshasa, RDC<br>Téléphone : 0999 952 341<br>Adresse électronique : bongopasi@gmail.com

Si vous avez des questions sur le prélèvement d'échantillons de sang pour le sous-groupe Immunogénicité de l'étude TUJIOKOWE ou sur vos droits, vous pouvez les poser à tout moment à n'importe quel membre de l'équipe de l'étude.

For peer review only - http://bmjopen.bmj.com/site/about/guidelines.xhtml

FORMULAIRE DE ASSENTIMENT DES PARTICIPANTS POUR LE SOUS-GROUPE IMMUNOGÉNICITÉ

Titre : Sous-groupe Immunogénicité de l'étude TUJIOKOWE

Investigateur principal de cette étude : Prof. JJ Muyembe

**Déclarations**	**Signez ou apposez votre empreinte digitale dans chaque case**
J'ai lu les informations contenues dans ce formulaire sur le prélèvement d'un échantillon de sang lors de deux visites pour le sous-groupe Immunogénicité de l'étude TUJIOKOWE (ou le personnel de l'étude m'a expliqué ces informations avec des mots que je comprends). L'objectif du prélèvement d'échantillons de sang et les procédures correspondantes m'ont été expliqués en détail. J'ai pu poser des questions et j'ai reçu des réponses satisfaisantes à toutes mes questions.	
Je comprends que ma participation est volontaire, que je peux retirer son consentement à fournir des échantillons de sang à tout moment sans avoir à me justifier et que cela ne remettra pas en cause ma participation à l'étude TUJIOKOWE.	

Je suis âgé de 12 à 17 ans et j'accepte de fournir des échantillons de sang pour le sous-groupe Immunogénicité pendant ma participation à l'étude TUJIOKOWE. (Signez ou apposez votre empreinte digitale ci-dessous)

		____/____/_____<br>*jj mmm aaaa*
Nom de l'enfant (en caractères d'imprimerie)	Signature/Empreinte digitale de l'enfant	Date

		____/____/_____<br>*jj mmm aaaa*
Nom de l'investigateur (en caractères d'imprimerie)	Signature de l'investigateur	Date

For peer review only - http://bmjopen.bmj.com/site/about/guidelines.xhtml

Si le participant est analphabète, remplissez cette section :

Témoin de l'entretien d'Assentiment

J'ai assisté à l'entretien d'assentiment pour le sous-groupe Immunogénicité de l'étude TUJIOKOWE décrite dans ce document. J'atteste avoir expliqué avec exactitude les informations sur l'étude au participant et que, à ma connaissance, le participant les a comprises et a donné librement son assentiment à participer, en ma présence.

		____/____/_____ *jj mmm aaaa*
Nom du témoin impartial (en caractères d'imprimerie)	Signature du témoin impartial	Date

Apposez l'étiquette d'identification par code à barres ci-dessous :

For peer review only - http://bmjopen.bmj.com/site/about/guidelines.xhtml

Protocol for a phase 3 trial to evaluate effectiveness and safety of a heterologous, two-dose vaccine for Ebola virus disease in the Democratic Republic of the Congo

Appendix 5A

Immunogenicity Informed Consent Form for Parents in English, Version 1.1 (10 Aug 2020)

For peer review only

For peer review only - http://bmjopen.bmj.com/site/about/guidelines.xhtml

Evaluation of a heterologous, two-dose preventive Ebola vaccine for effectiveness and safety in the Democratic Republic of the Congo

"The TUJIOKOWE Study" Immunogenicity Subset Information Sheet and Informed Consent Form for Parents/Guardians of Paediatric Participants

LSHTM Protocol: DRC-EB-001

Principal Investigator (PI):
Professor Jean Jacques Muyembe
Director-General
Institut National de Recherche Biomédicale
Professor of Microbiology, Kinshasa University Medical School
Kinshasa Gombe, Democratic Republic of the Congo (DRC)
Phone: 0898 949 289; Email: jjmuyembet@gmail.com

Sponsor: London School of Hygiene & Tropical Medicine, United Kingdom

Site: Democratic Republic of the Congo

INTRODUCTION

The 'TUJIOKOWE study' is a research study to find out if a new two-dose vaccine called the 'Janssen Ebola vaccine' can protect people from getting Ebola and to check whether the vaccine is safe. The TUJIOKOWE study is being implemented by the Ministry of Health of DRC through the Institut National de Recherche Biomédicale (INRB) and with Médecins Sans Frontières, Epicentre, and the London School of Hygiene & Tropical Medicine.

When we started the TUJIOKOWE study, we planned to give everybody the second dose of the Ebola vaccine about two months after the first dose. Some people will now receive the second dose of Ebola vaccine later than originally planned. This is because it was necessary to temporarily close the study clinics due to the COVID-19 outbreak. We want to understand if people who receive their second dose of vaccine later than two months have the same immune responses as the people who received their second dose of vaccine at two months after the first dose. To do this, we will need to collect blood samples from approximately 50 adults, 25 adolescents, and 25 children participating in the TUJIOKOWE study who received the second dose of the Ebola vaccine after July 2020. We are asking your child to join this small study (which we are calling the Immunogenicity Subset) and to provide two blood samples because your child will be receiving the second dose of the vaccine after July 2020. If you agree for your child to participate, one blood sample will be collected from your child before the second dose of Ebola vaccine is given and the second blood sample will be collected 21 days later.

For peer review only - http://bmjopen.bmj.com/site/about/guidelines.xhtml

We would like to be sure that your child's body responds to the vaccine as well as the children who received the second dose of the vaccine two months after the first dose. To check this, we will be looking at the antibodies that your child's body produces after he/she receives a vaccine. Antibodies are produced by your body after vaccination to protect you from infection. The information from testing your child's blood for antibodies will be very important when the vaccine is used to protect against Ebola in the future because it may not always be possible to give the second dose at exactly two months after the first dose of the vaccine.

If you are the parent or guardian of a child under 18 years of age and you agree to your child providing blood samples, you will need to sign separate documents for you and your child. If your child is aged 12 to 17 years old, he/she will also need to agree to provide blood samples himself/herself and sign a different document, called the immunogenicity assent form.

DOES YOUR CHILD HAVE TO GIVE BLOOD SAMPLES?

Your child does not have to provide any blood samples. If you agree to your child providing blood samples, we will ask you to sign this consent form. If you do not want your child to give any blood samples, this will not affect your child's participation in the TUJIOKOWE study.

WHAT WILL HAPPEN IF I AGREE TO MY CHILD GIVING BLOOD SAMPLES?

We will describe the blood collection process and answer any questions that you may have. If you would like a copy of the written information, we will give it to you. You will be asked to sign or put your fingerprint on this consent form. Putting your name or your fingerprint on the consent form means that you agree to your child providing blood samples, but you can change your mind at any time.

After your child has given his/her first blood sample , your child will follow the normal TUJIOKOWE study procedures to receive the second dose of vaccine. To have your child's second blood sample taken, you will need to bring your child to the study clinic for an extra visit 21 days after your child received the second injection of vaccine. If you agree to your child participating in the Immunogenicity Subset, then we will give your child some refreshments at the clinic.

WHAT DOES GIVING BLOOD INVOLVE?

A small amount of your child's blood will be collected from your child using a small clean needle. We will take blood before your child gets the second injection of vaccine and at 21 days after the second injection. To have your child's second blood sample taken your child will need to come to the study clinic for an extra visit 21 days after your child has received the second dose of vaccine. For children aged 6 to 17 years, we will collect approximately 5 mL (about 1 teaspoon) of blood each time. For young children aged 4 to 5 years, we will only collect 2.5 mL of blood each time.

As explained to you earlier, the blood samples your child gives will be used to test whether your child's body has produced antibodies in his/her blood against Ebola after vaccination. You

For peer review only - http://bmjopen.bmj.com/site/about/guidelines.xhtml

and your child will not receive the results of these tests because they are only for scientific research.

After we do our tests on your child's blood samples for the Immunogenicity Subset of the TUJIOKOWE study, we will destroy any remaining blood that has been collected from your child. We will not use your child's blood for any other purpose except the antibody tests that we have explained in this consent form. Your child's blood samples will not be sold.

Your child's blood samples will be labelled with a code and not his/her name. Other information, such as sex, age, or health history might also be shared with other investigators, but your child's name will not be shared.

WHAT ARE THE POSSIBLE RISKS OF GIVING A BLOOD SAMPLE?

Providing a blood sample is generally safe but occasionally people experience pain, bruising, bleeding, or (very rarely) infection from giving a blood sample. The medical team will provide care if your child experiences side-effects from the blood sampling. They are available if your child has any problems or if you have any questions.

WHAT ARE THE BENEFITS OF GIVING A BLOOD SAMPLE?

There is no direct benefit to your child for providing blood samples in the Immunogenicity Subset of the TUJIOKOWE study. Your agreement for your child to provide blood samples will help in the development of vaccines to prevent Ebola and, in the future, may help people in different parts of the world.

WHAT HAPPENS TO THE BLOOD SAMPLES COLLECTED FROM MY CHILD?

The blood samples collected from your child during the Immunogenicity Subset of the TUJIOKOWE study, will only be used to understand how the Ebola vaccine works when the second dose is given more than two months after the first dose.

The samples will be coded in a way that only limited study staff can link them to your child. Your child's blood samples will be tested in a laboratory in the United States. If any blood remains in DRC, it will be stored in the INRB laboratory in Goma. We will destroy all the samples in the United States and in Goma at the end of the study.

For peer review only - http://bmjopen.bmj.com/site/about/guidelines.xhtml

WHO CAN I TALK TO ABOUT MY CHILD GIVING BLOOD SAMPLES?

If you want to talk to someone about your child giving blood samples or if you think your child has been harmed by providing a blood sample, you can contact the following people:

1) The Principal Investigator responsible for this study

Professor Jean Jacques Muyembe<br>*Director General,* Institut National de Recherche Biomédicale, Kinshasa, DRC<br>Phone: 0898 949 289<br>Email: jjmuyembet@gmail.com<br><br>Local study representative<br>Dr Hugo Kavunga<br>Phone: 0823 875 153<br>Email: hugokavunga@gmail.com

2) The DRC ethics committees that approved this study

Professeur Félicien Munday<br>National Ethics Committee<br>Kinshasa-Gombe, DRC<br>Phone: 0998 419 816<br>Email: feli1munday@yahoo.fr
Professeur Willy Bongopasi<br>Comité d'éthique de l'école de santé publique<br>Université de Kinshasa<br>Phone: 0999 952 341<br>Email: bongopasi@gmail.com

If you have any questions about your child providing blood samples for the Immunogenicity Subset of the TUJIOKOWE study or about your child's rights, you may ask anyone on the study team at any time.

For peer review only
For peer review only - http://bmjopen.bmj.com/site/about/guidelines.xhtml

PARTICIPANT IMMUNOGENICITY SUBSET CONSENT FORM

Title: Immunogenicity Subset of the TUJIOKOWE Study

Principal Investigator of this study: Prof. JJ Muyembe

**Statements**	**Please sign or fingerprint each box**
I have read the information in this form about my child giving a blood sample at two visits for the Immunogenicity Subset of the TUJIOKOWE Study (or I have had this information explained to me by the study staff in a language that I understand). The purpose of providing blood samples and the procedures to give blood samples have been fully explained to me. I was able to ask questions and have all of these questions answered to my satisfaction.	
I understand that my child's participation is voluntary, that my child can withdraw consent to provide blood samples at any time without giving any reason, and that this will not affect my child's participation in the TUJIOKOWE Study.	

I am the parent or guardian of a participant aged 4 to 17 years and I agree for my child to provide blood samples during his/her participation in the TUJIOKOWE study.
(Please sign or put your fingerprint below)

		____/____/_____ *dd mon yyyy*
Printed name of the parent or guardian	Signature/fingerprint of the parent or guardian	Date

Printed name of the child participant (if aged 4-17)

For peer review only - http://bmjopen.bmj.com/site/about/guidelines.xhtml

		____/____/_____ *dd mon yyyy*
Printed name of investigator	Signature of investigator	Date

Complete next section if the parent/guardian is illiterate:

Witness to Consent Interview

I witnessed the consent interview for the Immunogenicity Subset of the TUJIOKOWE Study in this document. I attest that I have explained the study information accurately to the parent/guardian and was understood to the best of my knowledge by the parent/guardian, and that he/she has freely given their consent for his/her child to participate in my presence.

		____/____/_____ *dd mon yyyy*
Printed name of impartial witness	Signature of impartial witness	Date

Attach ID barcode label below:

For peer review only

Protocol for a phase 3 trial to evaluate effectiveness and safety of a heterologous, two-dose vaccine for Ebola virus disease in the Democratic Republic of the Congo

Appendix 5B

Immunogenicity Informed Consent Form for Parents in French, Version 1.1 (10 Aug 2020)

For peer review only

For peer review only - http://bmjopen.bmj.com/site/about/guidelines.xhtml

Évaluation de l'efficacité et de la sécurité d'emploi d'un vaccin préventif hétérologue à deux doses contre Ebola en République démocratique du Congo

Note d'information et formulaire de consentement éclairé pour le sous-groupe Immunogénicité de l'« étude TUJIOKOWE » destinés aux parents/tuteurs des participants pédiatriques

Protocole LSHTM : DRC-EB-001

Investigateur principal (IP) :
Professeur Jean Jacques Muyembe
Directeur général
Institut national de recherche biomédicale
Professeur de microbiologie, Faculté de médecine de l'Université de Kinshasa
Kinshasa Gombe, République démocratique du Congo (RDC)
Téléphone : 0898 949 289 ; Adresse électronique : jjmuyembet@gmail.com

Promoteur : London School of Hygiene & Tropical Medicine, Royaume-Uni

Centre : République démocratique du Congo

INTRODUCTION

L'« étude TUJIOKOWE » est une étude de recherche visant à déterminer si un nouveau vaccin à deux doses appelé « vaccin contre le virus Ebola de Janssen » peut protéger contre le virus Ebola et à vérifier si le vaccin est sans danger. L'étude TUJIOKOWE est mise en œuvre par le ministère de la Santé de la RDC par l'intermédiaire de l'Institut national de recherche biomédicale (INRB), Epicentre et la London School of Hygiene & Tropical Medicine.

Lorsque nous avons commencé l'étude TUJIOKOWE, nous avions prévu d'administrer la seconde dose du vaccin contre le virus Ebola à tous les participants 2 mois environ après la première dose. Certaines personnes vont maintenant recevoir la seconde dose du vaccin contre le virus Ebola plus tard que prévu. Cela s'explique par la nécessité de fermer temporairement les centres de l'étude en raison de l'épidémie de COVID-19. Nous voulons comprendre si les personnes qui reçoivent leur seconde dose de vaccin plus de 2 mois après présentent la même réponse immunitaire que les personnes qui l'ont reçue 2 mois après la première dose. À cet effet, nous allons devoir prélever des échantillons de sang auprès de 50 adultes, 25 adolescents et 25 enfants environ participant à l'étude TUJIOKOWE, qui ont reçu la seconde dose du vaccin contre le virus Ebola après juillet 2020. Nous demandons à votre enfant de participer à cette petite étude (que nous appelons « sous-groupe Immunogénicité ») et de fournir deux échantillons de sang, car votre enfant va recevoir la seconde dose du vaccin après juillet 2020. Si vous acceptez que votre enfant participe à l'étude, un échantillon de sang lui sera prélevé

For peer review only - http://bmjopen.bmj.com/site/about/guidelines.xhtml
For peer review only

avant l'administration de la seconde dose du vaccin contre le virus Ebola et le second échantillon de sang sera prélevé 21 jours plus tard.

Nous voulons nous assurer que l'organisme de votre enfant réagit au vaccin aussi bien que celui des enfants qui ont reçu la seconde dose du vaccin 2 mois après la première dose. À cet effet, nous allons rechercher les anticorps que produit l'organisme de votre enfant après l'administration du vaccin. Les anticorps sont produits par l'organisme après la vaccination pour vous protéger contre l'infection. Les informations obtenues en analysant le sang de votre enfant pour rechercher la présence d'anticorps seront très importantes lorsque le vaccin sera utilisé pour protéger contre le virus Ebola à l'avenir, car il ne sera peut-être pas toujours possible d'administrer la seconde dose du vaccin 2 mois exactement après la première dose.

Si vous êtes le parent ou le tuteur d'un enfant de moins de 18 ans et si vous acceptez qu'il fournisse les échantillons de sang, vous devrez signer des documents distincts pour vous et votre enfant. Si votre enfant est âgé de 12 à 17 ans, il devra également accepter de lui-même de fournir les échantillons de sang et signer un autre document, appelé « formulaire d'assentiment » pour le sous-groupe Immunogénicité.

VOTRE ENFANT EST-IL OBLIGÉ DE DONNER DES ÉCHANTILLONS DE SANG ?

Votre enfant n'est pas obligé de fournir des échantillons de sang. Si vous acceptez que votre enfant fournisse des échantillons de sang, nous vous inviterons à signer ce formulaire de consentement. Si vous ne souhaitez pas que votre enfant donne des échantillons de sang, cela ne remettra pas en cause sa participation à l'étude TUJIOKOWE.

QUE SE PASSERA-T-IL SI J'ACCEPTE QUE MON ENFANT DONNE DES ÉCHANTILLONS DE SANG ?

Nous allons décrire les modalités de prise de sang et répondre à toutes les questions que vous pourriez avoir. Si vous souhaitez une copie de cette note d'information, nous vous la remettrons. Nous vous inviterons à signer ce formulaire de consentement ou à y apposer votre empreinte digitale. En inscrivant votre nom ou en apposant votre empreinte digitale sur le formulaire de consentement, vous acceptez que votre enfant fournisse des échantillons de sang, mais vous pourrez changer d'avis à tout moment.

Une fois que votre enfant aura donné son premier échantillon de sang, il suivra les procédures habituelles de l'étude TUJIOKOWE pour recevoir la seconde dose de vaccin. Pour le prélèvement du second échantillon de sang de votre enfant, vous devrez amener votre enfant au centre de l'étude pour une visite supplémentaire 21 jours après l'administration de la seconde injection du vaccin à votre enfant. Si vous acceptez que votre enfant participe au sous-groupe Immunogénicité, chaque fois qu'il donnera son sang, nous lui donnerons des rafraîchissements.

For peer review only - http://bmjopen.bmj.com/site/about/guidelines.xhtml

EN QUOI CONSISTE LE PRÉLÈVEMENT DE SANG ?

Une petite quantité de sang sera prélevée à votre enfant à l'aide d'une petite aiguille propre. Nous lui prélèverons un échantillon de sang avant qu'il reçoive la seconde injection du vaccin et 21 jours après la seconde injection. Pour le prélèvement du second échantillon de sang de votre enfant, il devra venir effectuer une visite supplémentaire au centre de l'étude 21 jours après l'administration de la seconde dose de vaccin. Pour les enfants âgés de 6 à 17 ans, nous prélèverons 5 ml environ (soit 1 cuillère à café environ) de sang à chaque fois. Pour les jeunes enfants âgés de 4 à 5 ans, nous ne prélèverons que 2,5 ml de sang à chaque fois.

Comme indiqué précédemment, les échantillons de sang que votre enfant donnera serviront à vérifier si son organisme a produit des anticorps dans son sang contre le virus Ebola après la vaccination. Vous et votre enfant ne recevrez pas les résultats de ces analyses, car ils sont destinés uniquement à la recherche scientifique.

Après avoir réalisé nos analyses sur les échantillons de sang de votre enfant pour le sous-groupe Immunogénicité de l'étude TUJIOKOWE, nous détruirons tous les échantillons de sang restants prélevés à votre enfant. Nous n'utiliserons pas les échantillons de sang de votre enfant à d'autres fins que les analyses de détection d'anticorps mentionnés dans ce formulaire de consentement. Les échantillons de sang de votre enfant ne seront pas vendus.

Les échantillons de sang de votre enfant seront étiquetés avec un code et non avec son nom. D'autres informations, comme le sexe, l'âge ou les antécédents médicaux, pourront également être partagées avec d'autres investigateurs, mais le nom de votre enfant ne sera pas communiqué.

QUELS SONT LES RISQUES ÉVENTUELS LIÉS AU DON D'UN ÉCHANTILLON DE SANG ?

Le prélèvement d'un échantillon de sang est généralement sans danger, mais il arrive que des personnes ressentent des douleurs ou présentent un bleu, un saignement ou (dans de très rares cas) une infection après un prélèvement de sang. Si votre enfant présente des effets secondaires après la prise de sang, l'équipe médicale lui apportera les soins nécessaires. L'équipe est disponible à tout moment si votre enfant a des problèmes ou des questions.

QUELS SONT LES BÉNÉFICES LIÉS AU DON D'UN ÉCHANTILLON DE SANG ?

Il n'y a aucun bénéfice direct pour votre enfant à fournir des échantillons de sang dans le cadre du sous-groupe Immunogénicité de l'étude TUJIOKOWE. Le consentement de votre enfant à fournir des échantillons de sang contribuera au développement de vaccins pour prévenir le virus Ebola et, à l'avenir, pourrait aider des personnes dans différentes régions du monde.

QU'ADVIENDRA-IL DES ÉCHANTILLONS DE SANG PRÉLEVÉS À MON ENFANT ?

Les échantillons de sang prélevés à votre enfant dans le cadre du sous-groupe Immunogénicité de l'étude TUJIOKOWE ne seront utilisés que pour comprendre l'efficacité du vaccin contre le virus Ebola lorsque la seconde dose est administrée plus de 2 mois après la première dose.

For peer review only - http://bmjopen.bmj.com/site/about/guidelines.xhtml

Les échantillons seront codés de manière à limiter le nombre de membres du personnel de l'étude qui peut les relier à votre enfant. Les échantillons de sang de votre enfant seront analysés dans un laboratoire aux États-Unis. S'il reste des échantillons de sang en RDC, ils seront conservés dans le laboratoire de l'INRB à Goma. Nous détruirons tous les échantillons conservés aux États-Unis et à Goma à la fin de l'étude.

À QUI PUIS-JE M'ADRESSER CONCERNANT LE PRÉLÈVEMENT D'ÉCHANTILLONS DE SANG À ENFANT ?

Si vous souhaitez parler à quelqu'un au sujet du prélèvement d'échantillons de sang à votre enfant ou si vous pensez que votre enfant a subi un préjudice en fournissant un échantillon de sang, vous pouvez contacter les personnes suivantes :

1) Investigateur principal responsable de cette étude

Professeur Jean-Jacques Muyembe<br>*Directeur général*, Institut national de recherche biomédicale, Kinshasa, RDC<br>Téléphone : 0898 949 289<br>Adresse électronique : jjmuyembet@gmail.com<br><br>Représentant locale de l'étude<br>Dr Hugo Kavunga<br>Téléphone : 0823 875 153<br>Adresse électronique : hugokavunga@gmail.com

2) Comités d'éthique de la RDC ayant approuvé cette étude

Professeur Félicien Munday<br>Comité national d'éthique et de la santé<br>Kinshasa-Gombe, RDC<br>Téléphone : 0998 419 816<br>Adresse électronique : feli1munday@yahoo.fr
Professeur Willy Bongopasi<br>Comité d'éthique de l'école de santé publique<br>Université de Kinshasa<br>Téléphone: 0999 952 341<br>Adresse électronique : bongopasi@gmail.com

Si vous avez des questions sur le prélèvement d'échantillons de sang à votre enfant pour le sous-groupe Immunogénicité de l'étude TUJIOKOWE ou sur les droits de votre enfant, vous pouvez les poser à tout moment à n'importe quel membre de l'équipe de l'étude.

For peer review only - http://bmjopen.bmj.com/site/about/guidelines.xhtml

FORMULAIRE DE CONSENTEMENT DES PARTICIPANTS POUR LE SOUS-GROUPE IMMUNOGÉNICITÉ

Titre : Sous-groupe Immunogénicité de l'étude TUJIOKOWE

Investigateur principal de cette étude : Prof. JJ Muyembe

**Déclarations**	**Signez ou apposez votre empreinte digitale dans chaque case**
J'ai lu les informations contenues dans ce formulaire sur le prélèvement d'un échantillon de sang à mon enfant lors de deux visites pour le sous-groupe Immunogénicité de l'étude TUJIOKOWE (ou le personnel de l'étude m'a expliqué ces informations avec des mots que je comprends). L'objectif du prélèvement d'échantillons de sang et les procédures correspondantes m'ont été expliqués en détail. J'ai pu poser des questions et j'ai reçu des réponses satisfaisantes à toutes mes questions.	
Je comprends que la participation de mon enfant est volontaire, que mon enfant peut retirer son consentement à fournir des échantillons de sang à tout moment sans avoir à se justifier et que cela ne remettra pas en cause sa participation à l'étude TUJIOKOWE.	

Je suis le parent ou le tuteur d'un participant âgé de 4 à 17 ans et j'accepte que mon enfant fournisse des échantillons de sang pendant sa participation à l'étude TUJIOKOWE. (Signez ou apposez votre empreinte digitale ci-dessous)

		____/____/_____ *jj mmm aaaa*
Nom du parent ou tuteur (en caractères d'imprimerie)	Signature/Empreinte digitale du parent ou tuteur	Date

Nom de l'enfant participant (si âgé de 4 à 17 ans ; en caractères d'imprimerie)

For peer review only - http://bmjopen.bmj.com/site/about/guidelines.xhtml

		____/____/_____ *jj mmm aaaa*
Nom de l'investigateur (en caractères d'imprimerie)	Signature de l'investigateur	Date

Si le parent/tuteur est analphabète, remplissez cette section :

Témoin de l'entretien de consentement

J'ai assisté à l'entretien de consentement pour le sous-groupe Immunogénicité de l'étude TUJIOKOWE décrite dans ce document. J'atteste avoir expliqué avec exactitude les informations sur l'étude au parent/tuteur et que, à ma connaissance, cette personne les a comprises et a donné librement son consentement pour que son enfant participe, en ma présence.

		____/____/_____ *jj mmm aaaa*
Nom du témoin impartial (en caractères d'imprimerie)	Signature du témoin impartial	Date

Apposez l'étiquette d'identification par code à barres ci-dessous :

For peer review only

For peer review only - http://bmjopen.bmj.com/site/about/guidelines.xhtml

Protocol for a phase 3 trial to evaluate effectiveness and safety of a heterologous, two-dose vaccine for Ebola virus disease in the Democratic Republic of the Congo

Appendix 6

Data and Safety Monitoring Board (DSMB)

The following will be invited to participate on the Data and Safety Monitoring Board (DSMB) of this study. For detailed description of the DSMB responsibilities and activities, refer to the latest DSMB Charter.

Point of contact: Yazdan Yazdanpanah

Yazdan Yazdanpanah<br>Adult Clinician<br>yazdan.yazdanpanah@aphp.fr	Matthias Egger<br>Methodologist (DSMB Statistician)<br>matthias.egger@ispm.unibe.ch
Ebunoluwa Adejuyigbe<br>Paediatrician<br>ebunadejuyigbe@hotmail.com	Florian Marks<br>Vaccinologist/Epidemiologist<br>fmarks@ivi.int
Benoit Kebela Ilunga<br>Epidemiologist<br>kebelailunga@gmail.com	Tamara Giles-Vernick<br>Social Scientist<br>tamara.giles-vernick@pasteur.fr
Oumou Younoussa Bah Sow<br>Ethicist; President of the National Committee of Ethics for Health Research (CNERS)<br>oumou45@yahoo.fr	Marie Onyamboko<br>Epidemiologist; Kinshasa School of Public Health<br>akatshimarie@yahoo.fr

For peer review only

Trial Steering Committee (TSC)

The TSC is responsible for conducting the clinical study and for solving strategic problems that require high-level interventions.

Point of Contact: Richard Hatchett

Richard Hatchett/Gerald Voss<br>Coalition for Epidemic Preparedness Innovations<br>Richard.Hatchett@cepi.net<br>gerald.voss@cepi.net	Sylvain Yuma<br>Ministère de la Santé, DRC<br>sylvainyuma@gmail.com
Daniel Bausch<br>UK Public Health Rapid Support Team/LSHTM<br>Daniel.Bausch@phe.gov.uk	Jean-Jacques Muyembe-Tamfum (and Steve Ahuka)<br>Institut National de Recherche Biomédicale, DRC<br>jjmuyembet@gmail.com (amstev04@yahoo.fr)
Mike Ryan/Ana Maria Henao-Restrepo<br>World Health Organization<br>ryanm@who.int; henaorestrepoa@who.int	Jeremy Farrar/Josie Golding<br>Wellcome Trust<br>J.Farrar@wellcome.ac.uk

	J.Golding@wellcome.ac.uk
Peter Piot/Deborah Watson-Jones<br>London School of Hygiene & Tropical Medicine<br>Peter.Piot@lshtm.ac.uk;<br>deborah.watson-jones@lshtm.ac.uk	Johan Van Hoof (and Macaya Douoguih)<br>Janssen Pharmaceuticals R&D<br>JVHOOF1@its.jnj.com (MDouogui@its.jnj.com)
Rebecca F Grais<br>Director of Research<br>Epicentre MSF, Paris, France<br>rebecca.grais@epicentre.msf.org	Isabelle Defourny/Clair Mills<br>MSF Operational Centre Paris<br>Médecins Sans Frontières, Paris, France<br>Isabelle.defourny@paris.msf.org;<br>Clair.Mills@paris.msf.org
Robert Kanwagi<br>World Vision<br>Robert.kanwagi@wveu.org	

For peer review only

Protocol for a phase 3 trial to evaluate effectiveness and safety of a heterologous, two-dose vaccine for Ebola virus disease in the Democratic Republic of the Congo

Appendix 8

<u>Sponsor and Funders Responsibilities</u>

Funders CEPI, Wellcome and DFID advised on the study design and protocol.

	Sponsor	Funders
Study design	A/R	R
Data collection	A/R	I
Data management	A/R	I
Data analysis	A/R	I
Data interpretation	A/R	I
Report writing	A/R	C
Report submission for publication	A/R	C

A = Accountable
R = Responsible
C = Contributing
I = Informed

For peer review only

Reporting checklist for protocol of a clinical trial.

Based on the SPIRIT guidelines.

Instructions to authors

Complete this checklist by entering the page numbers from your manuscript where readers will find each of the items listed below.

Your article may not currently address all the items on the checklist. Please modify your text to include the missing information. If you are certain that an item does not apply, please write "n/a" and provide a short explanation.

Upload your completed checklist as an extra file when you submit to a journal.

In your methods section, say that you used the SPIRITreporting guidelines, and cite them as:

Chan A-W, Tetzlaff JM, Gøtzsche PC, Altman DG, Mann H, Berlin J, Dickersin K, Hróbjartsson A, Schulz KF, Parulekar WR, Krleža-Jerić K, Laupacis A, Moher D. SPIRIT 2013 Explanation and Elaboration: Guidance for protocols of clinical trials. BMJ. 2013;346:e7586

		Reporting Item	Page Number
**Administrative information**			
Title	#1	Descriptive title identifying the study design, population, interventions, and, if applicable, trial acronym	5
Trial registration	#2a	Trial identifier and registry name. If not yet registered, name of intended registry	2
Trial registration: data set	#2b	All items from the World Health Organization Trial Registration Data Set	21, Table 1
Protocol version	#3	Date and version identifier	17
Funding	#4	Sources and types of financial, material, and other support	20
Roles and responsibilities: contributorship	#5a	Names, affiliations, and roles of protocol contributors	18-19

For peer review only

Roles and responsibilities: sponsor contact information	#5b	Name and contact information for the trial sponsor	21, Table 1
Roles and responsibilities: sponsor and funder	#5c	Role of study sponsor and funders, if any, in study design; collection, management, analysis, and interpretation of data; writing of the report; and the decision to submit the report for publication, including whether they will have ultimate authority over any of these activities	Appendix 7
Roles and responsibilities: committees	#5d	Composition, roles, and responsibilities of the coordinating centre, steering committee, endpoint adjudication committee, data management team, and other individuals or groups overseeing the trial, if applicable (see Item 21a for data monitoring committee)	16, Appendix 6
**Introduction**			
Background and rationale	#6a	Description of research question and justification for undertaking the trial, including summary of relevant studies (published and unpublished) examining benefits and harms for each intervention	4-6
Background and rationale: choice of comparators	#6b	Explanation for choice of comparators	6
Objectives	#7	Specific objectives or hypotheses	5
Trial design	#8	Description of trial design including type of trial (eg, parallel group, crossover, factorial, single group), allocation ratio, and framework (eg, superiority, equivalence, non-inferiority, exploratory)	6
**Methods: Participants, interventions, and outcomes**			
Study setting	#9	Description of study settings (eg, community clinic, academic hospital) and list of countries where data will be collected. Reference to where list of study sites can be obtained	7

For peer review only

Eligibility criteria	#10	Inclusion and exclusion criteria for participants. If applicable, eligibility criteria for study centres and individuals who will perform the interventions (eg, surgeons, psychotherapists)	8, 24, Box 1
Interventions: description	#11a	Interventions for each group with sufficient detail to allow replication, including how and when they will be administered	5
Interventions: modifications	#11b	Criteria for discontinuing or modifying allocated interventions for a given trial participant (eg, drug dose change in response to harms, participant request, or improving / worsening disease)	10
Interventions: adherance	#11c	Strategies to improve adherence to intervention protocols, and any procedures for monitoring adherence (eg, drug tablet return; laboratory tests)	12,13
Interventions: concomitant care	#11d	Relevant concomitant care and interventions that are permitted or prohibited during the trial	24, Box 1
Outcomes	#12	Primary, secondary, and other outcomes, including the specific measurement variable (eg, systolic blood pressure), analysis metric (eg, change from baseline, final value, time to event), method of aggregation (eg, median, proportion), and time point for each outcome. Explanation of the clinical relevance of chosen efficacy and harm outcomes is strongly recommended	13-15
Participant timeline	#13	Time schedule of enrolment, interventions (including any run-ins and washouts), assessments, and visits for participants. A schematic diagram is highly recommended (see Figure)	10-12, 23, Table 2
Sample size	#14	Estimated number of participants needed to achieve study objectives and how it was determined, including clinical and statistical assumptions supporting any sample size calculations	7, 8, 25, Table 3
Recruitment	#15	Strategies for achieving adequate participant enrolment to reach target sample size	8, 9
**Methods: Assignment of interventions (for controlled trials)**			

For peer review only

Allocation: sequence generation	#16a	Method of generating the allocation sequence (eg, computer-generated random numbers), and list of any factors for stratification. To reduce predictability of a random sequence, details of any planned restriction (eg, blocking) should be provided in a separate document that is unavailable to those who enrol participants or assign interventions	N/A
Allocation concealment mechanism	#16b	Mechanism of implementing the allocation sequence (eg, central telephone; sequentially numbered, opaque, sealed envelopes), describing any steps to conceal the sequence until interventions are assigned	N/A
Allocation: implementation	#16c	Who will generate the allocation sequence, who will enrol participants, and who will assign participants to interventions	N/A
Blinding (masking)	#17a	Who will be blinded after assignment to interventions (eg, trial participants, care providers, outcome assessors, data analysts), and how	N/A
Blinding (masking): emergency unblinding	#17b	If blinded, circumstances under which unblinding is permissible, and procedure for revealing a participant's allocated intervention during the trial	N/A
**Methods: Data collection, management, and analysis**			
Data collection plan	#18a	Plans for assessment and collection of outcome, baseline, and other trial data, including any related processes to promote data quality (eg, duplicate measurements, training of assessors) and a description of study instruments (eg, questionnaires, laboratory tests) along with their reliability and validity, if known. Reference to where data collection forms can be found, if not in the protocol	14, 17
Data collection plan: retention	#18b	Plans to promote participant retention and complete follow-up, including list of any outcome data to be collected for participants who discontinue or deviate from intervention protocols	9, 17
Data management	#19	Plans for data entry, coding, security, and storage, including any related processes to promote data quality (eg, double data	14

For peer review only

		entry; range checks for data values). Reference to where details of data management procedures can be found, if not in the protocol	
Statistics: outcomes	#20a	Statistical methods for analysing primary and secondary outcomes. Reference to where other details of the statistical analysis plan can be found, if not in the protocol	15
Statistics: additional analyses	#20b	Methods for any additional analyses (eg, subgroup and adjusted analyses)	16
Statistics: analysis population and missing data	#20c	Definition of analysis population relating to protocol non-adherence (eg, as randomised analysis), and any statistical methods to handle missing data (eg, multiple imputation)	16
**Methods: Monitoring**			
Data monitoring: formal committee	#21a	Composition of data monitoring committee (DMC); summary of its role and reporting structure; statement of whether it is independent from the sponsor and competing interests; and reference to where further details about its charter can be found, if not in the protocol. Alternatively, an explanation of why a DMC is not needed	16, Appendix 6
Data monitoring: interim analysis	#21b	Description of any interim analyses and stopping guidelines, including who will have access to these interim results and make the final decision to terminate the trial	N/A
Harms	#22	Plans for collecting, assessing, reporting, and managing solicited and spontaneously reported adverse events and other unintended effects of trial interventions or trial conduct	11-12, 23, Table 2
Auditing	#23	Frequency and procedures for auditing trial conduct, if any, and whether the process will be independent from investigators and the sponsor	16
**Ethics and dissemination**			
Research ethics approval	#24	Plans for seeking research ethics committee / institutional review board (REC / IRB) approval	17
Protocol amendments	#25	Plans for communicating important protocol modifications (eg, changes to eligibility criteria, outcomes, analyses) to	10, 17

For peer review only

		relevant parties (eg, investigators, REC / IRBs, trial participants, trial registries, journals, regulators)	
Consent or assent	#26a	Who will obtain informed consent or assent from potential trial participants or authorised surrogates, and how (see Item 32)	9
Consent or assent: ancillary studies	#26b	Additional consent provisions for collection and use of participant data and biological specimens in ancillary studies, if applicable	10-12
Confidentiality	#27	How personal information about potential and enrolled participants will be collected, shared, and maintained in order to protect confidentiality before, during, and after the trial	13,14
Declaration of interests	#28	Financial and other competing interests for principal investigators for the overall trial and each study site	20
Data access	#29	Statement of who will have access to the final trial dataset, and disclosure of contractual agreements that limit such access for investigators	17
Ancillary and post trial care	#30	Provisions, if any, for ancillary and post-trial care, and for compensation to those who suffer harm from trial participation	12
Dissemination policy: trial results	#31a	Plans for investigators and sponsor to communicate trial results to participants, healthcare professionals, the public, and other relevant groups (eg, via publication, reporting in results databases, or other data sharing arrangements), including any publication restrictions	17
Dissemination policy: authorship	#31b	Authorship eligibility guidelines and any intended use of professional writers	17
Dissemination policy: reproducible research	#31c	Plans, if any, for granting public access to the full protocol, participant-level dataset, and statistical code	17
**Appendices**			
Informed consent materials	#32	Model consent form and other related documentation given to participants and authorised surrogates	Appendices 1-5
Biological specimens	#33	Plans for collection, laboratory evaluation, and storage of biological specimens for genetic or molecular analysis in the	10-11

For peer review only

current trial and for future use in ancillary studies, if applicable

None The SPIRIT Explanation and Elaboration paper is distributed under the terms of the Creative Commons Attribution License CC-BY-NC. This checklist can be completed online using https://www.goodreports.org/, a tool made by the EQUATOR Network in collaboration with Penelope.aib

For peer review only

BMJ Open

Protocol for a phase 3 trial to evaluate the effectiveness and safety of a heterologous, two-dose vaccine for Ebola virus disease in the Democratic Republic of the Congo

Journal:	*BMJ Open*
Manuscript ID	bmjopen-2021-055596.R1
Article Type:	Protocol
Date Submitted by the Author:	14-Dec-2021
Complete List of Authors:	Watson-Jones, Deborah; London School of Hygiene & Tropical Medicine; Mwanza Intervention Trials Unit<br>Kavunga-Membo, Hugo; L'Institut National de Recherche Biomédicale<br>Grais, RF; Epicentre<br>Ahuka, Steve; L'Institut National de Recherche Biomédicale<br>Roberts, Natalie; MSF<br>Edmunds, John; London School of Hygiene & Tropical Medicine<br>Choi, Edward; London School of Hygiene & Tropical Medicine, Department of Clinical Research<br>Roberts, Chrissy; London School of Hygiene & Tropical Medicine<br>Edwards, Tansy; London School of Hygiene & Tropical Medicine,<br>Camacho, Anton; Epicentre<br>Lees, Shelley; London School of Hygiene & Tropical Medicine<br>Leyssen, Maarten; Janssen Vaccines and Prevention BV<br>Spiessens, Bart; Janssen Vaccines and Prevention BV<br>Luhn, Kerstin; Janssen Vaccines and Prevention BV<br>Douoguih, Macaya; Janssen Vaccines and Prevention BV<br>Hatchett, Richard; Coalition for Epidemic Preparedness Innovations<br>Bausch, Daniel; London School of Hygiene & Tropical Medicine; Public Health England, UK Public Health Rapid Support Team<br>Muyembe, Jean-Jacques; L'Institut National de Recherche Biomédicale
**Primary Subject Heading**:	Infectious diseases
Secondary Subject Heading:	Immunology (including allergy), Public health, Research methods, Global health
Keywords:	PUBLIC HEALTH, VIROLOGY, IMMUNOLOGY, INFECTIOUS DISEASES, TROPICAL MEDICINE

SCHOLARONE™
Manuscripts

Protocol for a phase 3 trial to evaluate the effectiveness and safety of a heterologous, two-dose vaccine for Ebola virus disease in the Democratic Republic of the Congo

Deborah Watson-Jones[1,2], Hugo Kavunga-Membo[3], Rebecca Grais[4], Steve Ahuka[3], Natalie Roberts[5], W. John Edmunds[1], Edward M. Choi[1], Chrissy H. Roberts[1], Tansy Edwards[1], Anton Camacho[4], Shelley Lees[1], Maarten Leyssen[6], Bart Spiessens[6], Kerstin Luhn[6], Macaya Douoguih[6], Richard Hatchett[7], Daniel G. Bausch[1,8], Jean Jacques Muyembe[3], DRC-EB-001 protocol writing team[9].

Correspondence to Deborah Watson-Jones; Deborah.watson-jones@lshtm.ac.uk

Keywords: Public health, virology, immunology, infectious diseases, tropical medicine

Word Count: 5083

For peer review only

For peer review only - http://bmjopen.bmj.com/site/about/guidelines.xhtml

Abstract

Introduction

Ebola virus disease (EVD) continue to be a significant public health problem in sub-Saharan Africa, especially in the Democratic Republic of the Congo (DRC). Large-scale vaccination during outbreaks may reduce virus transmission. We established a large population-based clinical trial of a heterologous, two-dose prophylactic vaccine during an outbreak in eastern DRC to determine vaccine effectiveness.

Methods and analysis

This open label, non-randomised population-based trial enrolled eligible adults and children aged one year and above. Participants were offered the two-dose candidate EVD vaccine regimen VAC52150 (Ad26.ZEBOV, MVA-BN-Filo), with the doses being given 56 days apart. After vaccination, serious adverse events (SAEs) were passively recorded until 1 month post-dose 2. 1000 safety subset participants were telephoned at 1 month post-dose 2 to collect SAEs. 500 pregnancy subset participants are contacted to collect SAEs at D7 and D21 post-dose 1 and at D7, 1 month, 3 months and 6 months post-dose 2, unless delivery is before these time points. The first 100 infants born to these women are given a clinical examination 3 months post-delivery. Due to COVID-19 and temporary suspension of dose 2 vaccinations, 50 paediatric and 50 adult participants were enrolled into an immunogenicity subset to examine immune responses following a delayed second dose. Samples collected pre-dose 2 and at 21 days post-dose 2 will be tested using the EBOV GP Filovirus Animal Nonclinical Group (FANG) enzyme-linked immunosorbent assay. For qualitative research, in-depth interviews and focus group discussions are being conducted with participants or parents/care-providers of paediatric participants.

Ethics and dissemination

Approved by Comité National d'Ethique et de la Santé du Ministère de la santé de RDC, Comité d'Ethique de l'Ecole de Santé Publique de l'Université de Kinshasa, the LSHTM Ethics Committee and the MSF Ethics Review Board. Findings will be presented to stakeholders and conferences. Study data will be made available for open access.

Trial registration number

For peer review only - http://bmjopen.bmj.com/site/about/guidelines.xhtml

NCT04152486

STRENGTHS AND LIMITATIONS OF THIS STUDY

- This is the first large-scale trial designed to measure effectiveness of a two-dose Ebola vaccine through open label delivery to general populations in an Ebola-affected area.
- This is the first clinical trial to administer this vaccine regimen to pregnant women.
- The pragmatic design facilitated trial delivery during an active Ebola epidemic where the future location of new cases is uncertain.
- A test negative case control analysis to estimate vaccine effectiveness in a situation where a randomised controlled trial with a control arm was not possible.
- A non-randomised design is a limitation of this study.

For peer review only

For peer review only - http://bmjopen.bmj.com/site/about/guidelines.xhtml

INTRODUCTION

Background and rationale

Ebola virus disease (EVD) is caused by Ebola viruses (EBOV) and has a case fatality rate ranging from 30-90%.[1] Bats are thought to be the reservoir, with human infection resulting from contact with excrement or saliva from bats or other intermediate animal hosts.[2-5]. Large outbreaks are fueled almost exclusively by subsequent human-to-human transmission.[2,6,7]

Vaccination is now a proven preventative measure for EVD control. During the 2013-16 EVD outbreak in West Africa, a single dose of the rVSV-ZEBOV-GP vaccine (ERVEBO®, Merck & Co., Inc.) given through a phase 3 cluster-randomised, ring-vaccination trial in Guinea showed 100% protective efficacy against EVD between day 10 and day 31 post-randomisation.[8] In 2016, the World Health Organization (WHO) Strategic Advisory Group of Experts on Immunization (SAGE) recommended the rapid deployment of rVSV-ZEBOV-GP for EVD outbreaks under an expanded access (compassionate use) protocol and the vaccine was licensed by the Democratic Republic of the Congo (DRC), Burundi, Ghana and Zambia in February 2020.[9,10]

Since the discovery of EBOV in 1976, the DRC has recorded 12 outbreaks. On 1 August 2018, the tenth EVD outbreak was declared in North Kivu Province.[11] The outbreak progressively expanded across a large, highly populated area, characterized in recent years by civil unrest, armed militia groups, internally displaced persons and a substantial United Nations peacekeeping mission.[12] The outbreak response focused on strategies that were previously adopted for the control of EVD outbreaks but was periodically hindered by attacks on Ebola Treatment Centres (ETCs) and on response workers. New cases continued to emerge in North Kivu and neighbouring provinces, prompting consideration of additional vaccine programmes that might assist in preventing the spread of this infection to unaffected communities. In early 2019, WHO conducted an evaluation of the suitability of candidate EVD vaccines for further clinical study and recommended several vaccines including VAC52150 (Ad26.ZEBOV, MVA-BN-Filo), a heterologous two-dose prophylactic vaccine manufactured by Janssen Vaccines and Prevention B.V.[13]

For peer review only - http://bmjopen.bmj.com/site/about/guidelines.xhtml

Objectives

Following the WHO evaluation, we are conducting an open label, non-randomised, population-based trial in North Kivu Province. This trial is named "Evaluation of a heterologous, two-dose preventive Ebola vaccine for effectiveness and safety in the Democratic Republic of the Congo". The protocol number is DRC-EB-001. The primary objective is to estimate the vaccine effectiveness (VE) of Ad26.ZEBOV, MVA-BN-Filo for prevention of EVD in adults and children aged 1 year or above. Secondary objectives are to: (1) assess the safety of Ad26.ZEBOV, MVA-BN-Filo, (2) estimate the coverage of dose 1 and dose 2 of Ad26.ZEBOV, MVA-BN-Filo overall and in different target groups (by gender, age and location), and (3) to explore the knowledge and perceptions of persons eligible for large-scale delivery of a preventative EVD vaccine with a two-dose vaccine strategy. An exploratory objective is assessing immunogenicity of Ad26.ZEBOV, MVA-BN-Filo in a subset of participants who received dose 2 outside the recommended interval between doses 1 and 2. This is the first study providing Ad26.ZEBOV, MVA-BN-Filo to pregnant women (Table 1).

METHODS

The vaccine

The vaccination regimen comprised two vaccine candidates, both given as a 0.5ml intramuscular injection in the upper section of the deltoid muscle or in the thigh in the case of young children:

1) Ad26.ZEBOV: a monovalent vaccine expressing the full-length glycoprotein (GP) from EBOV Mayinga, that is produced in the human cell line PER.C6®, $5x10^{10}$ viral particles and given at day zero
2) MVA-mBN226B, or Modified Vaccinia Ankara (MVA)-BN®-Filo: a multivalent vaccine expressing the GP of EBOV (100% homologous with the GP expressed by Ad26.ZEBOV), Sudan, and Marburg Musoke viruses; and the nucleoprotein of Taï Forest virus, $1x10^{8}$ infectious units given at day 56 (-14 day +28 day).

This regimen has been evaluated for immunogenicity and safety in 11 clinical trials in the United Kingdom, United States, and East and West Africa, including in previously EVD-affected countries.[14-21] When administered in a 0, 56 day schedule in Phase 2 and 3 studies, geometric mean concentrations of immunoglobulin G (IgG) binding antibody to the EBOV

For peer review only - http://bmjopen.bmj.com/site/about/guidelines.xhtml

glycoprotein (GP) measured by the Filovirus Animal Non-Clinical Group (FANG) Enzyme-Linked Immunosorbent Assay (ELISA) 21 days post-dose-2 were 3,810-11,790 EU/mL in healthy participants. The FANG ELISA assay has been validated for use in human sera.[22] Unblinded safety data from 2,390 adults showed only mild-to-moderate adverse events of short duration with no sequelae.[17] No safety concerns were raised in HIV-infected individuals.[18] In 649 children, the vaccine was highly immunogenic and had similar safety profile as adults, with no Suspected Unexpected Serious Adverse Reactions.[19] The European Medicines Agency granted marketing authorization for Ad26.ZEBOV, MVA-BN-Filo on 1 July 2020.[20,21]

For this study, vaccine was donated by Janssen Vaccines and Prevention B.V.. Vials were stored and shipped according to Good Manufacturing Practice guidelines with maintenance of the cold chain from Belgium to the vaccine depot in Goma, DRC, and onto the vaccination clinics.

Trial design

The protocol development team and investigators considered various trial designs that would have allow estimation of vaccine efficacy or effectiveness in the context of an epidemic and vaccination activities with the rVSV-ZEBOV-GP vaccine. The value of placebo-controlled randomised controlled trial (RCT) designs was recognized but, given the ongoing EVD outbreak at the time of initiation of the study and taking into account socio-cultural sensitivities and civil instability in the region, an RCT was not considered feasible or ethical. Rather, we adopted a test-negative case-control design, in which vaccine effectiveness (VE) is estimated by comparing the history of vaccination in cases (test positives) compared to that in controls (test negatives), among individuals presenting with suspected EVD, with cases and controls defined retrospectively after EVD test results were confirmed. The test-negative design allows evaluation of VE in the general population without randomization, and has been proposed as a valid approach for evaluation of EVD vaccines when RCTs are not feasible.[23,24] The validity of this approach has been demonstrated by comparing results from test-negative case-control studies with those from randomized clinical trials of influenza and rotavirus vaccines. The two type of study yielding similar VE estimates.[25,26]

For peer review only

Trial setting

This trial was planned to start in November 2019 and to end in February 2022. After discussion with local authorities, we initiated the trial in two administrative health areas in Goma, the provincial capital of North Kivu Province (population 634,197 in 2020). The choice of these locations was determined based on their potential risk for EVD transmission since there were strong ethnic, family and business links between these two health areas and the epidemic epicentres of Beni and Butembo, with frequent travel between the communities. Provincial administrative and health authorities provided data on the number of residents of these areas as well as the number of individuals working in health and community health settings. These people were provided with information about when and where vaccination would take place. We planned to subsequently move to health areas closer to the outbreak epicentre, guided by data on estimated risk of EVD, population density, security issues and transport links from Goma. However, plans to expand into new study sites were abandoned when the outbreak came under control and was declared over on 25 June 2020. As a consequence there was no possibility of acquiring vaccine effectiveness data through the trial as the epidemic had not spread to the initial trial areas.

Sample size

The required sample size for the primary effectiveness evaluation was 110 cases (test positives), to provide 80% power with alpha = 0.05 for a two-sided test to demonstrate 70% VE under assumptions for coverage (percentage of vaccinated test negative individuals) ranging from 20-70% and ratio ranging from 1-4 test negatives per positive case (Table 2).

This sample size also provides 80% power to demonstrate 90% VE under assumptions for coverage ranging from 10-70% and ratio of test positives to test negatives ranging from 1-4 test negatives to a test positive case. It will also provide 80% power to demonstrate 50% VE under assumptions for coverage ranging from 50-70% and ratio of test positives to test negatives ranging from 2-4 test negatives to a test positive case.

We estimated that vaccination of approximately 500,000 people with Ad26.ZEBOV, MVA-BN-Filo in transmission areas would achieve the sample size of 110 confirmed EVD cases under the following assumptions: i) an EVD incidence of 4.1/10,000 in the absence of Ad26.ZEBOV/MVA-BN-Filo-induced immunity, corresponding to the median attack rate per

For peer review only - http://bmjopen.bmj.com/site/about/guidelines.xhtml

health area after the first year of the epidemic in the DRC; and ii) 30% of confirmed cases had received the rVSV-ZEBOV-GP (in accordance with surveillance data) and would therefore be excluded from the effectiveness analysis. We assumed a homogeneous attack rate, while recognising that it could vary according to the location of vaccine deployment and the evolution of the epidemic.

After the VE analysis had been conducted based on 110 cases, the study team planned discussions to review the findings and the value of continuing to enrol participants.

Two subsets of individuals, 1000 vaccinated individuals (500 pre-dose 1 and 500 pre-dose 2) and 500 pregnant participants (250 pre-dose 1 and 250 pre-dose 2), were enrolled for more active follow-up for safety reporting (Table 3). The sample size allowed for 95% probability to detect at least one vaccine related SAE if the true incidence of that SAE is at least 1.2% with 250 individuals, 0.6% with 500 individuals, or 0.3% with 1000 individuals.

Community engagement and feedback

Prior to recruiting participants, we set up a specific community engagement structure. Its mission was to inform, sensitize and engage directly with the local population. Initially overseen by Médecins Sans Frontières France (MSF), World Vision was involved in the introduction of specific techniques, including mobile messaging.

Within each health area, Community Advisory Committees (CACs) were constituted. They conducted initial meetings with local political and administrative leaders, social groups and organisations, local associations and other nongovernmental organisations, and traditional and religious leaders, and their input was included in the planning of the trial implementation. We then conducted regular, separate discussion meetings with these different groups every two weeks to receive and integrate feedback on social harms, individual and community level risks, perceptions about the vaccine and the study, legal or administrative complaints and vaccination implementation issues.

Eligibility, recruitment and enrolment

For peer review only - http://bmjopen.bmj.com/site/about/guidelines.xhtml

Using pre-defined eligibility criteria (Box 1), we invited all adults and children aged one year or greater, including pregnant and breastfeeding women, to participate if they lived or worked in the selected health zones and, at the time of vaccination, planned to remain there for one month post-dose 2. Health workers were specifically encouraged to participate. We provided information about the study to local populations through the distribution of posters and flyers, banner displays in the target health areas, radio and television broadcasts and interviews during church gatherings and through interactive street visits using loudspeakers. Until the outbreak was declared over, we provided participants with information on where to present for care if they developed EVD symptoms and informed participants that they could still receive rVSV-ZEBOV-GP as recommended by WHO if eligible.[27] We instructed Ebola Emergency Treatment Centres (ETCs) on how to report that a presenting patient had received Ad26.ZEBOV, MVA-BN-Filo. We required a parent or caregiver to accompany any child aged less than 18 years to the study vaccination site.

Intervention delivery

We established six study vaccination sites within the participating health zones in Goma. Communicating in Swahili, French or a local dialect, Nande, we conducted symptom screening and a temperature check for possible EVD for participants at a triage desk before they could proceed into the clinic. We provided a short medical consultation to participants presenting at triage with fever and/or illness and including, if appropriate, a test for malaria, and treatment if positive, as well as free treatment for other minor illnesses according to national medical protocols. A post-vaccination medical consultation was also offered for any participants complaining of illness during the observation period. We then referred participants to the nearest appropriate health facility if any further medical intervention was required.

At the first visit, delegated medical staff of the trial confirmed eligibility, explained the study aims and procedures, answered any questions, and sought informed consent (and informed assent for children aged 12-17 years), with witnessed consent for illiterate individuals. We offered urine pregnancy tests at the time of vaccination if a participant believed that she might be pregnant and/or if her last menstrual period was more than one month ago. We gave participants the Ad26.ZEBOV vaccination and asked them to remain at the site for 15 minutes to monitor for any immediate adverse effects. We provided participants with a laminated vaccination card with their photograph, which we asked them to present at subsequent visits. Enrolment and dose 1 vaccination took place between 14 November 2019-29 February 2020.

For peer review only - http://bmjopen.bmj.com/site/about/guidelines.xhtml

We collected data on place of residence and telephone numbers from all trial participants and contacted them through mobile phone calls and automated SMS to remind them to attend for dose 2 and followed up, with home visits if these were needed. At the clinic visits for dose 2, similar triage, eligibility and health checks were conducted. Participants who attended more than 84 days post-dose 1 were still able to receive the second dose until the study vaccination sites closed on 10 February 2021.

After the study start, all individuals presenting to an ETC, peripheral health facility or an EVD testing centre were expected to have their rVSV-ZEBOV-GP and Ad26.ZEBOV, MVA-BN-Filo vaccination status confirmed as part of routine data collection, with this information being recorded in the routine Ministry of Health EVD surveillance database. This included recording the participant ID number from the study vaccination card. If the patients were not able to present their cards at the ETC but mentioned being a study participant, ETC staff requested the family to bring the card to the ETC. For lost cards, we instructed ETC staff to collect the patient's name, vaccination and clinic site details, and, if possible, the last vaccination date, and provide this information to the study data management team to retrieve the participant's ID and manually complete the ETC line-list.

Due to the waning EVD outbreak, the Trial Steering Committee decided to suspend dose 1 vaccination on 29 February 2020, after 20,427 participants had been vaccinated. Dose 2 vaccination continued, recognizing that, while the objective of evaluating VE could no longer be met, the other objectives were still achievable. However, due to the COVID-19 outbreak in DRC, all study vaccinations were suspended from 10 April to14 September 2020, while special precautions to ensure the safety of participants and staff from COVID-19 were established. As a result of suspension of activities, the protocol was amended (10 August 2020, version 7.0) to include immunogenicity assessment of the impact of delayed dose 2 vaccination beyond 84 days post-dose 1 and safety follow up windows (Table 3). Following implementation of infection control measures vaccination continued and dose 2 delivery was completed on 9 February 2021.

In addition to the main study, we enrolled participants into three subsets, with consent sought in separate sections in the main study informed consent and assent forms (Appendices 1-5):

1) Immunogenicity sub-study: 57 non-pregnant adults and 90 children (54 children aged 4-11 years and 36 adolescents aged 12-17 years) were enrolled in an immunogenicity sub-study. At selected vaccination centres, we collected venous blood samples (5 mL

For peer review only - http://bmjopen.bmj.com/site/about/guidelines.xhtml

per visit for adults and children aged 6-17 years and 2.5mL per visit for children aged 4-5 years) from consenting participants at the dose 2 visit and 21 days later (window -3/+7 days). We made 0.5mL serum aliquots, which were stored at -20°C in the INRB laboratory in Goma. One aliquot was shipped to Q2 Solutions in the USA for measurement of IgG binding antibodies to EBOV GP by FANG ELISA. The remaining aliquots are being stored in Goma until the end of study and will then be destroyed. We provided participants with an immunogenicity subset attendance card with a photograph and ID number for identification at the 21 days post-dose 2 visit. We deferred blood drawing if participants were unwell at the second visit. Enrolment and site visits of this subset were completed on 7 December 2020. Details of laboratory handling of biological specimens can be found in the Laboratory Analytical Plan.

2) Safety subset: 1000 participants (approximately 500 adults and 500 children pre-dose 1) were asked to consent to a telephone call at 1 month post-dose 2 to collect data on SAEs.

3) Pregnancy subset: up to 500 pregnant participants (250 each, pre-dose 1 and pre-dose 2) were asked to consent to receive telephone calls to collect SAEs at D7 and D21 post-dose 1, and at D7, 1 month, 3 months and 6 months post-dose 2 (unless delivery has occurred before these time points) and data are being collected on pregnancy outcomes as described below.

Safety measurements

Following each vaccination, we gave participants instructions to contact the study team for any SAEs occurring up to one month post-dose 2 and where to seek care for medical problems, including antenatal care for pregnant women (Table 3). Whenever possible, we documented the diagnosis or syndrome related to SAEs, rather than multiple symptoms. Investigators then assessed any potential causal relationship to vaccination. We followed up SAEs to resolution or stabilization, irrespective of severity or whether considered vaccine-related. We allowed unscheduled study follow up visits on adverse events at the investigator's discretion.

Follow-up of pregnant women is ongoing. We compiled a registry of pregnant women and we encouraged women who become pregnant within 1 month of any vaccine dose to contact the team. We are telephoning all known pregnant women 1-3 months after delivery to record birth

For peer review only - http://bmjopen.bmj.com/site/about/guidelines.xhtml

outcomes or, when possible, a study clinician visits them in person if they are not contactable by telephone. In the pregnancy subset, we actively follow by telephone 250 women who were pregnant at the time of dose 1 and up to 250 who become pregnant within 30 days (one month) of administration of either dose 1 or 2. We make calls to collect SAE data at D7 (-3/+7 day) and D21 (-6/+7 day) post-dose 1, D7 (-3/+7 day) post-dose 2, and at 1 month (-7 day/+1 month), 3 months (+/-14 day) and 6 months (-14/+28 day) post-dose 2 (unless delivery occurs before these time points). We are performing a clinical examination on the first 100 infants born to women in the pregnancy subset at around 3 months of age (ranging from 1-6 months). We offer a small bag of rice, cassava or equivalent to mothers returning to the clinic for infant safety follow-up visits.

In the safety subset, we actively followed the first 500 adults and first 500 children enrolled by telephone one month (-7 days/+1 month) after administration of dose 2.

For acute medical problems, we provide care to participants with referral to health clinic and hospitals as necessary. The trial sponsor and funder have put in place global and local clinical trial insurance covers to compensate any potential harm to participants.

Social science methods

We are using multiple modalities to explore the socio-economic and socio-cultural contexts of EVD and the study, including perception of illness and disease, trust in medical research and the motives for the study, key community dynamics and power relations, barriers and opportunities. This information can be used to trace any ongoing rumours and concerns emerging at the community level and to provide appropriate feedback to the study team.

The social science component of the study includes the following:

1) 30 In-depth Interviews: 5 women, 5 men, 5 girls and 5 boys aged 12-17 years old and 10 parents/care-providers of children aged 1-11 years, post-dose-1 or 2.
2) Eight Focus Group Discussions: Including adult participants, parents of children aged 1-11 years, and boys and girls aged 12-17 years.
3) Up to 10 Key Informant Interviews: Local and national stakeholders.
4) Participant exit interviews at vaccination visits to document immediate concerns related to clinic experiences.

For peer review only - http://bmjopen.bmj.com/site/about/guidelines.xhtml

5) Ethnographic observation in study clinics and key sites (e.g. markets, motorbike parking grounds).

At the beginning of all interviews, the social science team ask participants to state that they consent to taking part, recording this both digitally and by hand on the consent form, or only by hand if permission is not given for digital recording. Parents are being asked to provide consent for children under 17 years and children aged 12-17 years to provide assent. Interviews are being conducted in the participant's/parent's/guardian's primary language. Exit interviews are extremely basic and rapid and simply record whether participants had any immediate concerns they wished to report. If in-person interviews are not possible, we conduct phone interviews with adult participants, with researchers reading out information from an information sheet.

Interviews are conducted in French, Swahili, or Nande and then translated to English. We remove personal identifiable information prior to uploading recordings to a SharePoint which is only accessible to the study team. We give each interview participant a unique ID number and delete their phone numbers from study databases immediately after use.

OUTCOMES

The primary study outcomes are:

1) The numbers and odds of vaccination status in EVD cases and in EVD-negative controls in a test negative case control study with a target sample size of 110 laboratory confirmed EVD cases matched to controls who test negative for EVD. Effectiveness is derived from the odds ratio for vaccination in cases compared to controls to calculate VE

The secondary outcomes include:

1) The number and proportion of adults and children with solicited and unsolicited SAEs from the date of first vaccination to one month post-dose 2. Data on SAEs within one month post-dose 2 are considered related to vaccination with Ad26.ZEBOV, MVA-BN®-Filo in adults and children.
2) The number and proportion of adults and children receiving dose 1.
3) The number and proportion of adults and children receiving dose 2.

For peer review only - http://bmjopen.bmj.com/site/about/guidelines.xhtml

4) The number of participants participating in in-depth interviews and focus group discussions on participant and community perceptions of the trial and on vaccine acceptability.

The exploratory outcome includes:

1) The level of IgG antibodies against EBOV GP in samples collected from participants in the immunogenicity subset who received dose 2 outside the recommended interval between dose 1 and dose 2.

DATA COLLECTION, MANAGEMENT AND ANALYSIS

Data Collection

Data collection is ongoing. We collect data using electronic case record forms (eCRFs) developed using Open Data Kit (ODK) software on password protected tablets, and on standardized paper CRFs when eCRF use is not possible.[28] We designed a modular system of ODK forms in English and French to collect data on different aspects of the study, including consent, vaccinations and errors on eCRF completion. Prior to starting the study, we provided and reviewed guidelines for CRF completion with study site personnel. Automated programs in R or STATA summarise study progress and create line-lists for follow-up and dose 2 visits, and to identify errors. Data can be exported as delimited text files. Automated checks ensure data completion. Study team members receive daily updates and reports via a password protected dashboard. eCRFS are also transferred into REDCAP GCP compliant software for an audit trail of data cleaning. Data collected in paper-based CRFs are double-entered directly into a RedCAP database.

After encryption, data are sent via end-to-end encrypted https protocols to a server hosted at the London School of Hygiene & Tropical Medicine (LSHTM), with ongoing transfer of data to servers hosted by INRB and Epicentre via SSH File Transfer Protocol (sFTP). Only the data management team at Epicentre and INRB are able to decrypt the raw data. Study personnel save, store, back-up, transfer and share data in encrypted file formats and over encrypted transmission routes. To the extent possible, we use de-identified data for operational and analytic purposes. Permissions to decrypt data are limited to key personnel. Working copies of databases such as those used by staff who are responsible for progress monitoring and statistical

For peer review only - http://bmjopen.bmj.com/site/about/guidelines.xhtml

analysis are automatically stripped of personal identification data via a process applied by data managers at Epicentre.

We anonymise interview transcripts and field notes and download them to password protected and encrypted computers for transcription and translation and analysis using NVIVO software. We keep all recordings, transcripts and field notes in locked filing cabinets at the study's Goma data centre, before planned long-term storage at INRB Kinshasa. Details of database validation, data collection, coding, security, automatic, query, cleaning and safety data reconciliation can be found in the Data Management Plan.

Vaccine effectiveness analysis

We define cases (test positives) as laboratory-confirmed EVD cases as per DRC Ministry of Health centralized reporting and controls (test-negatives) as those with suspected EVD but whose laboratory tests were negative. Prior to the end of the outbreak, we envisaged that identification of controls would be retrospective, blind to vaccination status, on individuals residing or working in areas that were offered vaccination prior to presentation with symptoms at health facilities, with geographical matching to allow cases and controls to have had equal opportunities to have been vaccinated. Frequency matching or adjustments using regression techniques for other characteristics such as age, sex and possibly also exposure to EBOV, were to be described in the Statistical Analysis Plan.

Since the likely ratio of test-negatives to test-positives at screening centres was unknown, it was recognised that, if a high number of individuals with a very low percentage of confirmed cases presented in a short period of time, random sampling of test-negatives on a frequency matched basis may be required.

If the duration of Ebola outbreak had permitted measurement of vaccine effectiveness, individuals who received rVSV-ZEBOV-GP, or the first but not the second dose of Ad26.ZEBOV, MVA-BN-Filo or who received the second dose less than 21 days prior to testing for EVD would have been excluded from the primary analysis.

For the primary analysis, the odds of having been fully vaccinated (having received the 2 doses, in the right order, at least one month apart, at least 21 days before the onset of symptoms) would have been compared with the odds of not being vaccinated between the cases and controls through the odds ratio (OR), providing an estimate of VE: (VE(%)=(1-OR)x100), with

For peer review only - http://bmjopen.bmj.com/site/about/guidelines.xhtml

regression analyses allowing for appropriate account of any matching and adjustment for potential confounders.

We also planned to examine VE of at least one dose of the vaccine and development of disease within 21 days after the second dose as secondary analyses.

Safety analysis

Safety analyses will include all participants receiving at least dose 1. We will summarise the incidence of serious adverse events (SAEs) overall, by time since vaccination and by age and whether or not vaccination was received during pregnancy. The incidence of SAEs will also be tabulated by MedDRA preferred term and system organ class (Medical Dictionary for Regulatory Activities version 23.1).

Immunogenicity subset analysis

We will determine the number of overall participants with detectable antibodies and maximum titres post-vaccination, including analysis by age group and by time since dose 1 vaccination. We will make descriptive comparisons with other studies of the same vaccine in sub-Saharan Africa where dose 2 was given according to the recommended 56-day interval and, for adults, with other delayed dose 2 immunogenicity data. We will analyse continuous immunologic parameters (e.g. geometric mean concentration of IgG binding antibodies to EBOV GP) and responder rates, and assess the impact of extending the interval between the two doses.

More detailed information on analysis populations, data handling and sub-group analyses is provided in the Statistical Analysis Plan.

Qualitative research analysis

We are conducting inductive and deductive analysis of qualitative data collected by in-depth interviews, focus group discussions and exit interviews. All recordings are being transcribed into French and imported to NVIVO 12. Data are being coded by constant comparative methods to create emergent themes.

Trial Management

LSHTM is the study sponsor. A Trial Steering Committee holds regular meetings by teleconference to monitor study progress (Appendix 6). An independent Data Safety and

For peer review only - http://bmjopen.bmj.com/site/about/guidelines.xhtml

Monitoring Board (DSMB) periodically reviews the safety data and meets according to the participant recruitment timeline specified in the DSMB Charter (Appendix 6). Fatal, life-threatening events and Suspected Unexpected Serious Adverse Reactions are reported to the DSMB within 48 hours and other SAEs within 7 days. An independent Good Clinical Practice (GCP) monitor checks to compliance with the protocol, GCP, standard operating procedures and informed consent processes through review of documents for 5% of study participants through on-site visits and remote monitoring. There will also be one on-site GCP audit by a sponsor representative not involved in conducting the trial before primary data collection has completed. Please see Appendix 7 for the responsibilities of the study sponsor and funders.

ETHICS AND DISSEMINATION

The protocol was reviewed and approved by the Comité National d'Ethique et de la Santé and Comité d'Ethique de l'Ecole de Santé Publique in DRC, the LSHTM Ethics Committee and the Ethics Review Board of Médecins Sans Frontières (Table 1). This study is being implemented according to the Declaration of Helsinki principles and the principles of the International Conference on Harmonisation (ICH) Guideline for GCP. Since the original approved protocol version 5.0, dated 12 September 2019, there has been 1 protocol amendment. This article is based on protocol version 7.0, dated 10 August 2020. All versions and amendments have been approved by the four ethics committees listed above and by the DRC Direction de la Pharmacie et du Médicament. The study has been registered in ClinicalTrials.gov (ClinicalTrials.gov Identifier: NCT04152486).

The population of North Kivu Province is considered vulnerable. Study participation is strictly voluntary. Participants can withdraw from the trial at any time. If provided, the reasons for withdrawal will be recorded. Both communities and, at the time of the outbreak, individuals were informed that study participation did not mean that other EVD infection protection and control measures should be discarded and that they should endeavour to attend for the second dose.

Results of the study will be presented to relevant stakeholders in DRC, shared with WHO's technical expert groups and published in Open Access journals. Any research articles

For peer review only - http://bmjopen.bmj.com/site/about/guidelines.xhtml

generating from this trial will be subject to the Publication Policy and the Authorship Guidelines agreed by all partners.

Data Availability Statement

The rights of study subjects and partners, the sharing of data between partners and the transfer of data to external third party will be governed by the Data Sharing Agreement. Deidentified participant-level data collected in this trial will be disseminated through a FAIR-compliant data repository, such as the LSHTM Data Compass (https://datacompass.lshtm.ac.uk/), from 6 to 60 months after the publication of the main trial results. Other study documents (e.g. full protocol, statistical codes, Statistical Analytical Plan, DSMB Charter) will be available upon request to Deborah Watson-Jones (corresponding author, ORCID: 0000-0001-6247-1746), Tansy Edwards (study statistician, ORCID: 0000-0002-6110-014X) or Edward Choi (study coordinator, ORCID: 0000-0002-8148-120X).

Patient and Public Involvement Statement

Because we invited the general population to participate in this trial, no patients were involved in the discussions on trial design, conduct, reporting or dissemination. Prior to trial commencement, we engaged the Goma population through consultation with local authorities and leaders. Refer to the main article for details.

Author affiliations

[1] London School of Hygiene & Tropical Medicine, London, United Kingdom
[2] Mwanza Intervention Trials Unit, National Institute for Medical Research, Mwanza, Tanzania
[3] L'Institut National de Recherche Biomédicale (INRB), Kinshasa, Democratic Republic of Congo
[4] Epicentre, Paris, France
[5] Médecins Sans Frontières France, Paris, France
[6] Janssen Vaccines and Prevention B.V., Leiden, Netherlands
[7] Coalition for Epidemic Preparedness Innovations (CEPI), Oslo, Norway
[8] UK Public Health Rapid Support Team, Public Health England, United Kingdom
[9] DRC-EB-001 protocol writing team

PROTOCOL WRITING TEAM FOR DRC-EB-001 TRIAL

London School of Hygiene & Tropical Medicine (LSHTM)
Deborah Watson-Jones

For peer review only - http://bmjopen.bmj.com/site/about/guidelines.xhtml

W John Edmunds
Edward M Choi
Chrissy H Roberts
Shelley Lees
Tansy Edwards
Daniela Manno
Peter G Smith
Brian Greenwood
Daniel G Bausch

L'Institut National de Recherche Biomédicale (INRB)
Jean-Jacques Muyembe
Steve Ahuka
Hugo Kavunga-Membo

EPICENTRE
Rebecca Grais
Susan Rattigan
Anton Camacho
Grace Mambula

MSF France
Natalie Roberts
John Johnson
Patient Mumbere Kighoma
Marie Burton

Janssen Vaccines and Prevention B.V.
Maarten Leyssen
Macaya Douoguih
Bart Spiessens

Coalition for Epidemic Preparedness Innovations (CEPI)
Richard Hatchett
Nathalie Imbault
Gerald Voss
Melanie Saville

Inserm-IRD-Université de Montpellier
Eric Delaporte

World Health Organization
Ira M Longini

Acknowledgements

For peer review only

We thank participants, medical and nursing staff for their participation in this study and World Vision for community engagement support.

Contributors

DWJ, JJM, DGB, CHR, RG, WJE, NR, PGS, TE, HKM, ML, AC, EMC, BG, IML, ED and SA conceived and designed the study. DWJ and EMC wrote the protocol with inputs from RG, SR, DGB, HKM, PGS, NI, TE, CHR, WJE, BG, BS, KL, MD, RH, SL, AC and the protocol writing team. TE and PGS provided statistical advice. DWJ and EMC wrote the article. RG, DGB, HKM, PGS, NI, TE, CHR, WJE, BG, JJ, BS, KL, ML, MD, NR, RH, AC, SR, SL, SA and JJM reviewed and revised the manuscript. All authors and the protocol writing team approved the final submitted manuscript.

Funding statement

This study is supported through funds from the Coalition for Epidemic Preparedness Innovations (CEPI) [FELS1903] and the Paul G. Allen Family Foundation. This work was also supported by the Department for International Development (DFID) and Wellcome [220506/Z/20/Z] and by the European Union's Horizon 2020 research and innovation programme under grant agreement No 857935. This publication reflects only the authors' view and the European Commission is not responsible for any use that may be made of the information it contains. CEPI, Wellcome and DFID advised on the study design and protocol.

Competing interests

DWJ, BG and LSHTM are partners on two research consortia (EBOVAC1, EBOVAC3) with Janssen Vaccines and Prevention B.V. funded by the European Commission.

Patient consent for publication

Not required.

For peer review only

For peer review only - http://bmjopen.bmj.com/site/about/guidelines.xhtml

Table 1. The World Health Organization Trial Registration Data Set for the DRC-EB-001 trial

Data category	Information
Primary registry and trial identifying number	ClinicalTrials.gov NCT04152486
Date of registration in primary registry	5 November 2019
Source(s) of monetary or material support	Coalition for Epidemic Preparedness Innovations (CEPI), the Paul G. Allen Family Foundation and Wellcome
Sponsor	London School of Hygiene & Tropical Medicine (LSHTM)
Sponsor contact and Contact for public queries	Deborah Watson-Jones deborah.watson-jones@lshtm.ac.uk
Public title	Evaluating the Effectiveness and Safety of a Two-dose Ebola Vaccine in the Democratic Republic of the Congo
Scientific title	Evaluation of a heterologous, two-dose preventive Ebola vaccine for effectiveness and safety in the Democratic Republic of the Congo
Countries of recruitment	The Democratic Republic of the Congo
Health condition(s) studied	Ebola virus disease (EVD)
Intervention(s)	Ad26.ZEBOV, MVA-BN-Filo
Key inclusion criteria	Healthy volunteers<br>Aged ≥ 1 year<br>Both sexes (including pregnant and breastfeeding women)
Key exclusion criteria	Known EVD infection<br>Having received any experimental EVD vaccines within 1 month
Study type	Interventional
Allocation	Open label, single arm
Primary purpose	Prevention
Phase	III
Date of first enrolment	14 November 2019
Target sample size	500,000
Recruitment status	Complete

For peer review only - http://bmjopen.bmj.com/site/about/guidelines.xhtml

**Primary outcome(s)**	To estimate the vaccine effectiveness of population-based vaccination delivery with the Ad26.ZEBOV, MVA-BN-Filo vaccine regimen for the prevention of EVD in adults and children aged 1 year or above. (Timeframe: From 2 weeks after intervention to end of study)
**Key secondary outcomes**	To assess safety of the Ad26.ZEBOV, MVA-BN-Filo vaccine (Timeframe: From dose 1 vaccination to 1 month post dose 2)<br>To estimate the coverage of dose 1 and dose 2 of the Ad26.ZEBOV, MVA-BN-Filo vaccine regimen overall and in different target groups (Timeframe: vaccination period)<br>To explore the knowledge and perceptions of persons eligible for large-scale delivery of a preventative Ebola vaccine with a two-dose vaccine strategy (Timeframe: vaccination period)
**Other outcomes**	To assess immunogenicity of the Ad26.ZEBOV, MVA-BN-Filo vaccine in a subset of participants who received dose 2 outside the recommended interval between dose 1 and dose 2.
**Ethics Review 1**	Approved by LSHTM Ethics Committee<br>Date of approval: 15 Oct 2019<br>Name and contact details: Rebecca Carter<br>ethics@lshtm.ac.uk
**Ethics Review 2**	Approved by Médecins Sans Frontières Ethics Review Board<br>Date of approval: 15 Octo 2019<br>Name and contact details: Raffaella Ravinetto<br>raffaella.ravinetto@gmail.com
**Ethics Review 3**	Approved by Comité d'éthique de l'école de santé publique, Université de Kinshasa<br>Date of approval: 22 Oct 2019<br>Name and contact details: Willy Bongopasi<br>bongopasi@gmail.com
**Ethics Review 4**	Approved by Comité national d'éthique et de la santé du Ministère de la Santé Publique de la République Démocratique du Congo (RDC)<br>Date of approval: 23 Oct 2019<br>Name and contact details: Félicien Munday<br>feli1munday@yahoo.fr

For peer review only

Table 2. Sample sizes for a test-negative case-control study design, assuming 80% power to detect an OR = 1-VE, by percent coverage amongst controls (those testing negative) and number of controls per case, with a two-sided alpha=0.05 test.

Vaccine efficacy (VE)	Coverage (% vaccinated amongst controls)	Sample size (no. of cases) by number of controls per case			
		1	2	3	
90%	5%	210	171	157	
	10%	103	84	77	
	20%	50	40	37	
	30%	32	26	23	
	50%	19	15	13	
	70%	14	11	9	
70%	5%	418	333	303	
	10%	211	167	152	
	20%	107	84	76	
	30%	74	57	52	
	50%	50	38	34	
	70%	46	34	30	
50%	5%	962	749	677	
	10%	492	383	345	
	20%	260	201	181	
	30%	186	143	128	
	50%	137	103	92	
	70%	141	105	92	
30%	5%	3070	2350	2109	
	10%	1593	1218	1092	
	20%	866	659	590	
	30%	638	483	432	
	50%	499	375	334	
	70%	553	412	364	

For peer review only

Table 3. Time and Event Schedule for Intervention Delivery and Safety Evaluations

Preparation	Up to 1 month before study starts	D0 Dose 1 visit	D7 (-3/ + 7 day)	D21 (-6/ +7 day)	D56 Dose 2 visit (-14/ +28 day)	D7 post-dose 2 (-3/ +7 day)	D21 post-dose 2 (-3/ +7 day)	1 month post-dose 2 (-7 day/+1 month)	3 months post-dose 2 (+/-14 day)	6 months post-dose 2 (-14/ +28 day)	3 months post-delivery
Mapping & selection of study sites	X										
Community engagement & Social Mobilization	X										
Consent, Vaccination dose 1		X									
Vaccination dose 2					X[2]						
SAE assessment					X[1]			X[3]			
Safety subset					X			X[4]			
All pregnant women					X[9]			X			X[6]
Pregnancy subset		X	X[5]	X[5]	X	X[5]		X[5]	X[5]	X[5]	X[7]
Immuno-genicity subset					X[8]		X[8]				

[1] D56 safety data are collected at dose 2 visits.

[2] Dose 2 may be given between D42 and D84. Participants who return for dose 2 after 84 days will still be vaccinated.

[3] Passive SAE recording from main study participants through participant-initiated phone calls or other contact with team, until 1 month post-dose 2.

[4] Safety subset: 1000 participants will be actively followed up for SAEs by phone at 1 month post-dose 2.

[5] Pregnancy subset: safety telephone calls to collect SAEs at D7 and D21 post-dose 1, and then at D7, 1 month, 3 months and 6 months post-dose 2, unless delivery is before these time points.

[6] All pregnant women will have their birth outcomes collected around 3 months post-delivery (between 1 to 3 months). When feasible, team to visit if the woman is not contactable by telephone.

[7] 100 infants born to women in the pregnancy subset will be given a full clinical examination around 3 months after delivery (between 1 to 6 months).

[8] Immunogenicity subset: A subset of paediatric and adult participants will have a venous blood draw at the dose 2 visit before vaccination. A second blood sample will be taken 21 days (-3/+7 day) after their dose 2 vaccination. Enrolment of this subset will continue until at least 50 adults and 50 children have had blood samples collected for immunogenicity at dose 2 and 21 days post-dose 2.

[9] If study vaccinations have been paused, all pregnant participants who have not received dose 2 will be called at D56 (+/-7 days) to collect safety data.

For peer review only

For peer review only - http://bmjopen.bmj.com/site/about/guidelines.xhtml

Box 1 DRC-EB-001 inclusion and exclusion criteria for vaccination

Inclusion criteria

1. Must provide a written or witnessed (if illiterate) informed consent form indicating that he or she understands the reasons for the study and is willing to participate in the study and to be vaccinated. If less than 18 years old, must have a parent or guardian that is able to meet this criterion.
2. Must be aged 1 year or older.
3. Must be healthy in the investigator's clinical judgment as assessed on the day of vaccination.
4. Must be willing to have a photograph taken.
5. Must be available and willing to participate for duration of study visits and follow up.

Exclusion criteria

1. Known history of EVD.
2. Has received any experimental EVD vaccine less than one month prior to visit 1.
3. Known allergy or history of anaphylaxis or other serious adverse reactions to vaccines or vaccine products, egg and egg proteins or gentamicin.
4. Presence of an acute illness (excluding minor illnesses such as mild diarrhea or mild upper respiratory tract infection) or temperature ≥38.0ºC at visit 1 (dose 1 visit). Participants with such symptoms will be temporarily excluded from vaccination at that time but may be rescheduled for vaccination at a later date if feasible (and if within 84 days of the first dose).
5. Presence of significant conditions or clinically significant findings at the vaccination visit for which, in the opinion of the investigator, vaccination would not be in the best interest of the participant.
6. History of recurrent generalized hives.

Note: Participants who have received treatment for acute, uncomplicated malaria are eligible for vaccination if at least 3 days have elapsed from the conclusion of a standard, recommended course of therapy for malaria.

Rescheduling for dose 1 or dose 2 is possible in case of acute illness at the time of planned vaccination as long as rescheduling for dose 2 is within 84 days of dose 1.

For peer review only - http://bmjopen.bmj.com/site/about/guidelines.xhtml

References

1. Blumberg L ED, Bausch D G. Viral Haemorrhagic Fevers. Oxford: Elsevier Expert Consult; 2014.
2. Leroy EM, Kumulungui B, Pourrut X et al. Fruit bats as reservoirs of Ebola virus. Nature. 2005;438(7068):575-6.
3. Letko M, Seifert SN, Olival KJ et al. Bat-borne virus diversity, spillover and emergence. Nat Rev Microbiol. 2020;18(8):461-71.
4. Goldstein T, Anthony SJ, Gbakima A et al. The discovery of Bombali virus adds further support for bats as hosts of ebolaviruses. Nat Microbiol. 2018;3(10):1084-9.
5. De Nys HM, Kingebeni PM, Keita AK et al. Survey of Ebola Viruses in Frugivorous and Insectivorous Bats in Guinea, Cameroon, and the Democratic Republic of the Congo, 2015-2017. Emerg Infect Dis. 2018;24(12):2228-40.
6. Leroy EM, Epelboin A, Mondonge V et al. Human Ebola outbreak resulting from direct exposure to fruit bats in Luebo, Democratic Republic of Congo, 2007. Vector Borne Zoonotic Dis. 2009;9(6):723-8.
7. Jacob ST, Crozier I, Fischer WA, et al. Ebola virus disease. Nat Rev Dis Primers. 2020;6(1):13. DOI: 10.1038/s41572-020-0147-3
8. Henao-Restrepo AM, Camacho A, Longini IM et al. Efficacy and effectiveness of an rVSV-vectored vaccine in preventing Ebola virus disease: final results from the Guinea ring vaccination, open-label, cluster-randomised trial (Ebola ça Suffit!). Lancet. 2017;389(10068):505-18.
9. World Health Organization SAGE Ebola Virus Disease, Meeting of the Strategic Advisory Group of Experts on Immunization, October 2018 - Conclusions and recommendations. Wkly Epidemiol Rec. 2018 49, 2018, 93, 661-680.
10. World Health Organization. News. Four countries in the African region license vaccine in milestone for Ebola prevention. 14 Feb 2020. Available at: https://www.who.int/news/item/14-02-2020-four-countries-in-the-african-region-license-vaccine-in-milestone-for-ebola-prevention (accessed 6 Mar 2021).
11. World Health Organization. Ebola Virus Disease - Democratic Republic of the Congo External Situation Report 1. World Health Organization; 7 August 2018. https://reliefweb.int/sites/reliefweb.int/files/resources/SITREP-EVD-DRC-20180511.pdf (accessed 8 May 2021).
12. MONUSCO. United Nations Organization Stabilization Mission in the DR Congo. 2019. https://monusco.unmissions.org/en (accessed 5 May 2021).
13. World Health Organization R&D Blueprint. Ebola vaccine candidates. World Health Organization; 12 April 2019. https://www.who.int/blueprint/priority-diseases/key-action/ebola-vaccine-candidates/en/ (accessed 7 Mar 2021).
14. Milligan ID, Gibani MM, Sewell R et al. Safety and Immunogenicity of Novel Adenovirus Type 26- and Modified Vaccinia Ankara-Vectored Ebola Vaccines: A Randomized Clinical Trial. JAMA 2016;315(15):1610-23.
15. Mutua G, Anzala O, Luhn K et al. Safety and Immunogenicity of a 2-Dose Heterologous Vaccine Regimen With Ad26.ZEBOV and MVA-BN-Filo Ebola Vaccines: 12-Month Data From a Phase 1 Randomized Clinical Trial in Nairobi, Kenya. J Infect Dis. 2019;220(1):57-67.

For peer review only - http://bmjopen.bmj.com/site/about/guidelines.xhtml

16. Pollard AJ, Launay O, Lelievre JD et al. Safety and immunogenicity of a two-dose heterologous Ad26.ZEBOV and MVA-BN-Filo Ebola vaccine regimen in adults in Europe (EBOVAC2): a randomised, observer-blind, participant-blind, placebo-controlled, phase 2 trial. Lancet Infect Dis. 2020;21(4):493-506.
17. Ishola D MD, Afolabi MO, Keshinro B et al. Safety and long-term immunogenicity of a two-dose Ad26.ZEBOV, MVA-BN-Filo Ebola vaccine regimen in adults: a randomised, double-blind, controlled trial in Sierra Leone. Lancet Infect Dis. 2021;In press.
18. Barry H MG, Kibuuka H, Anywaine Z et al. A randomized clinical trial of the safety and immunogenicity of a 2-dose heterologoour ebola vaccine regimen with Ad26.ZEBOV and MVA-BN-FILO® in healthy and HIV+ African Adults. American Society of Tropical Medicine and Hygiene, 68th Annual Meeting, National Harbor, MD, USA. 20-24 Nov 2019. Abstract number 1291.
19. Afolabi MO, Manno D, Keshinro B et al. Safety and immunogenicity of a two-dose Ad26. ZEBOV, MVA-BN-Filo Ebola vaccine in children: a randomised, double-blind, controlled trial in Sierra Leone. Lancet Infect Dis. 2021;In press.
20. Johnson & Johnson. Johnson & Johnson Announces European Commission Approval for Janssen's Preventive Ebola Vaccine. 1 Jul 2020. Available at: https://www.jnj.com/johnson-johnson-announces-european-commission-approval-for-janssens-preventive-ebola-vaccine (accessed 24 Jan 2021).
21. European Medicines Agency. New vaccine for prevention of Ebola virus disease recommended for approval in the European Union. 29 May 2020. Available at: https://www.ema.europa.eu/en/news/new-vaccine-prevention-ebola-virus-disease-recommended-approval-european-union (accessed 24 Jan 2021).
22. Rudge TL, Jr., Sankovich KA, Niemuth NA et al. Development, qualification, and validation of the Filovirus Animal Nonclinical Group anti-Ebola virus glycoprotein immunoglobulin G enzyme-linked immunosorbent assay for human serum samples. PLoS One. 2019;14(4):e0215457.
23. Dean NE, Gsell P-S, Brookmeyer R et al. Design of vaccine efficacy trials during public health emergencies. Sci Transl Med. 2019; 11(499) eaat0360.
24. World Health Organization R&D Blueprint. Design of vaccine efficacy trials to be used during public health emergencies – Points of considerations and key principles. 2018. https://www.who.int/docs/default-source/blue-print/working-group-for-vaccine-evaluation-(4th-consultation)/ap1-guidelines-online-consultation.pdf (accessed 4 May 2021).
25. De Serres G, Skowronski DM, Wu XW et al. The test-negative design: validity, accuracy and precision of vaccine efficacy estimates compared to the gold standard of randomised placebo-controlled clinical trials. Euro Surveill. 2013;18(37). DOI: 10.2807/1560-7917.es2013.18.37.20585.
26. Verani JR, Baqui AH, Broome CV et al. Case-control vaccine effectiveness studies: Preparation, design, and enrollment of cases and controls. Vaccine. 2017;35(25):3295-302.
27. World Health Organization Global Ebola Vaccine Implementation Team (GEVIT) Practical guidance on the use of Ebola vaccine in an outbreak response. World Health Organization. May 2016. https://www.who.int/csr/resources/publications/ebola/gevit_guidance_may2016.pdf (accessed 4 May 2021)
28. Marks M, Lai S, Brindle H et al. Electronic data management for vaccine trials in low resource settings: Upgrades, scalability and impact of ODK. MedRxiv 20210208 [Preprint]. February 10, 2021. https://www.doi.org/10.1101/2021.02.08.20191908 (accessed 4 May 2021).

For peer review only - http://bmjopen.bmj.com/site/about/guidelines.xhtml

Protocol for a phase 3 trial to evaluate the effectiveness and safety of a heterologous, two-dose vaccine for Ebola virus disease in the Democratic Republic of the Congo

Appendix 1A

Informed Consent Form in English, Version 7.1 (10 Aug 2020)

For peer review only

For peer review only - http://bmjopen.bmj.com/site/about/guidelines.xhtml

Evaluation of a heterologous, two-dose preventive Ebola vaccine for effectiveness and safety in the Democratic Republic of the Congo

"The TUJIOKOWE Study" Information Sheet and Informed Consent Form

LSHTM Protocol: DRC-EB-001

Principal Investigator (PI):
Professor Jean Jacques Muyembe
Director-General
Institut National de Recherche Biomédicale
Professor of Microbiology, Kinshasa University Medical School
Kinshasa Gombe, Democratic Republic of the Congo (DRC)
Phone: 0898 949 289; Email: jjmuyembet@gmail.com

Sponsor: London School of Hygiene & Tropical Medicine, United Kingdom

Site: Democratic Republic of the Congo

INTRODUCTION

You are being invited to take part in a research study called the 'TUJIOKOWE study' to find out if a new two-dose vaccine called the 'Janssen Ebola vaccine' can protect people from getting Ebola and to check whether the vaccine is safe. The TUJIOKOWE study is being implemented by the Ministry of Health of DRC through the Institut National de Recherche Biomédicale (INRB), Epicentre and the London School of Hygiene & Tropical Medicine (LSHTM).

WHAT IS EBOLA?

Ebola is a disease named after the Ebola virus. When a person gets Ebola, they can become very sick, and about half of the people who catch Ebola die. Once a person has Ebola, the disease can be spread to other people, especially if people are living in the same house.

WHAT IS A VACCINE AND WHAT IS A CLINICAL RESEARCH STUDY?

Vaccines are medical products that are used to stop people from having certain diseases. Many people get vaccines to prevent them catching diseases like measles, polio, and cholera, for example.

A clinical research study helps health professionals understand new ways to prevent or treat a disease. In this study, the vaccine is still under testing. Participants in research studies about vaccines are followed over time so that the researchers can learn more about the vaccine.

For peer review only - http://bmjopen.bmj.com/site/about/guidelines.xhtml

ARE ANY OTHER VACCINES BEING GIVEN TO PREVENT EBOLA IN DRC?

Vaccination is occurring with a vaccine called "VSV" in several places like Beni, Katwa and Butembo. This vaccine is only being given to people who are at risk of catching Ebola, such as people who have come into contact with someone who has Ebola, or who have come into contact with people close to someone with Ebola. It is also being given to community health workers and other workers who may met Ebola. However, about half of the people who become infected with Ebola in DRC are not known to have had contact with an Ebola case, so we need to protect them too. Therefore, we are looking for additional ways to protect people from Ebola, including this vaccine.

WHAT IS THIS STUDY ABOUT?

This study is going to find out if a new vaccine can help protect people against Ebola. This new vaccine is given in two injections. The first injection is a dose of a vaccine called Ad26.ZEBOV. A second injection is given about two months later, with a second dose called MVA-BN-Filo. In this study, we will call these two injections with two different vaccines the 'Janssen Ebola vaccine'.

In the rest of this document, we will talk about you. The word 'you' can mean you or your child. If you are the parent or guardian of a child under 18 years of age and you would like your child to join this study, you will need to sign separate documents for you and your child.

If your child is aged 12 to 17 years old, he/she will also need to agree to join the study himself/herself and sign a different document, called the assent form.

WHAT DO WE KNOW ABOUT THE VACCINE?

The Janssen Ebola vaccine is designed to protect people against Ebola. **It cannot make you sick with Ebola.** Over 6000 people have received the vaccine in many different countries, including 7 in Africa. So far, the vaccine is safe and well-tolerated in people who received it. The vaccine may help the body to fight Ebola infections, but we don't know for sure yet if the Janssen Ebola vaccine can protect people against Ebola.

WHO CAN TAKE PART IN THIS STUDY?

To be in this study, you must be living in or working in one of the communities that will be offered the Janssen Ebola vaccine. You must be able to return to get the second dose of vaccine and be willing to follow the requirements needed to take part in this study. Participants should be well in order to get each dose of the vaccine. People diagnosed with HIV (who are well), pregnant and breastfeeding women, and children aged 1 year or older can be included in this study.

You cannot be in this study if you are an Ebola survivor. You also cannot be in this study if you are allergic to eggs or egg products.

For peer review only - http://bmjopen.bmj.com/site/about/guidelines.xhtml

If you have received another Ebola vaccine or routine vaccine within the last month (30 days) or if you have a fever of 38°C or higher on the vaccination day, you may be asked to come back another day. You may receive the vaccine if you have a mild cold, cough or mild diarrhea.

DO I HAVE TO BE IN THIS STUDY?

You do not have to join this study if you don't want to. If you agree to be in the study, we will ask you to sign this consent form. If you decide that you don't want to be in the study, then this will not affect you in any way. If you don't want to be in the study, but you change your mind later, you will have another chance to join the study. After the second visit by the team to your community, it may not be possible to join this study to get the Janssen Ebola vaccine. You must be in the study to receive the vaccine.

WHAT WILL HAPPEN IF I AGREE TO TAKE PART IN THIS STUDY?

We will describe the study and answer any questions that you may have. If you would like a copy of the written information, we will give it to you. You will be asked some questions about the study by one of the study staff members to check that you have understood the information. You will be asked to sign or put your fingerprint on this informed consent form. Putting your name or your fingerprint on the consent form means that you agree to be in the study, but you can change your mind and choose to leave at any time.

WHAT DOES THE STUDY INVOLVE?

After you sign this consent form, you will be asked some questions to check how you are feeling. If the doctor decides that you can be vaccinated, you will get an injection of the first Janssen Ebola vaccine into your upper arm, or the thigh for younger children. We will give you a vaccination card with a phone number of who to contact in case you need it. The card will also show the date of when you need to come back for the second Janssen Ebola vaccine. We will ask you for a phone number and we may call you for the second vaccine and follow ups.

In order to identify you at the next visit, we will take photographs of your face. We do this to make sure that we know who has been vaccinated and ensure you receive the second dose at the right time.

You will be asked to attend on a specific date for the second Janssen Ebola vaccine. After the second dose, we will ask you to call us if you have any medical problems in the 30 days after your second vaccine dose. You should report any sickness to the study team in those 30 days by calling the phone number on your vaccination card. We will ensure that there are telephones available in your community.

A certain number of participants will be included in a group for follow up who will be contacted by phone or a home visit.

As a result of the study pause due to COVID-19, some participants had their second vaccination with the Janssen Ebola vaccine postponed. These participants will be called to receive their

For peer review only - http://bmjopen.bmj.com/site/about/guidelines.xhtml

second vaccination at a later date. Receiving the second Janssen Ebola vaccine at a later date will not have negative effects on the health of these participants.

WHAT ARE THE POSSIBLE RISKS OF BEING IN THIS STUDY?

We do not know for sure yet if the Janssen Ebola vaccine protects people from Ebola, so there is a risk that you will not be protected from Ebola even after you have taken the vaccine.

This vaccine has been given to more than 6000 people in other research studies. Some of the side effects that you may have after the injections are:

- pain, swelling, itchiness, redness, or warmth around the place where you got the injection.
- in the area around the injection it may be sore and a bit painful to move for a few days.
- you may notice that you have swollen bumps under your arms or in your neck for a few days.
- you may get a fever or headache, feel tired, or have chills, rash, nausea, change in appetite, vomiting, diarrhea, dizziness, muscle, joint or belly aches, mouth sores, or body pains.

In the previous research studies, not everyone has gotten these side effects after the injections. Side effects are usually mild and most only last a few days. In a few people they may last for a few weeks. Some people have reported that they felt some tingling in the hands and feet, or their muscles felt weak. These symptoms usually only lasted one or two days, but sometimes for several weeks. One person reported tingling, numbness and pain which lasted for several months and interfered with the person's daily activities.

We have learnt that 3 women living in other countries who were less than 40 years old had serious blood clots within 3 weeks of receiving a vaccine that was made in a similar way to the second Janssen Ebola vaccine. One of them had a stroke and died. A 50-year-old man also reported lack of blood flow to the leg. These cases also had medical conditions that are known to increase the risk of blood clots and none of these events was considered related to vaccine. Receiving the vaccine did not increase a person's chance of experiencing these problems.

Hundreds of babies (aged 1-3 years), children (aged 4-11 years) and adolescents (aged 12-17 years) have received the Janssen Ebola vaccine so far. The reported side effects in children and adolescents after vaccination were similar to those reported by adults. Injection site pain was the most frequently reported local side effect. Headache, fatigue and chills were also frequently reported in older children and adolescents. Decreased appetite, decreased activity and fever were the most frequently reported side effects in babies. Following vaccination, a few children experienced a fever above 39°C. Most of these side effects were mild to moderate and did not last more than 1 or 2 days. No serious medical problems have been seen in children who received the vaccine. The side effects in children are similar to those that happen with other vaccines in children.

The medical team will provide care if you experience side-effects from the injections up until a month after the second dose. They are available if you have any problems or questions.

For peer review only - http://bmjopen.bmj.com/site/about/guidelines.xhtml

WHAT ARE THE BENEFITS OF BEING IN THIS STUDY?

On the days of vaccination, a doctor will question you to check if you have any symptoms and, if necessary, you may receive free treatment or a referral for simple medical conditions like malaria. You will also learn how to protect yourself against Ebola. Your participation in this study will help in the development of vaccines to prevent Ebola and, in the future, may help people in different parts of the world.

WHAT ABOUT PREGNANCY AND/OR BREASTFEEDING DURING THE STUDY?

You can participate in this study if you are pregnant or breastfeeding. We do not know all the effects of the study vaccines in pregnancy or in a baby that is being breastfed. If you know you are pregnant or breastfeeding at the time of vaccination, please tell the study staff. If you are not sure, we will offer you a pregnancy test and medical interview and will let you know if you are pregnant before each vaccination. If you are pregnant or become pregnant within one month of each vaccination, we will contact you during your pregnancy to ask you questions about your health and your baby's health. We will also contact you within 3 months after the delivery of your baby. If we cannot contact you by telephone after your baby is born, the study team may visit you at your home. Among the pregnant women in the study, a certain number will be invited to join a pregnancy subset and will be followed up by telephone or through home visits more frequently and some babies of women in the pregnancy subset will also be seen within 3 months after birth. If you become pregnant within one month after vaccination, please contact the phone number on your vaccination card and let the study team know.

WHAT DO I NEED TO DO FOR MY OWN HEALTH DURING THIS STUDY?

If you become sick during the study, you should go to one of the recommended health facilities that are on the list that we will give you. You should also call the study contact phone number on your vaccination card and tell us if you are having any medical problems up until 30 days after you received the second dose of the vaccine. You may be referred for medical care if necessary.

We do not know for sure yet if the Janssen Ebola vaccine can protect people against Ebola. If you get the vaccine, you may be protected from Ebola, but it is possible that you may not be protected. **Therefore, you must continue to protect yourself from contact with Ebola.** We will give you specific information on how to prevent Ebola, and you should always contact a member of the study team if you have any questions about how to protect yourself from contact with Ebola. If you experience anything that seems like the symptoms of Ebola, such as fever, diarrhea, vomiting, or unexplained bleeding, it is very important that you let the study team know that you are sick and that you seek medical care right away.

If we find out anything new during this study that may be related to your health or affect your decision to continue in the study, we will talk to you about it.

For peer review only - http://bmjopen.bmj.com/site/about/guidelines.xhtml

WHO WILL BE ABLE TO SEE MY INFORMATION?

We will keep your study information private.

Staff working on the study, including people from overseas who are responsible for checking that this study is done properly, ethics committees, and regulatory authorities, may look at your medical records. All study staff are trained in confidentiality and will not remove any document with your name on it from the study files. All of these people understand that they must keep your identity private and they won't share your name or contact details with anyone.

A description of this study will be available on the internet at http://www.ClinicalTrials.gov. No information that can identify you will be on this website. You can look at this website at any time.

The study results will be shared, but no personal information will be included in the published study results. If you have any specific questions about your data, please contact the study Principal Investigator, Professor Jean Jacques Muyembe or his representative (see contact details below).

WHO IS CHECKING THAT THE STUDY IS SAFE?

This study is being implemented by partners including the Ministry of Health of DRC through the Institut National de Recherche Biomédicale (INRB), Epicentre, and the London School of Hygiene & Tropical Medicine (LSHTM). An independent committee made up of doctors and experts who are not directly involved in the study will be looking at the study information and checking on the safety of the vaccines.

For peer review only - http://bmjopen.bmj.com/site/about/guidelines.xhtml

WHO CAN I TALK TO ABOUT THIS STUDY?

If you want to talk to someone about this study or if you think you have been harmed by being part of the study, you can contact the following.

1) The Principal Investigator responsible for this study

Professor Jean Jacques Muyembe<br>*Director General*, Institut National de Recherche Biomédicale, Kinshasa, DRC<br>Phone: 0898 949 289<br>Email: jjmuyembet@gmail.com<br><br>Local study representative<br>Dr Hugo Kavunga<br>Phone: 0823 875 153<br>Email: hugokavunga@gmail.com

2) The DRC ethics committees that approved this study

Professor Félicien Munday<br>National Ethics Committee<br>Kinshasa-Gombe, DRC<br>Phone: 0998 419 816<br>Email: feli1munday@yahoo.fr
Professeur Willy Bongopasi<br>Comité d'éthique de l'école de santé publique<br>Université de Kinshasa<br>Téléphone: 0999 952 341<br>Email: bongopasi@gmail.com

If you have any questions about the TUJIOKOWE study or about your rights as a study participant, you may ask anyone on the study team at any time.

For peer review only - http://bmjopen.bmj.com/site/about/guidelines.xhtml

PARTICIPANT OR PARENT/GUARDIAN CONSENT FORM

Title: TUJIOKOWE Study

Principal Investigator of this study: Prof. JJ Muyembe

**Statements**	**Please sign or fingerprint each box**
I have read the information in this form about the TUJIOKOWE Study (or I have had this information explained to me by the study staff in a language that I understand). The purpose of this study and the procedures to receive this new vaccine have been fully explained to me. I was able to ask questions and have all of these questions answered to my satisfaction.	
I understand that my/my child's participation is voluntary, that I/my child can withdraw consent and leave this study at any time without giving any reason, and that this will not affect my/my child's medical care or legal rights.	
I have been informed that the new Janssen Ebola vaccine is still being tested, and that not all the possible risks from having this vaccine are completely known.	
I confirm that I/my child have **NOT** previously had Ebola or been vaccinated against Ebola within the past 30 days.	
I agree to have my/my child's photograph taken by the study team before vaccination.	
I agree to give the study team a contact phone number if I have one and to be contacted for the second vaccine and for follow up calls or SMS e.g. for health questions. If I don't have a phone, I agree to try and provide a relative or friend's number that can be used to contact me.	
I understand that the information collected about me/my child will be used to support other research in the future and may be shared anonymously with other researchers.	
I agree that my/my child's medical information that will not contain my/my child's name, can be shared with the national health authorities, the vaccine manufacturer (Janssen Vaccines & Prevention B.V.) and other foreign organizations outside the DRC, such as the US Food & Drug Administration. I give permission for individuals in these organizations to access and analyse my/my child's records and publish them in scientific journals.	

For peer review only - http://bmjopen.bmj.com/site/about/guidelines.xhtml

PARTICIPANT OR PARENT/GUARDIAN CONSENT FORM

Declarations Regarding Optional Procedures

Statements	Please sign or fingerprint each box for yes	Please sign or fingerprint each box for no
(For female participants only) I agree that, if I/my child am pregnant at the time of vaccination or become pregnant during the first month after receiving each vaccine, and I/my child am selected by the study team to participate in the pregnancy subset, the study team will contact me by telephone during my/my child's pregnancy more often than other pregnant women in the study and after the birth of my/my child's baby a member of the study team may see me/my child at home if I cannot be contacted by telephone.		
I agree that if I/ my child am selected by the study team to be in the safety group, the study team may contact me by telephone after my second vaccination and may see me/ my child at home if I cannot be contacted by telephone.		

FOR ADULT PARTICIPANT

I am aged 18 years or older and I agree to take part in this study. (Please sign or put your fingerprint below)

		____/____/_____ *dd mon yyyy*
Printed name of adult participant	Signature/fingerprint of adult participant	Date

FOR PARENT OR GUARDIAN

I am the parent or guardian of a participant aged 1 to 17 years and I agree for my child to take part in this study. (Please sign or put your fingerprint below)

		____/____/_____ *dd mon yyyy*
Printed name of the parent or guardian	Signature/fingerprint of the parent or guardian	Date

For peer review only - http://bmjopen.bmj.com/site/about/guidelines.xhtml

FOR PARENT OR GUARDIAN

Printed name of the participant (if aged 1-17)

		____/____/_____ *dd mon yyyy*
Printed name of investigator	Signature of investigator	Date

Complete next section if participant or parent/guardian is illiterate:

Witness to Consent Interview

I witnessed the consent interview for the TUJIOKOWE Study in this document. I attest that I have explained the study information accurately to the participant or parent/guardian and was understood to the best of my knowledge by the participant or parent/guardian, and that he/she has freely given their consent to participate/for his/her child to participate in my presence.

		____/____/_____ *dd mon yyyy*
Printed name of impartial witness	Signature of impartial witness	Date

---

Attach ID barcode label below:

For peer review only - http://bmjopen.bmj.com/site/about/guidelines.xhtml

For peer review only

Protocol for a phase 3 trial to evaluate the effectiveness and safety of a heterologous, two-dose vaccine for Ebola virus disease in the Democratic Republic of the Congo

Appendix 1B

Informed Consent Form in French, Version 7.1 (10 Aug 2020)

For peer review only

For peer review only - http://bmjopen.bmj.com/site/about/guidelines.xhtml

Évaluation de l'efficacité et de l'innocuité de l'emploi d'un vaccin préventif hétérologue à deux doses contre Ebola en République démocratique du Congo

Note d'information et formulaire de consentement éclairé de l'« étude TUJIOKOWE »

Protocole LSHTM : DRC-EB-001

Investigateur principal (IP) :
Professeur Jean Jacques Muyembe
Directeur général
Institut national de recherche biomédicale
Professeur de microbiologie, Faculté de médecine de l'Université de Kinshasa
Kinshasa Gombe, République démocratique du Congo (RDC)
Téléphone : 0898 949 289 ; E-mail : jjmuyembet@gmail.com

Promoteur : London School of Hygiene & Tropical Medicine, Royaume-Uni

Site : République démocratique du Congo

INTRODUCTION

Vous êtes invité(e) à participer à une étude de recherche appelée l'« étude TUJIOKOWE » pour savoir si un nouveau vaccin à deux doses appelé « vaccin Janssen Ebola » peut protéger contre le virus Ebola et pour vérifier si le vaccin est sans danger. L'étude TUJIOKOWE est mise en œuvre par le Ministère de la Santé de la RDC par l'intermédiaire de l'Institut national de recherche biomédicale (INRB), Epicentre et la London School of Hygiene & Tropical Medicine (LSHTM).

QU'EST-CE QUE LE VIRUS EBOLA ?

Ebola est une maladie dont le nom vient du virus Ebola. Lorsqu'une personne contracte le virus Ebola, elle peut parfois tomber très malade, et environ la moitié des personnes qui contractent le virus Ebola meurent. Une fois qu'une personne a contracté le virus Ebola, il peut se propager à d'autres personnes, en particulier si elles vivent sous le même toit.

QU'EST-CE QU'UN VACCIN ET QU'EST-CE QU'UNE ÉTUDE DE RECHERCHE CLINIQUE ?

Les vaccins sont des produits médicaux utilisés pour empêcher d'avoir certaines maladies. De nombreuses personnes sont vaccinées contre la rougeole, la polio et le choléra, par exemple.

Une étude de recherche clinique permet aux personnels de santé de comprendre de nouvelles façons de prévenir ou de traiter une maladie. Dans cette étude, le vaccin est encore sous test.

For peer review only - http://bmjopen.bmj.com/site/about/guidelines.xhtml

Les participants à des études de recherche sur les vaccins sont suivis pendant un certain temps afin de recueillir plus d'informations sur le vaccin.

D'AUTRES VACCINS SONT-ILS ADMINISTRÉS POUR PRÉVENIR LE VIRUS EBOLA EN RDC ?

Des vaccinations sont en cours avec un vaccin appelé « VSV » dans plusieurs endroits comme Beni, Katwa et Butembo. Ce vaccin est administré à des personnes à risque de contracter le virus Ebola, comme celles en contact avec une personne infectée par le virus Ebola ou avec des personnes en contact avec celle-ci. Il est également administré à des professionnels de la santé et d'autres travailleurs qui peuvent entrer en contact avec le virus Ebola. Cependant, environ la moitié des personnes infectées par le virus Ebola en RDC ne sont pas connues pour avoir été en contact avec un cas d'Ebola ; nous devons donc les protéger également. Par conséquent, nous cherchons d'autres moyens de protéger les gens contre Ebola, y compris ce vaccin.

EN QUOI CONSISTE CETTE ÉTUDE ?

Cette étude va déterminer si un autre vaccin peut aider à protéger la population contre Ebola. Ce nouveau vaccin est administré en deux injections. La première injection est une dose d'un vaccin appelé Ad26.ZEBOV. La seconde injection est administrée environ deux mois plus tard, avec une deuxième dose appelé MVA-BN-Filo. Dans cette étude, le vaccin « Janssen Ebola » désignera ces deux injections de deux vaccins différents.

Dans le reste de ce document, nous allons parler de vous. Le mot « vous » peut se rapporter à vous ou à votre enfant. Si vous êtes le parent ou le gardien d'un enfant de moins de 18 ans et si vous souhaitez que votre enfant participe à cette étude, vous devrez signer des documents distincts pour vous et votre enfant.

Si votre enfant est âgé de 12 à 17 ans, il/elle devra également accepter de participer à l'étude lui-même/elle-même et signer un autre document, appelé formulaire d'assentiment.

QUE SAVONS-NOUS DU VACCIN ?

Le vaccin Janssen Ebola est conçu pour protéger la population contre Ebola. **Vous ne pouvez pas contracter le virus Ebola par le vaccin.** Plus de 6 000 personnes ont reçu le vaccin dans de nombreux pays, dont 7 en Afrique. Jusqu'à présent, le vaccin est sûr et bien toléré chez les personnes vaccinées. Le vaccin pourrait aider le corps à lutter contre les infections à virus Ebola, mais nous ne savons pas encore avec certitude si le vaccin Janssen Ebola peut protéger la population contre Ebola.

QUI PEUT PARTICIPER À CETTE ÉTUDE ?

Pour participer à cette étude, vous devez vivre ou travailler au sein de l'une des communautés qui recevront le vaccin Janssen Ebola. Vous devez être en mesure de revenir pour recevoir la deuxième dose de vaccin et être disposé(e) à respecter les exigences nécessaires pour prendre part à cette étude. Les participants doivent être en bonne santé afin de recevoir chaque dose du

For peer review only - http://bmjopen.bmj.com/site/about/guidelines.xhtml

vaccin. Les personnes avec un diagnostic de VIH (qui sont en bonne santé), les femmes enceintes et allaitantes et les enfants âgés de 1 an ou plus peuvent être inclus dans cette étude.

Vous ne pouvez pas participer à cette étude si vous avez contracté la MVEbola.Vous ne pouvez pas non plus participer à cette étude si vous êtes allergique aux œufs ou aux produits contenant des œufs. Si vous avez été déjà vacciné contre la maladie à virus Ebola ou un autre vaccin de routine durant les 30 derniers jours ou si vous avez une fièvre de 38 °C ou plus le jour de la vaccination, vous devrez revenir un autre jour. Vous pouvez recevoir le vaccin si vous avez un léger rhume, de la toux ou une diarrhée légère.

SUIS-JE OBLIGÉ(E) DE PARTICIPER À CETTE ÉTUDE ?

Vous n'êtes pas obligé(e) de participer à cette étude si vous ne le souhaitez pas. Si vous décidez que vous ne souhaitez pas participer à l'étude, cela ne vous affectera en aucune façon. Si vous ne souhaitez pas participer à l'étude, mais que vous changez d'avis plus tard, vous pouvez participer à l'étude lorsque l'équipe de vaccination revient dans votre communauté. Après cette deuxième visite au sein de votre communauté, il peut ne plus être possible de participer à l'étude pour recevoir le vaccin Janssen Ebola. Vous devez être inclus dans l'étude pour recevoir le vaccin.

QUE SE PASSERA-T-IL SI J'ACCEPTE DE PARTICIPER À CETTE ÉTUDE ?

Nous allons décrire l'étude et répondre à toutes les questions que vous pourriez avoir. Si vous souhaitez une copie de cette note d'information, nous vous la donnerons. L'un des membres du personnel de l'étude vous posera des questions au sujet de l'étude pour vérifier que vous avez compris l'information. Nous vous demanderons de signer ou d'apposer votre empreinte digitale sur ce formulaire de consentement éclairé. En mettant votre nom ou en apposant votre empreinte digitale sur le formulaire de consentement, vous acceptez de participer à l'étude, mais vous pouvez changer d'avis et décider de quitter l'étude à tout moment.

QU'IMPLIQUE L'ÉTUDE ?

Après avoir signé ce formulaire de consentement, on vous posera quelques questions pour vérifier comment vous vous sentez. Si le médecin décide que vous pouvez être vacciné(e), vous recevrez une injection du premier vaccin Janssen Ebola contre Ebola dans la partie supérieure de votre bras ou dans la cuisse chez les jeunes enfants. Nous vous donnerons une carte de vaccination avec le numéro de téléphone de personnes à contacter en cas de besoin. La carte indiquera également la date à laquelle vous devrez revenir pour la deuxième dose de vaccin Janssen Ebola. Votre numéro de téléphone vous sera demandé, il est possible que vous soyez contacté afin de vous rappeler le rendez-vous de l'administration du second vaccin et également pour assurer un suivi.

Afin de vous identifier lors de la visite suivante, nous allons prendre une photographie de votre visage. Nous le faisons afin de savoir qui a été vacciné et veiller que vous receviez la deuxième dose au bon moment.

Vous serez invité(e) à vous présenter à une date spécifique pour le second vaccin Janssen Ebola. Après la deuxième dose, nous vous demanderons de nous appeler si vous avez des problèmes

For peer review only - http://bmjopen.bmj.com/site/about/guidelines.xhtml

médicaux dans les 30 jours après votre deuxième dose de vaccin. Vous devrez signaler toute maladie à l'équipe de l'étude dans les 30 jours en contactant le numéro de téléphone indiqué dans votre carte de vaccination. Nous veillerons à ce que des téléphones soient disponibles au sein de votre communauté.

Un certain nombre de participants seront inclus dans un groupe de suivi rapproché qui sera fait par téléphone ou par visite à domicile.

Suite à la pause de l'étude due à la COVID-19, certains participants ont vu leur deuxième dose de vaccin Janssen Ebola reportée. Ces participants seront appelés à recevoir leur deuxième dose à une date ultérieure. Le fait de recevoir le second vaccin Janssen Ebola à une date ultérieure n'aura pas d'effets négatifs sur la santé de ces participants.

QUELS SONT LES ÉVENTUELS RISQUES LIÉS À LA PARTICIPATION À CETTE ÉTUDE ?

Nous ne savons pas encore avec certitude si le vaccin Janssen Ebola protège la population contre Ebola. Il existe donc un risque que vous ne soyez pas protégé(e) contre le virus Ebola, même après la vaccination.

Ce vaccin a été administré à plus de 6 000 personnes dans d'autres études de recherche. Certains des effets secondaires que vous pourriez avoir après les injections sont les suivants :

- Douleur, gonflement, démangeaison, rougeur ou sensation de chaleur autour de la zone où vous avez reçu l'injection.
- Autour du site de l'injection, vous pourriez ressentir de la douleur lors de vos mouvements pendant quelques jours.
- Vous pourriez remarquer des bosses gonflées sous vos bras ou dans votre nuque pendant quelques jours.
- Vous pourriez avoir de la fièvre ou des maux de tête, une sensation de fatigue, des frissons, une éruption cutanée, des nausées, une modification de l'appétit, des vomissements, de la diarrhée, des étourdissements, des douleurs musculaires, articulaires ou au ventre, des plaies dans la bouche ou des courbatures.

Dans les études de recherche précédentes, tout le monde n'a pas eu ces effets secondaires après les injections. Les effets secondaires sont généralement légers et la plupart ne durent que quelques jours. Chez certaines personnes, ils peuvent durer pendant quelques semaines, très rarement plus longtemps. Certaines personnes ont signalé qu'elles avaient des picotements dans les mains et les pieds, ou une faiblesse musculaire. Généralement, ces symptômes ne duraient qu'un jour ou deux, mais parfois ils perduraient pendant plusieurs semaines. Une personne a signalé des picotements, un engourdissement et une douleur qui ont duré pendant plusieurs mois et interféré sur ses activités quotidiennes.

Nous avons appris que trois femmes de moins de 40 ans vivant dans d'autres pays ont présenté de graves caillots sanguins dans les trois semaines suivant l'administration d'un vaccin fabriqué de manière similaire au deuxième vaccin Janssen Ebola. L'une d'entre elles a eu un accident vasculaire cérébral et est décédée. Un homme de 50 ans a également signalé un manque de flux sanguin dans la jambe. Ces cas présentaient également des conditions médicales connues pour augmenter le risque de caillots sanguins et aucun de ces événements

For peer review only - http://bmjopen.bmj.com/site/about/guidelines.xhtml

n'a été considéré comme lié au vaccin. Le fait de recevoir le vaccin n'augmentait pas les risques d'avoir ces problèmes.
Des centaines de bébés (âgés de 1 à 3 ans), d'enfants (âgés de 4 à 11 ans) et d'adolescents (âgés de 12 à 17 ans) ont reçu le vaccin Janssen Ebola jusqu'à présent. Les effets secondaires signalés chez les enfants et les adolescents après la vaccination étaient similaires à ceux signalés par les adultes. La douleur au point d'injection était l'effet secondaire local le plus fréquemment signalé. Des maux de tête, de la fatigue et des frissons ont également été fréquemment signalés chez les enfants et les adolescents plus âgés. Une diminution de l'appétit, une baisse d'activité et de la fièvre ont été les effets secondaires les plus fréquemment signalés chez les bébés. Après la vaccination, quelques enfants ont eu une fièvre supérieure à 39ºC. La plupart de ces effets secondaires étaient légers à modérés et n'ont pas duré plus d'un ou deux jours. Aucun problème médical grave n'a été observé chez les enfants qui ont reçu le vaccin. Les effets secondaires chez les enfants sont similaires à ceux qui se produisent avec d'autres vaccins chez les enfants.

L'équipe médicale vous fournira fournir des soins si vous ressentez des effets secondaires liés aux injections jusqu'au un mois après la seconde dose. Elle est disponible à tout moment si vous avez des problèmes ou des questions.

QUELS SONT LES AVANTAGES LIÉS À LA PARTICIPATION À L'ÉTUDE ?

Les jours de vaccination, un médecin vous posera des questions pour vérifier votre état de santé ; si nécessaire, vous recevrez les soins nécessaires ou serez dirigé vers un spécialiste pour des affections médicales simples comme le paludisme. Vous apprendrez aussi comment vous protéger contre le virus Ebola. Votre participation à cette étude contribuera au développement de vaccins pour prévenir le virus Ebola et, à l'avenir, pourrait aider des personnes dans différentes régions du monde.

GROSSESSE ET L'ALLAITEMENT PENDANT L'ÉTUDE ?

Vous pouvez prendre part à cette étude si vous êtes enceinte ou si vous allaitez. Nous ne sommes pas sûr de tous les effets des vaccins de l'étude pendant la grossesse ou chez un bébé allaité. Si vous savez que vous êtes enceinte ou si vous allaitez au moment de la vaccination, informez-en le personnel de l'étude. Si vous n'êtes pas certaine, nous vous proposerons un test de grossesse et un entretien avec du personnel médical qui vous informera si vous etes emceinte avant chaque vaccination. Si vous êtes enceinte, ou si vous tombez enceinte dans le mois qui suit l'administration d'une des deux doses, vous serez contactee durant votre grossesse afin de vous poser des questions concernant votre sante et celle votre bebe. Dans ce cas, nous vous contacterons pendant votre grossesse pour vous poser des questions sur votre état de santé et celui de votre bébé. Nous vous contacterons également dans les 3 mois après votre accouchement. Si nous ne pouvons pas vous contacter par téléphone après votre accouchement, l'équipe de l'étude vous rendra visite à votre domicile. Parmi les femmes enceintes dans l'étude, certain nombre de participante seront invitée à faire partie d'un sous-groupe pour assurer un suivi plus rapproché par téléphone ou au domicile. Certains nouveaux-nés de maman faisant partie du sous-groupe seront également suivis dans les 3 mois suivant la naissance
Si vous tombez enceinte dans le mois qui suit une vaccination, merci d'appeler le numéro de téléphone indiqué sur votre carte de vaccination et de prévenir l'équipe de l'étude.

For peer review only - http://bmjopen.bmj.com/site/about/guidelines.xhtml

QUE DOIS-JE FAIRE POUR MA PROPRE SANTÉ PENDANT CETTE ÉTUDE ?

Si vous tombez malade pendant l'étude, vous devez vous rendre dans l'un des établissements de santé recommandés qui sont indiqués sur la liste que nous vous donnerons. . Vous devrez également appeler le numéro de téléphone de la personne de contact de l'étude indiquée sur votre carte de vaccination et lui dire si vous présentez un problème médical, jusqu'à 30 jours après l'administration de la deuxième dose du vaccin. Vous pourrez être orienté(e) vers des soins médicaux, si nécessaire.

Nous ne savons pas encore avec certitude si le vaccin Janssen Ebola peut protéger contre Ebola. Si vous recevez le vaccin, vous pourriez être protégé(e) contre le virus Ebola, mais il est possible que vous ne soyez pas protégé(e). **Par conséquent, vous devez continuer à éviter les contacts avec le virus Ebola.** Nous vous donnerons des informations spécifiques sur la prévention du virus Ebola, et n'hésitez pas à contacter un membre de l'équipe de l'étude si vous avez des questions sur la façon d'éviter les contacts avec le virus Ebola. Si vous présentez des symptômes qui ressemblent au virus Ebola, comme de la fièvre, de la diarrhée, des vomissements ou des saignements inexpliqués, il est très important que vous informiez l'équipe de l'étude que vous êtes malade et que vous receviez des soins médicaux dès que possible.

Si nous découvrons quelque chose de nouveau pendant cette étude qui pourrait être lié à votre santé ou influencer votre décision de poursuivre l'étude, nous vous en parlerons.

QUI POURRA CONSULTER MES INFORMATIONS ?

Nous préserverons la confidentialité de vos informations de l'étude.
Le personnel de l'étude, y compris des personnes en dehors du pays chargées de vérifier le bon déroulement de cette étude, les membres des comités d'éthique et les autorités réglementaires, peuvent consulter vos dossiers médicaux. Tous les membres du personnel de l'étude sont formés à maintenir la confidentialité et ne supprimeront aucun document avec votre nom dans les dossiers de l'étude. Toutes ces personnes comprennent qu'elles doivent garder votre identité confidentielle et qu'elles ne partageront votre nom ou vos coordonnées avec personne.

Une description de cette étude sera disponible sur le site internet http://www.ClinicalTrials.gov. Ce site internet ne contiendra aucune information permettant de vous identifier. Vous pouvez consulter ce site internet à tout moment.

Les résultats de l'étude seront partagés avec la population, mais aucune information personnelle ne sera mentionnée dans les résultats de l'étude. Si vous avez des questions spécifiques sur vos données, contactez l'investigateur principal de l'étude, le Professeur Jean Jacques Muyembe ou son représentant (voir les coordonnées ci-dessous).

QUI VÉRIFIE QUE L'ÉTUDE EST SANS DANGER ?

Cette étude est mise en œuvre par des partenaires, comme le Ministère de la Santé de la RDC par l'intermédiaire de l'Institut national de recherche biomédicale (INRB) avec l'aide d'Epicentre et de la London School of Hygiene & Tropical Medicine (LSHTM). Un comité

For peer review only - http://bmjopen.bmj.com/site/about/guidelines.xhtml

indépendant composé de médecins et d'experts qui ne sont pas directement impliqués dans l'étude examinera les informations de l'étude et vérifiera l'innocuité des vaccins.

À QUI PUIS-JE PARLER DE CETTE ÉTUDE ?

Si vous souhaitez parler à quelqu'un à propos de cette étude ou si vous pensez avoir subi un préjudice en lien avec votre participation à l'étude, vous pouvez contacter les personnes suivantes.

1) L'investigateur principal responsable de cette étude

Professeur Jean Jacques Muyembe<br>*Directeur général,* Institut national de recherche biomédicale, Kinshasa, RDC<br>Téléphone : 0898 949 289<br>E-mail : jjmuyembet@gmail.com<br><br>Représentant locale de l'étude<br>Dr Hugo Kavunga<br>Téléphone: 0823 875 153<br>E-mail : hugokavunga@gmail.com

2) Les comités d'éthique de la RDC qui ont approuvé cette étude

Professeur Félicien Munday<br>Comité national d'éthique et de la santé<br>Kinshasa-Gombe, RDC<br>Téléphone : 0998 419 816<br>E-mail : feli1munday@yahoo.fr
Professeur Willy Bongopasi<br>Comité d'éthique de l'école de santé publique<br>Université de Kinshasa, RDC<br>Téléphone: 0999 952 341<br>E-mail : bongopasi@gmail.com

Si vous avez des questions concernant l'étude TUJIOKOWE ou sur vos droits en tant que participant(e) à l'étude, vous pouvez les poser à un membre de l'équipe de l'étude à tout moment.

FORMULAIRE DE CONSENTEMENT DESTINÉ AU/À LA PARTICIPANT(E) OU AU PARENT/TUTEUR

Titre : Étude TUJIOKOWE

Investigateur principal de cette étude : Prof. JJ Muyembe

For peer review only - http://bmjopen.bmj.com/site/about/guidelines.xhtml

**Déclarations**	**Signez ou apposez votre empreinte digitale dans chaque case**
J'ai lu les informations contenues dans ce formulaire concernant l'étude TUJIOKOWE (ou le personnel de l'étude m'a expliqué ces informations avec des mots que je comprends). Le but de cette étude et les procédures pour recevoir ce nouveau vaccin m'ont été expliqués en détail. J'ai pu poser des questions et j'ai reçu des réponses satisfaisantes à toutes mes questions.	
Je comprends que ma participation/la participation de mon enfant est volontaire, que je peux retirer mon consentement/mon enfant peut retirer son consentement et quitter cette étude à tout moment sans avoir à me/se justifier, et que cela n'affectera pas mes soins médicaux/les soins médicaux de mon enfant ni mes droits légaux/les droits légaux de mon enfant.	
J'ai été informé(e) que le nouveau vaccin Janssen Ebola est en cours d'étude, et que les risques possibles liés à l'administration de ce vaccin ne sont pas tous connus.	
Je confirme que je n'ai/mon enfant n'a **PAS** contracté le virus Ebola ou été vacciné(e) contre Ebola au cours des 30 derniers jours.	
J'accepte que l'équipe de l'étude me photographie/photographie mon enfant avant la vaccination.	
Si j'ai un numéro de téléphone, j'accepte de le donner à l'équipe de l'étude et j'accepte d'être contacté par appel ou SMS pour l'administration de la seconde dose ainsi que pour le suivi (ex. questions de santé).<br>Si je n'ai pas de téléphone, j'accepte dans la mesure du possible de fournir le numéro d'un proche ou d'un ami qui pourrait être utilisé pour me joindre.	
Je comprends que les informations recueillies à mon sujet/au sujet de mon enfant seront utilisées pour étayer d'autres recherches à l'avenir et peuvent être partagées de manière anonyme avec d'autres chercheurs.	
J'accepte que mes informations médicales/les informations médicales de mon enfant qui ne contiennent pas mon/son nom, puissent être partagées avec les autorités sanitaires nationales, le fabricant du vaccin (Janssen Vaccines & Prevention B.V.) et d'autres organismes étrangers en dehors de la RDC, comme la Food & Drug Administration aux Etats-Unis. J'autorise le personnel de ces organismes à consulter et analyser mes dossiers/les dossiers de mon enfant et les publier dans des revues scientifiques.	

FORMULAIRE DE CONSENTEMENT DESTINÉ AU/À LA PARTICIPANT(E) OU AU PARENT/TUTEUR

Déclarations concernant les procédures facultatives

**Déclarations**	**Signez ou apposez votre empreinte digitale dans chaque case si oui**	**Signez ou apposez votre empreinte digitale dans chaque case si non**

For peer review only - http://bmjopen.bmj.com/site/about/guidelines.xhtml

(Pour les femmes participantes uniquement) J'accepte que, si je suis/ma fille est enceinte au moment de la vaccination ou devient enceinte pendant le premier mois après avoir reçu chaque vaccin, et que je suis/ma fille est sélectionnée par l'équipe de l'étude pour faire partie du groupe femme enceinte, l'équipe de l'étude puisse me contacter plus frequement que les autres femmes enceintes incluses dans l'étude par téléphone, pendant ma grossesse/la grossesse de ma fille et après mon accouchement/l'accouchement de ma fille et pourrait passer à mon domicile/au domicile de ma fille si je/ma fille ne peut pas être contactée par téléphone.		
J'accepte que si je/mon enfant est choisi par l'équipe d'étude pour faire parti au groupe de suivi de l'innocuité, l'équipe d'étude peut me contacter par téléphone après ma deuxième vaccination, et peut me voir/mon enfant à la maison si je ne peux pas être contacté par téléphone.		

POUR LES PARTICIPANTS ADULTES

Je suis âgé(e) de 18 ans ou plus et j'accepte de participer à cette étude. (Signez ou apposez votre empreinte digitale ci-dessous)

		____/____/_____ *jj mmm aaaa*
Nom du/de la participant(e) adulte, en majuscules	Signature/Empreinte digitale du/de la participant(e) adulte	Date

POUR LE PARENT OU GARDIEN

Je suis le parent ou gardien d'un(e) participant(e) âgé(e) entre 1 et 17 ans et j'accepte que mon enfant participe à cette étude. (Signez ou apposez votre empreinte digitale ci-dessous)

		____/____/_____ *jj mmm aaaa*
Nom en caractères d'imprimerie du parent ou tuteur	Signature/Empreinte digitale du parent ou gardien	Date

POUR LE PARENT OU GARDIEN

For peer review only - http://bmjopen.bmj.com/site/about/guidelines.xhtml

____
Nom en caractères d'imprimerie du/de la participant(e) (si âgé[e] entre 1 et 17 ans)

		____/____/_____<br>*Jj mmm aaaa*
Nom de l'investigateur en caractères d'imprimerie	Signature de l'investigateur	Date

Remplissez la rubrique suivante si le/la participant(e) ou le parent/gardien est analphabète :

Témoin de l'entretien de consentement

J'ai assisté à l'entretien de consentement pour l'étude TUJIOKOWE dans ce document. J'atteste avoir expliqué les informations de l'étude avec précision au/à la participant(e) ou à son parent/tuteur et que ce dernier/cette dernière a compris, et qu'il/elle a donné librement son consentement pour participer/la participation de son enfant, en ma présence.

		____/____/_____<br>*jj mmm aaaa*
Nom du témoin impartial en caractères d'imprimerie	Signature du témoin impartial	Date

Apposez l'étiquette d'identification par code à barres ci-dessous :

For peer review only
For peer review only - http://bmjopen.bmj.com/site/about/guidelines.xhtml

Protocol for a phase 3 trial to evaluate the effectiveness and safety of a heterologous, two-dose vaccine for Ebola virus disease in the Democratic Republic of the Congo

Appendix 2A

Informed Assent Form in English, Version 7.1 (10 Aug 2020)

For peer review only

For peer review only - http://bmjopen.bmj.com/site/about/guidelines.xhtml

Evaluation of a heterologous, two-dose preventive Ebola vaccine for effectiveness and safety in the Democratic Republic of the Congo

"The TUJIOKOWE Study" Information Sheet and Informed Assent Form for Children aged 12 years or older

LSHTM Protocol : DRC-EB-001

Principal Investigator (PI):
Professor Jean Jacques Muyembe
Director-General
Institut National de Recherche Biomédicale
Professor of Microbiology, Kinshasa University Medical School
Kinshasa Gombe, Democratic Republic of the Congo (DRC)
Phone: 0898 949 289; Email: jjmuyembet@gmail.com

Sponsor: London School of Hygiene & Tropical Medicine, United Kingdom

Site: Democratic Republic of the Congo

INTRODUCTION

You are being invited to take part in a research study called the 'TUJIOKOWE study' to find out if a new two-dose vaccine called the 'Janssen Ebola vaccine' can protect from getting Ebola and to check whether the vaccine is safe. The TUJIOKOWE study is being implemented by the Ministry of Health of DRC through the Institut National de Recherche Biomédicale (INRB), Epicentre and the London School of Hygiene & Tropical Medicine (LSHTM).

WHAT IS EBOLA?

Ebola is a disease that makes some people become very sick, and about half of the people who catch Ebola die from it. People catch Ebola from other people who have the disease.

WHAT IS A VACCINE AND WHAT IS A CLINICAL RESEARCH STUDY?

Vaccines are medical products that are used to stop people from getting certain diseases. You may have had vaccines when you were a young child to prevent you from catching diseases like measles and polio.

Clinical research studies help health professionals to understand if medicines or vaccines work to stop or treat diseases.

ARE ANY OTHER VACCINES BEING GIVEN TO PREVENT EBOLA IN DRC?

For peer review only - http://bmjopen.bmj.com/site/about/guidelines.xhtml

Lots of activities are ongoing to try to stop Ebola. Some people are being given a vaccine called VSV if they have been close to or looked after someone with Ebola, but that doesn't include everyone who might get Ebola – so we want to see if this vaccine can help.

WHAT IS THIS STUDY ABOUT?

People are getting sick with Ebola in North Kivu and Ituri provinces.

This study is going to find out if another new vaccine called the Janssen Ebola vaccine can help protect people against Ebola.

WHAT DO WE KNOW ABOUT THE VACCINE?

The Janssen Ebola vaccine is designed to protect people against Ebola. **It cannot make you sick with Ebola.** Over 6000 people have received the vaccine in many different countries, including7 in Africa. So far, the vaccine is safe in people who received it. The vaccine may help you fighting Ebola, but we don't know for sure yet if the Janssen Ebola vaccine can protect people against Ebola.

WHO CAN TAKE PART IN THIS STUDY?

To be given the Janssen Ebola vaccine, your parent or guardian must agree that you can join the study. You should also decide for yourself whether you want to join the study. You must be living in one of the communities chosen to be included in the study. You should be feeling well in order to get the vaccine.

People who have had Ebola cannot be in the TUJIOKOWE study. If you have received another Ebola vaccine or routine vaccine within the last month (30 days), or if you are sick on the day of vaccination, you may be asked to come back another time.

DO I HAVE TO BE IN THIS STUDY?

You do not have to join this study if you don't want to, even if your parents or guardians want you to join. If you agree to be in the study, we will ask you to sign this assent form. You can decide that you don't want to join, and it will not affect you in any way. If you don't want to be in the study at the moment, but you change your mind later, you can join the study when the vaccination team comes back. After that second visit by the team to your community, it may not be possible to get the Janssen Ebola vaccine. You need to be in the study to get this vaccine.

WHAT WILL HAPPEN IF I AGREE TO TAKE PART IN THIS STUDY?

Putting your name or your fingerprint on this assent form means that you agree to be in the study. You can change your mind and choose to leave the TUJIOKOWE study at any time.

For peer review only - http://bmjopen.bmj.com/site/about/guidelines.xhtml

You will be asked some questions about the study by one of the study staff members to check that you have understood the information.

WHAT DOES THE STUDY INVOLVE?

After you have signed this assent form, you will be asked some questions to check how you are feeling. If the doctor decides that you can be vaccinated, you will get an injection of the first Janssen Ebola vaccine against Ebola into your upper arm. We will give your parents or guardians a vaccination card with a phone number of who to contact in case you feel sick at any time after your visit. The vaccination card will also show the date of when you need to come back for the second Janssen Ebola vaccine. We will ask you for a phone number and we may text or call you for the second vaccine and follow ups.

In order to identify you at the next visit, we will take photographs of your face. We do this to make sure we know who has gotten the vaccine and that you receive the second dose at the right time.

A certain number of participants will be included in a group for follow up who will be contacted by phone or a home visit.

As a result of the study pause due to COVID-19, some participants had their second vaccination with the Janssen Ebola vaccine postponed. These participants will be contacted to receive their second vaccination at a later date. Receiving the second Janssen Ebola vaccine at a later date will not have negative effects on the health of these participants.

WHAT ARE THE POSSIBLE RISKS OF BEING IN THIS STUDY?

We do not know for sure yet if the Janssen Ebola vaccine protects people from Ebola, so there is a risk that you will not be protected from Ebola even after you have taken the vaccine.

As we have mentioned, this vaccine has been given to many people in other studies.

Some of the side effects that you may have after the injections are:

- pain, swelling, itchiness, redness, or warmth around the place where you got the injection and you may have a small sore which can happen after any other vaccine.
- in the area around the injection it may be sore and a bit painful to move for a few days.
- you may notice some swelling in your armpit or in your neck.
- you may get a fever or headache, feel tired, feel a bit sick or have some other side effects.

Not everyone gets these side effects and they should only last a few days. In a few people they may last for a few weeks and very rarely a few months.

In previous research studies some people have reported that they felt some tingling in the hands and feet, or their muscles felt weak. These symptoms usually only lasted one or two days, but sometimes for several weeks. One person reported tingling, numbness and pain which lasted for several months and interfered with the person's daily activities.

Very few people under 40 years old with other medical problems have had blood clots about 3

For peer review only - http://bmjopen.bmj.com/site/about/guidelines.xhtml

weeks after receiving a vaccine similar to the one we are giving; one woman died and one man had problems with his leg. These problems were not thought to be related to having the vaccine.

Hundreds of infants, children and teenagers have been given the Janssen Ebola vaccine. The most common problem reported by children is pain at the place where the injection was given. Older children and teenagers sometimes say they have headache, tiredness or can feel cold. Infants sometimes have less appetite or are less energetic or may have a fever for a short time. All these problems in children are the same as those after getting other vaccines and usually get better within 2 days. No serious medical problems have been reported in children who have been given the Janssen Ebola vaccine.

The medical team will help you if you don't feel well up until 1 month after your second injection. The medical team is available at any time if you have problems or questions.

WHAT ARE THE BENEFITS OF BEING IN THIS STUDY?

On the vaccination days a doctor will check your health status and you may receive treatment or a referral for simple medical conditions like malaria. You will also learn how to protect yourself against Ebola. Your participation in this study will help in the development of vaccines to prevent Ebola and, in the future, may help people in different parts of the world.

WHAT ABOUT PREGNANCY AND/OR BREASTFEEDING DURING THE STUDY?

Some older girls may be pregnant or breast feeding but they can still be in the study. We do not know all the effects of the Janssen Ebola vaccine in pregnancy or in a baby that is being breastfed. If you know you are pregnant or breastfeeding at the time of vaccination, please tell the study staff. If you are not sure, we will offer you a pregnancy test and meeting with a doctor so you can know whether you are pregnant before each injection. If you are pregnant or become pregnant within one month of each vaccination, we will contact you during your pregnancy until 3 months after delivery to ask about your health and the health of your baby. If we cannot contact you by phone after giving birth, the study team will visit you at your home. Among the pregnant women in the study, a number will be invited to join a subset and monitored more closely by telephone or through home visits. Some babies of women in this subset will also be seen within 3 months after birth. If you become pregnant within a month of vaccination, please call the phone number on your vaccination card and notify the study team.

WHAT DO I NEED TO DO FOR MY OWN HEALTH DURING THIS STUDY?

We will ask your parents or guardians to take you to a recommended health facility if you feel sick at any time during the study. They may also call the study contact number on your vaccination card to get advice up until 30 days after you receive the second dose of vaccine. You may be referred for medical care if necessary.

We do not know for sure yet if the Janssen Ebola vaccine can protect people against Ebola. **Therefore, you must continue to follow recommendations to protect yourself from Ebola.** We will give you information on how to prevent Ebola. If you experience anything like fever, diarrhea, vomiting, or unexplained bleeding, it is very important that you or your parents or

For peer review only - http://bmjopen.bmj.com/site/about/guidelines.xhtml

guardians let the study team know that you are sick and that you also get medical care as soon as possible.

WHO WILL BE ABLE TO SEE MY INFORMATION?

We will keep any information that you give us private.

Staff working on the study, as well as representatives of the ethics committee, including staff overseas, may look at your medical records. All of these people are trained to understand that they must keep your name and other details private.

The results of the study will be published in scientific journals so that other physicians and researchers can deepen their knowledge. It will not be possible to identify you.

If you have any specific questions about your data, please contact the study Principal Investigator, Professor Jean Jacques Muyembe or his representative (see contact details below).

WHO IS CHECKING THAT THE STUDY IS SAFE?

This study is being implemented by partners including the Ministry of Public Health of DRC through the Institut National de Recherche Biomédicale (INRB), Epicentre and the London School of Hygiene & Tropical Medicine (LSHTM). An independent committee made up of doctors and experts who are not directly involved in the study will be looking at the study information and checking that the study is safe.

For peer review only

For peer review only - http://bmjopen.bmj.com/site/about/guidelines.xhtml

WHO CAN I TALK TO ABOUT THIS STUDY?

If you or your parents or guardians would like to talk to someone about the TUJIOKOWE study or if you think you have been harmed by being part of the study, then your parents or guardians can contact the following people:

1) The Principal Investigator responsible for this study

Professor Jean Jacques Muyembe<br>*Director General*, Institut National de Recherche Biomédicale, Kinshasa, DRC<br>Phone: 0898 949 289<br>Email: jjmuyembet@gmail.com<br><br>Local study representative<br>Dr Hugo Kavunga<br>Phone: 0823 875 153<br>Email: hugokavunga@gmail.com

2) The DRC ethics committees that approved this study

Professor Félicien Munday<br>Comité National d’Ethique et de la Santé<br>Kinshasa-Gombe, DRC<br>Phone: 0998 419 816<br>Email: feli1munday@yahoo.fr
Professeur Willy Bongopasi<br>Comité d'éthique de l'école de santé Publique<br>Université de Kinshasa<br>Téléphone: 0999 952 341<br>Email: bongopasi@gmail.com

If you or your parents or guardians have any questions about the TUJIOKOWE study, you may ask someone on the study team at any time.

For peer review only - http://bmjopen.bmj.com/site/about/guidelines.xhtml

PARTICIPANT ASSENT FORM

Title: TUJIOKOWE Study

Principal Investigator of this study: Prof. JJ Muyembe

**Statements**	**Please sign or fingerprint each box**
I understand the information in this form, and I was able to ask questions and all of my questions were answered to my satisfaction.	
I understand that joining this study is voluntary and I can choose to leave the study at any time without it affecting me.	
I have been informed that the new Janssen Ebola vaccine is still being tested, and that not all the possible risks from having this vaccine are completely known.	
I confirm that I have **NOT** previously had Ebola or been vaccinated against Ebola within the past 30 days	
I agree to have my photograph taken by the study team before vaccination.	
I agree to give the study team a contact phone number if I have one and to be contacted for the second vaccine and for follow up calls or SMS e.g. for health questions. If I don't have a phone, I agree to try and provide a relative or friend's number that can be used to contact me.	
I understand that the information collected about me will be used to support other research in the future and may be shared anonymously with other researchers.	
I agree that my medical information that will not contain my name, can be shared with the national health authorities, the vaccine manufacturer (Janssen Vaccines & Prevention B.V.) and other foreign organizations outside the DRC, such as the US Food & Drug Administration. I give permission for individuals in these organizations to access and analyse my records and publish them in scientific journals.	

For peer review only

PARTICIPANT ASSENT FORM

Declarations regarding optional procedures

**Statements**	**Please sign or fingerprint each box for yes**	**Please sign or fingerprint each box for no**
(For female participants only) I agree that if I am pregnant or become pregnant during the first month after vaccination and I am selected to join the pregnancy subset, I may be telephoned during my pregnancy more often than other pregnant participants in the study and after the birth of my baby to collect some information on my health and my baby's health. If this information cannot be collected by telephone, I agree that I may be visited at home by a member of the study team.		
I agree that if I am selected by the study team to be in the safety group, the study team may contact me by telephone after my second vaccination and may see me at home if I cannot be contacted by telephone.		

I agree to take part in this study. (Please sign or put your fingerprint below)

		____/____/_____ *dd mon yyyy*
Printed name of child	Signature/fingerprint of child	Date

		____/____/_____ *dd mon yyyy*
Printed name of investigator	Signature of investigator	Date

For peer review only - http://bmjopen.bmj.com/site/about/guidelines.xhtml

Complete if participant is illiterate:

Witness to Assent Interview

I witnessed the assent interview for the TUJIOKOWE Study in this document. I attest that I have explained the study information accurately to the participant and was understood to the best of my knowledge by the participant, and that he/she has freely given their assent to participate in my presence.

		____/____/_____ *dd mon yyyy*
Printed name of impartial witness	Signature of impartial witness	Date

Attach ID barcode label below:

For peer review only - http://bmjopen.bmj.com/site/about/guidelines.xhtml

Protocol for a phase 3 trial to evaluate the effectiveness and safety of a heterologous, two-dose vaccine for Ebola virus disease in the Democratic Republic of the Congo

Appendix 2B

Informed Assent Form in French, Version 7.1 (10 Aug 2020)

For peer review only

For peer review only - http://bmjopen.bmj.com/site/about/guidelines.xhtml

Évaluation de l'efficacité et de l'innocuité de l'emploi d'un vaccin préventif hétérologue à deux doses contre Ebola en République démocratique du Congo

Note d'information et formulaire d'assentiment éclairé de l'« étude TUJIOKOWE » destinés aux enfants âgés de 12 ans ou plus

Protocole LSHTM : DRC-EB-001

Investigateur principal (IP) :
Professeur Jean Jacques Muyembe
Directeur général
Institut national de recherche biomédicale
Professeur de microbiologie, Faculté de médecine de l'Université de Kinshasa
Kinshasa Gombe, République démocratique du Congo (RDC)
Téléphone : 0898 949 289 ; E-mail : jjmuyembet@gmail.com

Promoteur : London School of Hygiene & Tropical Medicine, Royaume-Uni

Site : République démocratique du Congo

INTRODUCTION

Tu es invité(e) à participer à une étude de recherche appelée l'« étude TUJIOKOWE » pour savoir si un nouveau vaccin à deux doses appelé « vaccin Janssen Ebola » peut protéger contre le virus Ebola et pour vérifier si le vaccin est sans danger. L'étude TUJIOKOWE est mise en œuvre par le ministère de la santé publique de la RDC par l'intermédiaire de l'Institut national de recherche biomédicale (INRB), d'Epicentre et de la London School of Hygiene & Tropical Medicine (LSHTM).

QU'EST-CE QUE LE VIRUS EBOLA ?

Ebola est une maladie qui rend certaines personnes très malades, et environ la moitié des personnes qui contractent le virus Ebola en meurent. Le virus Ebola est transmis par des personnes atteintes de la maladie.

QU'EST-CE QU'UN VACCIN ET QU'EST-CE QU'UNE ÉTUDE DE RECHERCHE CLINIQUE ?

Les vaccins sont des produits médicaux utilisés pour empêcher les personnes d'avoir certaines maladies. Tu peux avoir eu des vaccins lorsque tu étais un jeune enfant pour te protéger de maladies comme la rougeole et la polio.

For peer review only - http://bmjopen.bmj.com/site/about/guidelines.xhtml

Les études de recherche clinique aident les personnels de santé à comprendre si les médicaments ou les vaccins agissent pour arrêter ou traiter les maladies.

D'AUTRES VACCINS SONT-ILS ADMINISTRÉS POUR PRÉVENIR LE VIRUS EBOLA EN RDC ?

De nombreuses activités sont en cours pour essayer d'arrêter le virus Ebola. Certaines personnes reçoivent un vaccin appelé le vaccin VSV si elles ont été en contact avec une personne infectée par Ebola ou si elles s'en sont en contact avec quelqu'un qui s'occupe des malades, mais cela n'inclut pas tous ceux qui peuvent avoir Ebola dans l'avenir - alors nous voulons voir si ce vaccin peut aider.

EN QUOI CONSISTE CETTE ÉTUDE ?

Dans les provinces du Nord-Kivu et d'Ituri, des individus sont infectés par le virus Ebola.

Cette étude va déterminer si un autre nouveau vaccin, le vaccin Janssen Ebola, peut aider à protéger contre Ebola.

QUE SAVONS-NOUS DU VACCIN ?

Le vaccin Janssen Ebola est conçu pour protéger la population contre Ebola. **Tu ne peux pas contracter le virus Ebola à cause du vaccin.** Plus de 6 000 personnes ont reçu le vaccin dans de nombreux pays, dont 7 en Afrique. Jusqu'à présent, le vaccin est sûr chez les personnes vaccinées. Le vaccin pourrait t'aider lutter contre les infections à virus Ebola, mais nous ne savons pas encore avec certitude si le vaccin Janssen Ebola peut protéger contre Ebola.

QUI PEUT PARTICIPER À CETTE ÉTUDE ?

Pour recevoir le vaccin Janssen Ebola, tes parents/gardiens doivent accepter que tu participes à l'étude. Tu dois également décider si tu souhaites participer à l'étude. Pour être inclus(e) dans l'étude, tu dois faire partie de l'une des communautés sélectionnées. Tu dois te sentir bien afin de recevoir le vaccin.

Les personnes qui ont été atteintes du virus Ebola ne peuvent pas participer à l'étude TUJIOKOWE.

Si tu as été déjà vacciné contre la maladie à virus Ebola ou un autre vaccin de routine durant les 30 derniers jours ou si tu es malade le jour de vaccination, tu seras vacciné un autre jour.

SUIS-JE OBLIGÉ(E) DE PARTICIPER À CETTE ÉTUDE ?

Tu n'es pas obligé(e) de participer à cette étude si tu ne veux pas le faire, même si tes parents ou tes gardiens veulent que tu y participes. Si tu acceptes de participer à l'étude, nous te demanderons de signer ce formulaire d'assentiment. Tu peux décider que tu ne souhaites pas participer et cela n'aura aucun effet sur toi. Si tu ne souhaites pas participer à l'étude pour le moment, mais que tu changes d'avis plus tard, tu peux participer à l'étude lorsque l'équipe de vaccination revient dans ta communauté. Après cette deuxième visite au sein de ta

For peer review only - http://bmjopen.bmj.com/site/about/guidelines.xhtml

communauté, il peut ne plus être possible de recevoir le vaccin Janssen Ebola. Il faut être dans l'étude pour recevoir le vaccin.

QUE SE PASSERA-T-IL SI J'ACCEPTE DE PARTICIPER À CETTE ÉTUDE ?

En mettant ton nom ou en apposant ton empreinte digitale sur ce formulaire d'assentiment, tu acceptes de participer à l'étude. Tu peux changer d'avis et décider de quitter l'étude Janssen Ebola à tout moment. L'un des membres du personnel de l'étude te posera des questions sur l'étude pour vérifier que tu as compris l'information.

QU'IMPLIQUE L'ÉTUDE ?

Après avoir signé ce formulaire d'assentiment, on te posera quelques questions pour vérifier comment tu te sens. Si le médecin décide que tu peux être vacciné(e), tu recevras une injection de la première dose de vaccin de Janssen Ebola contre Ebola dans la partie supérieure de ton bras. Nous donnerons à tes parents ou gardiens une carte de vaccination avec le numéro de téléphone de personnes à contacter au cas où tu te sens mal, à tout moment après ta visite. La carte de vaccination indiquera également la date à laquelle tu devras revenir pour la deuxième dose de vaccin Janssen Ebola. Votre numéro de téléphone vous sera demandé, il est possible que vous soyez contacté afin de vous rappeler le rendez-vous de l'administration du second vaccin et également pour assurer un suivi.

Afin de t'identifier lors de la visite suivante, nous allons prendre une photographie de ton visage. Nous le faisons afin de veiller à ce que tu reçoives la deuxième dose au bon moment et pour savoir qui a été vacciné.

Un certain nombre de participants seront inclus dans un groupe de suivi qui sera contacté par téléphone ou une visite à domicile.
Suite à la pause de l'étude due à la COVID-19, certains participants ont vu leur deuxième vaccination avec le vaccin Janssen Ebola reportée. Ces participants seront contactés pour recevoir leur deuxième vaccination à une date ultérieure. Le fait de recevoir le deuxième vaccin Janssen Ebola à une date ultérieure n'aura pas d'effets négatifs sur la santé de ces participants.

QUELS SONT LES ÉVENTUELS RISQUES LIÉS À LA PARTICIPATION À CETTE ÉTUDE ?

Nous ne savons pas encore avec certitude si le vaccin Janssen Ebola protège la population contre Ebola. Il existe donc un risque que tu ne sois pas protégé(e) contre le virus Ebola, même après la vaccination.

Comme nous l'avons mentionné, ce vaccin a été administré à de nombreuses personnes dans d'autres études.

Certains des effets secondaires que tu pourrais avoir après les injections sont les suivants :

- Douleur, gonflement, démangeaisons, rougeur ou sensation de chaleur autour de l'endroit où tu as reçu l'injection et tu pourrais être un peu endolori(e) comme après tout vaccin.

For peer review only - http://bmjopen.bmj.com/site/about/guidelines.xhtml

- Au site autour de l'injection, tu pourrais ressentir de la douleur lors de tes mouvements pendant quelques jours.
- Tu pourrais remarquer un gonflement sous ton bras ou dans ton cou.
- Tu pourrais avoir de la fièvre ou des maux de tête, une sensation de fatigue, te sentir un peu mal ou avoir d'autres effets secondaires.

Tout le monde ne présente pas ces effets secondaires, et ils ne durent que quelques jours. Chez certaines personnes, ils peuvent durer pendant quelques semaines et très rarement quelques mois.

Dans des études de recherche précédentes, certaines personnes ont signalé qu'elles avaient des picotements dans les mains et les pieds, ou une faiblesse musculaire. Généralement, ces symptômes ne duraient qu'un jour ou deux, mais parfois ils ont duré pendant plusieurs semaines. Une personne a signalé des picotements, un engourdissement et une douleur qui ont duré pendant plusieurs mois et interféré avec ses activités quotidiennes.

Très peu de personnes de moins de 40 ans ayant d'autres problèmes médicaux ont eu des caillots de sang environ 3 semaines après avoir reçu un vaccin similaire à celui que nous administrons ; une femme est morte et un homme a eu des problèmes à la jambe. Ces problèmes n'ont pas été considérés comme étant liés à l'administration du vaccin.

Des centaines de nourrissons, d'enfants et d'adolescents ont reçu le vaccin Janssen Ebola. Le problème le plus fréquent signalé par les enfants est la douleur à l'endroit où l'injection a été faite. Les enfants plus âgés et les adolescents disent parfois avoir mal à la tête, être fatigués ou avoir froid. Les nourrissons ont parfois moins d'appétit ou sont moins énergiques ou peuvent avoir de la fièvre pendant une courte période. Tous ces problèmes chez les enfants sont les mêmes qu'après avoir reçu d'autres vaccins et se résorbent généralement en deux jours. Aucun problème médical grave n'a été signalé chez les enfants qui ont reçu le vaccin Janssen Ebola.

L'équipe médicale vous aidera si vous ne vous sentez pas bien jusqu'à 1 mois après votre deuxième injection. Elle est disponible à tout moment si vous avez des problèmes ou des questions.

QUELS SONT LES AVANTAGES LIÉS À LA PARTICIPATION À L'ÉTUDE ?

Les jours d'administration du vaccin, un médecin vérifiera ton état de santé et tu pourrais recevoir un traitement ou être redirigé vers un spécialiste pour des affections médicales simples comme le paludisme. Tu apprendras aussi comment te protéger contre le virus Ebola. Ta participation à cette étude contribuera au développement de vaccins pour prévenir le virus Ebola et, à l'avenir, pourrait aider des personnes dans différentes régions du monde.

QU'EN EST-IL DE LA GROSSESSE ET/OU DE L'ALLAITEMENT PENDANT L'ÉTUDE ?

Certaines filles plus âgées peuvent être enceintes ou allaiter, mais elles peuvent toujours

For peer review only - http://bmjopen.bmj.com/site/about/guidelines.xhtml

participer à l'étude. Nous ne sommes pas sûr de tous les effets des vaccins de l'étude pendant la grossesse ou chez un bébé allaité. Si vous savez que vous êtes enceinte ou si vous allaitez au moment de la vaccination, informez-en le personnel de l'étude. Si vous n'êtes pas certaine, nous vous proposerons un test de grossesse et un entretien avec du personnel médical qui vous renseignera avant chaque vaccination. Si vous êtes enceinte ou si vous tombez enceinte dans le mois qui suit l'administration d'une des deux doses, vous serez contactee durant votre grossesse afin de vous poser des questions concernant votre sante et celle votre bebe. Dans ce cas, nous vous contacterons pendant votre grossesse et juste après votre accouchement pour vous poser des questions sur votre état de santé et celui de votre bébé. Nous vous contacterons également dans les 3 mois après votre accouchement. Si nous ne pouvons pas vous contacter par téléphone après votre accouchement, l'équipe de l'étude vous rendra visite à votre domicile. Parmi les femmes enceintes dans l'étude, un certain nombre de participante seront invitée à faire partie d'un sous-groupe pour assurer un suivi plus rapproché par téléphone ou au domicile. Certains nouveaux-nés de maman faisant partie du sous-groupe seront également suivis dans les 3 mois suivant la naissance. Si vous tombez enceinte dans le mois qui suit une vaccination, merci d'appeler le numéro de téléphone indiqué sur votre carte de vaccination et de prévenir l'équipe de l'étude.

QUE DOIS-JE FAIRE POUR MA PROPRE SANTÉ PENDANT CETTE ÉTUDE ?

Nous allons demander à tes parents ou gardiens de t'emmener à un établissement de santé recommandé si tu te sens malade à tout moment pendant l'étude. Ils peuvent également appeler le numéro de contact de l'étude indiqué sur ta carte de vaccination pour obtenir des conseils et ce jusqu'à 30 jours après avoir reçu la seconde dose du vaccin. Tu pourrais être orienté(e) vers des soins médicaux si nécessaire.

Nous ne savons pas encore avec certitude si le vaccin Janssen Ebola peut protéger la population contre Ebola. **Par conséquent, tu dois continuer à suivre les recommandations pour te protéger contre le virus Ebola.** Nous te donnerons des informations sur la façon de prévenir le virus Ebola. Si tu présentes des symptômes comme de la fièvre, de la diarrhée, des vomissements ou des saignements inexpliqués, il est très important que toi ou tes parents/gardiens informiez l'équipe de l'étude que tu es malade et que tu reçoives des soins médicaux dès que possible.

QUI POURRA CONSULTER MES INFORMATIONS ?

Nous préserverons la confidentialité des informations que tu nous donnes.

Le personnel travaillant sur l'étude, y compris les personnes en dehors du pays, peuvent consulter ton dossier médical. Toutes ces personnes sont formées à la confidentialité et comprennent qu'elles doivent garder ton nom et d'autres informations privés.
Les résultats de l'étude seront publiés dans des revues scientifiques afin que d'autres médecins et chercheurs puissent approfondir leurs connaissances. Il ne sera pas possible de t'identifier.

Si tu as des questions spécifiques sur tes données, contacte l'investigateur principal de l'étude, le Professeur Jean Jacques Muyembe ou son représentant (voir les coordonnées ci-dessous).

For peer review only - http://bmjopen.bmj.com/site/about/guidelines.xhtml

QUI VÉRIFIE QUE L'ÉTUDE EST SANS DANGER ?

Cette étude est mise en œuvre par des partenaires, comme le Ministère de la Santé de la RDC par l'intermédiaire de l'Institut national de recherche biomédicale (INRB), d'Epicentre et de la London School of Hygiene & Tropical Medicine (LSHTM). Un comité indépendant composé de médecins et d'experts qui ne sont pas directement impliqués dans l'étude examinera les informations de l'étude et vérifiera la sûreté de l'étude.

For peer review only

For peer review only - http://bmjopen.bmj.com/site/about/guidelines.xhtml

À QUI PUIS-JE PARLER DE CETTE ÉTUDE ?

Si tes parents/gardiens ou toi souhaitez parler à quelqu'un de l'étude TUJIOKOWE ou si tu penses avoir subi un préjudice en lien avec ta participation à l'étude, tes parents ou gardiens peuvent contacter les personnes suivantes :

1) L'investigateur principal responsable de cette étude

Professeur Jean Jacques Muyembe<br>*Directeur général*, Institut national de recherche biomédicale, Kinshasa, RDC<br>Téléphone : 0898 949 289<br>E-mail : jjmuyembet@gmail.com<br><br>Représentant locale de l'étude<br>Dr Hugo Kavunga<br>Téléphone : 0823 875 153<br>E-mail : hugokavunga@gmail.com

2) Les comités d'éthique de la RDC qui ont approuvé cette étude

Professeur Félicien Munday<br>Comité National d'Ethique et de la Santé<br>Kinshasa-Gombe, RDC<br>Téléphone : 0998 419 816<br>E-mail : feli1munday@yahoo.fr
Professeur Willy Bongopasi<br>Comité d'éthique de l'école de santé publique<br>Université de Kinshasa, RDC<br>Téléphone : 0999 952 341<br>E-mail : bongopasi@gmail.com

Si tes parents/gardiens ou toi avez des questions concernant l'étude TUJIOKOWE, vous pouvez les poser à un membre de l'équipe de l'étude à tout moment.

For peer review only - http://bmjopen.bmj.com/site/about/guidelines.xhtml

FORMULAIRE D'ASSENTIMENT DU/DE LA PARTICIPANT(E)

Titre : Étude TUJIOKOWE

Investigateur principal de cette étude : Prof. JJ Muyembe

**Déclarations**	**Signe ou appose ton empreinte digitale dans chaque case**
Je comprends les informations contenues dans ce formulaire et j'ai pu poser des questions et toutes mes questions ont reçu une réponse satisfaisante.	
Je comprends que ma participation à cette étude est volontaire et que je peux choisir de quitter l'étude à tout moment sans que cela ne m'affecte.	
J'ai été informé(e) que le nouveau vaccin Janssen Ebola est en cours d'étude, et que les risques possibles liés à l'administration de ce vaccin ne sont pas tous connus.	
Je confirme que je n'ai **PAS** contracté le virus Ebola ou été vacciné(e) contre Ebola au cours des 30 derniers jours	
J'accepte que l'équipe de l'étude me photographie avant la vaccination.	
Si j'ai un numéro de téléphone, j'accepte de le donner à l'équipe de l'étude et j'accepte d'être contacté par appel ou SMS pour l'administration de la seconde dose ainsi que pour le suivi (ex. questions de santé). Si je n'ai pas de téléphone, j'accepte dans la mesure du possible de fournir le numéro d'un proche ou d'un ami qui pourrait être utilisé pour me joindre.	
Je comprends que les informations recueillies à mon sujet seront utilisées pour étayer d'autres recherches à l'avenir et peuvent être partagées de manière anonyme avec d'autres chercheurs.	
J'accepte que mes informations médicales qui ne contiennent pas mon nom, puissent être partagées avec les autorités sanitaires nationales, le fabricant du vaccin (Janssen Vaccines & Prevention B.V.) et d'autres organismes étrangers en dehors de la RDC, comme la Food & Drug Administration des Etats Unis. J'autorise le personnel de ces organismes à consulter et analyser mes dossiers et les publier dans des revues scientifiques.	

For peer review only

For peer review only - http://bmjopen.bmj.com/site/about/guidelines.xhtml

FORMULAIRE D'ASSENTIMENT DU/DE LA PARTICIPANT(E)

Déclarations concernant les procédures facultatives

**Déclarations**	**Signe ou appose ton empreinte digitale dans chaque case si oui**	**Signe ou appose ton empreinte digitale dans chaque case si non**
(Pour les femmes participantes uniquement) J'accepte que si je suis enceinte au moment de la vaccination ou devient enceinte pendant le premier mois après avoir reçu chaque vaccin, et que je suis sélectionnée pour faire partie du groupe Grossesse, et qu'un membre de l'équipe de l'étude puisse me contacter plus frequement que les autres femmes enceintes incluses dans l'étude par téléphone pendant ma grossesse et, j'accepte également le suivi après mon accouchement pour recueillir des informations sur ma santé et celle de mon bébé. Si ces informations ne peuvent pas être recueillies par téléphone, j'accepte qu'un membre de l'équipe de l'étude vienne me voir à mon domicile.		
J'accepte que si je suis choisi par l'équipe d'étude pour faire appel au groupe de sécurité, l'équipe d'étude peut me contacter par téléphone après ma deuxième vaccination, et peut me voir à la maison si je ne peux pas être contacté par téléphone.		

J'accepte de participer à cette étude. (Signe ou appose ton empreinte digitale ci-dessous)

		____/____/_____ *jj mmm aaaa*
Nom de l'enfant en caractères d'imprimerie	Signature/Empreinte digitale de l'enfant	Date

		____/____/_____ *jj mmm aaaa*
Nom de l'investigateur en caractères d'imprimerie	Signature de l'investigateur	Date

For peer review only - http://bmjopen.bmj.com/site/about/guidelines.xhtml

Remplir si le/la participant(e) est analphabète :

Témoin de l'entretien d'assentiment

J'ai assisté à l'entretien d'assentiment pour l'étude [insérer nom] dans ce document. J'atteste avoir expliqué les informations de l'étude avec précision au/à la participant(e) et que ce dernier/cette dernière a compris, et qu'il/elle a donné librement son assentiment pour participer, en ma présence.

		____/____/_____ *jj mmm aaaa*
Nom du témoin impartial en caractères d'imprimerie	Signature du témoin impartial	Date

Apposez l'étiquette d'identification par code à barres ci-dessous :

For peer review only - http://bmjopen.bmj.com/site/about/guidelines.xhtml

Protocol for a phase 3 trial to evaluate the effectiveness and safety of a heterologous, two-dose vaccine for Ebola virus disease in the Democratic Republic of the Congo

Appendix 3A

Immunogenicity Informed Consent Form for adults in English, Version 1.1 (10 Aug 2020)

For peer review only

For peer review only - http://bmjopen.bmj.com/site/about/guidelines.xhtml

Evaluation of a heterologous, two-dose preventive Ebola vaccine for effectiveness and safety in the Democratic Republic of the Congo

"The TUJIOKOWE Study" Immunogenicity Subset Information Sheet and Informed Consent Form

LSHTM Protocol: DRC-EB-001

Principal Investigator (PI):
Professor Jean Jacques Muyembe
Director-General
Institut National de Recherche Biomédicale
Professor of Microbiology, Kinshasa University Medical School
Kinshasa Gombe, Democratic Republic of the Congo (DRC)
Phone: 0898 949 289; Email: jjmuyembet@gmail.com

Sponsor: London School of Hygiene & Tropical Medicine, United Kingdom

Site: Democratic Republic of the Congo

INTRODUCTION

The 'TUJIOKOWE study' is a research study to find out if a new two-dose vaccine called the 'Janssen Ebola vaccine' can protect people from getting Ebola and to check whether the vaccine is safe. The TUJIOKOWE study is being implemented by the Ministry of Health of DRC through the Institut National de Recherche Biomédicale (INRB), Epicentre, and the London School of Hygiene & Tropical Medicine.

When we started the TUJIOKOWE study, we planned to give everybody the second dose of the Ebola vaccine about two months after the first dose. Some people will now receive the second dose of Ebola vaccine later than originally planned. This is because it was necessary to temporarily close the study clinics due to the COVID-19 outbreak. We want to understand if people who receive their second dose of vaccine later than two months have the same immune responses as the people who received their second dose of vaccine at two months after the first dose. To do this, we will need to collect blood samples from approximately 50 adults, 25 adolescents, and 25 children participating in the TUJIOKOWE study who received the second dose of the Ebola vaccine after July 2020. We are asking you to join this small study (which we are calling the Immunogenicity Subset) and to provide two blood samples because you will be receiving the second dose of the vaccine after July 2020. If you agree to participate, one blood sample will be collected from you before the second dose of Ebola vaccine is given and the second blood sample will be collected 21 days later.

For peer review only - http://bmjopen.bmj.com/site/about/guidelines.xhtml

We would like to be sure that your body responds to the vaccine as well as people who received the second dose of the vaccine two months after the first dose. To check this, we will be looking at the antibodies that your body produces after you receive a vaccine. Antibodies are produced by your body after vaccination to protect you from infection. The information from testing your blood for antibodies will be very important when the vaccine is used to protect against Ebola in the future because it may not always be possible to give the second dose at exactly two months after the first dose of the vaccine.

If you are the parent or guardian of a child under 18 years of age and you agree to your child providing blood samples, you will need to sign separate documents for you and your child. If your child is aged 12 to 17 years old, he/she will also need to agree to provide blood samples himself/herself and sign a different document, called the immunogenicity assent form.

DO I HAVE TO GIVE BLOOD SAMPLES?

You do not have to provide any blood samples. If you agree to provide blood samples, we will ask you to sign this consent form. If you do not want to give any blood samples, this will not affect your participation in the TUJIOKOWE study.

WHAT WILL HAPPEN IF I AGREE TO GIVE BLOOD SAMPLES?

We will describe the blood collection process and answer any questions that you may have. If you would like a copy of the written information, we will give it to you. You will be asked to sign or put your fingerprint on this consent form. Putting your name or your fingerprint on the consent form means that you agree to provide blood samples, but you can change your mind at any time.

After you have given your first blood sample, you will follow the normal TUJIOKOWE study procedures to receive the second dose of vaccine. To have your second blood sample taken you will need to come to the study clinic for an extra visit 21 days after you have received the second dose of vaccine. If you participate in the Immunogenicity Subset, then we will give you some refreshments at the clinic.

WHAT DOES GIVING BLOOD INVOLVE?

A small amount of your blood will be collected from you using a small clean needle. We will take blood before you get the second injection of vaccine and at 21 days after the second injection. To have your second blood sample taken, you will need to come to the study clinic for an extra visit 21 days after you have received the second dose of vaccine. For adults, we will collect approximately 5 mL (about 1 teaspoon) of blood each time you provide a blood sample.

As explained earlier, the blood samples you give will be used to test whether your body has produced antibodies in your blood against Ebola after vaccination. You will not receive the results of these tests because they are only for scientific research.

For peer review only - http://bmjopen.bmj.com/site/about/guidelines.xhtml

After we do our tests on your blood samples for the Immunogenicity Subset of the TUJIOKOWE study, we will destroy any remaining blood that has been collected from you. We will not use your blood for any other purpose except the antibody tests that we have explained in this consent form. Your blood samples will not be sold.

Your blood samples will be labelled with a code and not your name. Other information, such as sex, age, or health history might also be shared with other investigators, but your name will not be shared.

WHAT ARE THE POSSIBLE RISKS OF GIVING A BLOOD SAMPLE?

Providing a blood sample is generally safe but occasionally people experience pain, bruising, bleeding, or (very rarely) infection from giving a blood sample. The medical team will provide care if you experience side-effects from the blood sampling. They are available if you have any problems or questions.

WHAT ARE THE BENEFITS OF GIVING A BLOOD SAMPLE?

There is no direct benefit to you for providing blood samples in the Immunogenicity Subset of the TUJIOKOWE study. Your agreement to provide blood samples will help in the development of vaccines to prevent Ebola and, in the future, may help people in different parts of the world.

WHAT HAPPENS TO THE BLOOD SAMPLES COLLECTED FROM ME?

The blood samples collected from you during the Immunogenicity Subset of the TUJIOKOWE study, will only be used to understand how the Ebola vaccine works when the second dose is given more than two months after the first dose.

The samples will be coded in a way that only limited study staff can link them to you. Your blood samples will be tested in a laboratory in the United States. If any blood remains in DRC, it will be stored in the INRB laboratory in Goma. We will destroy all the samples in the United States and in Goma at the end of the study.

For peer review only - http://bmjopen.bmj.com/site/about/guidelines.xhtml

WHO CAN I TALK TO ABOUT GIVING BLOOD SAMPLES?

If you want to talk to someone about giving a blood sample or if you think you have been harmed by providing a blood sample, you can contact the following people:

1) The Principal Investigator responsible for this study

Professor Jean Jacques Muyembe<br>*Director General*, Institut National de Recherche Biomédicale, Kinshasa, DRC<br>Phone: 0898 949 289<br>Email: jjmuyembet@gmail.com<br><br>Local study representative<br>Dr Hugo Kavunga<br>Phone: 0823 875 153<br>Email: hugokavunga@gmail.com

2) The DRC ethics committees that approved this study

Professeur Félicien Munday<br>National Ethics Committee<br>Kinshasa-Gombe, DRC<br>Phone: 0998 419 816<br>Email: feli1munday@yahoo.fr
Professeur Willy Bongopasi<br>Comité d'éthique de l'école de santé publique<br>Université de Kinshasa<br>Phone: 0999 952 341<br>Email: bongopasi@gmail.com

If you have any questions about providing blood samples for the Immunogenicity Subset of the TUJIOKOWE study or about your rights, you may ask anyone on the study team at any time.

For peer review only

For peer review only - http://bmjopen.bmj.com/site/about/guidelines.xhtml

PARTICIPANT IMMUNOGENICITY SUBSET CONSENT FORM

Title: Immunogenicity Subset of the TUJIOKOWE Study

Principal Investigator of this study: Prof. JJ Muyembe

**Statements**	**Please sign or fingerprint each box**
I have read the information in this form about giving a blood sample at two visits for the Immunogenicity Subset of the TUJIOKOWE Study (or I have had this information explained to me by the study staff in a language that I understand). The purpose of providing blood samples and the procedures to give blood samples have been fully explained to me. I was able to ask questions and have all of these questions answered to my satisfaction.	
I understand that my participation is voluntary, that I can withdraw consent to provide blood samples at any time without giving any reason, and that this will not affect my participation in the TUJIOKOWE Study.	

I am aged 18 years or older and I agree to provide Immunogenicity Subset blood samples during my participation in the TUJIOKOWE study. (Please sign or put your fingerprint below)

		____/____/_____ *dd mon yyyy*
Printed name of adult participant	Signature/fingerprint of adult participant	Date

		____/____/_____ *dd mon yyyy*
Printed name of investigator	Signature of investigator	Date

For peer review only - http://bmjopen.bmj.com/site/about/guidelines.xhtml

Complete this section if the participant is illiterate:

Witness to Consent Interview

I witnessed the consent interview for the Immunogenicity Subset of the TUJIOKOWE Study in this document. I attest that I have explained the study information accurately to the participant or parent/guardian and was understood to the best of my knowledge by the participant and that he/she has freely given their consent to participate in my presence.

		____/____/_____ *dd mon yyyy*
Printed name of impartial witness	Signature of impartial witness	Date

Attach ID barcode label below:

For peer review only - http://bmjopen.bmj.com/site/about/guidelines.xhtml

Protocol for a phase 3 trial to evaluate the effectiveness and safety of a heterologous, two-dose vaccine for Ebola virus disease in the Democratic Republic of the Congo

Appendix 3B

Immunogenicity Informed Consent Form for adults in French, Version 1.1 (10 Aug 2020)

For peer review only

Évaluation de l'efficacité et de la sécurité d'emploi d'un vaccin préventif hétérologue à deux doses contre Ebola en République démocratique du Congo

Note d'information et formulaire de consentement éclairé pour le sous-groupe Immunogénicité de l'« étude TUJIOKOWE »

Protocole LSHTM : DRC-EB-001

Investigateur principal (IP) :
Professeur Jean Jacques Muyembe
Directeur général
Institut national de recherche biomédicale
Professeur de microbiologie, Faculté de médecine de l'Université de Kinshasa
Kinshasa Gombe, République démocratique du Congo (RDC)
Téléphone : 0898 949 289 ; adresse électronique : jjmuyembet@gmail.com

Promoteur : London School of Hygiene & Tropical Medicine, Royaume-Uni

Centre : République démocratique du Congo

INTRODUCTION

L'« étude TUJIOKOWE » est une étude de recherche visant à déterminer si un nouveau vaccin à deux doses appelé « vaccin contre le virus Ebola de Janssen » peut protéger contre le virus Ebola et à vérifier si le vaccin est sans danger. L'étude TUJIOKOWE est mise en œuvre par le ministère de la Santé de la RDC par l'intermédiaire de l'Institut national de recherche biomédicale (INRB), Epicentre et la London School of Hygiene & Tropical Medicine.

Lorsque nous avons commencé l'étude TUJIOKOWE, nous avions prévu d'administrer la seconde dose du vaccin contre le virus Ebola à tous les participants 2 mois environ après la première dose. Certaines personnes vont maintenant recevoir la seconde dose du vaccin contre le virus Ebola plus tard que prévu. Cela s'explique par la nécessité de fermer temporairement les centres de l'étude en raison de l'épidémie de COVID-19. Nous voulons comprendre si les personnes qui reçoivent leur seconde dose de vaccin plus de 2 mois après présentent la même réponse immunitaire que les personnes qui l'ont reçue 2 mois après la première dose. À cet effet, nous allons devoir prélever des échantillons de sang auprès de 50 adultes, 25 adolescents et 25 enfants environ participant à l'étude TUJIOKOWE, qui ont reçu la seconde dose du vaccin contre le virus Ebola après juillet 2020. Nous vous demandons de participer à cette petite étude (que nous appelons « sous-groupe Immunogénicité ») et de fournir deux échantillons de sang, car vous allez recevoir la seconde dose du vaccin après juillet 2020. Si vous acceptez de participer à l'étude, un échantillon de sang vous sera prélevé avant l'administration de la seconde dose du vaccin contre le virus Ebola, et le second échantillon de sang sera prélevé 21 jours plus tard.

For peer review only - http://bmjopen.bmj.com/site/about/guidelines.xhtml

Nous voulons nous assurer que votre organisme réagit au vaccin aussi bien que celui des personnes qui ont reçu la seconde dose du vaccin 2 mois après la première dose. À cet effet, nous allons rechercher les anticorps que produit votre organisme après l'administration du vaccin. Les anticorps sont produits par l'organisme après la vaccination pour vous protéger contre l'infection. Les informations obtenues en analysant votre sang pour rechercher la présence d'anticorps seront très importantes lorsque le vaccin sera utilisé pour protéger contre le virus Ebola à l'avenir, car il ne sera peut-être pas toujours possible d'administrer la seconde dose du vaccin 2 mois exactement après la première dose.

Si vous êtes le parent ou le tuteur d'un enfant de moins de 18 ans et si vous acceptez qu'il fournisse les échantillons de sang, vous devrez signer des documents distincts pour vous et votre enfant. Si votre enfant est âgé de 12 à 17 ans, il devra également accepter de lui-même de fournir les échantillons de sang et signer un autre document, appelé « formulaire d'assentiment » pour le sous-groupe Immunogénicité.

SUIS-JE OBLIGÉ DE DONNER DES ÉCHANTILLONS DE SANG ?

Vous n'avez pas l'obligation de fournir des échantillons de sang. Si vous acceptez de fournir des échantillons de sang, nous vous inviterons à signer ce formulaire de consentement. Si vous ne souhaitez pas donner d'échantillons de sang, cela ne remettra pas en cause votre participation à l'étude TUJIOKOWE.

QUE SE PASSERA-T-IL SI J'ACCEPTE DE DONNER DES ÉCHANTILLONS DE SANG ?

Nous allons décrire les modalités de prise de sang et répondre à toutes les questions que vous pourriez avoir. Si vous souhaitez une copie de cette note d'information, nous vous la remettrons. Nous vous inviterons à signer ce formulaire de consentement ou à y apposer votre empreinte digitale. En inscrivant votre nom ou en apposant votre empreinte digitale sur le formulaire de consentement, vous acceptez de fournir des échantillons de sang, mais vous pourrez changer d'avis à tout moment.

Une fois que vous aurez donné votre premier échantillon de sang, vous suivrez les procédures habituelles de l'étude TUJIOKOWE pour recevoir la seconde dose de vaccin. Pour le prélèvement de votre second échantillon de sang, vous devrez venir effectuer une visite supplémentaire au centre de l'étude 21 jours après l'administration de la seconde dose de vaccin. Si vous participez au sous-groupe Immunogénicité, chaque fois que vous donnerez votre sang, nous vous donnerons des rafraîchissements.

For peer review only - http://bmjopen.bmj.com/site/about/guidelines.xhtml

EN QUOI CONSISTE LE PRELEVEMENT DE SANG ?

Une petite quantité de votre sang vous sera prélevée à l'aide d'une petite aiguille propre. Nous vous prélèverons un échantillon de sang avant que vous receviez la seconde injection du vaccin et 21 jours après la seconde injection. Pour le prélèvement de votre second échantillon de sang, vous devrez effectuer une visite supplémentaire au centre de l'étude 21 jours après l'administration de la seconde dose de vaccin. Pour les adultes, nous prélèverons 5 ml environ (soit 1 cuillère à café environ) de sang chaque fois que vous fournirez un échantillon de sang.

Comme indiqué précédemment, les échantillons de sang que vous donnerez serviront à vérifier si votre organisme a produit des anticorps dans votre sang contre le virus Ebola après la vaccination. Vous ne recevrez pas les résultats de ces analyses, car ils sont destinés uniquement à la recherche scientifique.

Après avoir réalisé nos analyses sur vos échantillons de sang pour le sous-groupe Immunogénicité de l'étude TUJIOKOWE, nous détruirons tous les échantillons de sang restants qui vous ont été prélevés. Nous n'utiliserons pas vos échantillons de sang à d'autres fins que les analyses de détection d'anticorps mentionnés dans ce formulaire de consentement. Vos échantillons de sang ne seront pas vendus.

Vos échantillons de sang seront étiquetés avec un code et non avec votre nom. D'autres informations, comme le sexe, l'âge ou les antécédents médicaux, pourront également être partagées avec d'autres investigateurs, mais votre nom ne sera pas communiqué.

QUELS SONT LES RISQUES EVENTUELS LIES AU DON D'UN ECHANTILLON DE SANG ?

Le prélèvement d'un échantillon de sang est généralement sans danger, mais il arrive que des personnes ressentent des douleurs ou présentent un bleu, un saignement ou (dans de très rares cas) une infection après un prélèvement de sang. Si vous présentez des effets secondaires après la prise de sang, l'équipe médicale vous apportera les soins nécessaires. L'équipe est disponible à tout moment si vous avez des problèmes ou des questions.

QUELS SONT LES BENEFICES LIES AU DON D'UN ECHANTILLON DE SANG ?

Il n'y a aucun bénéfice direct pour vous à fournir des échantillons de sang dans le cadre du sous-groupe Immunogénicité de l'étude TUJIOKOWE. Le consentement à fournir des échantillons de sang contribuera au développement de vaccins pour prévenir le virus Ebola et, à l'avenir, pourrait aider des personnes dans différentes régions du monde.

QU'ADVIENDRA-IL DES ECHANTILLONS DE SANG QUI ME SERONT PRELEVES ?

Les échantillons de sang qui vous ont été prélevés dans le cadre du sous-groupe Immunogénicité de l'étude TUJIOKOWE ne seront utilisés que pour comprendre l'efficacité du vaccin contre le virus Ebola lorsque la seconde dose est administrée plus de 2 mois après la première dose.

For peer review only - http://bmjopen.bmj.com/site/about/guidelines.xhtml

Les échantillons seront codés de manière à limiter le nombre de membres du personnel de l'étude qui peut les relier à vous. Vos échantillons de sang seront analysés dans un laboratoire aux États-Unis. S'il reste des échantillons de sang en RDC, ils seront conservés dans le laboratoire de l'INRB à Goma. Nous détruirons tous les échantillons conservés aux États-Unis et à Goma à la fin de l'étude.

À QUI PUIS-JE M'ADRESSER CONCERNANT LE PRÉLÈVEMENT D'ÉCHANTILLONS DE SANG ?

Si vous souhaitez parler à quelqu'un du prélèvement d'un échantillon de sang ou si vous pensez avoir subi un préjudice en fournissant un échantillon de sang, vous pouvez contacter les personnes suivantes :

1) Investigateur principal responsable de cette étude

Professeur Jean Jacques Muyembe<br>*Directeur général*, Institut national de recherche biomédicale, Kinshasa, RDC<br>Téléphone : 0898 949 289<br>Adresse électronique : jjmuyembet@gmail.com<br><br>Représentant locale de l'étude<br>Dr Hugo Kavunga<br>Téléphone : 0823 875 153<br>Adresse électronique : hugokavunga@gmail.com

2) Comités d'éthique de la RDC ayant approuvé cette étude

Professeur Félicien Munday<br>Comité national d'éthique et de la santé<br>Kinshasa-Gombe, RDC<br>Téléphone : 0998 419 816<br>Adresse électronique : feli1munday@yahoo.fr
Professeur Willy Bongopasi<br>Comité d'éthique de l'école de santé publique<br>Université de Kinshasa, RDC<br>Téléphone : 0999 952 341<br>Adresse électronique : bongopasi@gmail.com

Si vous avez des questions sur le prélèvement d'échantillons de sang pour le sous-groupe Immunogénicité de l'étude TUJIOKOWE ou sur vos droits, vous pouvez les poser à tout moment à n'importe quel membre de l'équipe de l'étude.

For peer review only - http://bmjopen.bmj.com/site/about/guidelines.xhtml

FORMULAIRE DE CONSENTEMENT DES PARTICIPANTS POUR LE SOUS-GROUPE IMMUNOGÉNICITÉ

Titre : Sous-groupe Immunogénicité de l'étude TUJIOKOWE

Investigateur principal de cette étude : Prof. JJ Muyembe

**Déclarations**	**Signez ou apposez votre empreinte digitale dans chaque case**
J'ai lu les informations contenues dans ce formulaire sur le prélèvement d'un échantillon de sang lors de deux visites pour le sous-groupe Immunogénicité de l'étude TUJIOKOWE (ou le personnel de l'étude m'a expliqué ces informations avec des mots que je comprends). L'objectif du prélèvement d'échantillons de sang et les procédures correspondantes m'ont été expliqués en détail. J'ai pu poser des questions et j'ai reçu des réponses satisfaisantes à toutes mes questions.	
Je comprends que ma participation est volontaire, que je peux retirer son consentement à fournir des échantillons de sang à tout moment sans avoir à me justifier et que cela ne remettra pas en cause ma participation à l'étude TUJIOKOWE.	

J'ai 18 ans ou plus et j'accepte de fournir des échantillons de sang pour le sous-groupe Immunogénicité pendant ma participation à l'étude TUJIOKOWE. (Signez ou apposez votre empreinte digitale ci-dessous)

		____/____/_____ *jj mmm aaaa*
Nom du participant adulte (en caractères d'imprimerie)	Signature/Empreinte digitale du participant adulte	Date

		____/____/_____ *jj mmm aaaa*
Nom de l'investigateur (en caractères d'imprimerie)	Signature de l'investigateur	Date

For peer review only - http://bmjopen.bmj.com/site/about/guidelines.xhtml

Si le participant est analphabète, remplissez cette section :

Témoin de l'entretien de consentement

J'ai assisté à l'entretien de consentement pour le sous-groupe Immunogénicité de l'étude TUJIOKOWE décrite dans ce document. J'atteste avoir expliqué avec exactitude les informations sur l'étude au participant ou au parent/tuteur et que, à ma connaissance, le participant les a comprises et a donné librement son consentement à participer, en ma présence.

		____/____/_____ *jj mmm aaaa*
Nom du témoin impartial (en caractères d'imprimerie)	Signature du témoin impartial	Date

Apposez l'étiquette d'identification par code à barres ci-dessous :

For peer review only

For peer review only - http://bmjopen.bmj.com/site/about/guidelines.xhtml

Protocol for a phase 3 trial to evaluate the effectiveness and safety of a heterologous, two-dose vaccine for Ebola virus disease in the Democratic Republic of the Congo

Appendix 4A

Immunogenicity Informed Assent Form for children in English, Version 1.1 (10 Aug 2020)

For peer review only

Evaluation of a heterologous, two-dose preventive Ebola vaccine for effectiveness and safety in the Democratic Republic of the Congo

"The TUJIOKOWE Study" Immunogenicity Subset
Information Sheet and Informed Assent Form for Children Aged 12 Years or Older

LSHTM Protocol: DRC-EB-001

Principal Investigator (PI):
Professor Jean Jacques Muyembe
Director-General
Institut National de Recherche Biomédicale
Professor of Microbiology, Kinshasa University Medical School
Kinshasa Gombe, Democratic Republic of the Congo (DRC)
Phone: 0898 949 289; Email: jjmuyembet@gmail.com

Sponsor: London School of Hygiene & Tropical Medicine, United Kingdom

Site: Democratic Republic of the Congo

INTRODUCTION

The 'TUJIOKOWE study' is a research study to find out if a new two-dose vaccine called the 'Janssen Ebola vaccine' can protect people from getting Ebola and to check whether the vaccine is safe. The TUJIOKOWE study is being implemented by the Ministry of Health of DRC through the Institut National de Recherche Biomédicale (INRB), Epicentre, and the London School of Hygiene & Tropical Medicine.

When we started the TUJIOKOWE study, we planned to give everybody the second injection of the Ebola vaccine about two months after the first injection. Some people will now receive the second injection of Ebola vaccine later than originally planned. This is because we needed to temporarily close the study clinics due to the COVID-19 outbreak. We want to understand if the Ebola vaccine works well if people receive their second injection later than two months. To do this, we will need to collect blood samples from about 50 adults, 25 adolescents, and 25 children participating in the TUJIOKOWE study who received the second injection of the Ebola vaccine after July 2020. We are asking you to join this small study (which we are calling the Immunogenicity Subset) and to provide two blood samples because you will be receiving the second injection of the vaccine after July 2020. If you agree to participate, one blood sample will be collected from you before the second injection of Ebola vaccine is given and the second blood sample will be collected 21 days later.

We would like to be sure that your body responds to the vaccine as well as people who received the second injection of the vaccine two months after the first injection. To check this, we will

For peer review only - http://bmjopen.bmj.com/site/about/guidelines.xhtml

be looking at the antibodies that your body produces after you receive a vaccine. Antibodies are produced by your body after vaccination to protect you from infection. The information from testing your blood for antibodies will be very important when the vaccine is used to protect against Ebola in the future because it may not always be possible to give the second injection at exactly two months after the first injection of the vaccine.

DO I HAVE TO GIVE BLOOD SAMPLES?

You do not have to provide any blood samples if you do not want to, even if your parents or guardians want you to. If you agree to provide blood samples, we will ask you to sign this assent form. If you do not want to give any blood samples, this will not affect your participation in the TUJIOKOWE study.

WHAT WILL HAPPEN IF I AGREE TO GIVE BLOOD SAMPLES?

We will describe the blood collection process and answer any questions that you may have. If you would like a copy of the written information, we will give it to you. You will be asked to sign or put your fingerprint on this assent form. Putting your name or your fingerprint on the assent form means that you agree to provide blood samples, but you can change your mind at any time.

After the first blood sample you give, you will follow the normal TUJIOKOWE study procedures to receive the second injection of vaccine. To have your second blood sample taken you will need to come to the study clinic for an extra visit 21 days after you have received the second injection of vaccine. If you participate in the immunogenicity subset, we will give you some refreshments at the clinic.

WHAT DOES GIVING BLOOD INVOLVE?

A small amount of your blood will be collected from you using a small clean needle. We will take blood before you get the second injection of vaccine and at 21 days after the second injection. To have your second blood sample taken you will need to come to the study clinic for an extra visit 21 days after you have received the second injection of vaccine. For children aged 6 years and older, we will collect approximately 5 mL (about 1 teaspoon) of blood each time you provide a blood sample.

As explained to you earlier, the blood samples you give will be used to test whether your body has produced antibodies in your blood against Ebola after vaccination. You will not receive the results of these tests because they are only for scientific research.

After we do our tests on your blood samples for the Immunogenicity Subset of the TUJIOKOWE study, we will destroy any remaining blood that you have given us. We will not use your blood for any other purpose except the antibody tests that we have explained in this assent form. Your blood samples will not be sold.

Your blood samples will be labelled with a code and not your name. Other information, such as sex, age, or health history might also be shared with other investigators, but your name will not be shared.

For peer review only - http://bmjopen.bmj.com/site/about/guidelines.xhtml

WHAT ARE THE POSSIBLE RISKS OF GIVING A BLOOD SAMPLE?

Giving a blood sample is generally safe but occasionally people experience pain, bruising, bleeding, or (very rarely) infection from giving a blood sample. The medical team will provide care if you experience side-effects from the blood sampling. They are available if you have any problems or questions.

WHAT ARE THE BENEFITS OF GIVING A BLOOD SAMPLE?

There is no direct benefit to you for providing blood samples in the Immunogenicity Subset of the TUJIOKOWE study. Your agreement to provide blood samples will help in the development of vaccines to prevent Ebola and, in the future, may help people in different parts of the world.

WHAT HAPPENS TO THE BLOOD SAMPLES COLLECTED FROM ME?

The blood samples collected from you during the Immunogenicity Subset of the TUJIOKOWE study, will only be used to understand how the Ebola vaccine works when the second injection is given more than two months after the first injection.

The samples will be coded in a way that only limited study staff can link them to you. Your blood samples will be tested in a laboratory in the United States. If any blood remains in DRC, it will be stored in the INRB laboratory in Goma. We will destroy all the samples in the United States and in Goma at the end of the study.

For peer review only

For peer review only - http://bmjopen.bmj.com/site/about/guidelines.xhtml

WHO CAN I TALK TO ABOUT GIVING BLOOD SAMPLES?

If you want to talk to someone about giving a blood sample or if you think you have been harmed by providing a blood sample, you can contact the following people:

1) The Principal Investigator responsible for this study

Professor Jean Jacques Muyembe<br>*Director General*, Institut National de Recherche Biomédicale, Kinshasa, DRC<br>Phone: 0898 949 289<br>Email: jjmuyembet@gmail.com<br><br>Local study representative<br>Dr Hugo Kavunga<br>Phone: 0823 875 153<br>Email: hugokavunga@gmail.com

2) The DRC ethics committees that approved this study

Professeur Félicien Munday<br>National Ethics Committee<br>Kinshasa-Gombe, DRC<br>Phone: 0998 419 816<br>Email: feli1munday@yahoo.fr
Professeur Willy Bongopasi<br>Comité d'éthique de l'école de santé publique<br>Université de Kinshasa<br>Phone: 0999 952 341<br>Email: bongopasi@gmail.com

If you have any questions about providing blood samples for the Immunogenicity Subset of the TUJIOKOWE study or about your rights, you may ask anyone on the study team at any time.

For peer review only - http://bmjopen.bmj.com/site/about/guidelines.xhtml

PARTICIPANT IMMUNOGENICITY SUBSET ASSENT FORM

Title: Immunogenicity Subset of the TUJIOKOWE Study

Principal Investigator of this study: Prof. JJ Muyembe

**Statements**	**Please sign or fingerprint each box**
I have read the information in this form about giving a blood sample at two visits for the Immunogenicity Subset of the TUJIOKOWE Study (or I have had this information explained to me by the study staff in a language that I understand). The purpose of providing blood samples and the procedures to give blood samples have been fully explained to me. I was able to ask questions and have all of these questions answered to my satisfaction.	
I understand that my participation is voluntary, that I can withdraw consent to provide blood samples at any time without giving any reason, and that this will not affect my participation in the TUJIOKOWE Study.	

I am aged 12-17 years and I agree to provide Immunogenicity Subset blood samples during my participation in the TUJIOKOWE study. (Please sign or put your fingerprint below)

		____/____/_____ *dd mon yyyy*
Printed name of child	Signature/fingerprint of child	Date

		____/____/_____ *dd mon yyyy*
Printed name of investigator	Signature of investigator	Date

For peer review only - http://bmjopen.bmj.com/site/about/guidelines.xhtml

Complete this section if the participant is illiterate:

Witness to Assent Interview

I witnessed the assent interview for the Immunogenicity Subset of the TUJIOKOWE Study in this document. I attest that I have explained the study information accurately to the participant and was understood to the best of my knowledge by the participant and that he/she has freely given their assent to participate in my presence.

		____/____/_____ *dd mon yyyy*
Printed name of impartial witness	Signature of impartial witness	Date

Attach ID barcode label below:

For peer review only

For peer review only - http://bmjopen.bmj.com/site/about/guidelines.xhtml

Protocol for a phase 3 trial to evaluate the effectiveness and safety of a heterologous, two-dose vaccine for Ebola virus disease in the Democratic Republic of the Congo

Appendix 4B

Immunogenicity Informed Assent Form for children in French, Version 1.1 (10 Aug 2020)

For peer review only

For peer review only - http://bmjopen.bmj.com/site/about/guidelines.xhtml

Évaluation de l'efficacité et de la sécurité d'emploi d'un vaccin préventif hétérologue à deux doses contre Ebola en République démocratique du Congo

Note d'information et formulaire d'assentiment éclairé pour le sous-groupe Immunogénicité de l'« étude TUJIOKOWE » destinés aux enfants âgés de 12 ans ou plus

Protocole LSHTM : DRC-EB-001

Investigateur principal (IP) :
Professeur Jean Jacques Muyembe
Directeur général
Institut national de recherche biomédicale
Professeur de microbiologie, Faculté de médecine de l'Université de Kinshasa
Kinshasa Gombe, République démocratique du Congo (RDC)
Téléphone : 0898 949 289 ; Adresse électronique : jjmuyembet@gmail.com

Promoteur : London School of Hygiene & Tropical Medicine, Royaume-Uni

Centre : République démocratique du Congo

INTRODUCTION

L'« étude TUJIOKOWE » est une étude de recherche visant à déterminer si un nouveau vaccin à deux doses appelé « vaccin contre le virus Ebola de Janssen » peut protéger contre le virus Ebola et à vérifier si le vaccin est sans danger. L'étude TUJIOKOWE est mise en œuvre par le ministère de la Santé de la RDC par l'intermédiaire de l'Institut national de recherche biomédicale (INRB), Epicentre et la London School of Hygiene & Tropical Medicine.

Lorsque nous avons commencé l'étude TUJIOKOWE, nous avions prévu d'administrer la seconde injection du vaccin contre le virus Ebola à tous les participants 2 mois environ après la première injection. Certaines personnes vont maintenant recevoir la seconde injection du vaccin contre le virus Ebola plus tard que prévu. Cela s'explique par la nécessité de fermer temporairement les centres de l'étude en raison de l'épidémie de COVID-19. Nous voulons savoir si le vaccin contre le virus Ebola est aussi efficace lorsque les personnes reçoivent leur seconde injection plus de 2 mois après. À cet effet, nous allons devoir prélever des échantillons de sang auprès de 50 adultes, 25 adolescents et 25 enfants environ participant à l'étude TUJIOKOWE, qui ont reçu la seconde injection du vaccin contre le virus Ebola après juillet 2020. Nous vous demandons de participer à cette petite étude (que nous appelons « sous-groupe Immunogénicité ») et de fournir deux échantillons de sang, car vous allez recevoir la seconde injection du vaccin après juillet 2020. Si vous acceptez de participer à l'étude, un

For peer review only - http://bmjopen.bmj.com/site/about/guidelines.xhtml

échantillon de sang vous sera prélevé avant l'administration de la seconde injection du vaccin contre le virus Ebola, et le second échantillon de sang sera prélevé 21 jours plus tard.

Nous voulons nous assurer que votre organisme réagit au vaccin aussi bien que celui des personnes qui ont reçu la seconde injection du vaccin 2 mois après la première injection. À cet effet, nous allons rechercher les anticorps que produit votre organisme après l'administration du vaccin. Les anticorps sont produits par l'organisme après la vaccination pour vous protéger contre l'infection. Les informations obtenues en analysant votre sang pour rechercher la présence d'anticorps seront très importantes lorsque le vaccin sera utilisé pour protéger contre le virus Ebola à l'avenir, car il ne sera peut-être pas toujours possible d'administrer la seconde injection du vaccin 2 mois exactement après la première injection du vaccin.

SUIS-JE OBLIGÉ DE DONNER DES ÉCHANTILLONS DE SANG ?

Vous n'êtes pas obligé de fournir des échantillons de sang si vous ne le souhaitez pas, même si vos parents ou tuteurs le souhaitent. Si vous acceptez de fournir des échantillons de sang, nous vous inviterons à signer ce formulaire d'assentiment. Si vous ne souhaitez pas donner d'échantillons de sang, cela ne remettra pas en cause votre participation à l'étude TUJIOKOWE.

QUE SE PASSERA-T-IL SI J'ACCEPTE DE DONNER DES ÉCHANTILLONS DE SANG ?

Nous allons décrire les modalités de prise de sang et répondre à toutes les questions que vous pourriez avoir. Si vous souhaitez une copie de cette note d'information, nous vous la remettrons. Nous vous inviterons à signer ce formulaire d'assentiment ou à y apposer votre empreinte digitale. En inscrivant votre nom sur le formulaire d'assentiment ou en y apposant votre empreinte digitale, vous acceptez de fournir des échantillons de sang, mais vous pourrez changer d'avis à tout moment.

Une fois que vous aurez donné votre premier échantillon de sang, vous suivrez les procédures habituelles de l'étude TUJIOKOWE pour recevoir la seconde injection du vaccin. Pour le prélèvement de votre second échantillon de sang, vous devrez venir effectuer une visite supplémentaire au centre de l'étude 21 jours après l'administration de la seconde injection du vaccin. Si vous participez au sous-groupe Immunogénicité, chaque fois que vous donnerez votre sang, nous vous donnerons des rafraîchissements.

EN QUOI CONSISTE LE PRELEVEMENT DE SANG ?

Une petite quantité de votre sang vous sera prélevée à l'aide d'une petite aiguille propre. Nous vous prélèverons un échantillon de sang avant que vous receviez la seconde injection du vaccin et 21 jours après la seconde injection. Pour le prélèvement de votre second échantillon de sang, vous devrez venir effectuer une visite supplémentaire au centre de l'étude 21 jours après l'administration de la seconde injection du vaccin. Pour les enfants âgés de 6 ans et plus, nous prélèverons 5 ml environ (soit 1 cuillère à café environ) de sang chaque fois que vous fournirez un échantillon de sang.

For peer review only - http://bmjopen.bmj.com/site/about/guidelines.xhtml

Comme indiqué précédemment, les échantillons de sang que vous donnerez serviront à vérifier si votre organisme a produit des anticorps dans votre sang contre le virus Ebola après la vaccination. Vous ne recevrez pas les résultats de ces analyses, car ils sont destinés uniquement à la recherche scientifique.

Après avoir réalisé nos analyses sur vos échantillons de sang pour le sous-groupe Immunogénicité de l'étude TUJIOKOWE, nous détruirons tous les échantillons de sang restants qui vous ont été prélevés. Nous n'utiliserons pas vos échantillons de sang à d'autres fins que les analyses de détection d'anticorps mentionnés dans ce formulaire d'assentiment. Vos échantillons de sang ne seront pas vendus.

Vos échantillons de sang seront étiquetés avec un code et non avec votre nom. D'autres informations, comme le sexe, l'âge ou les antécédents médicaux, pourront également être partagées avec d'autres investigateurs, mais votre nom ne sera pas communiqué.

QUELS SONT LES RISQUES EVENTUELS LIES AU DON D'UN ECHANTILLON DE SANG ?

Le prélèvement d'un échantillon de sang est généralement sans danger, mais il arrive que des personnes ressentent des douleurs ou présentent un bleu, un saignement ou (dans de très rares cas) une infection après un don de sang. Si vous présentez des effets secondaires après la prise de sang, l'équipe médicale vous apportera les soins nécessaires. L'équipe est disponible à tout moment si vous avez des problèmes ou des questions.

QUELS SONT LES BENEFICES LIES AU DON D'UN ECHANTILLON DE SANG ?

Il n'y a aucun bénéfice direct pour vous à fournir des échantillons de sang dans le cadre du sous-groupe Immunogénicité de l'étude TUJIOKOWE. Le consentement à fournir des échantillons de sang contribuera au développement de vaccins pour prévenir le virus Ebola et, à l'avenir, pourrait aider des personnes dans différentes régions du monde.

QU'ADVIENDRA-IL DES ECHANTILLONS DE SANG QUI ME SERONT PRELEVES ?

Les échantillons de sang qui vous ont été prélevés dans le cadre du sous-groupe Immunogénicité de l'étude TUJIOKOWE ne seront utilisés que pour comprendre l'efficacité du vaccin contre le virus Ebola lorsque la seconde injection est administrée plus de 2 mois après la première injection.

Les échantillons seront codés de manière à limiter le nombre de membres du personnel de l'étude qui peut les relier à vous. Vos échantillons de sang seront analysés dans un laboratoire aux États-Unis. S'il reste des échantillons de sang en RDC, ils seront conservés dans le laboratoire de l'INRB à Goma. Nous détruirons tous les échantillons conservés aux États-Unis et à Goma à la fin de l'étude.

For peer review only - http://bmjopen.bmj.com/site/about/guidelines.xhtml

À QUI PUIS-JE M'ADRESSER CONCERNANT LE PRÉLÈVEMENT D'ÉCHANTILLONS DE SANG ?

Si vous souhaitez parler à quelqu'un du prélèvement d'un échantillon de sang ou si vous pensez avoir subi un préjudice en fournissant un échantillon de sang, vous pouvez contacter les personnes suivantes :

1) Investigateur principal responsable de cette étude

Professeur Jean-Jacques Muyembe<br>*Directeur général*, Institut national de recherche biomédicale, Kinshasa, RDC<br>Téléphone : 0898 949 289<br>Adresse électronique : jjmuyembet@gmail.com<br><br>Représentant locale de l'étude<br>Dr Hugo Kavunga<br>Téléphone : 0823 875 153<br>Adresse électronique : hugokavunga@gmail.com

2) Comités d'éthique de la RDC qui ont approuvé cette étude

Professeur Félicien Munday<br>Comité national d'éthique et de la santé<br>Kinshasa-Gombe, RDC<br>Téléphone : 0998 419 816<br>Adresse électronique : feli1munday@yahoo.fr
Professeur Willy Bongopasi<br>Comité d'éthique de l'école de santé publique<br>Université de Kinshasa, RDC<br>Téléphone : 0999 952 341<br>Adresse électronique : bongopasi@gmail.com

Si vous avez des questions sur le prélèvement d'échantillons de sang pour le sous-groupe Immunogénicité de l'étude TUJIOKOWE ou sur vos droits, vous pouvez les poser à tout moment à n'importe quel membre de l'équipe de l'étude.

For peer review only - http://bmjopen.bmj.com/site/about/guidelines.xhtml

FORMULAIRE DE ASSENTIMENT DES PARTICIPANTS POUR LE SOUS-GROUPE IMMUNOGÉNICITÉ

Titre : Sous-groupe Immunogénicité de l'étude TUJIOKOWE

Investigateur principal de cette étude : Prof. JJ Muyembe

**Déclarations**	**Signez ou apposez votre empreinte digitale dans chaque case**
J'ai lu les informations contenues dans ce formulaire sur le prélèvement d'un échantillon de sang lors de deux visites pour le sous-groupe Immunogénicité de l'étude TUJIOKOWE (ou le personnel de l'étude m'a expliqué ces informations avec des mots que je comprends). L'objectif du prélèvement d'échantillons de sang et les procédures correspondantes m'ont été expliqués en détail. J'ai pu poser des questions et j'ai reçu des réponses satisfaisantes à toutes mes questions.	
Je comprends que ma participation est volontaire, que je peux retirer son consentement à fournir des échantillons de sang à tout moment sans avoir à me justifier et que cela ne remettra pas en cause ma participation à l'étude TUJIOKOWE.	

Je suis âgé de 12 à 17 ans et j'accepte de fournir des échantillons de sang pour le sous-groupe Immunogénicité pendant ma participation à l'étude TUJIOKOWE. (Signez ou apposez votre empreinte digitale ci-dessous)

		____/____/_____<br>*jj mmm aaaa*
Nom de l'enfant (en caractères d'imprimerie)	Signature/Empreinte digitale de l'enfant	Date

		____/____/_____<br>*jj mmm aaaa*
Nom de l'investigateur (en caractères d'imprimerie)	Signature de l'investigateur	Date

For peer review only - http://bmjopen.bmj.com/site/about/guidelines.xhtml

---

Si le participant est analphabète, remplissez cette section :

Témoin de l'entretien d'Assentiment

J'ai assisté à l'entretien d'assentiment pour le sous-groupe Immunogénicité de l'étude TUJIOKOWE décrite dans ce document. J'atteste avoir expliqué avec exactitude les informations sur l'étude au participant et que, à ma connaissance, le participant les a comprises et a donné librement son assentiment à participer, en ma présence.

		____/____/_____ *jj mmm aaaa*
Nom du témoin impartial (en caractères d'imprimerie)	Signature du témoin impartial	Date

---

Apposez l'étiquette d'identification par code à barres ci-dessous :

For peer review only - http://bmjopen.bmj.com/site/about/guidelines.xhtml

Protocol for a phase 3 trial to evaluate the effectiveness and safety of a heterologous, two-dose vaccine for Ebola virus disease in the Democratic Republic of the Congo

Appendix 5A

Immunogenicity Informed Consent Form for Parents in English, Version 1.1 (10 Aug 2020)

For peer review only

For peer review only - http://bmjopen.bmj.com/site/about/guidelines.xhtml

Evaluation of a heterologous, two-dose preventive Ebola vaccine for effectiveness and safety in the Democratic Republic of the Congo

"The TUJIOKOWE Study" Immunogenicity Subset Information Sheet and Informed Consent Form for Parents/Guardians of Paediatric Participants

LSHTM Protocol: DRC-EB-001

Principal Investigator (PI):
Professor Jean Jacques Muyembe
Director-General
Institut National de Recherche Biomédicale
Professor of Microbiology, Kinshasa University Medical School
Kinshasa Gombe, Democratic Republic of the Congo (DRC)
Phone: 0898 949 289; Email: jjmuyembet@gmail.com

Sponsor: London School of Hygiene & Tropical Medicine, United Kingdom

Site: Democratic Republic of the Congo

INTRODUCTION

The 'TUJIOKOWE study' is a research study to find out if a new two-dose vaccine called the 'Janssen Ebola vaccine' can protect people from getting Ebola and to check whether the vaccine is safe. The TUJIOKOWE study is being implemented by the Ministry of Health of DRC through the Institut National de Recherche Biomédicale (INRB) and with Médecins Sans Frontières, Epicentre, and the London School of Hygiene & Tropical Medicine.

When we started the TUJIOKOWE study, we planned to give everybody the second dose of the Ebola vaccine about two months after the first dose. Some people will now receive the second dose of Ebola vaccine later than originally planned. This is because it was necessary to temporarily close the study clinics due to the COVID-19 outbreak. We want to understand if people who receive their second dose of vaccine later than two months have the same immune responses as the people who received their second dose of vaccine at two months after the first dose. To do this, we will need to collect blood samples from approximately 50 adults, 25 adolescents, and 25 children participating in the TUJIOKOWE study who received the second dose of the Ebola vaccine after July 2020. We are asking your child to join this small study (which we are calling the Immunogenicity Subset) and to provide two blood samples because your child will be receiving the second dose of the vaccine after July 2020. If you agree for your child to participate, one blood sample will be collected from your child before the second dose of Ebola vaccine is given and the second blood sample will be collected 21 days later.

For peer review only - http://bmjopen.bmj.com/site/about/guidelines.xhtml

We would like to be sure that your child's body responds to the vaccine as well as the children who received the second dose of the vaccine two months after the first dose. To check this, we will be looking at the antibodies that your child's body produces after he/she receives a vaccine. Antibodies are produced by your body after vaccination to protect you from infection. The information from testing your child's blood for antibodies will be very important when the vaccine is used to protect against Ebola in the future because it may not always be possible to give the second dose at exactly two months after the first dose of the vaccine.

If you are the parent or guardian of a child under 18 years of age and you agree to your child providing blood samples, you will need to sign separate documents for you and your child. If your child is aged 12 to 17 years old, he/she will also need to agree to provide blood samples himself/herself and sign a different document, called the immunogenicity assent form.

DOES YOUR CHILD HAVE TO GIVE BLOOD SAMPLES?

Your child does not have to provide any blood samples. If you agree to your child providing blood samples, we will ask you to sign this consent form. If you do not want your child to give any blood samples, this will not affect your child's participation in the TUJIOKOWE study.

WHAT WILL HAPPEN IF I AGREE TO MY CHILD GIVING BLOOD SAMPLES?

We will describe the blood collection process and answer any questions that you may have. If you would like a copy of the written information, we will give it to you. You will be asked to sign or put your fingerprint on this consent form. Putting your name or your fingerprint on the consent form means that you agree to your child providing blood samples, but you can change your mind at any time.

After your child has given his/her first blood sample , your child will follow the normal TUJIOKOWE study procedures to receive the second dose of vaccine. To have your child's second blood sample taken, you will need to bring your child to the study clinic for an extra visit 21 days after your child received the second injection of vaccine. If you agree to your child participating in the Immunogenicity Subset, then we will give your child some refreshments at the clinic.

WHAT DOES GIVING BLOOD INVOLVE?

A small amount of your child's blood will be collected from your child using a small clean needle. We will take blood before your child gets the second injection of vaccine and at 21 days after the second injection. To have your child's second blood sample taken your child will need to come to the study clinic for an extra visit 21 days after your child has received the second dose of vaccine. For children aged 6 to 17 years, we will collect approximately 5 mL (about 1 teaspoon) of blood each time. For young children aged 4 to 5 years, we will only collect 2.5 mL of blood each time.

As explained to you earlier, the blood samples your child gives will be used to test whether your child's body has produced antibodies in his/her blood against Ebola after vaccination. You

For peer review only

For peer review only - http://bmjopen.bmj.com/site/about/guidelines.xhtml

and your child will not receive the results of these tests because they are only for scientific research.

After we do our tests on your child's blood samples for the Immunogenicity Subset of the TUJIOKOWE study, we will destroy any remaining blood that has been collected from your child. We will not use your child's blood for any other purpose except the antibody tests that we have explained in this consent form. Your child's blood samples will not be sold.

Your child's blood samples will be labelled with a code and not his/her name. Other information, such as sex, age, or health history might also be shared with other investigators, but your child's name will not be shared.

WHAT ARE THE POSSIBLE RISKS OF GIVING A BLOOD SAMPLE?

Providing a blood sample is generally safe but occasionally people experience pain, bruising, bleeding, or (very rarely) infection from giving a blood sample. The medical team will provide care if your child experiences side-effects from the blood sampling. They are available if your child has any problems or if you have any questions.

WHAT ARE THE BENEFITS OF GIVING A BLOOD SAMPLE?

There is no direct benefit to your child for providing blood samples in the Immunogenicity Subset of the TUJIOKOWE study. Your agreement for your child to provide blood samples will help in the development of vaccines to prevent Ebola and, in the future, may help people in different parts of the world.

WHAT HAPPENS TO THE BLOOD SAMPLES COLLECTED FROM MY CHILD?

The blood samples collected from your child during the Immunogenicity Subset of the TUJIOKOWE study, will only be used to understand how the Ebola vaccine works when the second dose is given more than two months after the first dose.

The samples will be coded in a way that only limited study staff can link them to your child. Your child's blood samples will be tested in a laboratory in the United States. If any blood remains in DRC, it will be stored in the INRB laboratory in Goma. We will destroy all the samples in the United States and in Goma at the end of the study.

For peer review only - http://bmjopen.bmj.com/site/about/guidelines.xhtml

WHO CAN I TALK TO ABOUT MY CHILD GIVING BLOOD SAMPLES?

If you want to talk to someone about your child giving blood samples or if you think your child has been harmed by providing a blood sample, you can contact the following people:

1) The Principal Investigator responsible for this study

Professor Jean Jacques Muyembe<br>*Director General*, Institut National de Recherche Biomédicale, Kinshasa, DRC<br>Phone: 0898 949 289<br>Email: jjmuyembet@gmail.com<br><br>Local study representative<br>Dr Hugo Kavunga<br>Phone: 0823 875 153<br>Email: hugokavunga@gmail.com

2) The DRC ethics committees that approved this study

Professeur Félicien Munday<br>National Ethics Committee<br>Kinshasa-Gombe, DRC<br>Phone: 0998 419 816<br>Email: feli1munday@yahoo.fr
Professeur Willy Bongopasi<br>Comité d'éthique de l'école de santé publique<br>Université de Kinshasa<br>Phone: 0999 952 341<br>Email: bongopasi@gmail.com

If you have any questions about your child providing blood samples for the Immunogenicity Subset of the TUJIOKOWE study or about your child's rights, you may ask anyone on the study team at any time.

For peer review only - http://bmjopen.bmj.com/site/about/guidelines.xhtml

PARTICIPANT IMMUNOGENICITY SUBSET CONSENT FORM

Title: Immunogenicity Subset of the TUJIOKOWE Study

Principal Investigator of this study: Prof. JJ Muyembe

**Statements**	**Please sign or fingerprint each box**
I have read the information in this form about my child giving a blood sample at two visits for the Immunogenicity Subset of the TUJIOKOWE Study (or I have had this information explained to me by the study staff in a language that I understand). The purpose of providing blood samples and the procedures to give blood samples have been fully explained to me. I was able to ask questions and have all of these questions answered to my satisfaction.	
I understand that my child's participation is voluntary, that my child can withdraw consent to provide blood samples at any time without giving any reason, and that this will not affect my child's participation in the TUJIOKOWE Study.	

I am the parent or guardian of a participant aged 4 to 17 years and I agree for my child to provide blood samples during his/her participation in the TUJIOKOWE study.
(Please sign or put your fingerprint below)

		____/____/_____<br>*dd mon yyyy*
Printed name of the parent or guardian	Signature/fingerprint of the parent or guardian	Date

Printed name of the child participant (if aged 4-17)

For peer review only - http://bmjopen.bmj.com/site/about/guidelines.xhtml

		____/____/_____ *dd mon yyyy*
Printed name of investigator	Signature of investigator	Date

Complete next section if the parent/guardian is illiterate:

Witness to Consent Interview

I witnessed the consent interview for the Immunogenicity Subset of the TUJIOKOWE Study in this document. I attest that I have explained the study information accurately to the parent/guardian and was understood to the best of my knowledge by the parent/guardian, and that he/she has freely given their consent for his/her child to participate in my presence.

		____/____/_____ *dd mon yyyy*
Printed name of impartial witness	Signature of impartial witness	Date

Attach ID barcode label below:

For peer review only - http://bmjopen.bmj.com/site/about/guidelines.xhtml

Protocol for a phase 3 trial to evaluate the effectiveness and safety of a heterologous, two-dose vaccine for Ebola virus disease in the Democratic Republic of the Congo

Appendix 5B

Immunogenicity Informed Consent Form for Parents in French, Version 1.1 (10 Aug 2020)

For peer review only

For peer review only - http://bmjopen.bmj.com/site/about/guidelines.xhtml

Évaluation de l'efficacité et de la sécurité d'emploi d'un vaccin préventif hétérologue à deux doses contre Ebola en République démocratique du Congo

Note d'information et formulaire de consentement éclairé pour le sous-groupe Immunogénicité de l'« étude TUJIOKOWE » destinés aux parents/tuteurs des participants pédiatriques

Protocole LSHTM : DRC-EB-001

Investigateur principal (IP) :
Professeur Jean Jacques Muyembe
Directeur général
Institut national de recherche biomédicale
Professeur de microbiologie, Faculté de médecine de l'Université de Kinshasa
Kinshasa Gombe, République démocratique du Congo (RDC)
Téléphone : 0898 949 289 ; Adresse électronique : jjmuyembet@gmail.com

Promoteur : London School of Hygiene & Tropical Medicine, Royaume-Uni

Centre : République démocratique du Congo

INTRODUCTION

L'« étude TUJIOKOWE » est une étude de recherche visant à déterminer si un nouveau vaccin à deux doses appelé « vaccin contre le virus Ebola de Janssen » peut protéger contre le virus Ebola et à vérifier si le vaccin est sans danger. L'étude TUJIOKOWE est mise en œuvre par le ministère de la Santé de la RDC par l'intermédiaire de l'Institut national de recherche biomédicale (INRB), Epicentre et la London School of Hygiene & Tropical Medicine.

Lorsque nous avons commencé l'étude TUJIOKOWE, nous avions prévu d'administrer la seconde dose du vaccin contre le virus Ebola à tous les participants 2 mois environ après la première dose. Certaines personnes vont maintenant recevoir la seconde dose du vaccin contre le virus Ebola plus tard que prévu. Cela s'explique par la nécessité de fermer temporairement les centres de l'étude en raison de l'épidémie de COVID-19. Nous voulons comprendre si les personnes qui reçoivent leur seconde dose de vaccin plus de 2 mois après présentent la même réponse immunitaire que les personnes qui l'ont reçue 2 mois après la première dose. À cet effet, nous allons devoir prélever des échantillons de sang auprès de 50 adultes, 25 adolescents et 25 enfants environ participant à l'étude TUJIOKOWE, qui ont reçu la seconde dose du vaccin contre le virus Ebola après juillet 2020. Nous demandons à votre enfant de participer à cette petite étude (que nous appelons « sous-groupe Immunogénicité ») et de fournir deux échantillons de sang, car votre enfant va recevoir la seconde dose du vaccin après juillet 2020. Si vous acceptez que votre enfant participe à l'étude, un échantillon de sang lui sera prélevé

For peer review only - http://bmjopen.bmj.com/site/about/guidelines.xhtml

avant l'administration de la seconde dose du vaccin contre le virus Ebola et le second échantillon de sang sera prélevé 21 jours plus tard.

Nous voulons nous assurer que l'organisme de votre enfant réagit au vaccin aussi bien que celui des enfants qui ont reçu la seconde dose du vaccin 2 mois après la première dose. À cet effet, nous allons rechercher les anticorps que produit l'organisme de votre enfant après l'administration du vaccin. Les anticorps sont produits par l'organisme après la vaccination pour vous protéger contre l'infection. Les informations obtenues en analysant le sang de votre enfant pour rechercher la présence d'anticorps seront très importantes lorsque le vaccin sera utilisé pour protéger contre le virus Ebola à l'avenir, car il ne sera peut-être pas toujours possible d'administrer la seconde dose du vaccin 2 mois exactement après la première dose.

Si vous êtes le parent ou le tuteur d'un enfant de moins de 18 ans et si vous acceptez qu'il fournisse les échantillons de sang, vous devrez signer des documents distincts pour vous et votre enfant. Si votre enfant est âgé de 12 à 17 ans, il devra également accepter de lui-même de fournir les échantillons de sang et signer un autre document, appelé « formulaire d'assentiment » pour le sous-groupe Immunogénicité.

VOTRE ENFANT EST-IL OBLIGÉ DE DONNER DES ÉCHANTILLONS DE SANG ?

Votre enfant n'est pas obligé de fournir des échantillons de sang. Si vous acceptez que votre enfant fournisse des échantillons de sang, nous vous inviterons à signer ce formulaire de consentement. Si vous ne souhaitez pas que votre enfant donne des échantillons de sang, cela ne remettra pas en cause sa participation à l'étude TUJIOKOWE.

QUE SE PASSERA-T-IL SI J'ACCEPTE QUE MON ENFANT DONNE DES ÉCHANTILLONS DE SANG ?

Nous allons décrire les modalités de prise de sang et répondre à toutes les questions que vous pourriez avoir. Si vous souhaitez une copie de cette note d'information, nous vous la remettrons. Nous vous inviterons à signer ce formulaire de consentement ou à y apposer votre empreinte digitale. En inscrivant votre nom ou en apposant votre empreinte digitale sur le formulaire de consentement, vous acceptez que votre enfant fournisse des échantillons de sang, mais vous pourrez changer d'avis à tout moment.

Une fois que votre enfant aura donné son premier échantillon de sang, il suivra les procédures habituelles de l'étude TUJIOKOWE pour recevoir la seconde dose de vaccin. Pour le prélèvement du second échantillon de sang de votre enfant, vous devrez amener votre enfant au centre de l'étude pour une visite supplémentaire 21 jours après l'administration de la seconde injection du vaccin à votre enfant. Si vous acceptez que votre enfant participe au sous-groupe Immunogénicité, chaque fois qu'il donnera son sang, nous lui donnerons des rafraîchissements.

For peer review only - http://bmjopen.bmj.com/site/about/guidelines.xhtml

EN QUOI CONSISTE LE PRELEVEMENT DE SANG ?

Une petite quantité de sang sera prélevée à votre enfant à l'aide d'une petite aiguille propre. Nous lui prélèverons un échantillon de sang avant qu'il reçoive la seconde injection du vaccin et 21 jours après la seconde injection. Pour le prélèvement du second échantillon de sang de votre enfant, il devra venir effectuer une visite supplémentaire au centre de l'étude 21 jours après l'administration de la seconde dose de vaccin. Pour les enfants âgés de 6 à 17 ans, nous prélèverons 5 ml environ (soit 1 cuillère à café environ) de sang à chaque fois. Pour les jeunes enfants âgés de 4 à 5 ans, nous ne prélèverons que 2,5 ml de sang à chaque fois.

Comme indiqué précédemment, les échantillons de sang que votre enfant donnera serviront à vérifier si son organisme a produit des anticorps dans son sang contre le virus Ebola après la vaccination. Vous et votre enfant ne recevrez pas les résultats de ces analyses, car ils sont destinés uniquement à la recherche scientifique.

Après avoir réalisé nos analyses sur les échantillons de sang de votre enfant pour le sous-groupe Immunogénicité de l'étude TUJIOKOWE, nous détruirons tous les échantillons de sang restants prélevés à votre enfant. Nous n'utiliserons pas les échantillons de sang de votre enfant à d'autres fins que les analyses de détection d'anticorps mentionnés dans ce formulaire de consentement. Les échantillons de sang de votre enfant ne seront pas vendus.

Les échantillons de sang de votre enfant seront étiquetés avec un code et non avec son nom. D'autres informations, comme le sexe, l'âge ou les antécédents médicaux, pourront également être partagées avec d'autres investigateurs, mais le nom de votre enfant ne sera pas communiqué.

QUELS SONT LES RISQUES EVENTUELS LIES AU DON D'UN ECHANTILLON DE SANG ?

Le prélèvement d'un échantillon de sang est généralement sans danger, mais il arrive que des personnes ressentent des douleurs ou présentent un bleu, un saignement ou (dans de très rares cas) une infection après un prélèvement de sang. Si votre enfant présente des effets secondaires après la prise de sang, l'équipe médicale lui apportera les soins nécessaires. L'équipe est disponible à tout moment si votre enfant a des problèmes ou des questions.

QUELS SONT LES BENEFICES LIES AU DON D'UN ECHANTILLON DE SANG ?

Il n'y a aucun bénéfice direct pour votre enfant à fournir des échantillons de sang dans le cadre du sous-groupe Immunogénicité de l'étude TUJIOKOWE. Le consentement de votre enfant à fournir des échantillons de sang contribuera au développement de vaccins pour prévenir le virus Ebola et, à l'avenir, pourrait aider des personnes dans différentes régions du monde.

QU'ADVIENDRA-IL DES ECHANTILLONS DE SANG PRELEVES A MON ENFANT ?

Les échantillons de sang prélevés à votre enfant dans le cadre du sous-groupe Immunogénicité de l'étude TUJIOKOWE ne seront utilisés que pour comprendre l'efficacité du vaccin contre le virus Ebola lorsque la seconde dose est administrée plus de 2 mois après la première dose.

For peer review only - http://bmjopen.bmj.com/site/about/guidelines.xhtml

Les échantillons seront codés de manière à limiter le nombre de membres du personnel de l'étude qui peut les relier à votre enfant. Les échantillons de sang de votre enfant seront analysés dans un laboratoire aux États-Unis. S'il reste des échantillons de sang en RDC, ils seront conservés dans le laboratoire de l'INRB à Goma. Nous détruirons tous les échantillons conservés aux États-Unis et à Goma à la fin de l'étude.

À QUI PUIS-JE M'ADRESSER CONCERNANT LE PRÉLÈVEMENT D'ÉCHANTILLONS DE SANG À ENFANT ?

Si vous souhaitez parler à quelqu'un au sujet du prélèvement d'échantillons de sang à votre enfant ou si vous pensez que votre enfant a subi un préjudice en fournissant un échantillon de sang, vous pouvez contacter les personnes suivantes :

1) Investigateur principal responsable de cette étude

Professeur Jean-Jacques Muyembe<br>*Directeur général*, Institut national de recherche biomédicale, Kinshasa, RDC<br>Téléphone : 0898 949 289<br>Adresse électronique : jjmuyembet@gmail.com<br><br>Représentant locale de l'étude<br>Dr Hugo Kavunga<br>Téléphone : 0823 875 153<br>Adresse électronique : hugokavunga@gmail.com

2) Comités d'éthique de la RDC ayant approuvé cette étude

Professeur Félicien Munday<br>Comité national d'éthique et de la santé<br>Kinshasa-Gombe, RDC<br>Téléphone : 0998 419 816<br>Adresse électronique : feli1munday@yahoo.fr
Professeur Willy Bongopasi<br>Comité d'éthique de l'école de santé publique<br>Université de Kinshasa<br>Téléphone: 0999 952 341<br>Adresse électronique : bongopasi@gmail.com

Si vous avez des questions sur le prélèvement d'échantillons de sang à votre enfant pour le sous-groupe Immunogénicité de l'étude TUJIOKOWE ou sur les droits de votre enfant, vous pouvez les poser à tout moment à n'importe quel membre de l'équipe de l'étude.

For peer review only - http://bmjopen.bmj.com/site/about/guidelines.xhtml

FORMULAIRE DE CONSENTEMENT DES PARTICIPANTS POUR LE SOUS-GROUPE IMMUNOGÉNICITÉ

Titre : Sous-groupe Immunogénicité de l'étude TUJIOKOWE

Investigateur principal de cette étude : Prof. JJ Muyembe

**Déclarations**	**Signez ou apposez votre empreinte digitale dans chaque case**
J'ai lu les informations contenues dans ce formulaire sur le prélèvement d'un échantillon de sang à mon enfant lors de deux visites pour le sous-groupe Immunogénicité de l'étude TUJIOKOWE (ou le personnel de l'étude m'a expliqué ces informations avec des mots que je comprends). L'objectif du prélèvement d'échantillons de sang et les procédures correspondantes m'ont été expliqués en détail. J'ai pu poser des questions et j'ai reçu des réponses satisfaisantes à toutes mes questions.	
Je comprends que la participation de mon enfant est volontaire, que mon enfant peut retirer son consentement à fournir des échantillons de sang à tout moment sans avoir à se justifier et que cela ne remettra pas en cause sa participation à l'étude TUJIOKOWE.	

Je suis le parent ou le tuteur d'un participant âgé de 4 à 17 ans et j'accepte que mon enfant fournisse des échantillons de sang pendant sa participation à l'étude TUJIOKOWE. (Signez ou apposez votre empreinte digitale ci-dessous)

		____/____/_____ *jj mmm aaaa*
Nom du parent ou tuteur (en caractères d'imprimerie)	Signature/Empreinte digitale du parent ou tuteur	Date

Nom de l'enfant participant (si âgé de 4 à 17 ans ; en caractères d'imprimerie)

For peer review only - http://bmjopen.bmj.com/site/about/guidelines.xhtml

		____/____/_____ *jj mmm aaaa*
Nom de l'investigateur (en caractères d'imprimerie)	Signature de l'investigateur	Date

---

Si le parent/tuteur est analphabète, remplissez cette section :

Témoin de l'entretien de consentement

J'ai assisté à l'entretien de consentement pour le sous-groupe Immunogénicité de l'étude TUJIOKOWE décrite dans ce document. J'atteste avoir expliqué avec exactitude les informations sur l'étude au parent/tuteur et que, à ma connaissance, cette personne les a comprises et a donné librement son consentement pour que son enfant participe, en ma présence.

		____/____/_____ *jj mmm aaaa*
Nom du témoin impartial (en caractères d'imprimerie)	Signature du témoin impartial	Date

---

Apposez l'étiquette d'identification par code à barres ci-dessous :

For peer review only - http://bmjopen.bmj.com/site/about/guidelines.xhtml

Protocol for a phase 3 trial to evaluate the effectiveness and safety of a heterologous, two-dose vaccine for Ebola virus disease in the Democratic Republic of the Congo

Appendix 6

Data and Safety Monitoring Board (DSMB)

The following will be invited to participate on the Data and Safety Monitoring Board (DSMB) of this study. For detailed description of the DSMB responsibilities and activities, refer to the latest DSMB Charter.

Point of contact: Yazdan Yazdanpanah

Yazdan Yazdanpanah<br>Adult Clinician<br>yazdan.yazdanpanah@aphp.fr	Matthias Egger<br>Methodologist (DSMB Statistician)<br>matthias.egger@ispm.unibe.ch
Ebunoluwa Adejuyigbe<br>Paediatrician<br>ebunadejuyigbe@hotmail.com	Florian Marks<br>Vaccinologist/Epidemiologist<br>fmarks@ivi.int
Benoit Kebela Ilunga<br>Epidemiologist<br>kebelailunga@gmail.com	Tamara Giles-Vernick<br>Social Scientist<br>tamara.giles-vernick@pasteur.fr
Oumou Younoussa Bah Sow<br>Ethicist; President of the National Committee of Ethics for Health Research (CNERS)<br>oumou45@yahoo.fr	Marie Onyamboko<br>Epidemiologist; Kinshasa School of Public Health<br>akatshimarie@yahoo.fr

Trial Steering Committee (TSC)

The TSC is responsible for conducting the clinical study and for solving strategic problems that require high-level interventions.

Point of Contact: Richard Hatchett

Richard Hatchett/Gerald Voss<br>Coalition for Epidemic Preparedness Innovations<br>Richard.Hatchett@cepi.net<br>gerald.voss@cepi.net	Sylvain Yuma<br>Ministère de la Santé, DRC<br>sylvainyuma@gmail.com
Daniel Bausch<br>UK Public Health Rapid Support Team/LSHTM<br>Daniel.Bausch@phe.gov.uk	Jean-Jacques Muyembe-Tamfum (and Steve Ahuka)<br>Institut National de Recherche Biomédicale, DRC<br>jjmuyembet@gmail.com (amstev04@yahoo.fr)
Mike Ryan/Ana Maria Henao-Restrepo<br>World Health Organization<br>ryanm@who.int; henaorestrepoa@who.int	Jeremy Farrar/Josie Golding<br>Wellcome Trust<br>J.Farrar@wellcome.ac.uk<br>J.Golding@wellcome.ac.uk

For peer review only

Peter Piot/Deborah Watson-Jones<br>London School of Hygiene & Tropical Medicine<br>Peter.Piot@lshtm.ac.uk;<br>deborah.watson-jones@lshtm.ac.uk	Johan Van Hoof (and Macaya Douoguih)<br>Janssen Pharmaceuticals R&D<br>JVHOOF1@its.jnj.com (MDouogui@its.jnj.com)
Rebecca F Grais<br>Director of Research<br>Epicentre MSF, Paris, France<br>rebecca.grais@epicentre.msf.org	Isabelle Defourny/Clair Mills<br>MSF Operational Centre Paris<br>Médecins Sans Frontières, Paris, France<br>Isabelle.defourny@paris.msf.org;<br>Clair.Mills@paris.msf.org
Robert Kanwagi<br>World Vision<br>Robert.kanwagi@wveu.org	

For peer review only

Protocol for a phase 3 trial to evaluate the effectiveness and safety of a heterologous, two-dose vaccine for Ebola virus disease in the Democratic Republic of the Congo

Appendix 7

Sponsor and Funders Responsibilities

Funders CEPI, Wellcome and DFID advised on the study design and protocol.

	Sponsor	Funders
Study design	A/R	R
Data collection	A/R	I
Data management	A/R	I
Data analysis	A/R	I
Data interpretation	A/R	I
Report writing	A/R	C
Report submission for publication	A/R	C

A = Accountable
R = Responsible
C = Contributing
I = Informed

For peer review only

Reporting checklist for protocol of a clinical trial.

Based on the SPIRIT guidelines.

Instructions to authors

Complete this checklist by entering the page numbers from your manuscript where readers will find each of the items listed below.

Your article may not currently address all the items on the checklist. Please modify your text to include the missing information. If you are certain that an item does not apply, please write "n/a" and provide a short explanation.

Upload your completed checklist as an extra file when you submit to a journal.

In your methods section, say that you used the SPIRITreporting guidelines, and cite them as:

Chan A-W, Tetzlaff JM, Gøtzsche PC, Altman DG, Mann H, Berlin J, Dickersin K, Hróbjartsson A, Schulz KF, Parulekar WR, Krleža-Jerić K, Laupacis A, Moher D. SPIRIT 2013 Explanation and Elaboration: Guidance for protocols of clinical trials. BMJ. 2013;346:e7586

		Reporting Item	Page Number
**Administrative information**			
Title	#1	Descriptive title identifying the study design, population, interventions, and, if applicable, trial acronym	5
Trial registration	#2a	Trial identifier and registry name. If not yet registered, name of intended registry	2
Trial registration: data set	#2b	All items from the World Health Organization Trial Registration Data Set	21, Table 1
Protocol version	#3	Date and version identifier	17
Funding	#4	Sources and types of financial, material, and other support	20
Roles and responsibilities: contributorship	#5a	Names, affiliations, and roles of protocol contributors	18-19

For peer review only

Roles and responsibilities: sponsor contact information	#5b	Name and contact information for the trial sponsor	21, Table 1
Roles and responsibilities: sponsor and funder	#5c	Role of study sponsor and funders, if any, in study design; collection, management, analysis, and interpretation of data; writing of the report; and the decision to submit the report for publication, including whether they will have ultimate authority over any of these activities	Appendix 7
Roles and responsibilities: committees	#5d	Composition, roles, and responsibilities of the coordinating centre, steering committee, endpoint adjudication committee, data management team, and other individuals or groups overseeing the trial, if applicable (see Item 21a for data monitoring committee)	16, Appendix 6
**Introduction**			
Background and rationale	#6a	Description of research question and justification for undertaking the trial, including summary of relevant studies (published and unpublished) examining benefits and harms for each intervention	4-6
Background and rationale: choice of comparators	#6b	Explanation for choice of comparators	6
Objectives	#7	Specific objectives or hypotheses	5
Trial design	#8	Description of trial design including type of trial (eg, parallel group, crossover, factorial, single group), allocation ratio, and framework (eg, superiority, equivalence, non-inferiority, exploratory)	6
**Methods: Participants, interventions, and outcomes**			
Study setting	#9	Description of study settings (eg, community clinic, academic hospital) and list of countries where data will be collected. Reference to where list of study sites can be obtained	7

For peer review only

Eligibility criteria	#10	Inclusion and exclusion criteria for participants. If applicable, eligibility criteria for study centres and individuals who will perform the interventions (eg, surgeons, psychotherapists)	8, 24, Box 1
Interventions: description	#11a	Interventions for each group with sufficient detail to allow replication, including how and when they will be administered	5
Interventions: modifications	#11b	Criteria for discontinuing or modifying allocated interventions for a given trial participant (eg, drug dose change in response to harms, participant request, or improving / worsening disease)	10
Interventions: adherance	#11c	Strategies to improve adherence to intervention protocols, and any procedures for monitoring adherence (eg, drug tablet return; laboratory tests)	12,13
Interventions: concomitant care	#11d	Relevant concomitant care and interventions that are permitted or prohibited during the trial	24, Box 1
Outcomes	#12	Primary, secondary, and other outcomes, including the specific measurement variable (eg, systolic blood pressure), analysis metric (eg, change from baseline, final value, time to event), method of aggregation (eg, median, proportion), and time point for each outcome. Explanation of the clinical relevance of chosen efficacy and harm outcomes is strongly recommended	13-15
Participant timeline	#13	Time schedule of enrolment, interventions (including any run-ins and washouts), assessments, and visits for participants. A schematic diagram is highly recommended (see Figure)	10-12, 23, Table 2
Sample size	#14	Estimated number of participants needed to achieve study objectives and how it was determined, including clinical and statistical assumptions supporting any sample size calculations	7, 8, 25, Table 3
Recruitment	#15	Strategies for achieving adequate participant enrolment to reach target sample size	8, 9
**Methods: Assignment of interventions (for controlled trials)**			

For peer review only

Allocation: sequence generation	#16a	Method of generating the allocation sequence (eg, computer-generated random numbers), and list of any factors for stratification. To reduce predictability of a random sequence, details of any planned restriction (eg, blocking) should be provided in a separate document that is unavailable to those who enrol participants or assign interventions	N/A
Allocation concealment mechanism	#16b	Mechanism of implementing the allocation sequence (eg, central telephone; sequentially numbered, opaque, sealed envelopes), describing any steps to conceal the sequence until interventions are assigned	N/A
Allocation: implementation	#16c	Who will generate the allocation sequence, who will enrol participants, and who will assign participants to interventions	N/A
Blinding (masking)	#17a	Who will be blinded after assignment to interventions (eg, trial participants, care providers, outcome assessors, data analysts), and how	N/A
Blinding (masking): emergency unblinding	#17b	If blinded, circumstances under which unblinding is permissible, and procedure for revealing a participant's allocated intervention during the trial	N/A
**Methods: Data collection, management, and analysis**			
Data collection plan	#18a	Plans for assessment and collection of outcome, baseline, and other trial data, including any related processes to promote data quality (eg, duplicate measurements, training of assessors) and a description of study instruments (eg, questionnaires, laboratory tests) along with their reliability and validity, if known. Reference to where data collection forms can be found, if not in the protocol	14, 17
Data collection plan: retention	#18b	Plans to promote participant retention and complete follow-up, including list of any outcome data to be collected for participants who discontinue or deviate from intervention protocols	9, 17
Data management	#19	Plans for data entry, coding, security, and storage, including any related processes to promote data quality (eg, double data	14

For peer review only

		entry; range checks for data values). Reference to where details of data management procedures can be found, if not in the protocol	
Statistics: outcomes	#20a	Statistical methods for analysing primary and secondary outcomes. Reference to where other details of the statistical analysis plan can be found, if not in the protocol	15
Statistics: additional analyses	#20b	Methods for any additional analyses (eg, subgroup and adjusted analyses)	16
Statistics: analysis population and missing data	#20c	Definition of analysis population relating to protocol non-adherence (eg, as randomised analysis), and any statistical methods to handle missing data (eg, multiple imputation)	16
**Methods: Monitoring**			
Data monitoring: formal committee	#21a	Composition of data monitoring committee (DMC); summary of its role and reporting structure; statement of whether it is independent from the sponsor and competing interests; and reference to where further details about its charter can be found, if not in the protocol. Alternatively, an explanation of why a DMC is not needed	16, Appendix 6
Data monitoring: interim analysis	#21b	Description of any interim analyses and stopping guidelines, including who will have access to these interim results and make the final decision to terminate the trial	N/A
Harms	#22	Plans for collecting, assessing, reporting, and managing solicited and spontaneously reported adverse events and other unintended effects of trial interventions or trial conduct	11-12, 23, Table 2
Auditing	#23	Frequency and procedures for auditing trial conduct, if any, and whether the process will be independent from investigators and the sponsor	16
**Ethics and dissemination**			
Research ethics approval	#24	Plans for seeking research ethics committee / institutional review board (REC / IRB) approval	17
Protocol amendments	#25	Plans for communicating important protocol modifications (eg, changes to eligibility criteria, outcomes, analyses) to	10, 17

For peer review only

		relevant parties (eg, investigators, REC / IRBs, trial participants, trial registries, journals, regulators)	
Consent or assent	#26a	Who will obtain informed consent or assent from potential trial participants or authorised surrogates, and how (see Item 32)	9
Consent or assent: ancillary studies	#26b	Additional consent provisions for collection and use of participant data and biological specimens in ancillary studies, if applicable	10-12
Confidentiality	#27	How personal information about potential and enrolled participants will be collected, shared, and maintained in order to protect confidentiality before, during, and after the trial	13,14
Declaration of interests	#28	Financial and other competing interests for principal investigators for the overall trial and each study site	20
Data access	#29	Statement of who will have access to the final trial dataset, and disclosure of contractual agreements that limit such access for investigators	17
Ancillary and post trial care	#30	Provisions, if any, for ancillary and post-trial care, and for compensation to those who suffer harm from trial participation	12
Dissemination policy: trial results	#31a	Plans for investigators and sponsor to communicate trial results to participants, healthcare professionals, the public, and other relevant groups (eg, via publication, reporting in results databases, or other data sharing arrangements), including any publication restrictions	17
Dissemination policy: authorship	#31b	Authorship eligibility guidelines and any intended use of professional writers	17
Dissemination policy: reproducible research	#31c	Plans, if any, for granting public access to the full protocol, participant-level dataset, and statistical code	17
**Appendices**			
Informed consent materials	#32	Model consent form and other related documentation given to participants and authorised surrogates	Appendices 1-5
Biological specimens	#33	Plans for collection, laboratory evaluation, and storage of biological specimens for genetic or molecular analysis in the	10-11

For peer review only

current trial and for future use in ancillary studies, if applicable

None The SPIRIT Explanation and Elaboration paper is distributed under the terms of the Creative Commons Attribution License CC-BY-NC. This checklist can be completed online using https://www.goodreports.org/, a tool made by the EQUATOR Network in collaboration with Penelope.aib

For peer review only
